# Benchmarking short-read metagenomics tools for removing host contamination

**DOI:** 10.1093/gigascience/giaf004

**Published:** 2025-02-27

**Authors:** Yunyun Gao, Hao Luo, Hujie Lyu, Haifei Yang, Salsabeel Yousuf, Shi Huang, Yong-Xin Liu

**Affiliations:** Genome Analysis Laboratory of the Ministry of Agriculture and Rural Affairs, Agricultural Genomics Institute at Shenzhen, Chinese Academy of Agricultural Sciences, Shenzhen 518120, China; Genome Analysis Laboratory of the Ministry of Agriculture and Rural Affairs, Agricultural Genomics Institute at Shenzhen, Chinese Academy of Agricultural Sciences, Shenzhen 518120, China; Department of Life Sciences, Imperial College of London, London SW7 2AZ, UK; Genome Analysis Laboratory of the Ministry of Agriculture and Rural Affairs, Agricultural Genomics Institute at Shenzhen, Chinese Academy of Agricultural Sciences, Shenzhen 518120, China; College of Life Sciences, Qingdao Agricultural University, Qingdao 266000, China; Genome Analysis Laboratory of the Ministry of Agriculture and Rural Affairs, Agricultural Genomics Institute at Shenzhen, Chinese Academy of Agricultural Sciences, Shenzhen 518120, China; Faculty of Dentistry, The University of Hong Kong, Hong Kong SAR, China; Genome Analysis Laboratory of the Ministry of Agriculture and Rural Affairs, Agricultural Genomics Institute at Shenzhen, Chinese Academy of Agricultural Sciences, Shenzhen 518120, China

**Keywords:** metagenome, microbiome, host removal, microbial enrichment

## Abstract

**Background:**

The rapid evolution of metagenomic sequencing technology offers remarkable opportunities to explore the intricate roles of microbiome in host health and disease, as well as to uncover the unknown structure and functions of microbial communities. However, the swift accumulation of metagenomic data poses substantial challenges for data analysis. Contamination from host DNA can substantially compromise result accuracy and increase additional computational resources by including nontarget sequences.

**Results:**

In this study, we assessed the impact of computational host DNA decontamination on downstream analyses, highlighting its importance in producing accurate results efficiently. We also evaluated the performance of conventional tools like KneadData, Bowtie2, BWA, KMCP, Kraken2, and KrakenUniq, each offering unique advantages for different applications. Furthermore, we highlighted the importance of an accurate host reference genome, noting that its absence negatively affected the decontamination performance across all tools.

**Conclusions:**

Our findings underscore the need for careful selection of decontamination tools and reference genomes to enhance the accuracy of metagenomic analyses. These insights provide valuable guidance for improving the reliability and reproducibility of microbiome research.

## Graphical Abstract

**Graphical Abstract:**
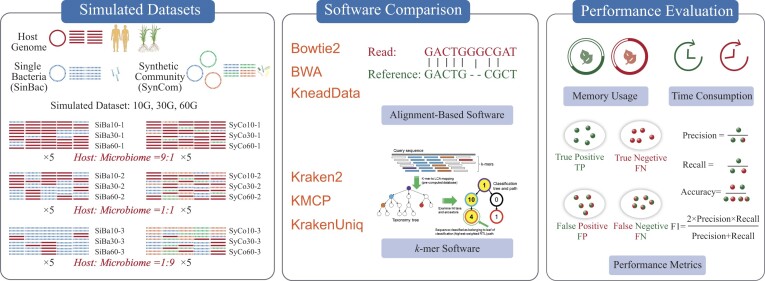

## Background

The advancement of second-generation sequencing technology and data analysis methods has greatly facilitated microbiome research, broadening our horizons in the widespread influences of microbiome on their host [1–4]. Compared to amplicon sequencing, shotgun metagenomic sequencing offers comprehensive assessments of bacterial communities with less bias and improved resolution in identifying profiles at the species, strain, or functional levels [5]. As sequencing technology costs rapidly decreased and sequencing depth continues to expand, the volume of metagenomic data is growing exponentially [6]. Some studies have even produced over 100 gigabase pairs (Gbps) per sample to characterize the dark matter of the microbiome in human gut [7]. However, a major challenge exists when analyzing the metagenome data from complex host-associated microbiomes, such as those found in saliva, throat, and vaginal swabs. These samples often contain over 90% human-aligned reads [5, 8, 9] due to the high contamination of host DNA. This contamination undermines the characterization of microbiomes, especially for low abundant species [8], leading to biased observations of the true underlying microbial composition. Additionally, privacy concerns have become particularly significant when the host is human [10], highlighting the importance of removing host contamination.

Despite efforts to remove host contamination during the experimental stage [9, 11–14], particularly in DNA isolation, residual DNA remains polluted with numerous host DNA fragments. The overall efficacy of different experimental protocols varies, and potential biases for the preferential enrichment of specific microbial taxa remain a concern. Even after significant host DNA reduction, high biomass samples like the mucosal microbiome can still exhibit up to 90% host contamination in metagenomic data [9, 11]. This persistence is due to differences in cell and genome size between animals and microbiomes. Similarly, in low biomass samples like endophytic microorganisms, around 70% of host sequencing reads may still be present despite efforts to collect and concentrate the microbiota [15]. This high level of contamination often necessitates deeper sequencing to adequately capture the microbial reads of interest. Sequencing these unwanted host DNA reads, followed by computational removal from large next-generation sequencing (NGS) datasets, is both wasteful and time-consuming [16]. It compromises the accuracy of downstream analyses and consumes valuable research time and computing resources. This underscores the importance of devising a host contamination removing tool in the data analysis stage that is both accurate and efficient [17].

After searching 2,853 publications using the keywords “metagenome” and “microbiome” (Supplementary Fig. S1A, Supplementary Table S1-1), we found that 57.94% of the studies addressed the removal of host contamination, with a discernible increasing trend from 2015 to 2024. The absence of standardized criteria for selecting host decontamination software has led to the use of 51 different tools, many relying on alignment and *k*-mer strategies. Among these, 10 tools exhibit notable popularity and generally employ 2 main strategies: alignment-based and the *k*-mer approaches [10, 18, 19]. The alignment software, such as Bowtie2 [20] and BWA [21], align sequencing reads to reference genomes. Kraken2 [22] and KMCP [23] are also popular *k*-mer–based software that identify exact matches between small substrings (*k*-mers) from the reads in the reference database. In addition, some host contamination removal pipelines integrate these modules. For instance, DeconSeq [24] integrates a modified version of BWA, while KneadData [25] integrates Bowtie2. Several new tools, like Hostile [10] and HoCoRT [18], have also been developed to enhance the accuracy of the host decontamination process. Although some studies have explored the impact of varying amounts of host DNA on microbiomes [26, 27], the impact of removing host DNA contamination on the bioinformatic downstream analysis and the microbial genome assembly remains unclear [13].

In this study, we will compare the efficiency of metagenomic sequencing on the microbiome by removing host contamination and thoroughly evaluate the accuracy and speed of state-of-the-art computational host DNA decontamination. These results will serve as a guide for researchers in rationally selecting suitable tools for processing various metagenomic datasets.

## Results

### High host contamination increased the processing time and skewed interpretation of the microbiome results

**Figure 1:**
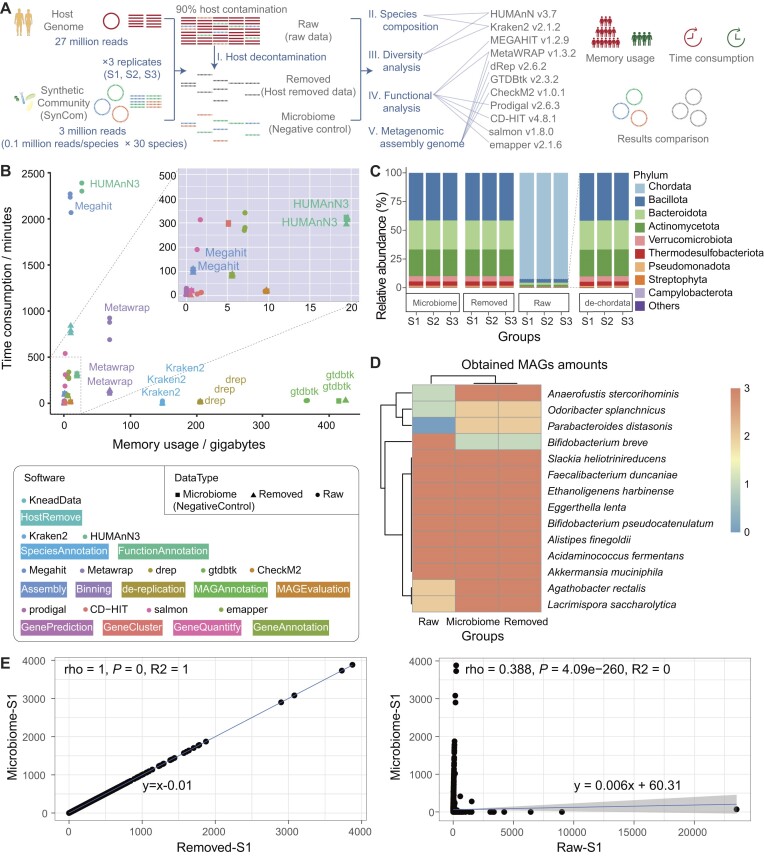
Host contamination consumed extra computing resources and affected the accuracy of the results in metagenomic analysis. (A) Simulated data design and the main downstream pipeline. Three samples (S1, S2, S3) with 90% host contamination were generated from the genomes of 30 bacteria and *Homo sapiens*. The raw data (Raw) underwent host decontamination to produce the removed data (Removed). The microbiome data were used as a negative control (Microbiome). Subsequent downstream analyses included host decontamination, species composition, diversity analysis, functional analysis, and metagenomic assembly genome evaluations. (B) Host contamination increased computing resource consumption by 7.63 to 20.55 times in Megahit and HUMAnN3. The performance in terms of time and memory usage during downstream analyses was assessed on 3 samples (∼9 GB per sample). (C) The relative abundance at the phylum level. We also displayed the composition of raw data without chordata (de-chordata) to demonstrate that host removal can accurately reflect the true microbiota composition. (D) Evaluation of metagenomic assembly genome (MAG) amounts. Removed data can generate more MAGs than raw data during binning in all samples. (E) Correlation assessment of Gene Ontology (GO) terms between microbiome data and removed data (left) or raw data (right) in the S1 group. Each step of analysis was based on 3 sample replicates, with each replicate consisting of 30 million paired-end 150-bp reads (∼9 GB).

Here we simulated 3 groups (S1, S2, S3) of data using CAMISIM with 90% host contamination (Supplementary Table S1-2), incorporating microbial reads from 30 species, each represented at equal abundance, along with human reads from *Homo sapiens* (GRCh38). We used KneadData, a popular host decontamination software in recent years, to remove host contamination from the raw data (Raw), resulting in host-removed data (Remove), while the 30 microbial groups served as a negative control (Microbiome). Our metagenomic analysis involved key steps such as species composition, diversity analysis, functional analysis, and metagenome-assembled genome (MAG) evaluations (Fig. 1A). Based on these simulations, we evaluated the impact of host contamination removal on metagenomic analysis of the microbiome (Fig. 1A), focusing on memory usage, processing time, and the effects on the accuracy of the results.

Throughout the analysis, no significant differences in memory usage were observed among the Raw data, Removed data, and Microbiome data during high-memory steps exceeding 100 gigabytes (GB), such as species-level taxonomic annotation (Kraken2), de-replication (drep), and MAG annotation (GTDBtk). However, compared to the Raw data, the host-read-removed data significantly reduced the runtime of downstream analyses (Fig. 1B). Specifically, processing the host removal data took 5.98 times shorter for binning (MetaWRAP), 7.63 times shorter for function annotation (HUMAnN3), and 20.55 times shorter for assembly (MEGAHIT). The average processing time for Remove data was 139.14 minutes (min) for MetaWRAP compared to 832.64 min for Raw data, 308.92 min for HUMAnN3 compared to 2,357.95 min for Raw data, and 106.59 min for MEGAHIT compared to 2,190.27 min for Raw data. Additionally, handling the negative control data (Microbiome) required similar resources to the host removal data in terms of both memory usage and time consumption.

Compared to Microbiome data, Raw data altered the relative abundance of microbiota community, while Remove data showed a similar composition to that annotated by Kraken2 (Fig. 1C). Interestingly, the remaining taxa were similar to the Microbiome data, after removing the Chordata (the phylum of *H. sapiens*) from the Raw data (Fig. 1C, de-chordata group). There was no difference in richness index between Microbiome data and Remove data, whereas Raw data showed a significantly lower richness index than both (Supplementary Fig. S2A). Principal coordinates analysis (PCoA) was performed to visualize changes in community composition, revealing that the first axes of PCoA explained 100% of the overall variations. This observation suggested low dimensionality and distinct separation of sample groups. Specifically, samples from Raw data were clearly separated from those of Microbiome and the Removed data along PCo1 (Supplementary Fig. S2B). This finding underscores the effectiveness of host decontamination in highlighting the underlying microbial community structure.

Despite simulating Metagenomic data from 30 microbial species, with each species having 0.1 million reads, only 14 MAGs were obtained. No significant differences were detected in completeness rate and contamination rates among the Microbiome data, Raw data, and Removed data (Supplementary Fig. S2C). However, the number of MAGs was much more in Microbiome and Removed data compared to Raw data, except for *Bifidobacterium breve*, which was detected in all 3 groups (S1, S2, S3) in Raw data but only in S2 in Microbiome and Removed data (Fig. 1D). Next, we compared the Gene Ontology (GO) terms to Microbiome data. We found a stronger correlation in GO terms between Removed data and Microbiome data than that between Raw data and Microbiome data (Fig. 1E, Supplementary Fig. S2D), indicating that the host removal process results in more specific gene function annotation.

### Kraken2 was a fast and low-resource tool for host removal

**Figure 2:**
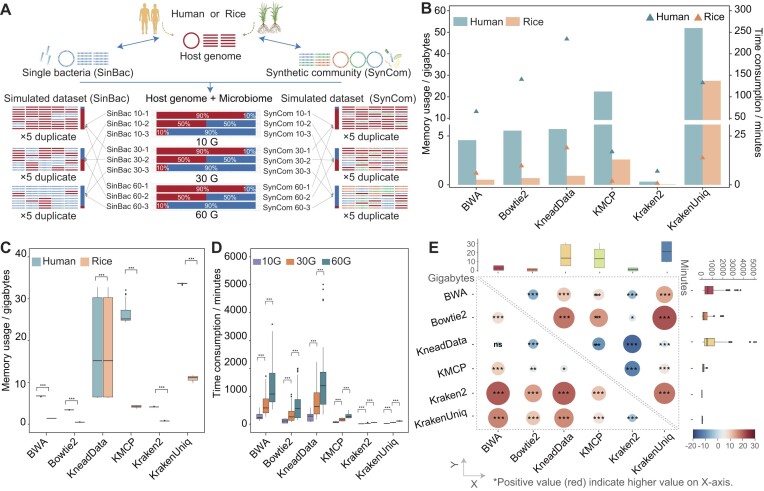
Benchmarking calculates resources of 6 host removal software on simulated human and rice metagenomic data. The computational resources of all software were mostly influenced by the host reference genome size and the metagenomic data size, with Kraken2 consistently utilizing minimal computational resources. (A) Simulating metagenome datasets using CAMISIM. The datasets were designed to encompass diverse scenarios, featuring varying proportions of host genome contamination. Derived from human or rice genomes, the datasets come in 3 different sizes, each containing either a single bacterium (SinBac) or a synthetic community (SynCom, detail in Supplementary Table S2-1, S2-2). (B) Comparison of time and memory usage in software for indexing the host reference genome. The size of the reference genome affects the resource consumption, with Kraken2 utilizing the fewest resources during the indexing step. (C) Memory usage for different software, measured in gigabytes (Gb). The maximum computational memory usage is influenced by the host reference genome, except in KneadData. (D) Running time of different software, shown in minutes. The decontamination process for large datasets requires more time. (E) Memory usage (top-right diagonal) and execution time (bottom-left diagonal) among different software based on the Kruskal–Wallis test. Positive values (red circle) indicate higher time or memory requirements for the software on the x-axis. The size of the circles represents the *z*-value, which is the standardized score corresponding to each pairwise comparison. Kraken2 was observed to use significantly lower time and memory usage compared to others. “*” is shown as significant difference. (ns, not significant; *P ≤ 0.05; **P ≤ 0.01; ***P ≤ 0.001.)

To further compare the differences in host removal among various software tools, we obtained 1,080 simulated metagenomic datasets, which include single bacterium (SinBac) and synthetic community (SynCom) across various sizes (10 Gbps, 30 Gbps, and 60 Gbps). The simulations were conducted separately for human (*H. sapiens*) and rice (*Oryza sativa indica*) hosts, each with 90%, 50%, and 10% levels of host contamination (Fig. 2A; see Methods for more details). For convenience, we have assigned abbreviations to various datasets. For example, SinBac10-1 refers to a 10-Gbps dataset with 90% host genome reads and 10% reads from a single bacterium genome. Similarly, SynCom 30-2 represented a 30-Gbps dataset with 50% host genome reads and 50% reads from the synthetic community genome. SynCom 60-3 refers to a 60-Gbps dataset with 10% host genome reads and 90% reads from the synthetic community genome (Fig. 2A). Based on these simulated data, we compared the computational resources required and host decontamination performance of 6 existing tools: KneadData, Bowtie2, and BWA (for alignment-based software) and KMCP, Kraken2, and KrakenUniq (for *k*-mer strategy software).

Before removing host contamination, indexing of host reference genomes is crucial. In this study, we constructed reference genomes for *H. sapiens* (GRCh38) and *O. sativa indica* (GWHBFPX00000000) with sizes approximately 3.1 Gbps and 373.8 Megabase pairs (Mbps), respectively. Kraken2 utilized minimal computational resources for indexing both human and rice genomes, requiring only 0.3 gigabyte (Gb) memory and taking 6.94 min to create a custom database for the human genome (Fig. 2B). In contrast, the other 5 tools required an average of 18.05 Gb memory and 117.98 min.

We then compared the resource consumption during the host contamination removal process across 6 software. In summary, Bowtie2 (1.95 Gb [0.410, 3.42]) and Kraken2 (2.47 Gb [0.710, 4.12]) demonstrated the lowest maximum memory usage across all simulated datasets for alignment and *k*-mer–based software, respectively (Fig. 2C, Supplementary Fig. S3). These values were significantly lower than that of the other 4 tools, with BWA requiring 3.995 Gb (1.40, 6.74), KneadData requiring 15.17 Gb (6.47, 30.27), KMCP requiring 14.45 Gb (4.27, 25.110), and KrakenUniq requiring 22.410 Gb (11.13, 33.67). Regarding data size, handling 60 Gbps data consumed significantly more resources than 10 Gbps data in KneadData and Kraken2. Different host types also significantly influenced resource consumption across all software (Supplementary Table S3-1), with human data requiring notably more resources than rice data (*P <* 0.05), whereas there was no significant difference between the different microbiome types for all software, except for KrakenUniq (Supplementary Table S3-1).

For time usage, the *k*-mer software (KMCP, 156.10 min [90.47, 231.16]; Kraken2, 29.34 min [13.42, 55.45]; and KrakenUniq, 59.23 min [26.66, 98.14]) required less time than the alignment-based software (BWA, 582.26 min [300.15, 1,065.64]; Bowtie2, 209.00 min [111.66, 512.61]; KneadData, 501.38 min [287.41, 1,177.06]), with Kraken2 demonstrating significantly shorter execution times compared to other tools (Fig. 2E, Supplementary Table S3-2, S3-3). The diversity of microbiomes exhibited no impact on processing time across all 6 software (Supplementary Table S3-2). However, the size of the metagenomic data significantly influenced execution time (Fig. 2D, E), which emphasized the importance of utilizing fast software for efficient processing, especially when dealing with large metagenomic datasets. Simulated metagenomic data from humans took more time to process than data from rice across all tools, indicating that host genome complexity leads to increased processing time (Supplementary Fig. S4, Supplementary Table S3). Noticeably, a high proportion of host genome contamination significantly reduced the speed of alignment-based tools like BWA, Bowtie2, KneadData, and KrakenUniq (Supplementary Fig. S4, Supplementary Table S3-2). For instance, processing a large 60-Gbps metagenomic dataset containing 90% human genome contamination resulted in a significant increase in processing time (1.35-fold in KrakenUniq, 2.59-fold in BWA, 5.36-fold in KneadData, and 6.76-fold in Bowtie2) compared to the same dataset with only 10% contamination. This suggested that these tools may be less suitable for datasets with substantial host contamination. Nevertheless, this significant slowdown was not observed in KMCP and Kraken2.

### Performance in host decontamination accuracy of 6 software

**Figure 3:**
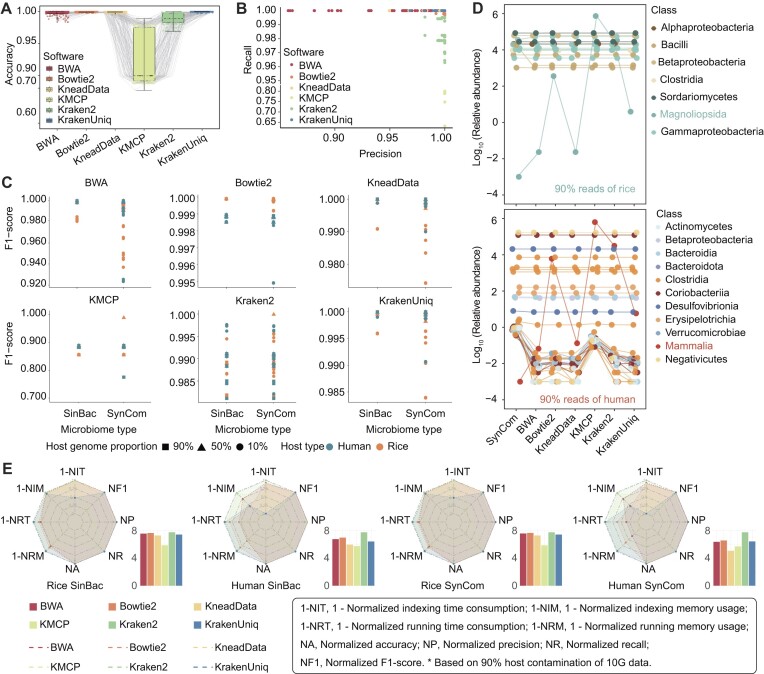
Assessing the accuracy of host contamination removal across various software. The alignment-based software displayed a higher rate of false positives, consequently diminishing the accuracy of microbiome information, while *k*-mer software exhibited an elevated occurrence of false negatives, thereby contributing to contamination of the data with host genome sequences. (A) Accuracy among 6 software, showing BWA, Bowtie2, KneadData, and KrakenUniq perform well. Accuracy = (True positive + True negative)/(True positive + True negative + False positive + False negative). (B) Precision–recall of software. The alignment-based software (BWA, Bowtie2, and KneadData) exhibited higher false positives (some microbiota reads misaligned as the host genome for removal), resulting in reduced microbiome information. However, the *k*-mer software (KMCP, Kraken2, and KrakenUniq) showed increased false negatives (some host reads not be found), leading to the host genome contamination. Precision = True positive/(True positive + False positive), Recall = True positive/(True positive + False negative). (C) High host contamination rate and microbiome complex rate reduce F1-score in 6 software. F1-score = 2 * Precision * Recall/(Precision + Recall). (D) Composition of the metagenomic dataset with a synthetic community after host contamination removal using 6 software based on 90% host contamination. BWA and KneadData retained lower host contamination in alignment-based software, and KrakenUniq and Kraken2 retained lower host contamination in *k*-mer–based software. (E) Comparative analysis of computational efficiency and host contamination removal performance across simulated 60-Gbps datasets with 90% host contamination. The bar plot presents summary values for all indicators, highlighting Kraken2’s excellence in comprehensive comparisons. The abbreviations for the indicators are as follows: 1-NIT, 1–normalized indexing time consumption; 1-NIM, 1–normalized indexing memory usage; 1-NRT, 1–normalized running time consumption; 1-NRM, 1–normalized running memory usage; NA, normalized accuracy; NP, normalized precision; NR, normalized recall; NF1, normalized F1-score; SinBac, single bacterium; SynCom, synthetic community.

Four metrics (accuracy, recall, precision, and F1-score) were calculated to evaluate the performance of host decontamination accuracy in 6 software based on the 1,080 simulated data generated according to the rule in Fig. 2A. We observed significant differences among the 6 software (*P* < 0.05) in the accuracy, recall, precision, and F1-score (Supplementary Tables S3-4, S3-5, S3-6, S3-7, S3-8). In terms of accuracy, the alignment-based software (BWA, 0.9989 [0.9966, 0.9998]; Bowtie2, 0.9997 [0.9988, 0.9998]; and KneadData, 0.9997 [0.9989, 0.9998]) outperformed the *k*-mer software (KMCP, 0.8947 [0.8133, 0.9748]; Kraken2, 0.9891 [0.9832, 0.9974]), with the exception of KrakenUniq (0.9998 [0.9994, 0.9999]), which consistently exhibited a high and stable performance (Fig. 3A). However, the alignment-based software exhibited lower precision performance (BWA, 0.9980 [0.9853, 0.9996]; Bowtie2, 0.9999 [0.9999, 0.9999]; and KneadData, 0.9981 [0.9971, 0.9998]), potentially leading to an increased number of false positives associated with the host genome. This implied that some microbiome reads may be erroneously mapped as part of the host genome and subsequently removed as contamination. Conversely, *k*-mer software (KMCP, 0.7686 [0.7477, 0.7925]; Kraken2, 0.9787 [0.9787, 0.9823]; and KrakenUniq, 0.9999 [0.9999, 1]) showed lower recall performance, leading to an increased number of false negatives associated with the host genome. This suggested that some host reads may be erroneously unmapped, thereby retaining some host contamination in the downstream analyses (Fig. 3B). For F1-score (2 * Precision * Recall/(Precision + Recall)), the type of microbiome, host type, and the proportion of host genome all influenced the performance of these tools. Notably, BWA, KneadData, and KrakenUniq performed significantly better on human datasets compared to rice datasets. Conversely, Bowtie2 showed great performance with the rice dataset (Fig. 3C).

We compared the composition of the metagenomic dataset with a synthetic community (SynCom) after host decontamination using the abovementioned 6 software (Fig. 2A). The classes Magnoliopsida and Mammalia displayed low values (i.e., the log_10_-transformed relative abundance) in BWA, KneadData, and KrakenUniq (Fig. 3D). Among these, BWA and KneadData, both alignment-based tools, demonstrated superior performance in removing host contamination compared to KrakenUniq, which, as a *k*-mer–based tool, tends to be less effective. All tools, except for KMCP, identified a few groups, belonging to Actinomycetes, Clostridia, and Negativicutes, as host contamination and removed them from the raw data (Fig. 3D). For the integrated comparison of resource consumption and the performance of host decontamination across software, we normalized all data using min–max normalization. This enabled a comparative analysis of computational efficiency and host contamination removal effectiveness across simulated datasets (Fig. 3E, Supplementary Fig. S5). Based on the summarized normalized data, Kraken2 showed significant excellence (*P* < 0.05) under high levels (90%) of host contamination, in both humans (7.8093 [7.7236, 7.8215]) and rice (7.8268 [7.7109, 7.8330]) datasets through comprehensive comparisons. Additionally, when comparing the host removal performance among alignment-based software, focusing on normalized accuracy (NA), normalized precision (NP), normalized recall (NR), and normalized F1-score (NF1) under the high levels (90%) of host contamination, KneadData demonstrated significant superiority (*P* < 0.05) in humans (3.9996 [3.9996, 3.9997]).

### The absence of accurate host reference genome affected the decontamination performance

**Figure 4:**
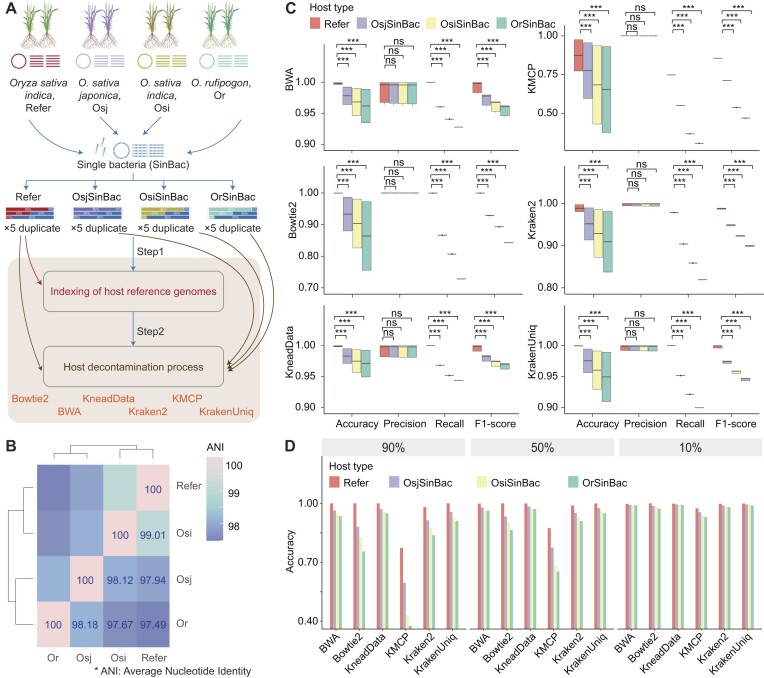
Impact of lacking a host reference genome on the performance of host decontamination tools. (A) Impact of the absence of the host reference genome on host decontamination tools. Simulated datasets were derived from 3 *Oryza* species (*Oryza sativa japonica*, Osj; *Oryza sativa indica*, Osi, *Oryza rufipogon*, Or) as hosts and a single bacterium. Each dataset contained varying levels of host DNA contamination (10%, 50%, and 90%) and was 10 Gbps in size, with 5 replicates per condition. The reference genome of *O. sativa indica* (refer) was used to create the indexing database for various host removal tools. All simulated data were aligned to this reference database to evaluate the performance of these tools in the absence of a specific host reference genome. (B) Average nucleotide identity (ANI) analysis using FastANI. ANI values for *O. sativa japonica* (Osj), *O. sativa indica* (Osi), *O. rufipogon* (Or), and reference genome (*O. sativa indica*, refer) are shown. Osi (99.01%) showed the highest similarity to the reference genome, followed by Osj (97.94%) and Or (97.49%). (C) Accuracy, precision, recall, and F1-score of 6 tools on the simulated metagenomic data from the *Oryza* genus. All tools demonstrated significantly lower accuracy, recall, and F1-score for OsjSinBac, OsiSinBac, and OrSinBac compared to the reference data when aligned to the indexing database, which was created using the reference genome. (D) Accuracy index of different software across various host genome proportions (90%, 50%, 10%). High host contamination of metagenomic data in the absence of the host reference genome notably affected the performance of existing host removal tools.

Next, we assessed the impact of lacking a host reference genome on the effectiveness of existing host decontamination software. Three *Oryza* species—*Oryza sativa japonica* (GWHBFOO00000000, Osj), *Oryza sativa indica* (GWHBFPT00000000, Osi), and *Oryza rufipogon* (GWHBFHN00000000, Or)—were selected as the resource of host metagenomic reads to generate simulated datasets. *O. sativa indica* (GWHBFPX00000000, Refer) was chosen as the reference genome (Fig. 4A). The average nucleotide identity (ANI) between the 3 species (Osj, Osi, Or) and the reference genome showed that Osi had the highest similarity (99.01%) to the reference genome, followed by Osj at 97.94% and Or at 97.49% (Fig. 4B). The simulated metagenomic reads derived from a single bacterium (SinBac) was generated as before, resulting in 3 datasets (OsjSinBac, OsiSinBac, OrSinBac) that contained varying levels of host DNA contamination (10%, 50%, and 90%) and each 10 Gbps in size. Subsequently, the indexing databases for the 6 tools were built using the reference genome, and we compared the performance of host decontamination tools (BWA, Bowtie2, KneadData, KMCP, Kraken2, and KrakenUniq) under these conditions (Fig. 4A).

In terms of time and memory consumption, KneadData and KrakenUniq used more memory (Supplementary Fig. S6A), while alignment software took more time than *k*-mer software (Supplementary Fig. S6B), as previously described. When comparing with reference metagenomic data, processing OsjSinBac, OsiSinBac, and OrSinBac data resulted in all software requiring significantly more time (Supplementary Fig. S6B). Notably, the absence of a closely aligned and accurate host reference genome negatively impacted the decontamination performance of all tools (Fig. 4C). Specifically, accuracy, recall, and F1-score were significantly lower for datasets representing OsjSinBac, OsiSinBac, and OrSinBac compared to reference data aligned to the indexing database created with the reference genome. Precision, however, did not show significant differences, indicating that decontaminated datasets still contained some residual host reads.

Then we compared the comprehensive performance for all software during the absence of the accurate host reference genome based on the summarized normalized data of resource consumption during running and host decontamination metrics (accuracy, precision, recall, and F1-score). All software performed better with reference metagenomic data (5.41 [5.15, 5.91]) than with OsjSinBac (4.93 [4.32, 5.50]), OsiSinBac (4.54 [3.91, 5.35]), and OrSinBac (4.64 [4.08, 5.19]) datasets, whereas less difference across these datasets was observed with Kraken2 and KrakenUniq (Supplementary Fig. S6C). Moreover, while different tools performed better on high host contamination samples with a reference genome, this advantage was not evident in the absence of a reference genome. In the presence of 90% host contamination, all tools showed a significant reduction in accuracy (Fig. 4D, Supplementary Table S4-1), emphasizing the importance of a host reference genome for high contamination metagenomic data.

## Discussion

Host contamination in metagenomic data analysis remains a critical challenge, particularly for datasets derived from complex environments or ultra-high-depth sequencing. In our study, we observed 57.94% of publications eliminated host contamination before analyzing their data, while the remaining studies opted to analyze their data directly, in case they potentially sacrificed valuable microbiome information in the process. Some software was developed to decontaminate the host genome [10, 20–24], but limited research provided the distinctions among them. For accelerating the reproducibility, comparability, and standardization of metagenomic data, it is important to understand the impact of these different tools, broadly referred to downstream analyses [12]. The significant influence of function annotation, species annotation, reads assembly, and binning on the consumption of computational resources makes it necessary to remove host contamination, particularly in ultra-high-depth sequencing data.

We generated a simulated dataset with 0.1 million reads of 30 microbial species and 27 million reads of *H. sapiens*. Among the microbiome species, 6 belonged to Actinomycetota, 15 to Bacillota, 6 to Bacteroidota, and 1 each to Pseudomonadota, Thermodesulfobacteriota, and Verrucomicrobiota. However, the proportion of the relative abundance was not equal, and 2 phyla not included in the simulation (Streptophyta and Campylobacterota) were annotated. This discrepancy may be attributed to the limitations of the Kraken2 annotation process, despite our use of the most comprehensive database (PlusPFP), as well as the heterogeneity of our simulated data. In addition, although each of simulated data comprises 30 microbial groups, we only obtained 14 MAGs. Notably, *Bifidobacterium breve* was recovered exclusively from the raw samples when using strict bin refinement parameters (-c 50, -x 10). By adjusting the MetaWRAP refinement parameters to -c 0 -x 100, additional MAGs of *B. breve* were recovered in the Microbiome and Remove datasets, with improved completeness and contamination metrics. This finding highlights the trade-offs between stringent parameters that prioritize MAG quality and more lenient settings that enhance the recovery of microbial diversity. It underscores the need for further refinement of metagenomic analysis software to achieve a better balance between these objectives, especially in the complex datasets. Nonetheless, the importance of the host decontamination was still being demonstrated in terms of gene functional annotation. Meanwhile, the significant difference in the alpha diversity index between microbiome (negative control) data and raw or removed data highlights the importance of establishing a “gold standard” for host removal.

It is noteworthy that we observed significantly higher accuracy, precision, and F1-score in datasets with high (90%) compared to low (10%) levels of host contamination in KneadData. Similar trends were also observed in other alignment software. This phenomenon may be attributed to 2 factors. First, our simulated data, derived from the host genome used as the indexing reference database, resulted in a higher proportion of host reads for high host contamination data (90%) aligning to the host reference in alignment software (BWA, Bowtie2, KneadData). Second, reads from the microbiome may be challenging to distinguish from reference genomes, leading to a higher number of microbiome reads being discarded in datasets with lower host contamination (10%).

We agree that comparing the impact of parameter settings across various software is an important consideration for optimizing host contamination removal. Different tools have different default parameters that can significantly influence both the decontamination accuracy and computational efficiency. For example, several studies have compared the performance of tools like Bowtie2 [28] and Kraken2 [19] under various parameter settings, highlighting the trade-offs between computational resources and host removal effectiveness. Future work focusing on systematically comparing parameter settings across a range of tools could provide valuable insights into how to balance these factors, particularly when dealing with large datasets or high levels of host contamination. This comparative approach would help standardize workflows, improve reproducibility, and enhance the efficiency of host decontamination processes in metagenomic studies.

Additionally, the challenge of predicting the host genome when it is unavailable, potentially down to the genus, family, or order level, needs consideration. Interestingly, despite Osi having a closer ANI to the reference genome compared to Osj, the decontamination performance for OsiSinBac was not as effective as for OsjSinBac. The high host contamination also showed a significant reduction of accuracy in all software, emphasizing the importance of a host reference genome for high contamination metagenomic data [29]. The absence of an accurate host reference genome can lead to residual host sequences, which in turn reduces the precision of subsequent functional annotations. One possible solution might be the combination of alignment-based and *k*-mer methods for more accurate host contamination removal. However, a unique challenge remains in distinguishing microbial sequences resulting from horizontal gene transfer (HGT) rather than host contamination. HGT between the microbiome and host genomes, often involving mobile genetic elements (MGEs), plays a crucial role in microbial adaptation to diverse environments. Metagenomic sequencing, particularly with short-read technologies, faces significant difficulties in accurately identifying these horizontally transferred gene regions [30]. In the future, advances in artificial intelligence algorithms [31] and long-read sequencing may help overcome not only host contamination but also the problem of horizontal transfer of bacteria.

## Conclusion

In conclusion, host decontamination not only speeds up downstream analysis but also enhances the accuracy of gene function annotation, particularly in ultra-high-depth sequencing data. Each of these tools (BWA, Bowtie2, KneadData, Krakne2, KMCP, and KrakenUniq) also offers unique strengths that can be harnessed based on the specific requirements of a research study. Briefly, Bowtie2, and KneadData provide more accurate removal capabilities, albeit with increased computational demands. Kraken2 and KrakenUniq offer a fast and user-friendly solution, while KMCP can retain more low-abundance taxa. When reference genomes are lacking, BWA and KneadData are less impacted among alignment software, and Kraken2 and KrakenUniq are less affected among *k*-mer software.

Understanding the trade-offs between speed, accuracy, and computational resources is crucial for selecting the most suitable tool for host DNA removal in metagenomic analyses. As research increasingly focuses on understanding the impact of host contamination on microbiome annotation, particularly for low-abundance taxa [29], this study provides a comprehensive evaluation that lays the groundwork for refining tools and methodologies. Ultimately, these advancements will empower researchers to derive meaningful biological insights from complex metagenomic datasets.

## Methods

### Literature searches and data collection

A literature search was conducted in the Web of Science Core Collection Database on 18 April 2024, using the search terms “metagenome” and “microbiome” (Supplementary Table S1-1). Only research articles were utilized to gather information on software usage (Supplementary Fig. S1A). We excluded publications solely focused on amplicon sequencing data, long-read metagenomes, environmental samples, or food samples. Subsequently, we compiled the percentage of publications mentioning host contamination removal and the number of publications for each software.

### Simulated dataset description for the downstream analysis

Three groups of data (S1, S2, S3) with 90% host contamination were simulated, including genomes from 30 microbiota species and *H. sapiens* (GRCh38). The 30 microbial species were randomly selected based on a previously published human-associated microbial community [32]. Here we used CAMISIM to generate metagenomic data for 30 microbial genomes, simulating paired-end reads (PE150) with 3 replicates for each microbial species (number_of_samples = 3). Each dataset comprised 3 million microbial reads, with 0.1 million reads per microbial species, combined with 27 million reads from *H. sapiens*. The reads were labeled accordingly before mixing (Supplementary Table S1-2). Among the microbiome species, 6 belonged to Actinomycetota, 15 to Bacillota, 6 to Bacteroidota, and 1 each to Pseudomonadota, Thermodesulfobacteriota, and Verrucomicrobiota. To remove host contamination from the raw data (Raw), we used KneadData, a bioinformatics tool specifically designed for this purpose. The output from KneadData processing constituted the host removal data (Remove). The 30 microbial groups served as a negative control (Microbiome) to evaluate the accuracy and effectiveness of the host contamination removal process.

### Metagenomic analysis

In order to compare the difference of analysis in direct data (Raw data) and the host contamination removal data (Remove data), we selected 10-Gbps synthetic community datasets of humans with varying host contamination levels (Fig. 1A). The resource consumption was tested as follows, and metagenomic analysis directly refers to the steps of EasyMetagenome 1.10 pipeline [17, 33]. Briefly, taxonomic profiling was performed using Kraken2 [34], with the PlusPFP database, and relative abundances were obtained using Bracken. Functional profiling was preformed via HUMAnN3 [35] using Uniref90 gene families. After assembling the metagenomic data using Megahit 1.0 (RRID:SCR_018551) [36], metagenomic binning and bin refinement were conducted using MetaWRAP [37]. The MetaWRAP refinement aimed to enhance the quality of our MAG binning, utilizing the parameters -c 50 -x 10, which retained only bins with completeness greater than 50% and contamination less than 10%. Redundancies in the MAGs were removed with dRep v2.6.2. MAGs were annotated using GTDBtk v2.3.2 [38], and their quality was evaluated using CheckM2 v1.0.1 [39]. Gene prediction was performed using Prodigal v2.6.3 (RRID:SCR_011936) [40], clustering of genes with CD-HIT v4.8.1 (RRID:SCR_007105) [41], quantification of genes with salmon v1.8.0 (RRID:SCR_017036), and gene annotation with emapper v2.1.6. Then, alpha diversity and beta diversity analyses were analyzed using R 4.2.3 (RRID:SCR_001905) as described in EasyAmplicon [42, 43]. The completeness and contamination rates of MAGs were normal measurement data, and thus they were presented as median (P25, P75). We also annotated the GO terms with eggnog-mapper and calculated their correlation with microbiome data, which only retained microbiome from raw data, using Spearman [44].

### Simulated dataset description for the comparison of six tools using human and rice data

We selected humans and rice, both of significant economic and medical importance, and with well-characterized genomes, as the focus of our study. Six tools were selected for analysis, 3 of which are alignment-based software (BWA [RRID:SCR_010910], Bowtie2 [RRID:SCR_016368], and KneadData), while the others are *k*-mer based (KMCP, Kraken2, KrakenUniq). Simulated datasets were generated using CAMISIM, and analyses were conducted using default or author-recommended parameters. To ensure comparability and reliability, each dataset comprised 5 replicates. These datasets covered various data sizes (10 Gbps, 30 Gbps, 60 Gbps), different levels of host DNA contamination (90%, 50%, 10%), and diverse microbial complexities (SinBac or SynCom) from both human and rice samples (Fig. 2A). Each dataset had 5 replicates per condition. For the rice SynCom, we selected 14 commonly reported species, chosen randomly from known rice-associated microbes. The human SynCom was constructed with 35 species (Supplementary Table S2-1), based on a previously published human-associated microbial community [32]. For the SinBac simulations, we utilized default parameters in CAMISIM to generate paired-end reads (PE150) for both the host and a single bacterial genome, conducting 5 replicates for each simulation (number_of_samples = 5). We then mixed reads from the host and microbial genomes in varying proportions of host DNA contamination. In the case of the SynCom simulations, we employed the same methods as in the SinBac for generating the host’s metagenomic data. However, for the microbial metagenomic data, we used the differential mode in CAIMISM. The resulting data were then mixed according to different levels of host DNA contamination. Taxonomy information and their genome IDs are provided in Supplementary Table S2-1.

To ensure comprehensive evaluation, we generated 1,080 simulated datasets using CAMISIM. These datasets encompass 3 distinct sizes (10 Gbps, 30 Gbps, and 60 Gbps) and represent both simple (SinBac) and complex (SynCom) microbiomes. The simulations were conducted separately for human (*H. sapiens*, GRCh38) and rice (*O. sativa indica*, GWHBFPX00000000) hosts, each with 3 levels of host contamination (10%, 50%, and 90%), enabling a nuanced exploration of host genome contamination removal across various conditions (Fig. 1A). For the species containing multiple chromosomes, we just downloaded all of them and stimulated the information, and all of reference and fasta information have been attached in Supplementary Table S2-1. Here, for convenience, we have assigned abbreviations to various datasets. For example, SinBac10-1 denoted a 10-Gbps dataset with 90% of reads originating from the host genome and 10% from a single bacteria genome. Similarly, SynCom 30-2 represented a 30-Gbps dataset with an even split of 50% reads from the host genome and 50% from the synthetic community genome. Based on these simulated data, we assessed the impact of metagenomic sequencing on the microbiome by removing host contamination.

### Simulated dataset description for the comparison of 6 tools within a genus level

To assess the performance of host decontamination tools at the genus level, we utilized 3 rice species and simple microbiomes separately: *O. sativa japonica* (GWHBFOO00000000, Osj), *O. sativa indica* (GWHBFPT00000000, Osi), and *O. rufipogon* (GWHBFHN00000000, Or). For each species, we generated datasets by combining the host genome with a simple bacterial genome, resulting in 3 separate datasets: Or with simple bacteria (OrSinBac), Osj with simple bacteria (OsjSinBac), and Osi with simple bacteria (OsiSinBac). Each dataset was simulated using CAMISIM with different levels of host DNA contamination (10%, 50%, and 90%) for 10-Gbps datasets, with 5 replicates per condition.

*O. sativa indica* served as the reference genome (GWHBFPX00000000, Refer), and indices were created for 6 tools (BWA, Bowtie2, KneadData, KMCP, Kraken2, KrakenUniq), as described above. Detailed fasta information is provided in Supplementary Table S2-1. These simulated datasets were analyzed to compare the performance of the 6 tools in removing host contamination within the genus. The comparison involved evaluating computational resources and the effectiveness of host decontamination. Genome similarity was calculated using fastANI v1.34 (RRID:SCR_021091) [45], and the impact of metagenomic sequencing on the microbiome has been assessed by testing the following performance metrics. Based on these datasets, we aimed to determine how each tool performs in removing host contamination when genome data are limited within the same genus.

### Performance metrics tests

All software utilized 8 threads for building the database and running the processes across simulated datasets [46]. We assessed a comparative analysis of resource consumption, focusing on maximum RAM usage and processing time. Additionally, we evaluated their performance metrics including true positive (TP), true negative (TN), false positive (FP), and false negative (FN). The precision, recall, and F1-score were also calculated, with the following formulas: Accuracy = (TP + TN)/(TP + FN + FP + TN), Precision = (TP/(TP + FP)), Recall = (TP/(TP + FN)), F1-score = 2 * Precision * Recall/(Precision + Recall) [47]. Then, all bioinformatics analyses were all analyzed within R 4.2.3. We conducted normality and homogeneity tests on all data. Measurement data were expressed as mean ± SE for normally distributed data and as median (P25, P75) for nonnormally distributed data. For normally distributed data with homogeneous variances in 2-group comparisons, we used paired *t*-tests. Nonnormally distributed or nonhomogeneous data were analyzed using nonparametric Wilcoxon tests. In multiple group comparisons, normally distributed data with homogeneous variances were analyzed with analysis of variance, while nonnormally distributed or nonhomogeneous data were analyzed with nonparametric Kruskal–Wallis tests [48]. Bonferroni post hoc tests were performed on the data within each group to analyze the differences between different datasets [49]. For the average species composition plot, when dealing with data containing zeros, we adopted the straightforward method of adding a positive constant (0.001) to all leaf trait values [50] and then taking the logarithm of the resulting average relative abundance. For the integrated comparison of resource consumption and the performance of host purge in each software, we normalized all data by using min–max normalization [51], which is a technique that performs a linear transformation of the original data. Data visualization was done using the ggplot2 package, and *P* ≤ 0.05 was regarded as statistically significant [52].

## Availability of Source Code and Requirements

**1. Workflow**

Project name: HostPurge-DownstreamAnalysis

Project homepage: https://github.com/YunyunGao374/HostPurge/blob/main/0HostDecontaminationImpactiononDownstreamAnalysis.sh [44]

Operating system(s): Linux

Programming language: Bash, R

Other Requirements: Environment Modules, Conda

**2. GitHub for benchmarking dataset**

Project name: HostPurge-SoftwareComparison

Project homepage: https://github.com/YunyunGao374/HostPurge/blob/main/1HostDecontaminationSoftwareComparison.sh [46]

Operating system(s): Linux

Programming language: Bash, R

Other Requirements: Environment Modules, Conda

**3. GitHub for figures**

Project name: HostPurge-paper-figures

Project homepage: https://github.com/YunyunGao374/HostPurge [52]

Operating system(s): Windows, MacOS, Linux

Programming language: R

Other requirements: N/A

**License:** GNU General Public License v3.0

## Supplementary Material

giaf004_Supplemental_Files

giaf004_GIGA-D-24-00318_Original_Submission

giaf004_GIGA-D-24-00318_Revision_1

giaf004_GIGA-D-24-00318_Revision_2

giaf004_Response_to_Reviewer_Comments_Original_Submission

giaf004_Response_to_Reviewer_Comments_Revision_1

giaf004_Reviewer_1_Report_Original_Submission Car Reen Kok -- 9/19/2024

giaf004_Reviewer_1_Report_Revision_1 Car Reen Kok -- 11/27/2024

giaf004_Reviewer_2_Report_Original_Submission Calum Walsh -- 9/25/2024

giaf004_Reviewer_2_Report_Revision_1 Calum Walsh -- 11/25/2024

## Data Availability

The raw data of simulated metagenomic sequencing reads have been deposited at the Genome Warehouse (GWH) under accession PRJCA028271 and also uploaded to the National Center for Biotechnology Information (NCBI) under accession PRJNA1148749. All pipelines, data analyses, and plotting code are available via the GitHub repository [52]. An archival copy of the code and supporting data are available via the *GigaScience* database, GigaDB [53].

## Additional Files

**Supplementary Fig. S1**. Literature search for existing studies addressing host contamination. (A) Literature search criteria. (B) The proportion of publications mentioning the removal of host contamination. (C) The existing software utilized for removing host genomes.

**Supplementary Fig. S2**. Comparison of host removal performance on the accuracy of the results in metagenomic analysis. (A) Shannon index, richness index, and principal coordinates analysis (PCoA) across microbiome removed and raw data. (B) The completeness and contamination rates of metagenome-assembled genomes (MAGs) were assessed in microbiome, removed, and raw data. (C) Correlation assessment of Gene Ontology (GO) terms between microbiome data and removed data or raw data in S2 and S3 samples.

**Supplementary Fig. S3**. Memory usage during the host-removing process of BWA, Bowtie2, KneadData, KMCP, Kraken2, and KrakenUniq in simulation rice and human metagenome. SinBac, single bacteria; SynCom, synthetic community.

**Supplementary Fig. S4**. Time consumption during the host-removing process of BWA, Bowtie2, KneadData, KMCP, Kraken2, and KrakenUniq. SinBac, single bacterium; SynCom, synthetic community.

**Supplementary Fig. S5**. Comparative analysis of composition, computational efficiency, and host contamination removal performance across 6 software. (A) Composition of the metagenomic dataset with a synthetic community after host contamination removal using 6 software. (B) Computational efficiency and host contamination removal performance. 1-NIT, 1–Normalized indexing time consumption; 1-NIM, 1–nrmalized indexing memory usage; 1-NRT, 1–normalized running time consumption; 1-NRM, 1–normalized running memory usage; NA, normalized accuracy; NP, normalized precision; NR, normalized recall; NF1, normalized F1-score. SinBac, single bacterium; SynCom, synthetic community.

**Supplementary Fig. S6**. Assessing the performance of 6 tools at the genus level. (A) Memory usage during the host-removing process for 6 tools across OsjSinBac, OsiSinBac, OrSinBac, and reference metagenomic data. (B) Time consumption during the host-removing process for 6 tools across OsjSinBac, OsiSinBac, OrSinBac, and reference metagenomic data. (C) Computational efficiency and host contamination removal performance for 6 tools across OsjSinBac, OsiSinBac, OrSinBac, and reference metagenomic data. 1-NRT, 1–normalized running time consumption; 1-NRM, 1–normalized running memory usage; NA, normalized accuracy; NP, normalized precision; NR, normalized recall; NF1, normalized F1-score; OsjSinBac, *Oryza sativa japonica* with a single bacterium; OsiSinBac, *Oryza sativa indica* with a single bacterium; OrSinBac, *Oryza rufipogon* with a single bacterium.

**Supplementary Table S1-1**. A literature review of host contamination purging methods in metagenomic studies since April 18th, 2024.

**TableS1-2**. The microbiome composition of simulated dataset for metagenomic downstream analysis.

**TableS2-1**. The data resource information from different genomes in rice (a) and human (b) samples.

**TableS2-2**. The data composition of complex microbiomes (SynCom) in rice (a) and human (b) samples.

**TableS2-3**. Difference of time consumption and memory usuage bweteen Raw data and HostRemove data.

**TableS2-4**. Comparison of the completeness and contamination of metagenome assembled genomes between Raw data and HostRemoved data.

**Supplementary Table S3-1**. Statistical analysis of variance and Wilcoxon/Kruskal-Wallis test results of memory usage on simulated metagenomic data.

**Supplementary Table S3-2**. Statistical analysis of variance and Wilcoxon/Kruskal-Wallis test results of time consumption for different software on simulated metagenomic data.

**Supplementary Table S3-3**. The comparison of peak memory usage (top right) and execution time (bottom left) among different software based on Kruskal-Wallis test (Z-score, p-value).

**Supplementary Table S3-4**. Exploring variances and Wilcoxon/Kruskal-Wallis test for accuracy value for different software on simulated metagenomic data.

**Supplementary Table S3-5**. Exploring variances and Kruskal-Wallis test results for precision value for different software on simulated metagenomic data.

**Supplementary Table S3-6**. Exploring variances and Kruskal-Wallis test results for recall value for different software on simulated metagenomic data.

**Supplementary Table S3-7**. Exploring variances and Kruskal-Wallis test results for F1-value for different software on simulated metagenomic data.

**Supplementary Table S3-8**. Computational efficiency and host contamination removal performance with min-max normalization data.

**Supplementary Table S4-1**. Exploring variances and Wilcoxon/Kruskal-Wallis test for accuracy value for different software of different simulated metagenome data in rice.

## Abbreviations

ANI: average nucleotide identity; FN: false negative; FP: false positive; GO: Gene Ontology; HGT: horizontal gene transfer; MAG: metagenome-assembled genome; MGE: mobile genetic element; NGS: next-generation sequencing; PCoA: principal coordinates analysis; SinBac: single bacterium; SynCom: synthetic community; TN: true negative; TP: true positive.
