# Supplementary figures and images for: Benchmarking short-read metagenomics tools for removing host contamination
Source: Gigascience. 2025 Feb 27;14:giaf004. doi: 10.1093/gigascience/giaf004 (PMC11878760; doi:10.1093/gigascience/giaf004)

A

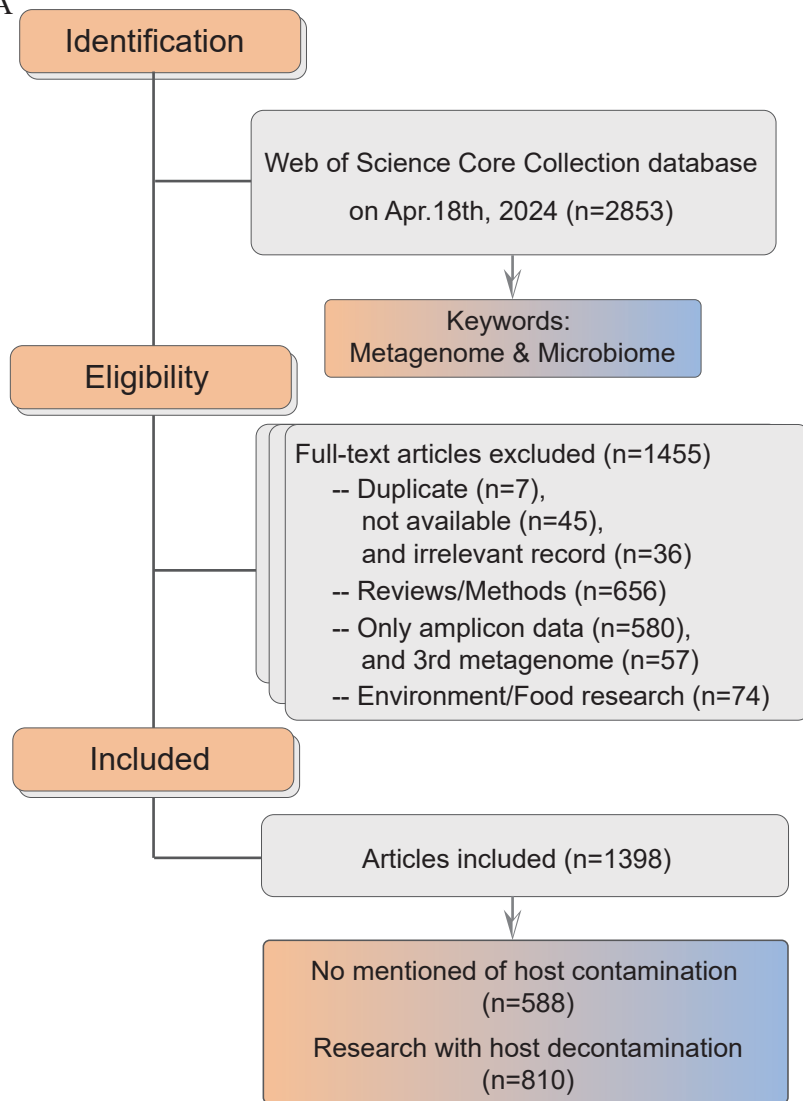

B

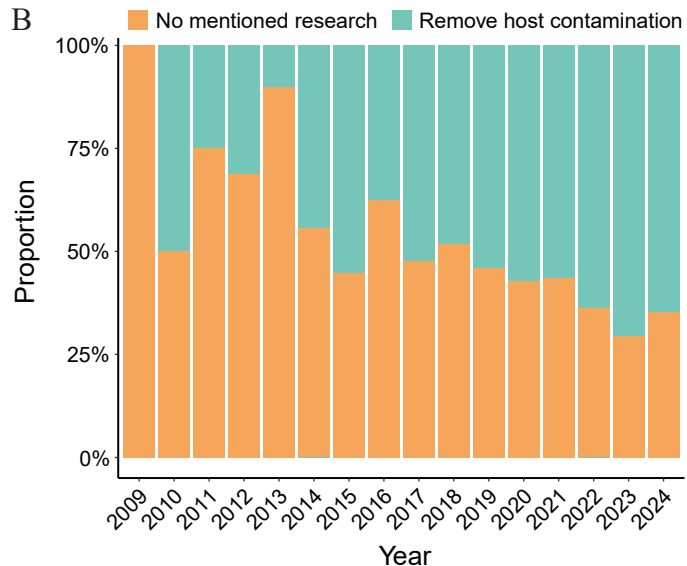

C

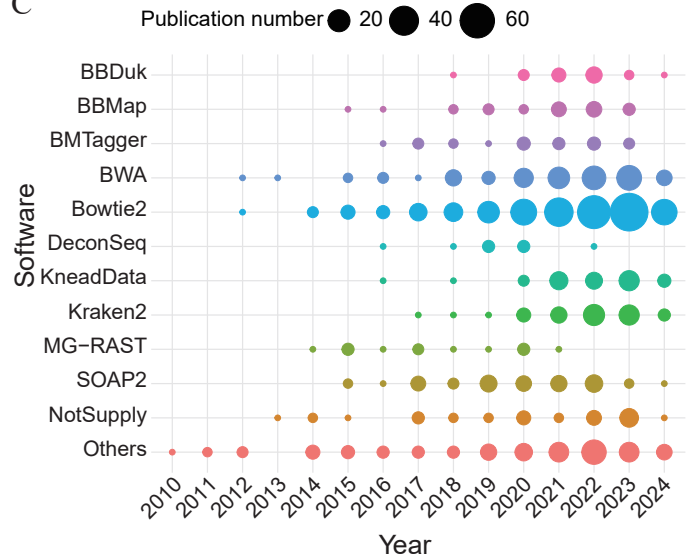

Supplement: giaf004_Supplemental_Files [file giaf004_supplemental_files.zip › FigureS1.pdf]

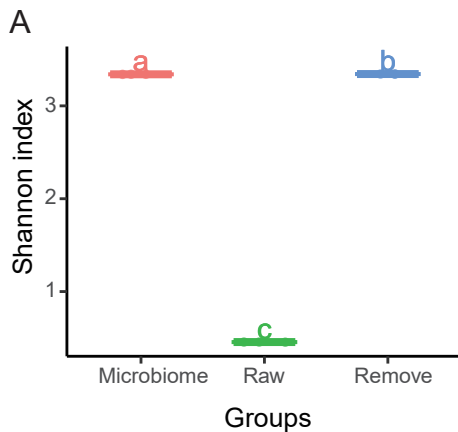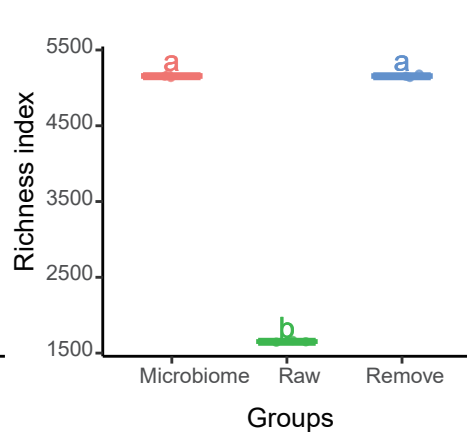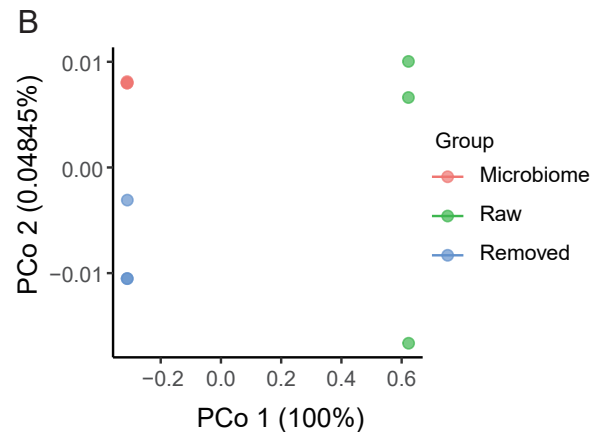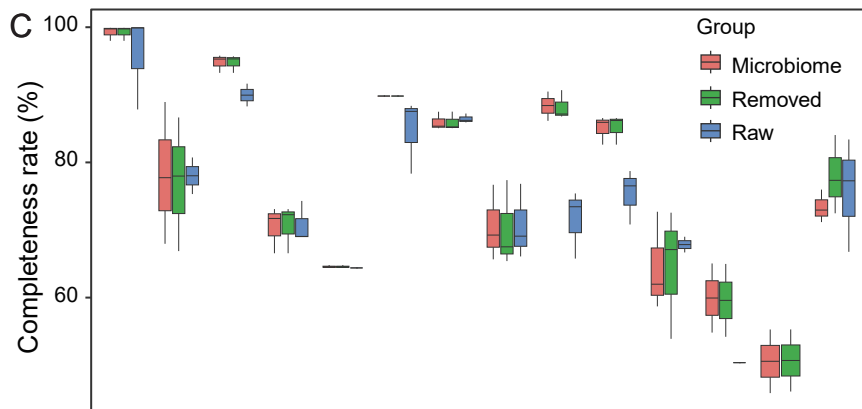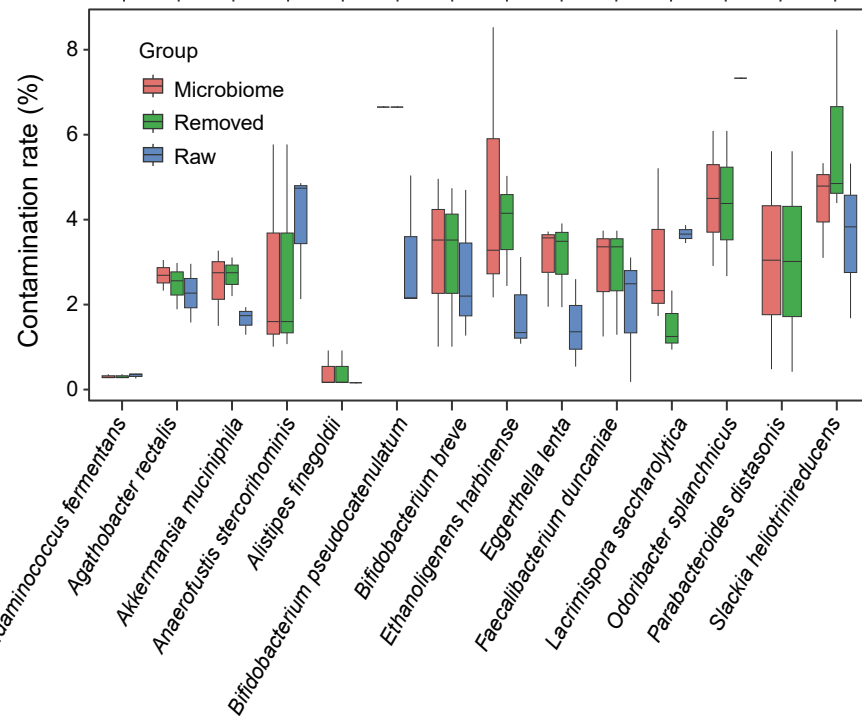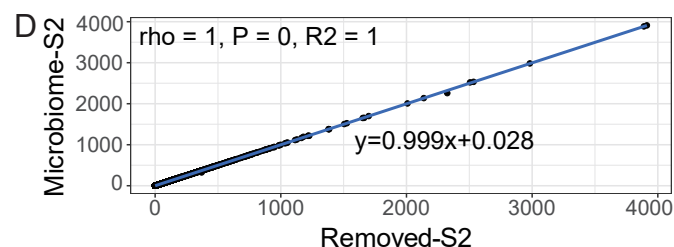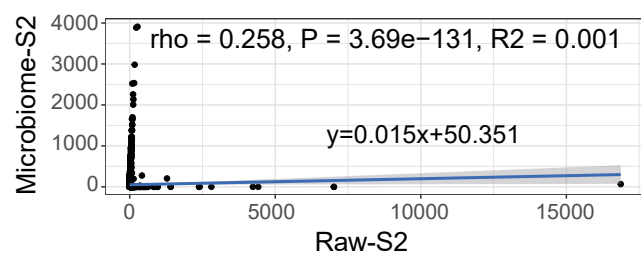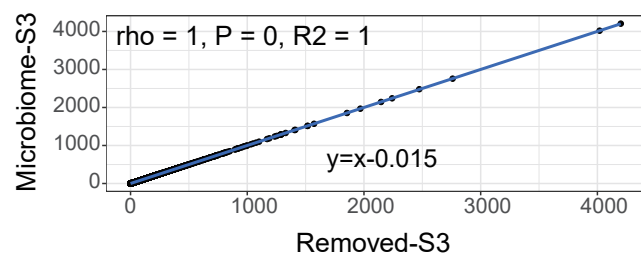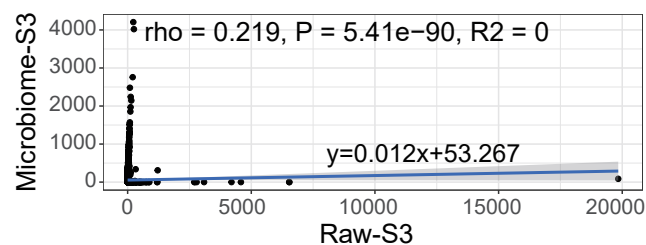

Supplement: giaf004_Supplemental_Files [file giaf004_supplemental_files.zip › FigureS2-F.pdf]

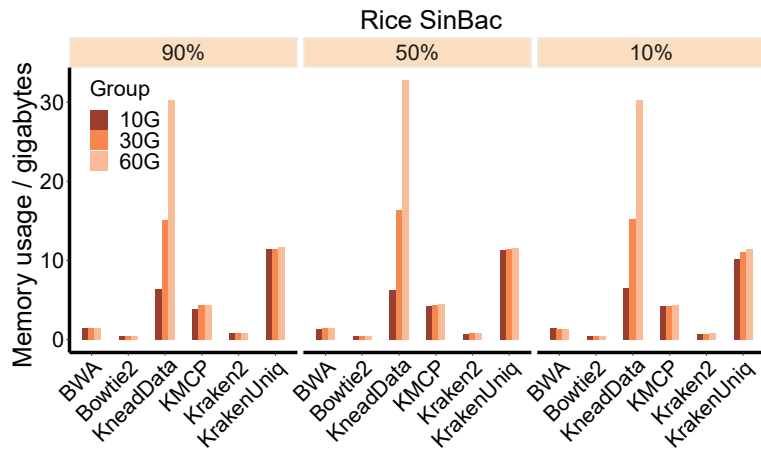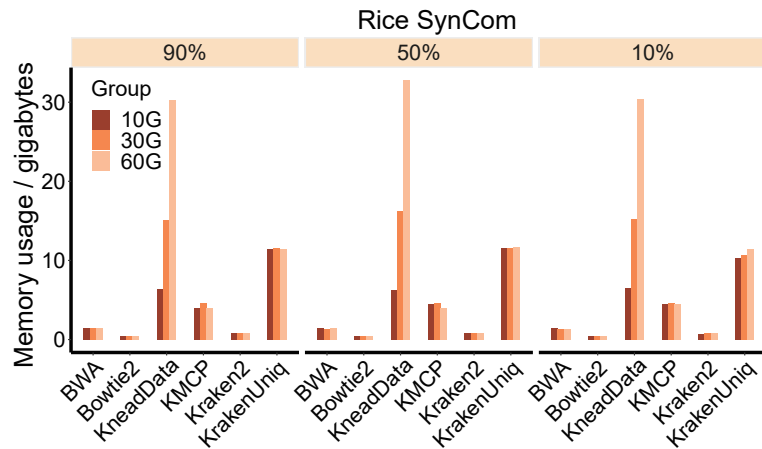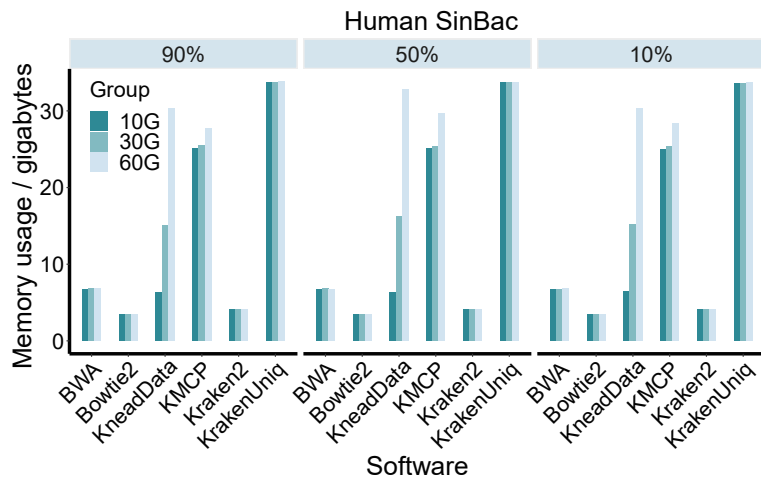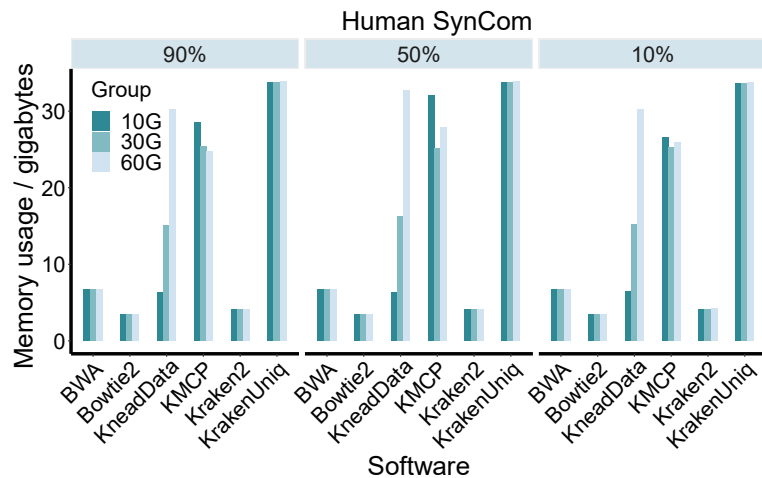

Supplement: giaf004_Supplemental_Files [file giaf004_supplemental_files.zip › FigureS3.pdf]

Rice SinBac

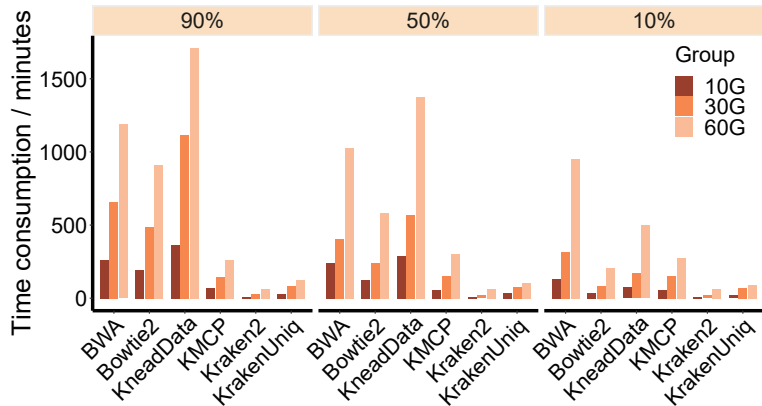

Rice SynCom

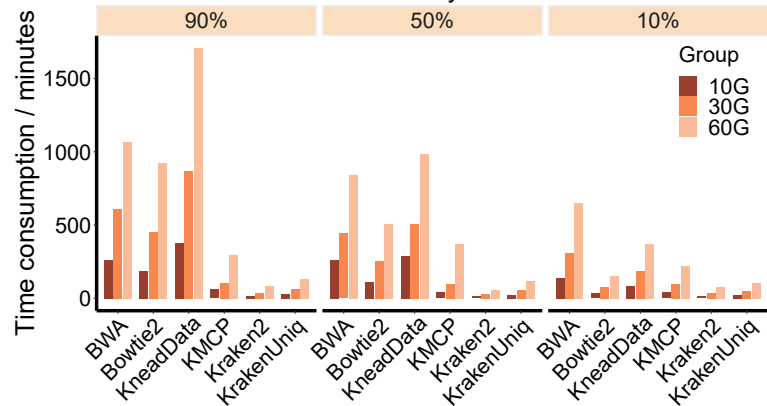

Human SinBac

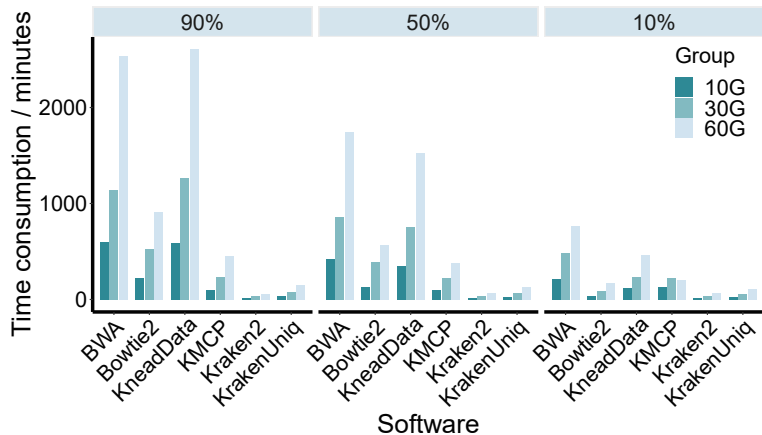

Human SynCom

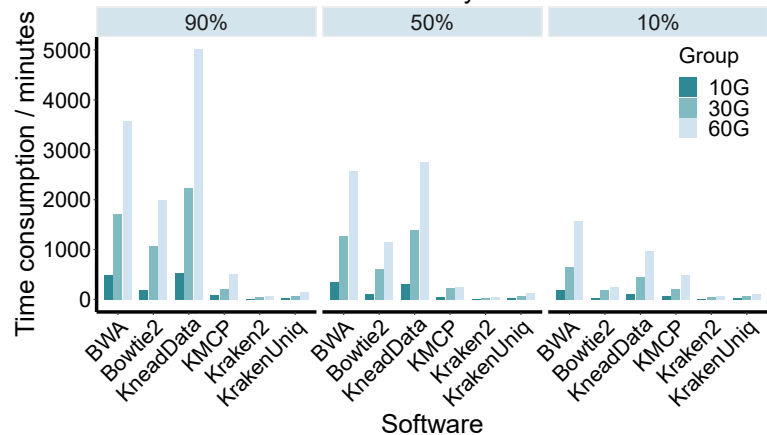

Supplement: giaf004_Supplemental_Files [file giaf004_supplemental_files.zip › FigureS4.pdf]

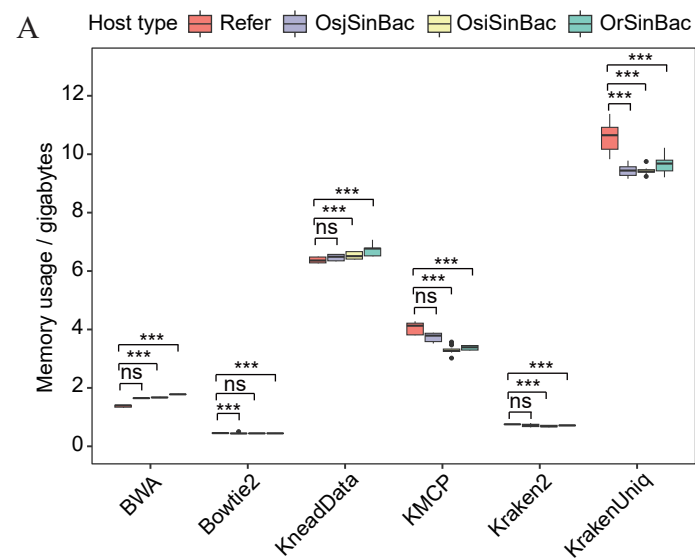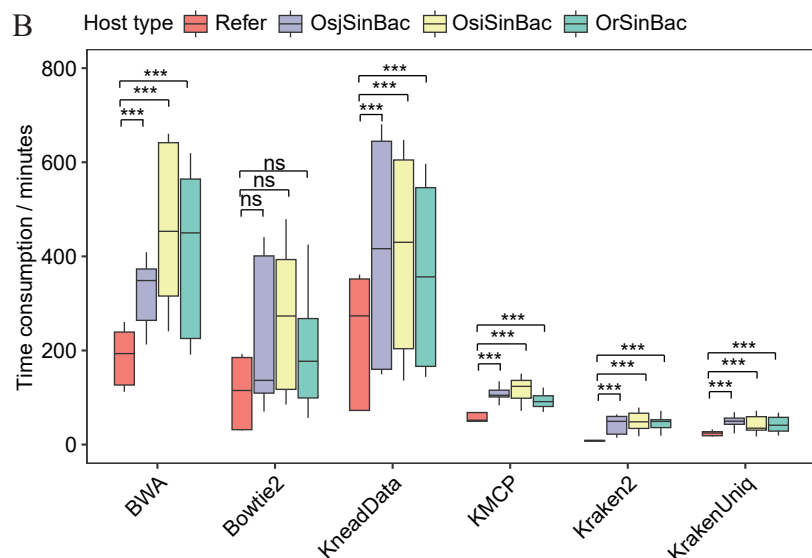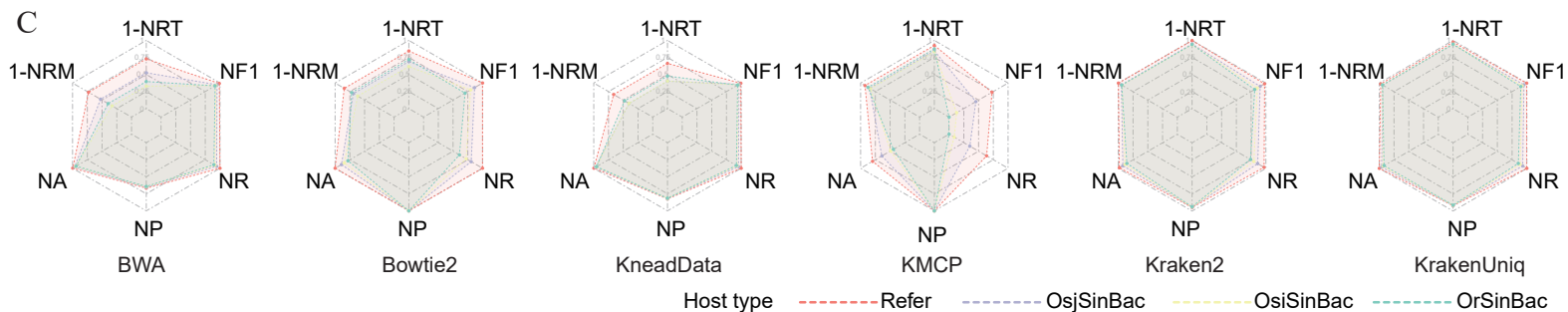

Supplement: giaf004_Supplemental_Files [file giaf004_supplemental_files.zip › FigureS6.pdf]
