# Supplementary material for: Benchmarking short-read metagenomics tools for removing host contamination
Source: Gigascience. 2025 Feb 27;14:giaf004. doi: 10.1093/gigascience/giaf004 (PMC11878760; doi:10.1093/gigascience/giaf004)

GigaScience

Benchmarking metagenomics tools for removing host contamination

--Manuscript Draft--

**Manuscript Number:**	GIGA-D-24-00318
**Full Title:**	Benchmarking metagenomics tools for removing host contamination
**Article Type:**	Research
**Funding Information:**	China Postdoctoral Science Foundation (2024M753580) — Dr. Yunyun Gao<br>National Natural Science Foundation of China (U23A20148) — Dr. Yunyun Gao
**Abstract:**	The rapid evolution of metagenomic sequencing technology offers remarkable opportunities to explore the intricate roles of microbiome in host health and disease, as well as to uncover the unknown structure and functions of microbial communities. However, the swift accumulation of metagenomic data poses substantial challenges for data analysis. Contamination from host DNA can substantially compromise result accuracy, and increase additional computational resources by including non-target sequences. In this study, we assessed the impact of computational host-DNA decontamination on downstream analyses, highlighting its importance in producing accurate results efficiently. We also evaluated the performance of conventional tools like KneadData, Bowtie2, BWA, KMCP, Kraken2, and KrakenUniq, each offering unique advantages for different applications. Furthermore, we highlighted the importance of a host reference genome, noting that its absence negatively affected the decontamination performance across all tools. Our findings underscore the need for careful selection of decontamination tools and reference genomes to enhance the accuracy of metagenomic analyses.
**Corresponding Author:**	Yongxin Liu<br>Chinese Academy of Agricultural Sciences<br>Shenzhen, Guangdong CHINA
**Corresponding Author Secondary Information:**	
**Corresponding Author's Institution:**	Chinese Academy of Agricultural Sciences
**Corresponding Author's Secondary Institution:**	
**First Author:**	Yunyun Gao, Ph.D.
**First Author Secondary Information:**	
**Order of Authors:**	Yunyun Gao, Ph.D.
	Hao Luo
	Hujie Lyu
	Haifei Yang
	Salsabeel Yousuf
	Shi Huang
	Yongxin Liu
**Order of Authors Secondary Information:**	
**Additional Information:**	
**Question**	**Response**
Are you submitting this manuscript to a special series or article collection?	No

Powered by Editorial Manager® and ProduXion Manager® from Aries Systems Corporation

**Experimental design and statistics**<br><br>Full details of the experimental design and statistical methods used should be given in the Methods section, as detailed in our Minimum Standards Reporting Checklist. Information essential to interpreting the data presented should be made available in the figure legends.<br><br>Have you included all the information requested in your manuscript?	Yes
**Resources**<br><br>A description of all resources used, including antibodies, cell lines, animals and software tools, with enough information to allow them to be uniquely identified, should be included in the Methods section. Authors are strongly encouraged to cite Research Resource Identifiers (RRIDs) for antibodies, model organisms and tools, where possible.<br><br>Have you included the information requested as detailed in our Minimum Standards Reporting Checklist?	Yes
**Availability of data and materials**<br><br>All datasets and code on which the conclusions of the paper rely must be either included in your submission or deposited in publicly available repositories (where available and ethically appropriate), referencing such data using a unique identifier in the references and in the "Availability of Data and Materials" section of your manuscript.<br><br>Have you have met the above requirement as detailed in our Minimum Standards Reporting Checklist?	Yes

Powered by Editorial Manager® and ProduXion Manager® from Aries Systems Corporation

Manuscript Click here to access/download;Manuscript;Manuscript.docx

Benchmarking metagenomics tools for removing host contamination

Yunyun Gao[1*], Hao Luo[1*], Hujie Lyu[2], Haifei Yang[1,3], Salsabeel Yousuf[1], Shi Huang[4], Yong-Xin Liu[1#]

[1]Shenzhen Branch, Guangdong Laboratory of Lingnan Modern Agriculture, Genome Analysis Laboratory of the Ministry of Agriculture and Rural Affairs, Agricultural Genomics Institute at Shenzhen, Chinese Academy of Agricultural Sciences, Shenzhen 518120, China

[2]Department of Life Sciences, Imperial College of London, London SW7 2AZ, UK

[3]College of Life Sciences, Qingdao Agricultural University, Qingdao, China

[4]Faculty of Dentistry, The University of Hong Kong, Hong Kong SAR, China

[#] Correspondence: liuyongxin@caas.cn (Yong-Xin Liu)

* These two authors contributed equally to this work.

ORCIDs

Yunyun Gao: https://orcid.org/0000-0003-3389-9385

Hao Luo: https://orcid.org/0009-0005-3391-8576

Yong-Xin Liu: https://orcid.org/0000-0003-1832-9835

Abstract

The rapid evolution of metagenomic sequencing technology offers remarkable opportunities to explore the intricate roles of microbiome in host health and disease, as well as to uncover the unknown structure and functions of microbial communities. However, the swift accumulation of metagenomic data poses substantial challenges for data analysis. Contamination from host DNA can substantially compromise result accuracy, and increase additional computational resources by including non-target sequences. In this study, we assessed the impact of computational host-DNA decontamination on downstream analyses, highlighting its importance in producing accurate results efficiently. We also evaluated the performance of conventional tools like KneadData, Bowtie2, BWA, KMCP, Kraken2, and KrakenUniq, each offering unique advantages for different applications. Furthermore, we highlighted the importance of a host reference genome, noting that its absence negatively affected the decontamination performance across all tools. Our findings underscore the need for careful selection of decontamination tools and reference genomes to enhance the accuracy of metagenomic analyses.

Keywords

Metagenome, microbiome, host removal, microbial enrichment

Background

The advancement of second-generation sequencing technology and data analysis methods has greatly facilitated microbiome research, broadening our horizons in the widespread influences of microbiome on their host [1-3]. Compared to amplicon sequencing, shotgun metagenomic sequencing offers comprehensive assessments of bacterial communities with less bias, and improved resolution in identifying profiles at the species, strain, or functional levels [4]. As sequencing technology costs rapidly decreased and sequencing depth continues to expand, the volume of metagenomic data is growing exponentially [5]. Some studies even producing over 100 giga base pairs (Gbps) per sample to characterize the dark matter of the microbiome in human gut [6]. However, a major challenge exists when analyzing the metagenome data from complex host-associated microbiomes, such as those found in saliva, throat, and vaginal swabs. These samples often, contain over 90% human-aligned reads [4, 7, 8] due to the high contamination of host DNA. This contamination undermines the characterization of microbiomes, especially for low abundant species [7], leading to biased observations of the true underlying microbial composition. Additionally, privacy concerns have become particularly significant when the host is human [9], highlighting the importance of removing host contamination.

Despite efforts to remove host contamination during the experimental stage [8, 10-13], particularly in DNA isolation, residual DNA remains polluted with numerous host DNA fragments. The overall efficacy of different experimental protocols varies, and potential biases for the preferential enrichment of specific microbial taxa remain a concern. Even after significant host DNA reduction, high biomass samples like mucosal microbiome can still exhibit up to 90% host contamination in metagenomic data [8, 10]. This persistence is due to differences in cell and genome size between animals and microbiomes. Similarly, in low biomass samples like endophytic microorganisms, around 70% of host sequencing reads may still be present despite efforts to collect and concentrate the microbiota [14]. These limitations not only compromise the accuracy of downstream analyses but also waste researcher's efforts and computing resources. This underscores the importance of devising a host contamination removing tool in the data analysis stage that is both accurate and efficient.

After searching 2,853 publications using the keywords 'metagenome' and 'microbiome' (Figure S1A, Table S1), we found that 57.94% of the studies addressed the removal of host contamination, with a discernible increasing trend from 2015 to 2024. The absence of standardized criteria for selecting host decontamination software has led to the use of 51 different tools, many relying on alignment and *k*-mer strategies. Among these, ten tools exhibited notable popularity, generally employ two main strategies: alignment-based and the *k*-mer approaches [9, 15, 16]. The alignment software, such as Bowtie2 [17], BWA [18], aligning sequencing reads to reference genomes. And Kraken2 [19], KMCP [20] are popular *k*-mer based software that identify exact matches between small substrings (*k*-mers) from the reads in the reference database. Besides, some host

contamination removal pipelines integrate these modules. For instance, DeconSeq [21] integrates a modified version of BWA, while KneadData (http://huttenhower.sph.harvard.edu/kneaddata) integrates Bowtie2. Several new tools, like Hostile [9], HoCoRT [15] have also been developed to enhance the accuracy of host decontamination process. Although some studies have explore the impact of varying amounts of host DNA on microbiomes [22, 23], the impact of depleting host DNA contamination on the bioinformatic downstream analysis and the microbial genome assembly remains unclear [12].

In this study, we will compare the efficiency of metagenomic sequencing on microbiome by removing host contamination, and thoroughly evaluate the accuracy and speed of state-of-the-arts in computational host DNA decontamination. These results will serve as a guide for researchers in rationally selecting suitable tools for processing various metagenomic datasets.

Results

High host contamination increased the processing time and skewed interpretation the microbiome results

Here we simulated three groups (S1, S2, S3) of data using CAMISIM with 90% host contamination (Table S1-2), incorporating genomes from 30 microbiota and *Homo sapiens* (GRCh38). We used KneadData, a popular host decontamination software in recent years, to remove host contamination from the raw data (Raw), resulting in host-removed data (Remove), while the 30 microbial groups served as a negative control (Microbiome). Based on these simulations, we evaluated the impact of host contamination removal on metagenomic analysis of the microbiome (Figure 1A).

During the metagenomic analysis, no significant differences in memory usage were observed among the raw data, removed data, and microbiome data during high-memory steps exceeding 100 gigabytes (GBs), such as species-level taxonomic annotation (Kraken2), de-replication (drep), and MAG annotation (GTDBtk). However, compared to the raw data, the host-read-depleted data significantly reduced the run time of downstream analyses (Figure 1B). Specifically, processing the host-depleted data took 5.98 times shorter for binning (MetaWRAP), 7.63 times shorter for function annotation (HUMAnN3), and 20.55 times shorter for assembly (MEGAHIT). And the average processing time for Remove data was 139.14 minutes (min) for MetaWRAP compared to 832.64 min for raw data, 308.92 min for HUMAnN3 compared to 2357.95 min for raw data, and 106.59 min for MEGAHIT compared to 2190.27 min for raw data. Additionally, handling the negative control data (Microbiome) required similar resources to the host-depleted data in terms of both memory usage and time consumption.

Compared to microbiome data, raw data altered the relative abundance of microbiota community, while remove data showed a similar composition to that annotated by Kraken2 (Figure 1C). Interestingly, the released taxa were similar to the microbiome data, after removing the Chordata (the phylum of *Homo sapiens*) from the raw data. There was no difference in richness index between microbiome data and remove data, whereas raw data showed a significantly lower richness index

than both (Figure S2A). And principal coordinates analysis (PCoA) was performed to visualize changes in community composition, the first axes of PCoA explained 100% of the overall variations, and samples from raw data separated from those of microbiome and the removed data along PCo1 (Figure S2B).

Despite simulating metagenomic data from 30 microbiota, with each species having 0.1 million reads, only 14 metagenome-assembled genomes (MAGs) were obtained. No significant differences were detected in completeness rate and contamination rates among the microbiome data, raw data and removed data (Figure S2C). However, the number of MAGs was much more in microbiome and removed data compared to raw data, except for *Bifidobacterium breve*, which was detected in all three groups (S1, S2, S3) in raw data, but only in S2 in microbiome and removed data (Figure 1D). Next, we compared the gene ontology (GO) terms to microbiome data. We found a higher similarity in GO terms between removed data and microbiome data than that between raw data and microbiome data (Figure 1E, Figure S2D), indicating that the host removal process results in more specific gene function annotation.

Kraken2 was fast and low-resource tool for host removal.

To ensure a comprehensive evaluation, we generated 1080 simulated datasets using CAMISIM. These datasets encompass three distinct sizes (10 Gbps, 30 Gbps, and 60 Gbps) and representing both single bacterium (SinBac) and synthetic community (SynCom) of microbiomes. The simulations were conducted separately for human (*Homo sapiens*) and rice (*Oryza sativa indica*) hosts, each with three levels of host contamination (10%, 50%, and 90%), enabling a nuanced exploration of host genome contamination removal across various conditions (Figure 2A). For convenience, we have assigned abbreviations to various datasets. For example, SinBac10-1 denoted a 10 Gbps dataset with 90% of reads originating from the host genome and 10% from a single bacterium genome. Similarly, SinBac 60-3, represented a 60Gbps dataset with an even split of 90% reads from the host genome and 10% from the synthetic community genome. Based on these simulated data, we compared the computational resources required and host decontamination performance of six existing tools: KneadData, Bowtie2, and BWA (for alignment-based software), and KMCP, Kraken2, and KrakenUniq (for *k*-mer strategy software).

Before removing host contamination, indexing of host reference genomes is crucial. In this study, we constructed reference genomes for *Homo sapiens* (GRCh38) and *Oryza sativa indica* (GWHBFPX00000000) with sizes approximately 3.1 Gbps and 373.8 Megabase pairs (Mbps), respectively. Kraken2 utilized minimal computational resources for indexing both human and rice genomes, requiring only 0.3 gigabyte (Gb) memory and taking 6.94 minutes (min) to create a custom database for the human genome (Figure 2B). In contrast, the other five tools required an average of 18.05 Gb memory and 117.98 min.

We then compared the resource consumption during the host contamination removal process across six software. In summary, Bowtie2 (1.95 Gb (0.410, 3.42)), and Kraken2 (2.47 Gb (0.710, 4.12))

demonstrated the lowest maximum memory usage across all simulated datasets for alignment and *k*-mer based software, respectively (Figure 2C, Figure S3). These values were significantly lower than that of the other four tools, with BWA requiring 3.995 Gb (1.40, 6.74), KneadData requiring 15.17 Gb (6.47, 30.27), KMCP requiring 14.45 Gb (4.27, 25.110), KrakenUniq requiring 22.410 Gb (11.13, 33.67). Regarding data size, handling 60 Gbps data consumed significantly more resources than 10 Gbps data in KneadData and Kraken2. Different host types also significantly influenced resource consumption across all software (Table S3-1), with human data requiring notably more resources than rice data ($P < 0.05$). Whereas, no significant difference between the different microbiome types for all software, except for KrakenUniq (Table S3-1).

For time usage, the *k*-mer software (KMCP, 156.10 min (90.47, 231.16), Kraken2, 29.34 min (13.42, 55.45), and KrakenUniq, 59.23 min (26.66, 98.14), required less time than the alignment-based software (BWA 582.26 min (300.15, 1065.64), Bowtie2 209.00 min (111.66, 512.61), KneadData 501.38 mins (287.41, 1177.06)), with Kraken2 demonstrating significantly shorter execution times compared to other tools (Figure 2E, Table S3-2). The diversity of microbiomes exhibited no impact on processing time across all six software (Table S3-2). But, the size of the metagenomic data significantly influenced execution time (Figure 2D, 2E), this emphasized the importance of utilizing fast software for efficient processing, especially when dealing with large metagenomic datasets. Simulated metagenomic data from human took more time to process than data from rice across all tools, indicating that host genome complexity leads to increased processing time (Figure S4, Table S3). Noticeably, a high proportion of host genome contamination significantly reduced the speed of alignment-based tools like BWA, Bowtie2, KneadData, and KrakenUniq (Figure S4, Table S3-2). For instance, processing a large 60 Gbps metagenomic dataset containing 90% human genome contamination resulted in a significant increase in processing time (1.35-fold in KrakenUniq, 2.59-fold in BWA, 5.36-fold in KneadData, and 6.76-fold in Bowtie2) compared to the same dataset with only 10% contamination. This suggested that these tools may be less suitable for datasets with substantial host contamination. Nevertheless, this significant slowdown was not observed in KMCP and Kraken2.

Performance in host decontamination accuracy of six software

Then four metrics (accuracy, recall, precision, and F1-score) were calculated to evaluate the performance of host decontamination accuracy in six software based on with the 1080 simulated data generated according to the rule in Figure 2A. We observed significant differences among the six software ($P < 0.05$) in the accuracy, recall, precision, and F1-score (Table S3-4, S3-5, S3-6, S3-7). In terms of accuracy, the alignment-based software (BWA, 0.9989 (0.9966, 0.9998), Bowtie2, 0.9997 (0.9988, 0.9998), and KneadData, 0.9997 (0.9989, 0.9998)) outperformed the *k*-mer software (KMCP, 0.8947 (0.8133, 0.9748); Kraken2, 0.9891 (0.9832, 0.9974)), with the exception of KrakenUniq (0.9998 (0.9994, 0.9999)), which consistently exhibited a high and stable performance (Figure 3A). However, the alignment-based software exhibited lower precision

performance (BWA, 0.9980 (0.9853, 0.9996), Bowtie2, 0.9999 (0.9999, 0.9999), and KneadData, 0.9981 (0.9971, 0.9998)), potentially leading to an increased number of false positives associated with the host genome. This implied that some microbiome reads may be erroneously mapped as part of the host genome and subsequently was removed as contamination. Conversely, *k*-mer software (KMCP, 0.7686 (0.7477, 0.7925), Kraken2 0.9787 (0.9787, 0.9823), and KrakenUniq 0.9999 (0.9999, 1)) showed lower recall performance, leading to an increased number of false negatives associated with the host genome. This suggested that some host reads may be erroneously unmapped, thereby retaining some host contamination in the downstream analyses (Figure 3B). For F1-score (2 * Precision * Recall/ (Precision + Recall)), the type of microbiome, host type, and the proportion of host genome all influenced the performance on these tools. Notably, BWA, KneadData, and KrakenUniq performed significantly better on human datasets compared to rice datasets. Conversely, Bowtie2 showed great performance with rice dataset (Figure 3C).

We compared the composition of the metagenomic dataset with a synthetic community (SynCom) after host decontamination using the abovementioned six software (Figure 2A). The classes Magnoliopsida and Mammalia displayed low values (i.e., the $\log_{10}$-transformed relative abundance) in BWA, KneadData, and KrakenUniq (Figure 3D). This suggested that BWA and KneadData that belong to the alignment-based software category exhibit superior performance in host contamination removal performance, and KrakenUniq that falls into the *k*-mer based software category tends to remove less host contamination. All tools, except for KMCP, identified some few groups, which belongs to Actinomycetes, Clostridia, Negativicutes, as host contamination, and removed them from the raw data (Figure 3D). For the integrated comparison of resource consumption and the performance of host decontamination across software, we normalized all data using min-max normalization. This enables a comparative analysis of computational efficiency and host contamination removal effectiveness across simulated datasets (Figure 3E, Figure S5). Based on the summarized normalized data, Kraken2 showed significant excellence ($P < 0.05$) under the high levels (90%) of host contamination, in both human (7.8093 (7.7236, 7.8215)) and rice (7.8268 (7.7109, 7.8330)) datasets through comprehensive comparisons. Additionally, when comparing the host removal performance among alignment-based software, focusing on normalized accuracy (NA), normalized precision (NP), normalized recall (NR), and normalized F1-score (NF1) under the high levels (90%) of host contamination, KneadData demonstrated significant superiority ($P < 0.05$) in human (3.9996 (3.9996, 3.9997)).

The absence of host reference genome affected the decontamination performance

Next, we assessed the impact of lacking a host reference genome on the effectiveness of existing host decontamination software. Three *Oryza* species—*Oryza sativa japonica* (GWHBFOO00000000, Osj), *Oryza sativa indica* (GWHBFPT00000000, Osi), and *Oryza rufipogon* (GWHBFHN00000000, Or)—were selected as the resource of host metagenomic reads to generate simulated datasets. *Oryza sativa indica* (GWHBFPX00000000, Refer) was chosen as

the reference genome (Figure 4A). The average nucleotide identity (ANI) between the three species (Osj, Osi, Or) and the reference genome, showed that Osi had the highest similarity (99.01%) to the reference genome, followed by Osj at 97.94% and Or at 97.49% (Figure 4B). The simulated metagenomic reads derived from a single bacterium (SinBac) was generated as before, resulting in three datasets (OsjSinBac, OsiSinBac, OrSinBac) contained varying levels of host DNA contamination (10%, 50%, and 90%) and each 10 Gbps in size. Subsquently, the indexing databases for the six tools were built using the reference genome, and we compared the performance of host decontamination tools (BWA, Bowtie2, KneadData, KMCP, Kraken2 and KrakenUniq) under these conditions (Figure 4A).

In terms of time and memory consumption, KneadData and KrakenUniq used more memory (Figure S6A), while alignment software took more time than *k*-mer software (Figure S6B), as previously described. When comparing with reference metagenomic data, processing OsjSinBac, OsiSinBac, OrSinBac data resulted in all software requiring significantly more time (Figure S6B). Notably, the absence of a host reference genome negatively impacted the decontamination performance of all tools (Figure 4C). Specifically, accuracy, recall, and F1-score were significantly lower for datasets representing OsjSinBac, OsiSinBac, and OrSinBac compared to reference data aligned to the indexing database created with the reference genome. Precision, however, did not show significant differences, indicating that decontaminated datasets still contained some residual host reads.

Then we compared the comprehensive performance for all software during the absence of host reference genome. Based on the summarized normalized data of resource consumption during running and host decontamination metrics (accuracy, precision, recall, and F1-score). All software performed better with reference metagenomic data (5.41 (5.15, 5.91)) than with OsjSinBac (4.93 (4.32, 5.50)), OsiSinBac (4.54 (3.91, 5.35)), OrSinBac (4.64 (4.08, 5.19)) datasets, whereas less difference across these datasets was observed with Kraken2 and KrakenUniq (Figure S6C). Moreover, while different tools performed better on high-host contamination samples with a reference genome, this advantage was not evident in the absence of a reference genome. In the presence of 90% host contamination, all tools showed a significant reduction in accuracy (Figure 4D, Table S4-1), emphasizing the importance of a host reference genome for high contamination metagenomic data.

Methods

Literature searches and data collection

A literature search was conducted in the Web of Science Core Collection Database on April 18th, 2024, using the search terms 'metagenome' and 'microbiome' (Table S1-1). Only research articles were utilized to gather information on software usage (Figure S1A). We excluded publications solely focused on amplicon sequencing data, long-read metagenomes, environmental samples or food samples. Subsequently, we compiled the percentage of publications mentioning host contamination removing and the number of publications for each software.

Simulated dataset description for the downstream analysis

Three groups of data (S1, S2, S3) with 90% host contamination were simulated, including genomes from 30 microbiota species and *Homo sapiens* (GRCh38). Each dataset contained 0.1 million reads of microbiome data and 27 million reads of *Homo sapiens* (Table S1-2). Among the microbiome species, six belonged to Actinomycetota, 15 to Bacillota, six to Bacteroidota, and one each to Pseudomonadota, Thermodesulfobacteriota, and Verrucomicrobiota. To remove host contamination from the raw data (Raw), we used KneadData, a bioinformatics tool specifically designed for this purpose. The output from KneadData processing constituted the host-depleteddata (Remove). And the 30 microbial groups served as a negative control (Microbiome) to evaluate the accuracy and effectiveness of the host contamination removal process.

Metagenomic analysis

In order to compare the difference of analysis in direct data (Raw data) and the host contamination removing data (Remove data), we selected 10 Gbps synthetic community datasets of human with varying host contamination levels. The resource consumption was tested as following description, and metagenomic analysis directly refer to the steps of EasyMetagenome 1.10 pipeline [24]. Briefly, taxonomic profiling was performed using Kraken2 [25], with PlusPFP database, and relative abundances were obtained using Bracken. Functional profiling was preformed via HUMAnN3 [26] using Uniref90 gene families. After assembling the metagenomic data using Megahit 1.0. [27], metagenomic binning and bin refinement were conducted using MetaWRAP [28]. Redundancies in the metagenome-assembled genomes (MAGs) were removed with dRep v2.6.2. MAGs were annotated using GTDBtk v2.3.2 [29], and their quality was evaluated using CheckM2 v1.0.1 [30]. Gene prediction was performed using Prodigal v2.6.3 [31], clustering of genes with CD-HIT v4.8.1 [32], quantification of genes with salmon v1.8.0, and gene annotation with emapper v2.1.6. Then α diversity and β diversity analyses was analyzed using R 4.2.3 as descripted in EasyAmplicon [33]. The completeness and contamination rate of metagenome assembly genomes (MAGs) were normal measurement data, thus they were presented as median (*P25*, *P75*). And we also annotated the GO terms with eggnog-mapper, and calculated their correlation with microbiome data, that only retained microbiome from raw data, using Spearman. The pipeline could be found in https://github.com/YunyunGao374/HostPurge/blob/main/0HostDecontaminationImpactiononDownstreamAnalysis.sh.

Simulated dataset description for the comparison of six tools using human and rice data

Six tools were selected for analysis, three of which are alignment-based software (BWA, Bowtie2, and KneadData), while the others are *k*-mer based (KMCP, Kraken2, KrakenUniq). Simulated datasets were generated using CAMISIM, and analyses were conducted using default or author-recommended parameters. To ensure comparability and reliability, each dataset comprised five replicates. These datasets covered various data sizes (10 Gbps, 30 Gbps, 60 Gbps), different level of host DNA contamination (10%, 50%, 90%), and diverse microbial complexities (single bacterium,

SinBac, or synthetic community, SynCom) from both human and rice samples (Figure 2A). Each dataset had five replicates per condition. The synthetic rice community consists of common microorganisms randomly selected from widely reported strains, while the synthetic human community refers to a microbial community previously published [34]. The code for the generation of simulated data can be found in https://github.com/YunyunGao374/HostPurge/blob/main/1HostDecontaminationSoftwareComparison.sh.Taxonomy information and their genome IDs are provided in Table S2-1.

To ensure comprehensive evaluation, we generated 1080 simulated datasets using CAMISIM. These datasets encompass three distinct sizes (10 Gbps, 30 Gbps, and 60 Gbps) and representing both simple (SinBac) and complex (SynCom) microbiomes. The simulations were conducted separately for human (*Homo sapiens*, GRCh38) and rice (*Oryza sativa indica*, GWHBFPX00000000) hosts, each with three levels of host contamination (10%, 50%, and 90%), enabling a nuanced exploration of host genome contamination removal across various conditions (Figure 1A). For the species which contains multiple chromosomes, we just download all of them and stimulated the information, and all of reference and fasta information have been attached in Table S2-1. Here, for convenience, we have assigned abbreviations to various datasets. For example, SinBac10-1 denoted a 10 Gbps dataset with 90% of reads originating from the host genome and 10% from single bacteria genome. Similarly, SynCom 30-2, represented a 30 Gbps dataset with an even split of 50% reads from the host genome and 50% from the synthetic community genome. Based on these simulated data, we assessed the impact of metagenomic sequencing on microbiome by removing host contamination.

Simulated dataset description for the comparison of six tools within a genus level

To assess the performance of host decontamination tools at the genus level, we utilized three rice species and simple microbiomes separately: *Oryza sativa japonica* (GWHBFOO00000000, Osj), and *Oryza sativa indica* (GWHBFPT00000000, Osi), *Oryza rufipogon* (GWHBFHN00000000, Or). For each species, we generated datasets by combining the host genome with a simple bacterial genome, resulting in three separate datasets: Or with simple bacteria (OrSinBac), Osj with simple bacteria (OsjSinBac), and Osi with simple bacteria (OsiSinBac). Each dataset was simulated using CAMISIM with different levels of host DNA contamination (10%, 50%, and 90%) for 10 Gbps datasets, with five replicates per condition.

Oryza sativa indica served as the reference genome (GWHBFPX00000000, Refer), and indices were created for six tools (BWA, Bowtie2, KneadData, KMCP, Kraken2, KrakenUniq), as described above. Detailed fasta information is provided in Table S2-1. These simulated datasets were analyzed to compare the performance of the six tools in removing host contamination within the genus. The comparison involved evaluating computational resources and the effectiveness of host decontamination. Genome similarity was calculated using fastANI v1.34 [35], and the impact of metagenomic sequencing on microbiome have been assessed with the tested of following performance metrics. Based on these datasets, we aimed to determine how each tool performs in

removing host contamination when genome data is limited within the same genus.

Performance metrics tests

All software utilized eight threads for building the database and running the processes across simulated datasets, and the running code of different software can be found at https://github.com/YunyunGao374/HostPurge/blob/main/1HostDecontaminationSoftwareComparison.sh. We assessed a comparative analysis of resource consumption, focusing on maximum RAM usage and processing time. Additionally, we evaluated their performance metrics including true positive (TP), true negative (TN), false positive (FP), and false negative (FN). And the precision, recall and F1-score were also calculated, with the following formulas: Accuracy = (TP + TN) / (TP + FN + FP + TN), Precision = (TP / (TP + FP)), Recall = (TP / (TP + FN)), F1-score = 2 * Precision * Recall / (Precision + Recall) [36]. Then all bioinformatics analysis were all analyzed within R 4.2.3. We conducted normality and homogeneity tests on all data. Measurement data were expressed as mean ±SE for normally distributed data and as median (*P25, P75*) for non-normally distributed data. For normally distributed data with homogeneous variances in two-group comparisons, we used paired t-tests. Non-normally distributed or non-homogeneous data were analyzed using non-parametric Wilcoxon tests. In multiple group comparisons, normally distributed data with homogeneous variances were analyzed with ANOVA, while non-normally distributed or non-homogeneous data were analyzed with non-parametric Kruskal-Wallis tests [37]. Bonferroni post-hoc tests were performed on the data within each group to analyze the differences between different datasets [38]. For the average species composition plot, when dealing with data containing zeros, we adopted the straightforward method of adding a positive constant (0.001) to all leaf trait values [39], and then taking the logarithm of the resulting average relative abundance. For the integrated comparison of resource consumption and the performance of host purge in each software, we normalized all data use min-max normalization [40], which is a technique that performs a linear transformation of the original data. Data visualization was done using ggplot2 package, and $p \leqslant 0.05$ was regarded as statistically significant. All relevant data and plot code can be found at https://github.com/YunyunGao374/HostPurge.

Discussion

While 57.94% of publications eliminated host contamination before analyzing their data, while the remaining opted to analyze their data directly (Figure S1B), potentially sacrificing valuable microbiome information in the process. Some software was developed to decontaminate the host genome [9, 17-21], but limited research provided the distinctions among them. For accelerating the reproducibility, comparability, and standardization of metagenomic data, it is important to understand how these different tools, broadly referred to downstream analyses [11]. And the significant influence of function annotation, species annotation, reads assembly and binning in the consumption of computational resources, exists the necessity to remove host contamination, particularly in ultra-high-depth sequencing data.

In our study, we generated a simulated dataset with 0.1 million of 30 microbiome species, and 27 million of *Homo sapiens*. Among the microbiome species, six belonged to Actinomycetota, 15 to Bacillota, six to Bacteroidota, one each to Pseudomonadota, Thermodesulfobacteriota, and Verrucomicrobiota (Table S1-2). However, the proportion of the relative abundance were not qual, and two phyla not included in the simulation (Streptophyta and Campylobacterota) were annotated (Figure 1C). In addition, although each of simulated data comprises 30 microbial groups, we only obtained 14 MAGs. These may be attributed to the heterogeneity of our simulated data or the potential need for further improvement of the assembly and binning software. Nonetheless, the importance of the host decontamination was still being demonstrated in terms of gene functional annotation. Meanwhile, the significant difference in the Shannon index between microbiome (negative control) data and raw or removed data highlights the importance of establishing a 'gold standard' for host removal.

It is noteworthy that we observed significantly higher accuracy, precision, and F1-score in datasets with high (90%) compared to low (10%) levels of host contamination in KneadData. Similar trends were also observed in other alignment software (Tables S3-5, S3-7). This phenomenon may be attributed to two factors. Firstly, our simulated data, derived from the host genome used as the indexing reference database, resulted in a higher proportion of host reads for high host contamination data (90%) aligning to the host reference in alignment software (BWA, Bowtie2, KneadData). Secondly, reads from the microbiome may be challenging to distinguish from reference genomes, leading to a higher number of microbiome reads being discarded in datasets with lower host contamination (10%).

Additionally, the challenge of predicting the host genome when it's unavailable, potentially down to the genus, family, or order level, needs consideration. Interestingly, despite Osi having a closer ANI to the reference genome compared to Osj, the decontamination performance for OsiSinBac was not as effective as for OsjSinBac (Figure 4C). And the high host contamination showed a significant reduction of accuracy in all software (Figure 4D), emphasizing the importance of a host reference genome for high contamination metagenomic data [41]. In the future, advances in artificial intelligence algorithms [42] and long-read sequencing may help overcome not only host contamination but also the problem of horizontal transfer of bacteria.

Conclusion

In conclusion, host decontamination not only speeds up downstream analysis but also enhances the accuracy of gene function annotation, particularly in ultra-high-depth sequencing data. And each of these tools (BWA, Bowtie2, KneadData, Krakne2, KMCP and KrakenUniq) offers unique strengths that can be harnessed based on the specific requirements of a research study. Briefly, Bowtie2 and KneadData provides more accurate removal capabilities, albeit with increased computational demands. Kraken2 and KrakenUniq offer fast and user-friendly solution, while KMCP can retain more low-abundance taxa. When reference genomes are lacking, BWA and KneadData are less

impacted among alignment software, and Kraken2 and KrakenUniq are less affected among *k*-mer software.

Understanding the trade-offs between speed, accuracy, and computational resources is crucial for selecting the most suitable tool for host DNA removal in metagenomic analyses. As research increasingly focuses on understanding the impact of host contamination on microbiome annotation, particularly for low abundance taxa [41], this study provides a comprehensive evaluation that lays the groundwork for refining tools and methodologies. Ultimately, these advancements will empower researchers to derive meaningful biological insights from complex metagenomic datasets.

Acknowledgments

The work was financially supported by the China Postdoctoral Science Foundation (2024M753580) and National Natural Science Foundation of China (U23A20148). We would like to acknowledge the support of the Prof. Wei Shen (Institute for Viral Hepatitis, The Second Affiliated Hospital, Chongqing Medical University, China) for any help during running the KMCP. And we sincerely thank Dr. Yao Wang (Agricultural Genomics Institute at Shenzhen, Chinese Academy of Agricultural Sciences, China) for her help in data analyses, Dr. Kai Peng (Yangzhou University, China) for his help in drawing the spider diagram.

Authors' contributions

Yong-Xin Liu, Shi Huang and Yunyun Gao conceived and coordinated the study. Yunyun Gao, Hao Luo, and Haifei Yang collected and analyzed the simulated data. Yunyun Gao and Hujie Lyu did the literature searches and data collection. Yong-Xin Liu, Shi Huang, Salsabeel Yousuf and Yunyun Gao revised the manuscript. All authors have read the final manuscript and approved it for publication.

Conflict of interest

The authors declare that they have no conflicts of interest.

Data availability

The raw data of simulated metagenomic sequencing reads have been deposited at the Genome Warehouse (GWH) (https://bigd.big.ac.cn/gwh/) under PRJCA028271. All pipelines, data analyses, and plotting code can be found at https://github.com/YunyunGao374/HostPurge.

References

1. Rooks MG and Garrett WS. Gut microbiota, metabolites and host immunity. *Nature reviews immunology* 2016;6:341-52. https://doi.org/10.1038/nri.2016.42.

2. Gao Y, Li D and Liu Y-X. Microbiome research outlook: past, present, and future. *Protein & Cell* 2023;10:709-12. https://doi.org/10.1093/procel/pwad031.

3. Xia H, Zhang Z, Luo C, et al. MultiPrime: A reliable and efficient tool for targeted next‐generation sequencing. *iMeta* 2023;e143:1-16. https://doi.org/10.1002/imt2.143.

4. Saheb Kashaf S, Almeida A, Segre JA, et al. Recovering prokaryotic genomes from host-associated, short-read shotgun metagenomic sequencing data. *Nature protocols* 2021;5:2520-41. https://doi.org/10.1038/s41596-021-00508-2.

5. Tremblay J, Schreiber L and Greer CW. High-resolution shotgun metagenomics: the more data, the

better? *Briefings in Bioinformatics* 2022;6:bbac443. https://doi.org/10.1093/bib/bbac443.

6. Jin H, Quan K, He Q, et al. A high-quality genome compendium of the human gut microbiome of Inner Mongolians. *Nature Microbiology* 2023;1:150-61. https://doi.org/10.1038/s41564-022-01270-1.

7. Pereira-Marques J, Hout A, Ferreira RM, et al. Impact of host DNA and sequencing depth on the taxonomic resolution of whole metagenome sequencing for microbiome analysis. *Frontiers in microbiology* 2019:1277. https://doi.org/10.3389/fmicb.2019.01277.

8. Wu-Woods NJ, Barlow JT, Trigodet F, et al. Microbial-enrichment method enables high-throughput metagenomic characterization from host-rich samples. *Nature Methods* 2023;11:1672-82. https://doi.org/10.1038/s41592-023-02025-4.

9. Constantinides B, Hunt M and Crook DW. Hostile: accurate decontamination of microbial host sequences. *Bioinformatics* 2023;12:btad728. https://doi.org/10.1093/bioinformatics/btad728.

10. Heravi FS, Zakrzewski M, Vickery K, et al. Host DNA depletion efficiency of microbiome DNA enrichment methods in infected tissue samples. *Journal of microbiological methods* 2020:105856. https://doi.org/10.1016/j.mimet.2020.105856.

11. Marchukov D, Li J, Juillerat P, et al. Benchmarking microbial DNA enrichment protocols from human intestinal biopsies. *Frontiers in genetics* 2023:1184473. https://doi.org/10.3389/fgene.2023.1184473.

12. Cheng WY, Liu W-X, Ding Y, et al. High sensitivity of shotgun metagenomic sequencing in colon tissue biopsy by host DNA depletion. *Genomics, Proteomics & Bioinformatics* 2022; https://doi.org/10.1016/j.gpb.2022.09.003.

13. Feehery GR, Yigit E, Oyola SO, et al. A method for selectively enriching microbial DNA from contaminating vertebrate host DNA. *PloS one* 2013;10:e76096. https://doi.org/10.1371/journal.pone.0076096.

14. Soto-Giron MJ, Kim J-N, Schott E, et al. The edible plant microbiome represents a diverse genetic reservoir with functional potential in the human host. *Scientific Reports* 2021;1:24017. https://doi.org/10.1038/s41598-021-03334-4.

15. Rumbavicius I, Rounge TB and Rognes T. HoCoRT: host contamination removal tool. *BMC bioinformatics* 2023;1:371. https://doi.org/10.1186/s12859-023-05492-w.

16. Liu Y, Ghaffari MH, Ma T, et al. Impact of database choice and confidence score on the performance of taxonomic classification using Kraken2. *aBIOTECH* 2024:1-11. https://doi.org/10.1007/s42994-024-00178-0.

17. Langmead B and Salzberg SL. Fast gapped-read alignment with Bowtie 2. *Nature methods* 2012;4:357-9. https://doi.org/10.1038/nmeth.1923.

18. Li H and Durbin R. Fast and accurate short read alignment with Burrows–Wheeler transform. *bioinformatics* 2009;14:1754-60. https://doi.org/10.1093/bioinformatics/btp324.

19. Lu J, Rincon N, Wood DE, et al. Metagenome analysis using the Kraken software suite. *Nature protocols* 2022;12:2815-39. https://doi.org/10.1038/s41596-022-00738-y.

20. Shen W, Xiang H, Huang T, et al. KMCP: accurate metagenomic profiling of both prokaryotic and viral populations by pseudo-mapping. *Bioinformatics* 2023;1:btac845. https://doi.org/10.1093/bioinformatics/btac845.

21. Schmieder R and Edwards R. Fast identification and removal of sequence contamination from genomic and metagenomic datasets. *PloS one* 2011;3:e17288.

https://doi.org/10.1371/journal.pone.0017288.

22. Nearing JT, Comeau AM and Langille MG. Identifying biases and their potential solutions in human microbiome studies. *Microbiome* 2021;1:113. https://doi.org/10.1186/s40168-021-01059-0.

23. McArdle AJ and Kaforou M. Sensitivity of shotgun metagenomics to host DNA: abundance estimates depend on bioinformatic tools and contamination is the main issue. *Access microbiology* 2020;4:e000104. https://doi.org/10.1099/acmi.0.000104.

24. Liu Y-X, Qin Y, Chen T, et al. A practical guide to amplicon and metagenomic analysis of microbiome data. *Protein & cell* 2021;5:315-30. https://doi.org/10.1007/s13238-020-00724-8.

25. Wood DE, Lu J and Langmead B. Improved metagenomic analysis with Kraken 2. *Genome biology* 2019:1-13. https://doi.org/10.1186/s13059-019-1891-0.

26. Abubucker S, Segata N, Goll J, et al. Metabolic reconstruction for metagenomic data and its application to the human microbiome. *PLoS computational biology* 2012;6:e1002358. https://doi.org/10.1371/journal.pcbi.1002358.

27. Li D, Liu C-M, Luo R, et al. MEGAHIT: an ultra-fast single-node solution for large and complex metagenomics assembly via succinct de Bruijn graph. *Bioinformatics* 2015;10:1674-6. https://doi.org/10.1093/bioinformatics/btv033.

28. Uritskiy GV, DiRuggiero J and Taylor J. MetaWRAP—a flexible pipeline for genome-resolved metagenomic data analysis. *Microbiome* 2018:1-13. https://doi.org/10.1186/s40168-018-0541-1.

29. Chaumeil P-A, Mussig AJ, Hugenholtz P, et al. GTDB-Tk v2: memory friendly classification with the genome taxonomy database. *Bioinformatics* 2022;23:5315-6. https://doi.org/10.1093/bioinformatics/btac672.

30. Chklovski A, Parks DH, Woodcroft BJ, et al. CheckM2: a rapid, scalable and accurate tool for assessing microbial genome quality using machine learning. *Nature Methods* 2023;8:1203-12. https://doi.org/10.1038/s41592-023-01940-w.

31. Hyatt D, Chen G-L, LoCascio PF, et al. Prodigal: prokaryotic gene recognition and translation initiation site identification. *BMC bioinformatics* 2010:1-11. https://doi.org/10.1186/1471-2105-11-119.

32. Fu L, Niu B, Zhu Z, et al. CD-HIT: accelerated for clustering the next-generation sequencing data. *Bioinformatics* 2012;23:3150-2. https://doi.org/10.1093/bioinformatics/bts565.

33. Liu YX, Chen L, Ma T, et al. EasyAmplicon: An easy‐to‐use, open‐source, reproducible, and community‐based pipeline for amplicon data analysis in microbiome research. *iMeta* 2023;1:e83. https://doi.org/10.1002/imt2.83.

34. Cheng AG, Ho P-Y, Aranda-Díaz A, et al. Design, construction, and in vivo augmentation of a complex gut microbiome. *Cell* 2022;19:3617-36. https://doi.org/10.1016/j.cell.2022.08.003.

35. Jain C, Rodriguez-R LM, Phillippy AM, et al. High throughput ANI analysis of 90K prokaryotic genomes reveals clear species boundaries. *Nature communications* 2018;1:5114. https://doi.org/10.1038/s41467-018-07641-9.

36. Qin H, Ou L, Gao J, et al. DENA: training an authentic neural network model using Nanopore sequencing data of Arabidopsis transcripts for detection and quantification of N 6-methyladenosine on RNA. *Genome Biology* 2022;1:25. https://doi.org/10.1186/s13059-021-02598-3.

37. McKight PE and Najab J. Kruskal‐wallis test. *The corsini encyclopedia of psychology* 2010:1-. https://doi.org/10.1002/9780470479216.corpsy0491.

38. Duque M, Lee-Kubli CA, Tufail Y, et al. Sonogenetic control of mammalian cells using exogenous

Transient Receptor Potential A1 channels. *Nature communications* 2022;1:600. https://doi.org/10.1038/s41467-022-28205-y.

39. Harris T, Ottaviani G, Mulligan M, et al. Trait hypervolumes based on natural history collections can detect ecological strategies that are distinct to biogeographic regions. *Journal of Ecology* 2023;2:314-26. https://doi.org/10.1111/1365-2745.14005.

40. Song Q, Lee J, Akter S, et al. Prediction of condition-specific regulatory genes using machine learning. *Nucleic Acids Research* 2020;11:e62-e. https://doi.org/10.1093/nar/gkaa264.

41. Gihawi A, Ge Y, Lu J, et al. Major data analysis errors invalidate cancer microbiome findings. *MBio* 2023;5:e01607-23. https://doi.org/10.1128/mbio.01607-23.

42. Gao Y, Peng K, Bai D, et al. The Microbiome Protocols eBook initiative: Building a bridge to microbiome research. *iMeta* 2024;e182:1-7. https://doi.org/10.1002/imt2.182.

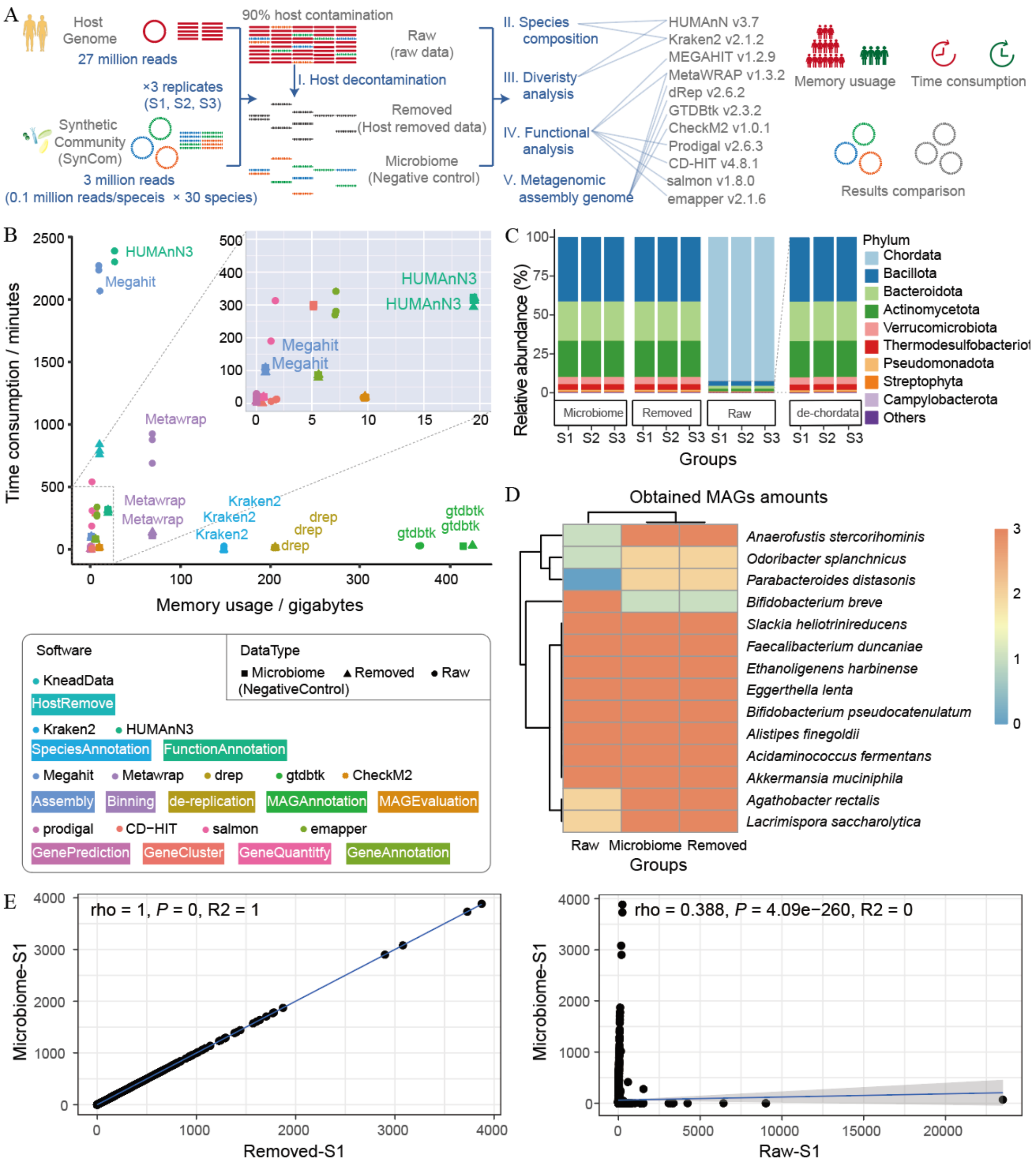

Figure 1. Host contamination consumed extra computing resources and affected the accuracy of the results in metagenomic analysis.

A. Simulated data design and the main downstream pipeline. Three samples (S1, S2, S3) with 90% host contamination were generated from the genomes of 30 bacteria and *Homo sapiens*. The raw data (Raw) underwent host decontamination to produce the removed data (Removed). The microbiome data was used as a negative control (Microbiome). Subsequent downstream analyses included host decontamination, species composition, diversity analysis, functional analysis, and metagenomic assembly genome evaluations. B. Host contamination increased computing resource consumption by 7.63 to 20.55 times in Megahit and HUMAnN3. The performance in terms of time and memory usage during downstream analyses was assessed on three samples (~9 GB per sample). C. The relative abundance at the phylum level. We also displayed the composition of raw data without chordata (de-chordata) to demonstrate that host removal can accurately reflect the true

microbiota composition. D. Evaluation of metagenomic assembly genome (MAG) amounts. Removed data can generate more MAGs than raw data during binning in all samples. E. Correlation assessment of gene ontology (GO) terms between microbiome data and removed data (left) or raw data (right) in S1 group. Each step of analysis was based on three sample replicates, with each replicate consisting of 30 million paired-end 150 bp reads (~ 9 GB).

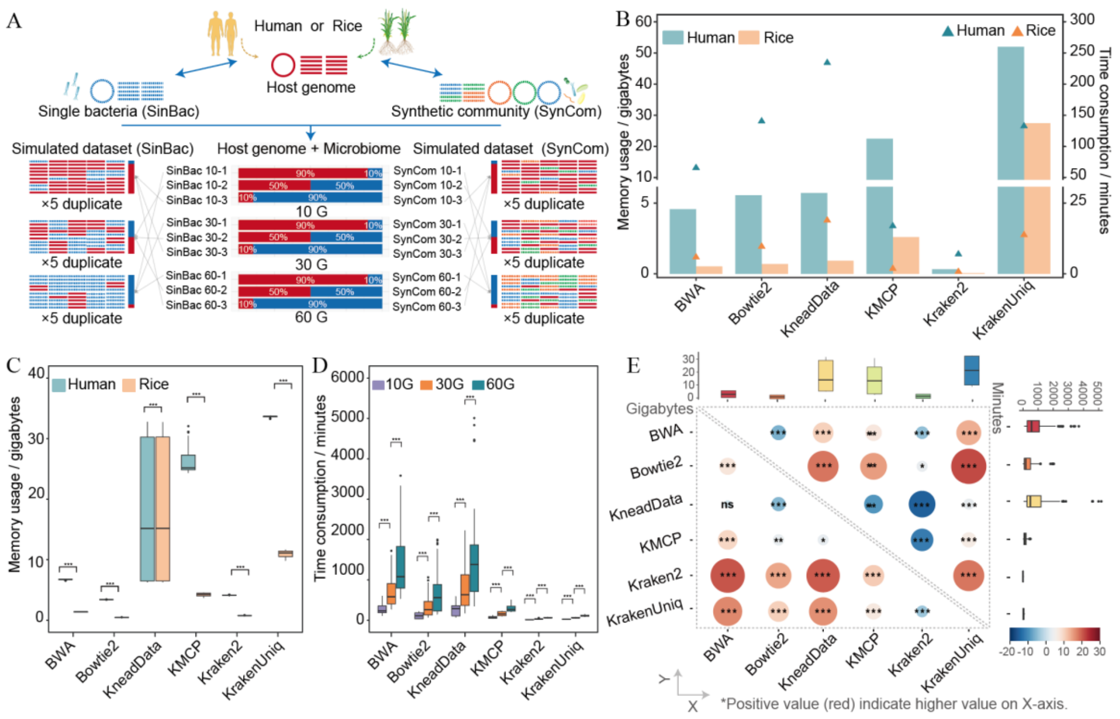

Figure 2. Benchmarking calculates resources of six host removal software on simulated human and rice metagenomic data.

The computational resources of all software were mostly influenced by the host reference genome size and the metagenomic data size, with Kraken2 consistently utilizing minimal computational resources.

A. Simulating metagenome datasets using CAMISIM. The datasets were designed to encompass diverse scenarios, featuring varying proportions of host genome contamination. Derived from human or rice genomes, the datasets come in three different sizes, each containing either a single bacterium (SinBac) or a synthetic community (SynCom, detail in Table S2-1). B. Comparison of time and memory usage in software for indexing host reference genome. The size of reference genome affects the resource consumption, with Kraken2 utilizing the fewest resources during the indexing step. C. Memory usage for different software, measured in giga bytes (Gb). The maximum computational memory usage is influenced by the host reference genome, except in KneadData. D. Running time of different software, showing in minutes. The decontamination process for large datasets requires more time. E. Memory usage (top-right diagonal) and execution time (bottom-left diagonal) among different software based on Kruskal-Wallis test. Positive values (red circle) indicate higher time or memory requirements for the software on the X-axis. The size of the circles

represents the Z-value, which is the standardized scores corresponding to each pairwise comparison. Kraken2 was observed to use significantly lower time and memory usage compared to others. '*' is shown as significant difference. (ns, not significant; *, $P \leqslant 0.05$; **, $P \leqslant 0.01$; ***, $P \leqslant 0.001$.)

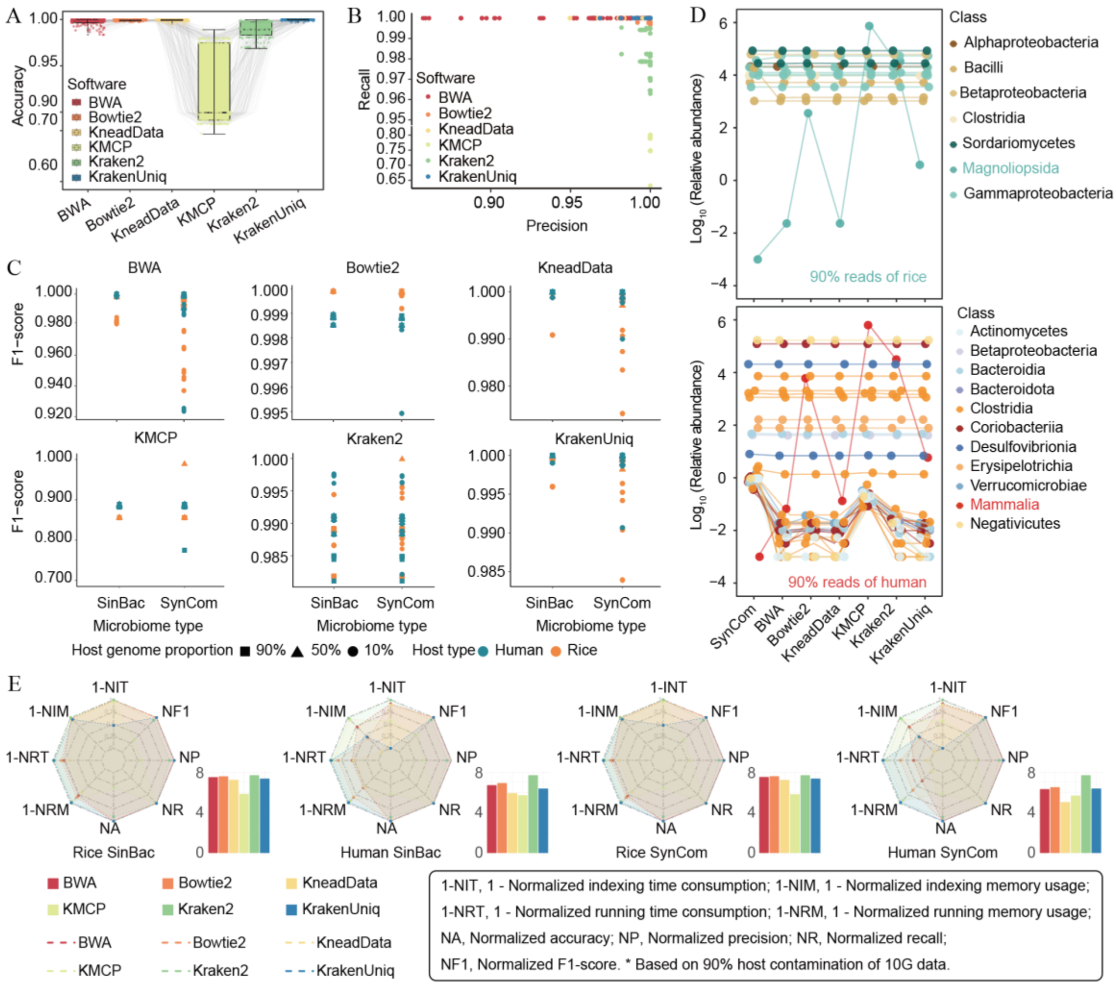

Figure 3. Assessing the accuracy of host contamination removal across various software. The alignment-based software displayed a higher rate of false positives, consequently diminishing the accuracy of microbiome information, while *k*-mer software exhibited an elevated occurrence of false negatives, thereby contributing to contamination of the data with host genome sequences. A. Accuracy among six software, showing BWA, Bowtie2, KneadData and KrakenUniq perform well. Accuracy = (True positive + True negative) / (True positive + True negative + False positive + False negative). B. Precision-recall of software. The alignment-based software (BWA, Bowtie2 and KneadData) exhibited higher false positive (some microbiota reads misaligned as host genome for removal), resulting in reduced microbiome information. However, the *k*-mer software (KMCP, Kraken2 and KrakenUniq) showed increased false negative (some host reads not be found), leading to the host genome contamination. Precision = Ture positive / (Ture positive+ False positive), Recall = True positive/ (True positive + False negative). C. High host contamination rate and microbiome complex rate reduce F1-score in six software. F1-score = 2 * Precision * Recall/ (Precision + Recall). D. Composition of the metagenomic dataset with a synthetic community after host contamination

removal using six software based on 90% host contamination. BWA and KneadData retained lower host contamination in alignment-based software, and KrakenUniq and Kraken2 retained lower host contamination in *k*-mer based software. E. Comparative analysis of computational efficiency and host contamination removal performance across simulated 60 Gbps datasets with 90% host contamination. The bar plot presented summary values for all indicators, highlighting Kraken2's excellence in comprehensive comparisons. The abbreviations for the indicators are as follows: 1-NIT, 1 - Normalized indexing time consumption; 1-NIM, 1 - Normalized indexing memory usage; 1-NRT, 1 - Normalized running time consumption; 1-NRM, 1 - Normalized running memory usage; NA, Normalized accuracy; NP, Normalized precision; NR, Normalized recall; NF1, Normalized F1-score. SinBac, Single bacterium; SynCom, Synthetic community.

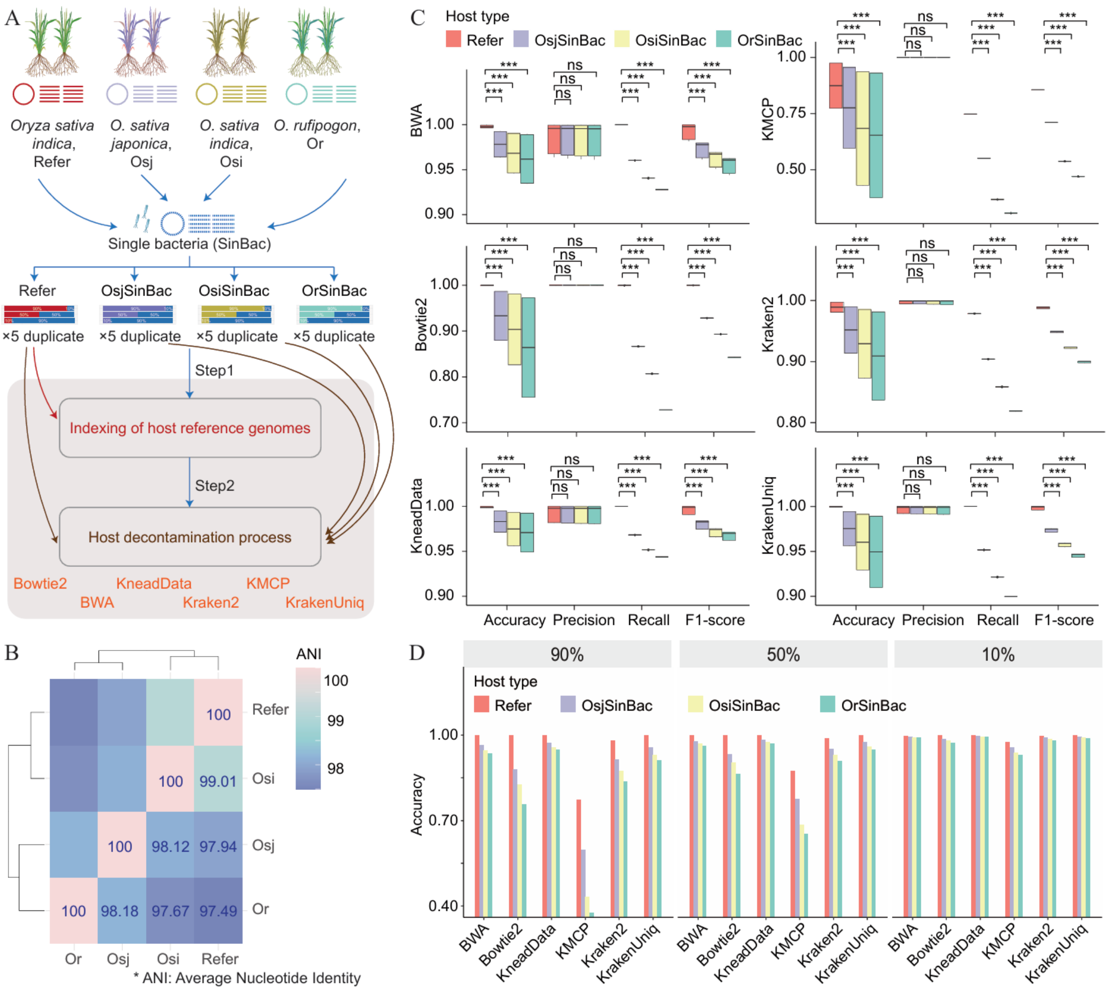

Figure 4. Impact of lacking a host reference genome on the performance of host decontamination tools

A. Impact of the absence of host reference genome on host decontamination tools. Simulated datasets were derived from three *Oryza* species (*Oryza sativa japonica*, Osj; *Oryza sativa indica*, Osi, *Oryza rufipogon*, Or) as hosts and a single bacterium. Each dataset contained varying levels of host DNA contamination (10%, 50%, and 90%) and was 10 Gbps in size, with five replicates per condition. The reference genome of *Oryza sativa indica* (refer) was used to create the indexing database for various host removal tools. All simulated data were aligned to this reference database to evaluate the performance of these tools in the absence of a specific host reference genome. B. Average nucleotide identity (ANI) analysis using FastANI. AIN values for *Oryza sativa japonica* (Osj), *Oryza sativa indica* (Osi), *Oryza rufipogon* (Or) and reference genome (*Oryza sativa indica*, refer) were shown. Osi (99.01%) showed the highest similarity to the reference genome, followed by Osj (97.94%) and Or (97.49%). C. Accuracy, precision, recall and F1-score of six tools on the simulated metagenomic data from the *Oryza* genus. All tools demonstrated significantly lower accuracy, recall, and F1-score for OsjSinBac, OsiSinBac, and OrSinBac compared to the reference data when aligned to the indexing database, which was created using the reference genome. D. Accuracy index of different software across various host genome proportion (90%, 50%, 10%). High host contamination of metagenomic data in the absence of host reference genome notably

affected the performance of existing host removal tools.

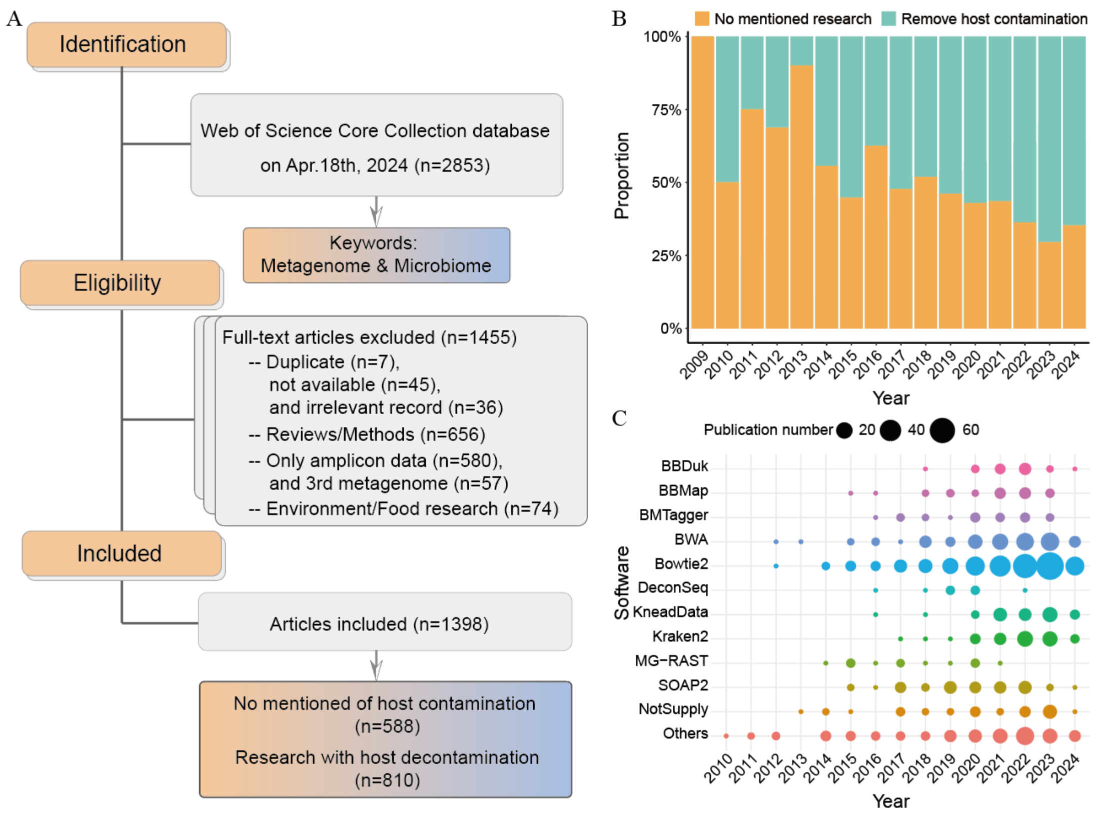

Figure S1. Literature search for existing studies addressing host contamination.

A. Literature search criteria. B. The proportion of publications mentioning the removal of host contamination. C. The existing software utilized for removing host genomes.

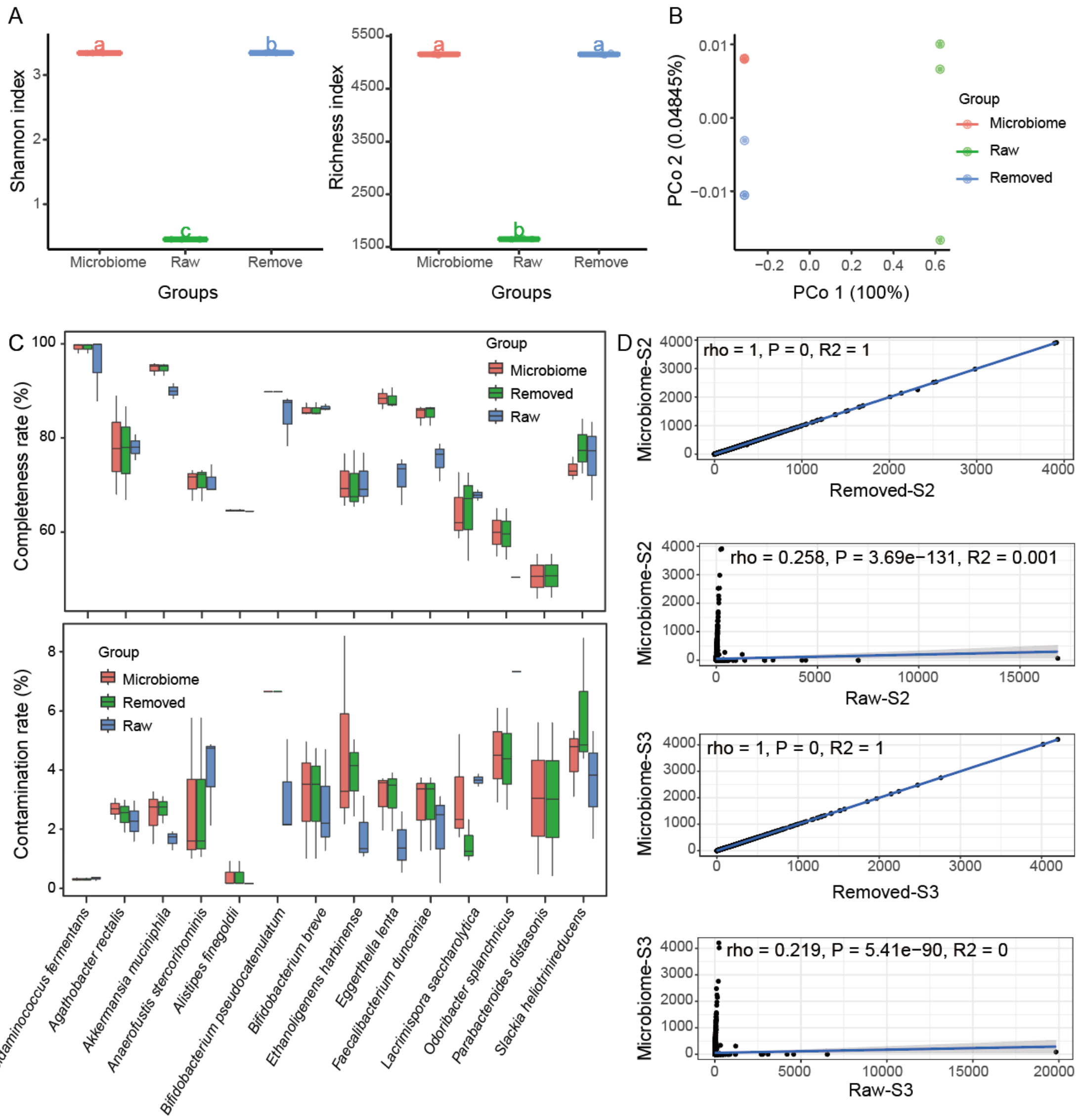

Figure S2. Comparison of host removal performance on the accuracy of the results in metagenomic analysis.

A. Shannon index, richness index, and principal coordinates analysis (PCoA) across microbiome removed and raw data. B. The completeness and contamination rates of MAGs (metagenome-assembled genomes) were assessed in microbiome, removed and raw data. C. Correlation assessment of gene ontology (GO) terms between microbiome data and removed data or raw data in S2 and S3 samples.

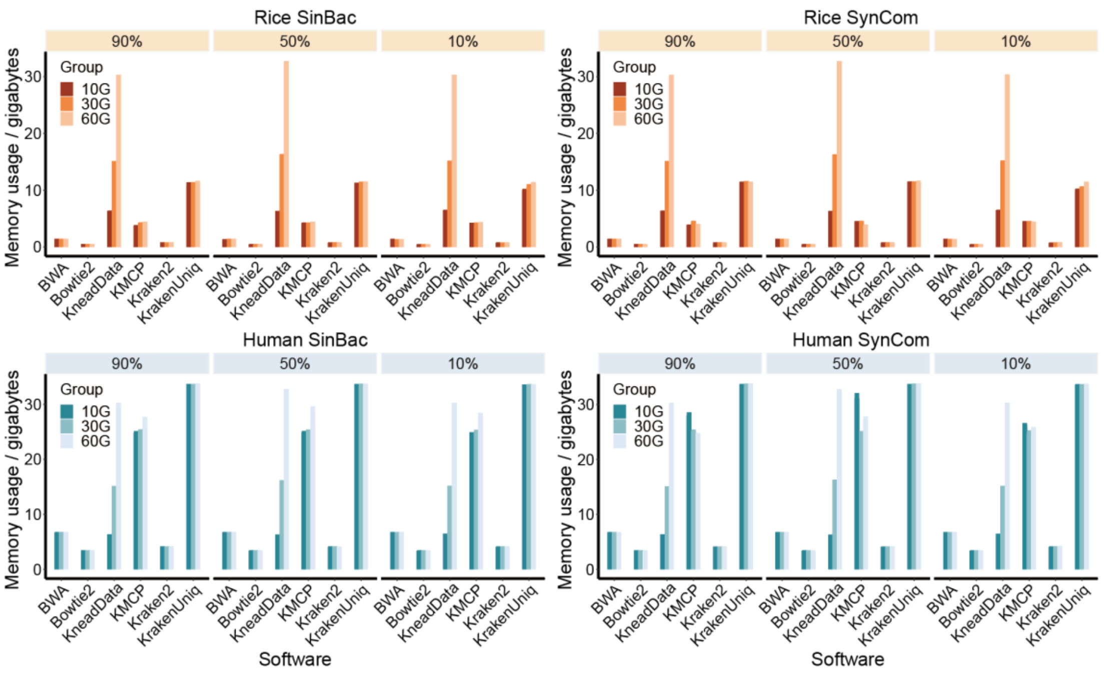

Figure S3. Memory usage during the host removing process of BWA, Bowtie2, KneadData, KMCP, Kraken2, KrakenUniq in simulation rice and human metagenome. SinBac, Single Bacteria; SynCom, Synthetic Community.

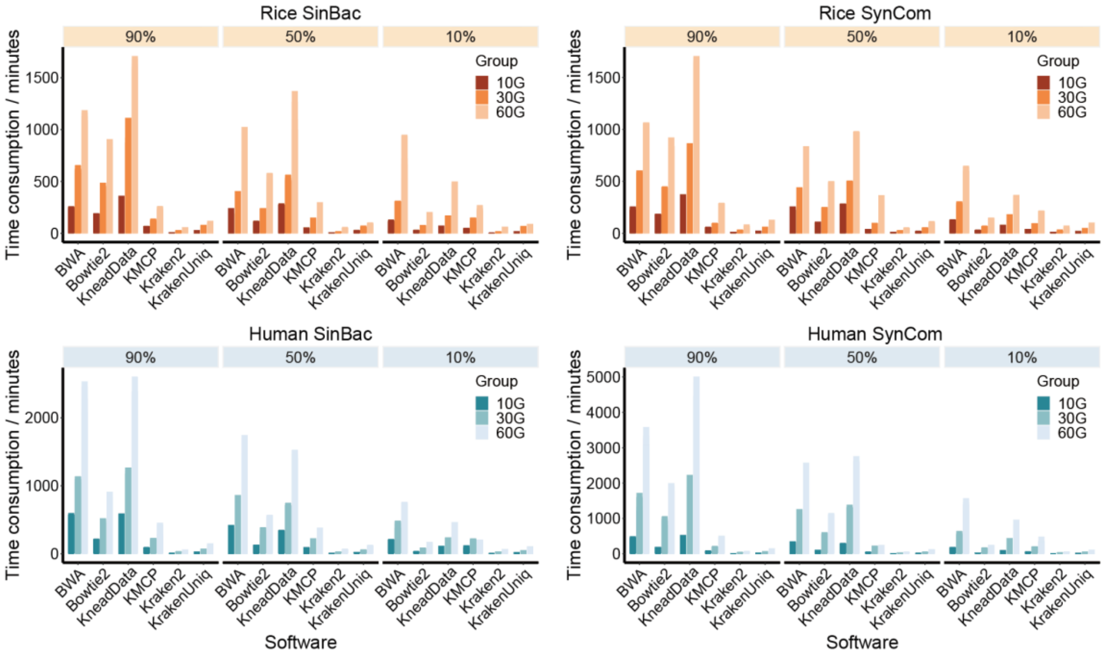

Figure S4. Time consumption during the host removing process of BWA, Bowtie2, KneadData, KMCP, Kraken2, KrakenUniq. SinBac, Single bacterium; SynCom, Synthetic community.

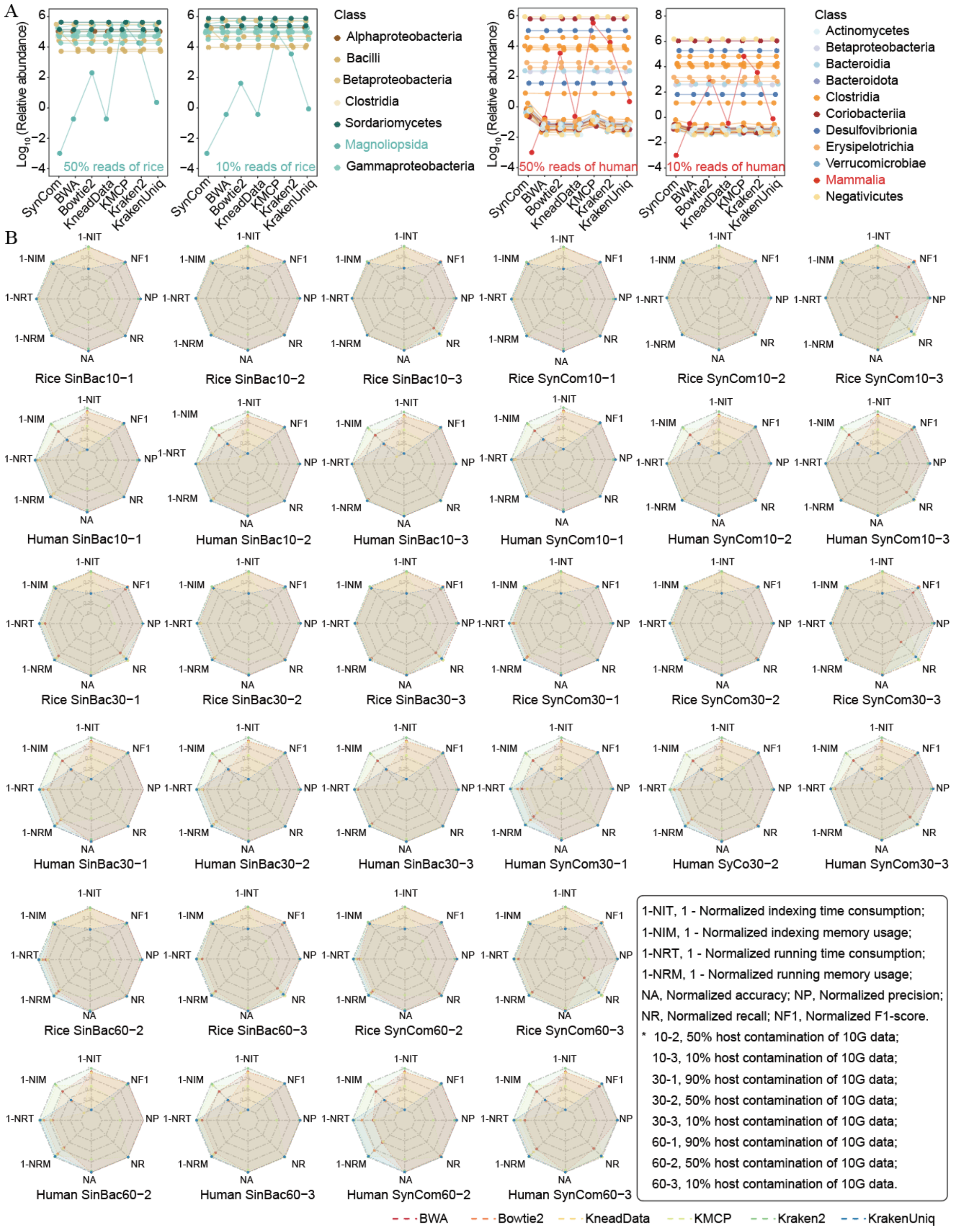

Figure S5. Comparative analysis of composition, computational efficiency and host contamination removal performance across six software.

A. Composition of the metagenomic dataset with a synthetic community after host contamination removal using six software. B. Computational efficiency and host contamination removal performance. 1-NIT, 1 - Normalized indexing time consumption; 1-NIM, 1 - Normalized indexing memory usage; 1-NRT, 1 - Normalized running time consumption; 1-NRM, 1 - Normalized running memory usage; NA, Normalized accuracy; NP, Normalized precision; NR, Normalized recall; NF1, Normalized F1-score. SinBac, Single bacterium; SynCom, Synthetic community.

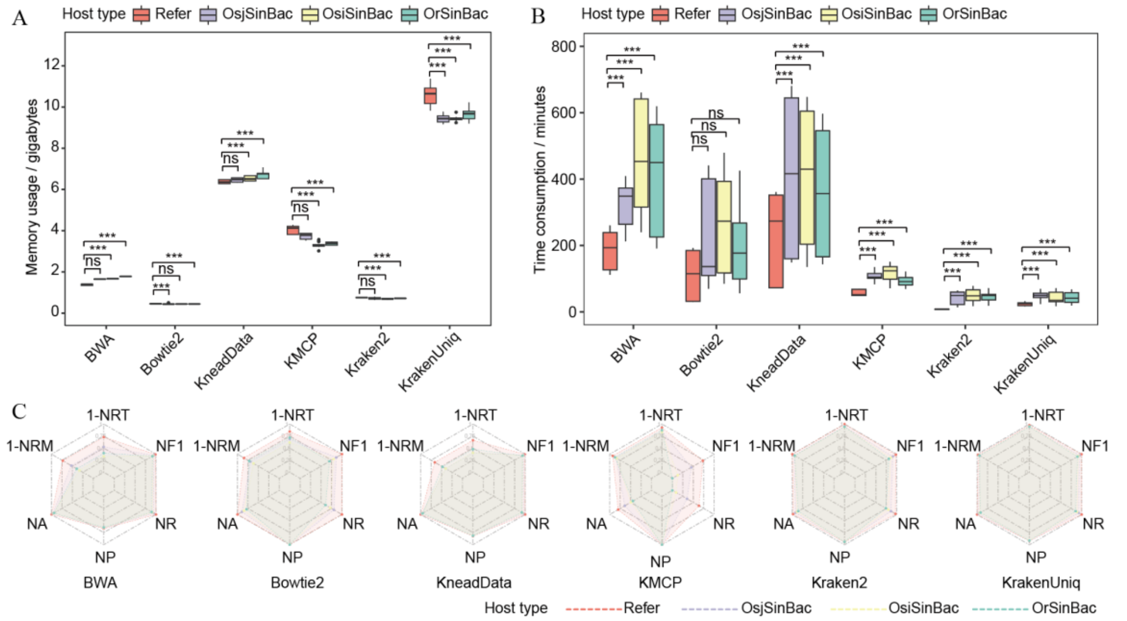

Figure S6. Assessing the performance of six tools at the genus level.

A. Memory usage during the host removing process for six tools across OsjSinBac, OsiSinBac, OrSinBac and reference metagenomic data. B. Time consumption during the host removing process for six tools across OsjSinBac, OsiSinBac, OrSinBac and reference metagenomic data. C. Computational efficiency and host contamination removal performance for six tools across OsjSinBac, OsiSinBac, OrSinBac and reference metagenomic data. 1-NRT, 1 - Normalized running time consumption; 1-NRM, 1 - Normalized running memory usage; NA, Normalized accuracy; NP, Normalized precision; NR, Normalized recall; NF1, Normalized F1-score. OsjSinBac, *Oryza sativa japonica* with a single bacterium; OsiSinBac, *Oryza sativa indica* with a single bacterium; OrSinBac, *Oryza rufipogon* with a single bacterium.

Figure1

Click here to access/download;Figure;Figure1.pdf

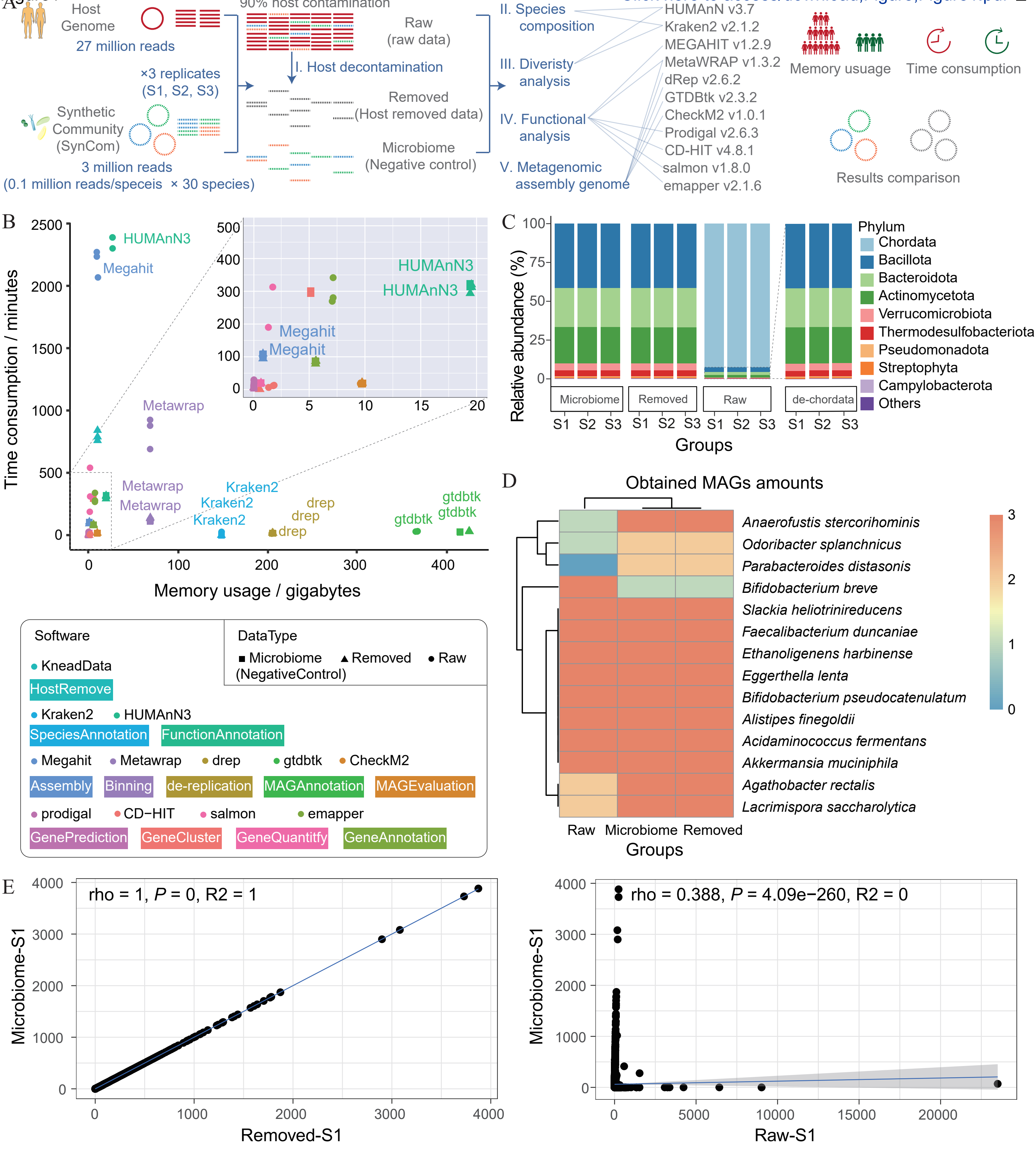

Figure2

Click here to access/download;Figure;Figure2.pdf

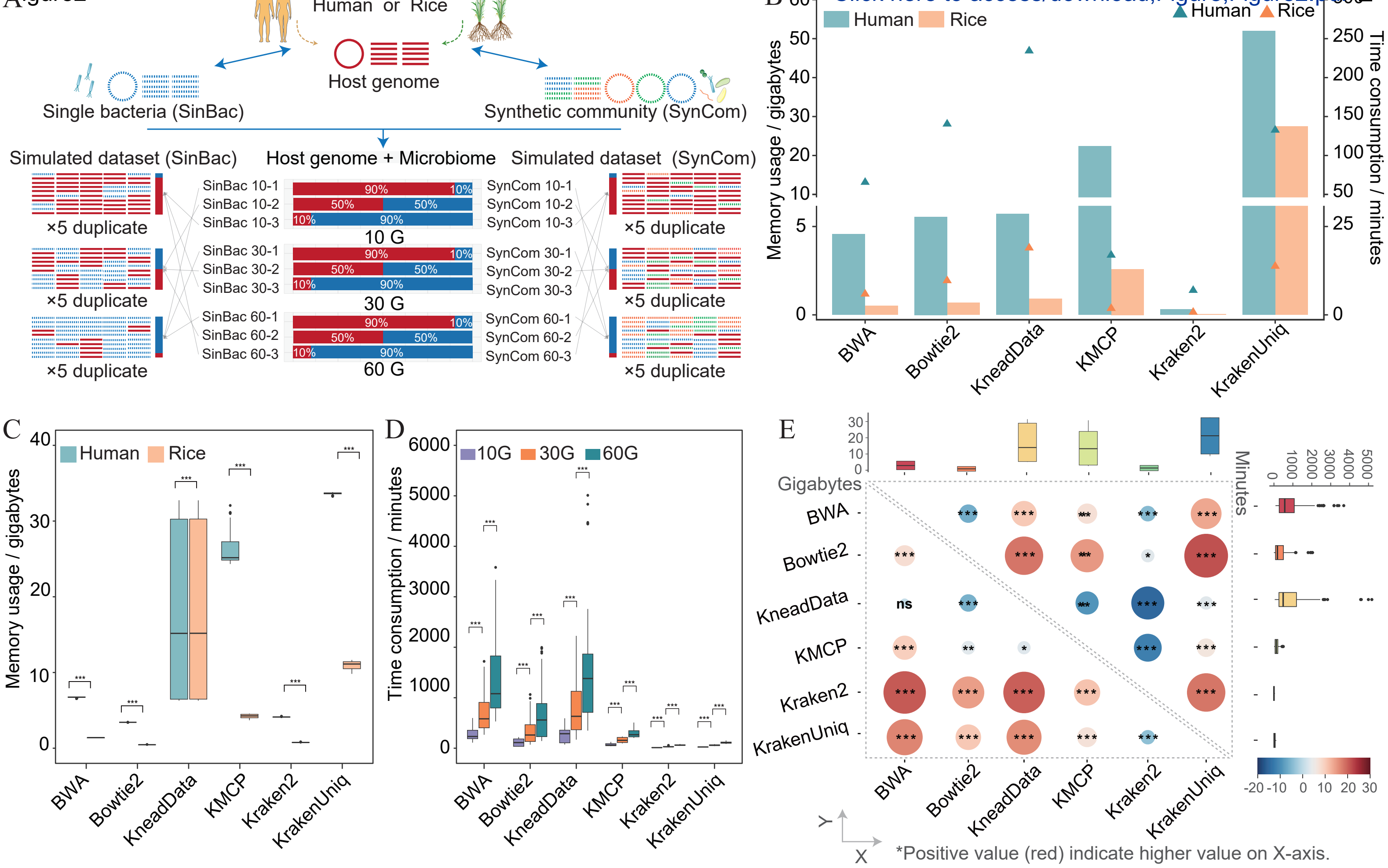

Figure3

Click here to access/download;Figure;Figure3.pdf

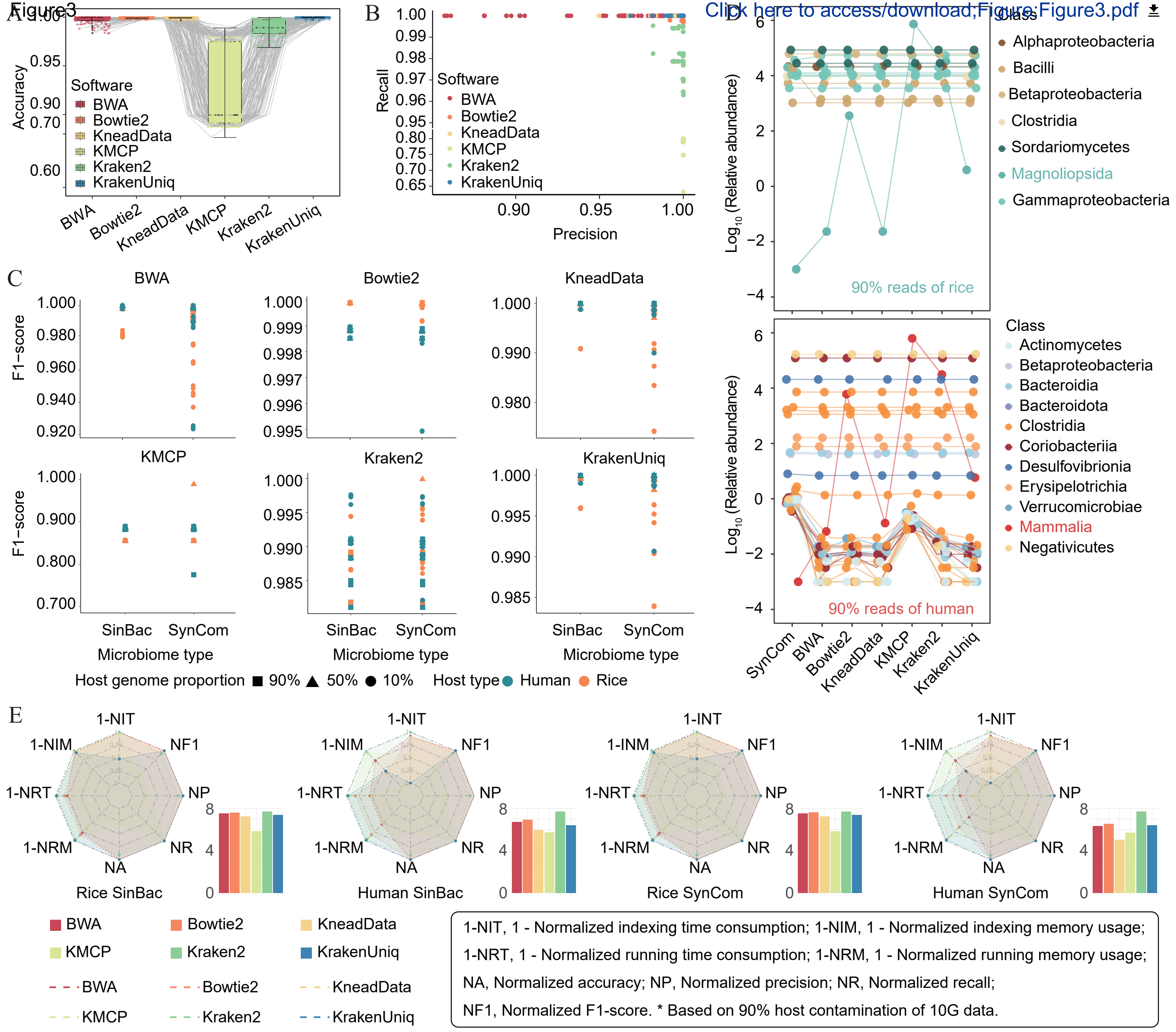

Figure4

Click here to access/download;Figure;Figure4.pdf

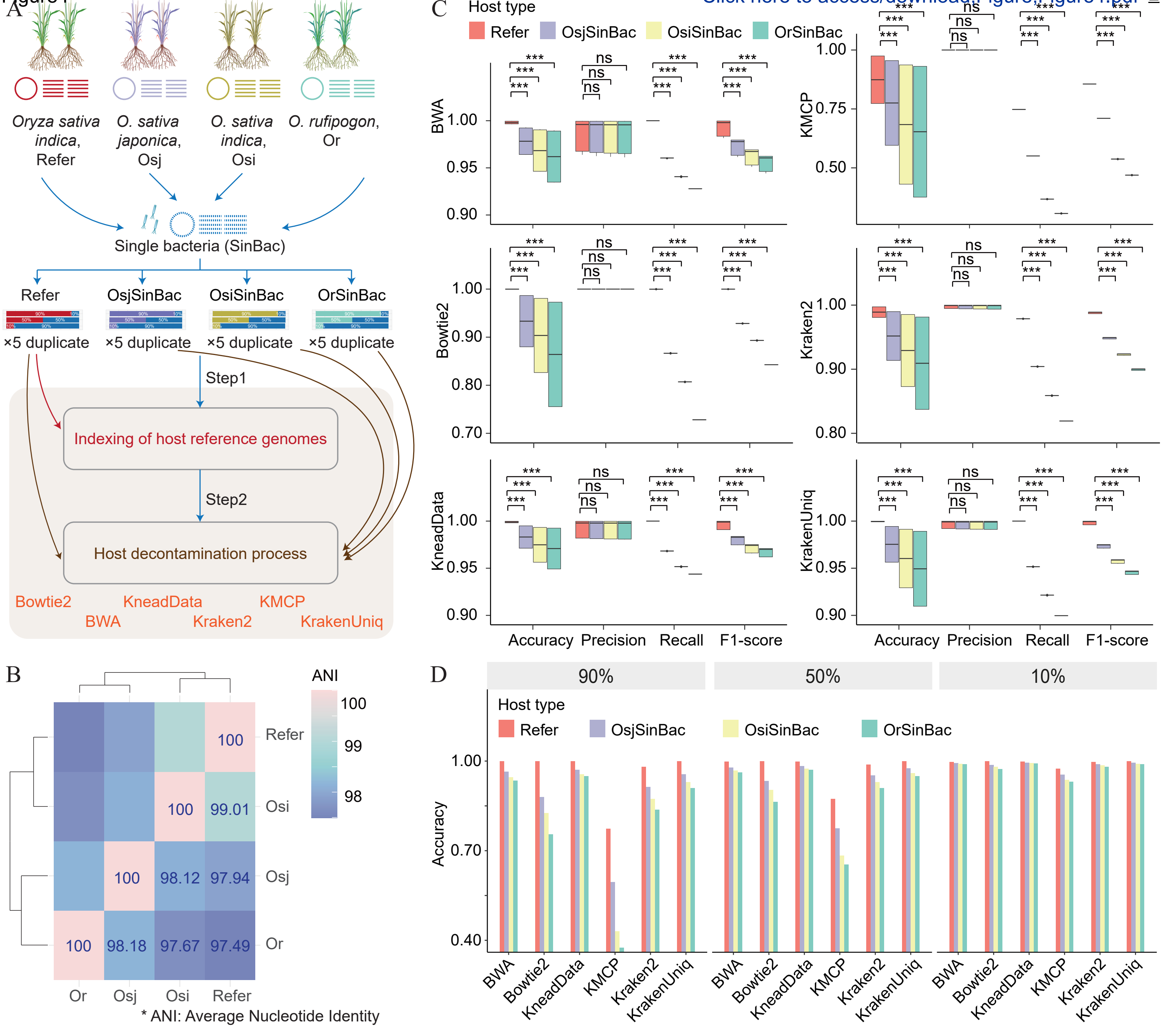

Supplementary tables

Click here to access/download
Supplementary Material
SupplymentaryTables.xlsx

FigureS1

Click here to access/download
Supplementary Material
FigureS1.pdf

FigureS2

Click here to access/download
Supplementary Material
FigureS2.pdf

FigureS3

Click here to access/download
Supplementary Material
FigureS3.pdf

FigureS4

Click here to access/download
Supplementary Material
FigureS4.pdf

FigureS5

Click here to access/download
Supplementary Material
FigureS5.pdf

FigureS6

Click here to access/download
Supplementary Material
FigureS6.pdf

GA

Click here to access/download;Graphical Abstract;GA.pdf

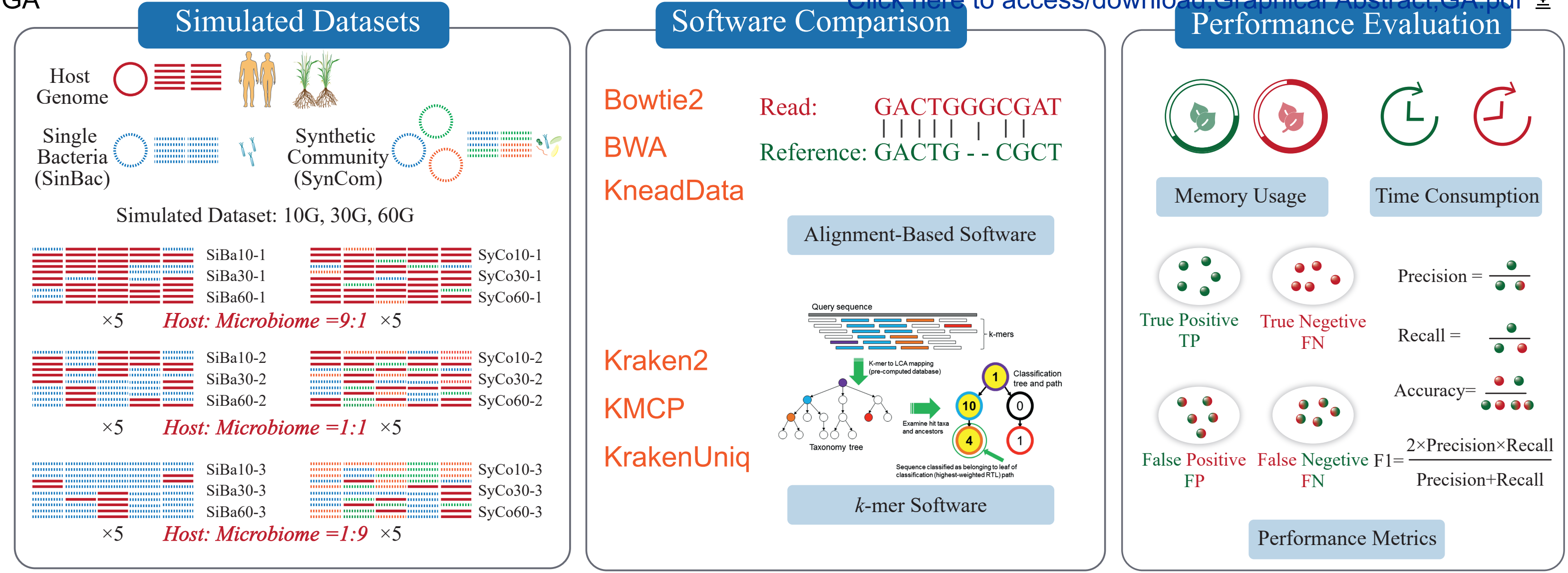

Supplement: giaf004_GIGA-D-24-00318_Original_Submission [file giaf004_giga-d-24-00318_original_submission.pdf]
