# Supplementary material for: Benchmarking short-read metagenomics tools for removing host contamination
Source: Gigascience. 2025 Feb 27;14:giaf004. doi: 10.1093/gigascience/giaf004 (PMC11878760; doi:10.1093/gigascience/giaf004)

GigaScience

Benchmarking short-read metagenomics tools for removing host contamination

--Manuscript Draft--

**Manuscript Number:**	GIGA-D-24-00318R1
**Full Title:**	Benchmarking short-read metagenomics tools for removing host contamination
**Article Type:**	Research
**Funding Information:**	China Postdoctoral Science Foundation (2024M753580) — Dr. Yunyun Gao<br>National Natural Science Foundation of China (U23A20148, 32470055) — Not applicable<br>Agricultural Science and Technology Innovation Program (CAAS-ZDRW202308) — Prof. Yongxin Liu
**Abstract:**	The rapid evolution of metagenomic sequencing technology offers remarkable opportunities to explore the intricate roles of microbiome in host health and disease, as well as to uncover the unknown structure and functions of microbial communities. However, the swift accumulation of metagenomic data poses substantial challenges for data analysis. Contamination from host DNA can substantially compromise result accuracy, and increase additional computational resources by including non-target sequences. In this study, we assessed the impact of computational host-DNA decontamination on downstream analyses, highlighting its importance in producing accurate results efficiently. We also evaluated the performance of conventional tools like KneadData, Bowtie2, BWA, KMCP, Kraken2, and KrakenUniq, each offering unique advantages for different applications. Furthermore, we highlighted the importance of an accurate host reference genome, noting that its absence negatively affected the decontamination performance across all tools. Our findings underscore the need for careful selection of decontamination tools and reference genomes to enhance the accuracy of metagenomic analyses.
**Corresponding Author:**	Yongxin Liu<br>Chinese Academy of Agricultural Sciences<br>CHINA
**Corresponding Author Secondary Information:**	
**Corresponding Author's Institution:**	Chinese Academy of Agricultural Sciences
**Corresponding Author's Secondary Institution:**	
**First Author:**	Yunyun Gao, Ph.D.
**First Author Secondary Information:**	
**Order of Authors:**	Yunyun Gao, Ph.D.<br>Hao Luo<br>Hujie Lyu<br>Haifei Yang<br>Salsabeel Yousuf<br>Shi Huang<br>Yongxin Liu
**Order of Authors Secondary Information:**	
**Response to Reviewers:**	Dear Editor,<br><br>We appreciate the thorough review and constructive feedback provided by you and the

Powered by Editorial Manager® and ProduXion Manager® from Aries Systems Corporation

reviewers. The suggestions have been instrumental in enhancing our manuscript. Here is a summary of the major suggestions and our responses:
1)Introduction & Methods:
We have expanded the introduction to include a brief description of the parameters analyzed and the rationale for selecting human and rice as hosts. Additionally, we have clarified the details regarding the synthetic community design and the simulations (Lines 300–317).
2)Discussion of Limitations:
We have added a section discussing the limitations of our analysis, especially the challenges of applying these tools to real-world data. We have also outlined future directions, including plans to incorporate real data simulations as suggested by the reviewer (Lines 414–422).
3)Terminology Standardization:
We have ensured consistent use of key terms such as Raw, Removed, Microbiome, and Host Removal across the entire manuscript. These changes aim to enhance clarity and avoid any potential confusion for the readers.
4)Research Title:
We have changed our title as ‘Benchmarking short-read metagenomics tools for removing host contamination’.
5)Figures Issues:
We thoroughly reviewed all figures and addressed issues such as adding simulation line functions and correcting typographical errors.
All the detailed answer are attached below, with our responses in blue. We believe these revisions address the reviewers’ concerns and significantly improve our manuscript. Your feedback has been invaluable, and we are committed to further refining our work.

Sincerely,

Yong-Xin Liu
Agricultural Genomics Institute at Shenzhen, Chinese Academy of Agricultural Sciences
liuyongxin@caas.cn
Google Scholar: https://scholar.google.com/citations?user=NoxNy_IAAAAJ

Authors response to Reviewers
Reviewer reports:
Reviewer #1: The authors present the importance of host decontamination for accurate metagenomic analyses and benchmarked six bioinformatics tools for the removal of host reads. The analyses presented is comprehensive and statistically rigorous. This would be a useful resource for other researchers in the field of metagenomics to guide selection of appropriate tools for host read removal. Nevertheless, the introduction of the paper is missing some foundational information that makes it difficult to follow. This includes a brief introduction of the parameters that are being analyzed and the strategy for designing the synthetic communities as well as the motivation of specifically looking at human and rice as host in this analysis. The methods are lacking clarity into how the simulations were carried out and the type of sequencing technology that is being simulated. The paper also lacks discussion on limitations of the analyses, its broader implication in real data and elaboration on the biological relevance of the analysis. How would real samples be reflected/represented by the different percent of host contaminated reads as presented in this analysis. ie is 90% typical when studying skin surfaces? Given artifacts derived from sequencing, the impact of this work can be benefited by including an analysis of real-world data - ie known species spike-ins into known host background that is subsequently sequenced and compared to expected abundances.
Response: Thank you very much for your detailed and thoughtful feedback. We greatly appreciate your positive comments regarding the comprehensiveness and statistical rigor of our analysis. We have carefully considered your suggestions and have made several revisions to enhance the clarity and depth of the manuscript.
1) For the missing foundational information, we have expanded them to include more essential background information. Specifically:
A brief description of the parameters used for synthetic community design has been added in the ‘Methods’ to improve clarity on the approach used. We also provide more

details on how the simulations were conducted, including descriptions of the SynCom and SinBac that were simulated, to clarify this aspect of the methods (Line 309-317). We have explained the rationale behind selecting human and rice as hosts in our analysis (Line 300-301). Human samples are emphasized due to their critical importance in medical research, especially in the context of the human microbiome. Rice, on the other hand, serves as a key model organism in agricultural research. Additionally, both species have well-annotated and complete genomes, which meet our computational needs.
2) For the discussion of limitations and broader implication, we have added the discussion the limitations of our analysis, including the potential challenges when applying these tools to ‘real-world data’ (Line 414-422). We have also elaborated on the broader implications of our findings for metagenomic research. For example, in microbiome studies, contamination levels can vary significantly, and high contamination percentages are not uncommon. We greatly appreciate your suggestion regarding the inclusion of real sequencing data performance. In future work, we plan to conduct real data simulations and incorporate new algorithms to develop host removal tools suited for various scenarios. Thank you again for your valuable suggestions.

Specific comments:
- Line 58 - Consider adding that sequencing resources could also be wasted in the process of requiring deeper sequencing to capture non-host reads.
Response: Thanks for your advice, here we have addressed this by emphasizing the waste of sequencing resources caused by sequencing unwanted host DNA reads and the additional need for deeper sequencing to accurately capture non-host reads.

- Line 85 - What does "genomes from 30 microbiota: refer to? 30 microbiome samples? Or 30 microbial species?
- What is the rationale for selection of 30 genomes/species? Was this based on a specific environment? Were the abundance of each species equal?
Response: Thank you for your valuable feedback. The phrase “genomes from 30 microbiota” refers to the genomes of 30 microbial species, as detailed in Table S1-2 (Microbiome Composition). We selected these species based on the reference “Cheng AG, Ho P-Y, Aranda-Díaz A, et al. Design, construction, and in vivo augmentation of a complex gut microbiome. Cell 2022; 19:3617-36”, where we randomly chose 30 common human-associated microbial species for simulation. Each species was represented by 0.1 million reads, resulting in a total of 3 million reads for the dataset, as described in the Methods section under “Simulated dataset description for the downstream analysis”.
To avoid any confusion for the readers, we have revised the wording in the relevant sections. Specifically, in the Results section, we changed “incorporating genomes from 30 microbiota and Homo sapiens (GRCh38)” to “incorporating microbial reads from 30 species, each represented at equal abundance, along with human reads from Homo sapiens (GRCh38)”. Additionally, in the Methods section, we provided the reference for the microbial community used in our simulations.

- Line 90 - what part of the metagenomic analysis? Does this refer to the classification task?
- More details can be provided here regarding the types of analyses that are being done downstream to evaluate efficacy of host decontamination, i.e. MAG assembly, functional annotation etc.
Response: Thank you for your insightful comments. We have revised the description of the metagenomic analysis to provide more details about the specific types of analyses performed to evaluate the efficacy of host decontamination, as well as clarifying what part of the analysis we are referring to.

- Line 102 - The different use of lower and uppercase to refer to the samples are confusing. Consistent use of the uppercase version "R"aw, "R"removed, "M"icrobiome would help with clarity.
Response: Thank you for your suggestion. We have standardized the terminology by consistently using uppercase for all sample references: ‘Raw’, ‘Removed’, and ‘Microbiome’.

- Line 102 - More context can be given here as to why the relative abundance is "altered". It is obvious that the presence of host reads would skew the relative

Powered by Editorial Manager® and ProduXion Manager® from Aries Systems Corporation

abundances. Would it be more informative to present the absolute counts?
Response: Thank you for your insightful suggestion. In Figure 1C, we observed that the Raw data impacted the relative abundance due to the presence of host reads. However, after applying Kraken2's decontamination (removing Chordata), the relative abundance results aligned with those annotated for the Microbiome and Removed datasets. This is consistent with the rationale presented in our introduction, where we discussed the use of Kraken2 for host decontamination.
However, due to limitations in the reference database, the annotation results may not fully match our simulated data. Although we used the Kraken2 PlusPFP database—the most comprehensive available at 150 GB (as mentioned in the Methods section under 'Metagenomic Analysis')—we opted not to directly compare the classification data with true data to avoid potential misinterpretation by readers. Instead, we addressed this limitation in the Discussion section.
Regarding your suggestion to present absolute abundances, we found that in the Raw data, the majority of reads were classified as Chordata, but after decontamination, the remaining taxa annotations were consistent with those in the Microbiome and Removed datasets (the Table 1 was attached below). After discussion among all co-authors, we decided to retain the current presentation of relative abundance results.
Table 1. Absolute counts of the top 10 phyla.
PhylumMicrobiome dataRaw dataRemove data
S1S2S3S1S2S3S1S2S3
Bacillota897000896623896190897001896623896190897000896623896190
Bacteroidota547070547173547005547070547173547006547070547173547005
Actinomycetota502823502948502800502823502948502800502823502948502800
Verrucomicrobiota100065100099100105100065100099100105100065100099100105
Thermodesulfobacteriota806678047180450806678047180450806678047180450
Pseudomonadota167471676116742168541676116777167471676116742
Streptophyta144281417014492144561425014586144281417014492
Campylobacterota164216421662164216421662164216421662
Chordata1343130313162700111227001115270009442828552879
Mycoplasmatota736775765736775765736775765
Others333732883246333932913248333732883246

- Line 104 - What does "released taxa" refer to? It seems that filtering out reads that align with Chordata achieved similar results as the Microbiome-only sample. If that is the case, what would the benefit of host decontamination be here?
Response: Thank you for your question. By "released taxa", we were referring to the microbial taxa that became visible after removing the Chordata (human) reads from the Raw data. This allowed us to better focus on the microbial community, resulting in similar outcomes to those observed in the Microbiome-only sample. To avoid any confusion, we have replaced "released taxa" with "remaining taxa" and added a reference to Figure 1C, de-chordata group.
Regarding the benefit of host decontamination, in the Raw data, Kraken2's species annotation included a large amount of host information (Chordata), which affected the accuracy of microbial community profiling. In contrast, the Remove data, where host reads were filtered out using KneadData, showed a microbial community composition more similar to the Microbiome data. Although Kraken2 can be used both for species annotation and host removal, this highlights the importance of using dedicated host decontamination tools like KneadData to improve species annotation accuracy. This is also why we further evaluate different tools in the next section of the manuscript. Additionally, due to limitations in the database of Kraken2, the annotated results differed from the actual simulated data, an issue we address in the Discussion section. We hope that by sharing our findings, we can contribute to the ongoing refinement of metagenomic analysis tools.

- Line 105 - What about the Shannon index results?
Response: Thank you for your question, and we are so sorry that we have included the Shannon index in Figure S2A, while not addressing the Shannon index results in our initial description. Our analysis showed a significant difference in the Shannon index across the Microbiome, Removed, and Raw data. The values for the Microbiome data were closely grouped (3.3397; 3.3390, 3.3402) and were higher than those for the Removed data (3.3425; 3.3419, 2.2431). In contrast, the Raw data had notably lower values (0.4549; 0.4549, 0.4565). This indicates that the removal of host reads improves the diversity estimates of the microbial community compared to the Raw

Powered by Editorial Manager® and ProduXion Manager® from Aries Systems Corporation

data. After discussion, we decided to focus solely on the Richness index in our presentation, as it provides a clearer representation of microbial diversity and avoids potential confusion.

- Line 108 - It seems to be unusual that the first axis of the PCoA explains 100% of variation. Is this expected?
Response: Thank you for your insightful question. We acknowledge that it may seem unusual for the first axis of the Principal Coordinates Analysis (PCoA) to explain 100% of the variation. This result typically indicates that the data has low dimensionality, which can occur when the microbial community composition is dominated by a few taxa or when there is minimal variation among the samples.
In our case, this observation suggests that the removal of host reads led to a clearer distinction between the sample groups, resulting in a single axis capturing all the variability. While this is not the most common scenario in more complex datasets, it highlights the significant impact of host decontamination on the observed microbial community structure. We appreciate your attention to this detail and will clarify this point in the manuscript to provide better context for our findings.

- Line 115 - There seems to be no further discussion about the recovery of B. breve from only the raw samples. Any hypotheses of why this could have been the case?
Response: Thank you for your question. To assess the recovery of B. breve, we compared the MAGs obtained from our analysis with the simulated Bifidobacterium breve data (GCF_001025175.1). The ANI similarity between the MAGs and the reference genome ranged from 99.8% to 99.9%. In our manuscript, we used MetaWRAP refinement to improve the quality of our MAGs binning, applying the parameters -c 50 -x 10, meaning we retained only bins with completeness over 50% and contamination below 10% (Table 2, MAGs in blue).
However, when we kept all binning data and adjusted the MetaWRAP refinement parameters to -c 0 -x 100, we indeed recovered additional low-quality MAGs (Table 2, MAGs in green). With this adjustment, B. breve was enriched in a greater number of Microbiome and Remove data, displaying a completeness of 85.42% to 85.89% and contamination of 12.91% to 14.46%. Additionally, we examined all enriched data and found no clear patterns, except that host removal generally improves the recovery of high- and medium-quality MAGs, though some specific groups may still be enriched in raw data.
To provide readers with more information, we have added our MAG filtering parameters to the Materials and Methods section and included this discussion in the discussion section.
Table2. Reconstruetd MAGs of B. breve.
SamplesfastANIcompletenesscontaminationGCN50sizeMAG quality
Microbiome199.4585.4213.60.587157881898963Low
Microbiome299.802494.818.1560.587185892083715Medium
Microbiome399.687685.8912.910.588187661967196Low
Remove199.410285.4214.460.587154441973302Low
Remove299.816894.818.1560.587185892083715Medium
Remove399.688285.8912.910.588187661965172Low
Raw199.890983.181.1090.588165121695982Medium
Raw299.891193.285.660.589192782050916Medium
Raw399.84390.342.7070.589190701910714High
* fastANI represents the comparison between reconstructed MAGs of different samples and reference genome (Bifidobacterium breve fasta data (GCF_001025175.1)).
High MAGs, completeness ≥ 90% and Contamination ≤ 5%; Medium MAGs, completeness ≥ 50% and Contamination < 10%; Low MAGs, completeness < 50% or Contamination > 10%

- Line 118 - In what aspect does “higher similarity” refer to here? What are the numbers in the x and y axis of Fig. 1E?
Response: Thank you for your insightful question. The term ‘higher similarity’ refers to the Spearman correlation between the GO terms in different datasets. Specifically, we compared the GO term annotations between Microbiome data, Raw data, and Removed data. In Figure 1E, the x-axis represents the GO term counts in the Microbiome data, while the y-axis represents the GO term counts in the Raw and Removed data, respectively. To make this clearer, we have now added linear regression lines to the plot for better visualization of the relationship. And we have

Powered by Editorial Manager® and ProduXion Manager® from Aries Systems Corporation

replaced the phrase ‘higher similarity’ with ‘stronger correlation’ in our manuscript to avoid any potential confusion.

- Line 124 - how many species are included in the SynCom and were the communities designed to reflect either a human-associated microbiome and a rice microbiome? This is later mentioned in the methods but stating in here could help with interpretability. Furthermore, what is the rationale for simulating equal abundances of each species?
Response: We apologize for not providing sufficient details regarding the SynCom. In our study, the SynCom included 14 species for the rice microbiome and 35 species for the human-associated microbiome. The simulated data were based on core microbiota reported in the literature (see Table S2-1). For further clarification, we have included a detailed description in the Methods section and noted in Line 124 that readers can find additional information there.
Regarding the rationale for simulating equal abundances of each species, we initially employed the differential mode from the CAMISIM software, which captures a greater degree of randomness and reflects natural samples more closely. However, in the first part of our results, we chose to use equal abundances to emphasize the impact of host decontamination on the recoverable microbial MAGs. In other parts of the manuscript, we utilized a differential approach that better represents natural sample dynamics.

- Line 130 - The description of the abbreviations are somewhat confusing. Is SinBac 60-3 supposed to be SynBac 60-3 here? In Fig. 2A the bar graphs in the center showing the proportions of host vs bacteria reads does not seem to be consistent with the vertical bar graphs in the same plot.
Response: Thank you so much for pointing out this issue. Here, it should be ‘SynCom 60-3’ instead of ‘SinBac 60-3’. We have reviewed the entire manuscript for consistency and made the necessary corrections. Additionally, we have updated Figure 2A to ensure that the proportions of host versus bacterial reads in the bar graphs are consistent with the vertical bar graphs in the same plot.

- Line 182 - What were some of the false positives/ false negatives? Were these specific species that are consistently misclassified? What taxonomic levels were the analysis caried out at? Do these metrics differ at different taxonomic levels?
Response: Thank you for your question. In our study, we evaluated the effectiveness of host read removal using various software tools. True positives refer to host reads that were correctly identified and removed as host genome sequences. False positives represent microbiota reads that were mistakenly classified as host reads and thus removed, while false negatives refer to host reads that were not identified and remained in the dataset.
Our analysis was conducted at the read level, as each simulated metagenomic read was labeled with its specific origin. Therefore, we did not focus on identifying consistently misclassified species at different taxonomic levels, since the classification was based on individual reads rather than specific taxa. However, the potential for misclassification of certain reads is an area of interest, and in future work, we plan to conduct a more detailed analysis of these misclassified reads to better understand patterns in distinguishing between host and microbial sequences more accurately.

- Lines 195-198 - If KrakenUniq also displays low Mammalia values, shouldn't it be considered as being able to remove more host contamination reads rather than less?
Response: Thank you for pointing this out. We appreciate your careful observation. What we intended to express here is that, among the three tools, BWA and KneadData demonstrate better host read removal performance compared to KrakenUniq. We have revised the original text to clarify this point.

- Line 212 - How does different versions of references genomes impact host removal? (ie GRCh38, telomere-to-telomere gapless sequence of the human genome). Can a similar evaluation be carried out to assess the impacts of these versions?
Response: Thanks so much for your suggestion, we acknowledge that different versions of reference genomes may have varying impacts on host removal efficiency. In our future work, we plan to evaluate these differences.

- Line 231 - Does "the absence of a host reference genome" mean absence of a host reference genome that is closely aligned with the host whereby samples/sequences were derived from? If yes, this might have to be reworded for clarity.

- It is not always possible to obtain host genomes that are specific to a given sample type - are there recommendations for what can be done to select a suitable host genome?
Response: We appreciate your suggestion. We have revised our wording to clarify that 'the absence of a host reference genome' now reads as 'the absence of a closely aligned and accurate reference genome'. Additionally, we have thoroughly reviewed and updated the relevant descriptions throughout the manuscript. Regarding the selection of a suitable host genome, our experience suggests that using a pangenome approach during database construction can be effective. We plan to validate this approach in our upcoming research. Additionally, we are exploring the integration of deep learning algorithms to address the challenges posed by the absence of precise reference genomes.

- Line 245 - How were the simulations carried out in CAMISIM? What were the simulation parameters?
Response: Thank you for your question. We have expanded the Methods section to provide a clearer understanding of how the simulations were conducted in CAMISIM. This includes a detailed description of the simulation parameters used, ensuring readers can better grasp how we obtained the simulated data.

- Lines 299-304 - These text appears to be repeated from the results section above (line 122-127).
Response: Thank you for pointing this out. Our initial intention was to maintain consistency to avoid confusion for readers. We have now streamlined the description in lines 122-127 to allow readers to focus more on the results section.

Please correct all spelling mistakes in the text, figures and figure legends.
For example:
- Figure 1A 0.1million reads/species, Diversity analysis, Memory usage
- Line 367 - 27 million "reads"
Line 369 - "equal"
Response: Thank you so much for your thorough review. We have carefully checked and revised our entire manuscript.

Reviewer #2: Overall Impressions
The authors present a nice comparison of different methods for removing host data from metagenomic sequencing data. As this focuses entirely on short-read metagenomic data, I suggest altering the title to communicate this.
Response: Thank you for your suggestion. We have changed our title as 'Benchmarking short-read metagenomics tools for removing host contamination'.

Major Issues
Can the authors comment on whether a two stage-approach would be beneficial for data from hosts with a good-quality reference genome (eg. human or mouse). I have noticed in my own data that, after decontamination by Bowtie2 alignment against the human reference genome, some reads are still classified by Kraken2 as Homo sapiens - which I then remove. Would implementing this as standard be beneficial or offer only a marginal increase in data quality in return for the additional processing time and resources. The authors highlight that Kraken2 seems to be suitable for identifying which reads are from Homo sapiens (L104, Fig 1C) - they could use this info to add another decontamination method to the evaluation (Kraken2 classification of Raw data followed by extracting non-chordata reads using the extract_kraken_reads.py script in KrakenTools).
Response: Thank you for your insightful question. In metagenomic studies, we often work with data from numerous samples, while accurate reference genomes for each host (human) are hardly available. Thus, we typically construct a reference index based on a publicly available host genome and perform host removal analyses using tools like Bowtie2 or KneadData. And it is also common for Kraken2 to annotate additional host reads. Based on our team's experience, we also directly remove these extra host reads use the extract_kraken_reads.py script.
The issue of residual host contamination due to the lack of an accurate reference genome is evident in our data. For example, when using Bowtie2 with a precise host

reference genome, we achieve data accuracies ranging from 0.9973 to 0.9999 (Figure 3A). In contrast, when a precise reference genome is absent, the accuracy of our data drops to a range of 0.7552 to 0.9999 (Figure 4C).
Regarding the potential benefits of a two-step approach for host contamination removal, we strongly support this method. Host contamination can significantly affect the accuracy of downstream functional annotations (Figure 1E). In our original manuscript, we discussed various host removal strategies that balance speed and accuracy, and we developed a workflow called HostPurge (https://github.com/HaoLuo-leo/HostPurge). In our future work, we will further optimize the HostPurge workflow to provide a precise and efficient solution for host contamination removal.

Why was the number of reads chosen for the microbial species included? The authors focus on the reconstruction of MAGs and note that some species were not recovered in some groups - it is likely that increasing the number of reads overall, without adjusting the % of reads from microbiome and host, will impact this.
Response: Thank you for your question. In our study, we aimed to assess the impact of host reads on the generation of Metagenome-Assembled Genomes (MAGs) from microbial communities, particularly in samples with high host proportions (such as 90%). Typically, metagenomic datasets are around 10 Gb in size. If we were to increase the number of reads from the microbial species, the overall sequencing coverage would also need to be increased, which would significantly raise the costs associated with sequencing and computational analysis.
Our focus was to investigate the effects of high host read proportions on the recoverability of MAGs under standard sequencing conditions. In our simulations, the 30 million reads generated approximately 8 Gb of data, which aligns with the typical sequencing output in routine studies. This design allows us to explore the dynamics of host contamination in a realistic context while keeping the analysis feasible.

Were the MAGs that were not recovered from species with larger genomes? The same number of reads from a larger genome will likely return a smaller MAGs due to a lower breath and depth of coverage. Also, can the authors state why MAGs were reconstructed in the first place? Was it for data quality purposes?
Response: Thank you for your question. The quality of the data is indeed crucial for recovering high-quality MAGs, which serve as a foundation for further investigation into the functionality, metabolism, and evolution of specific species or strains.
Reconstructing MAGs is a vital step in metagenomic data analysis, as it enables functional validation of microbial species or strains identified in the sample.
In our study, each microbial species was represented by 0.1 million reads, with genome sizes ranging from approximately 2 to 6 Mb (Table S1-2, S2-1). This resulted in a coverage depth of 5~15× for each genome. To determine whether insufficient coverage might explain the failure to reconstruct certain MAGs, we checked the genomes of reconstructed and unreconstructed MAGs. Here two reconstructed MAGs did not match our reference data, but we did not observe a strong correlation between genome size and the ability to recover MAGs (Table 1).
In this work, we used MetaWRAP refinement to improve the binning of our MAGs with parameters -c 50 -x 10, meaning we retained only those bins with completeness greater than 50% and contamination less than 10%. However, when we retained all binning data and adjusted the MetaWRAP refinement parameters to -c 0 -x 100, we indeed recovered additional low-quality MAGs, while we still did not see the strong correlation
We hypothesize that, in addition to genome size, factors such as the heterogeneity of the simulated data and the presence of specific bacterial genes might influence both the quantity or quality of the MAGs that can be recovered.
Table 1. The genome size of simulated microbiota.
SpeciesGenome_SizeReconstructed MAGs this workAdjusted parameter (-c 0 -x 100)
Veillonella_dispar2.1MbNoNo
Absiella_dolichum2.2MbNoNo
Ruminococcus_bromii2.2MbNoNo
Acidaminococcus_fermentans2.3MbYesYes
Anaerofustis_stercorihominis2.3MbYesYes
Collinsella_aerofaciens2.3MbNoYes
Bifidobacterium_breve2.3MbYesYes
Bifidobacterium_pseudocatenulatum2.3MbYesYes
Holdemanella_biformis2.5MbNoYes

Powered by Editorial Manager® and ProduXion Manager® from Aries Systems Corporation

	Burkholderiales_bacterium2.6MbNoNo<br>Akkermansia_muciniphila2.7 MbYesYes<br>Desulfovibrio_piger2.9MbNoNo<br>Adlercreutzia_equolifaciens2.9MbNoNo<br>Ethanoligenens_harbinense3.0MbYesYes<br>Slackia_heliotrinireducens3.1MbYesYes<br>Clostridiales_bacterium3.2MbNoNo<br>Anaerobutyricum_hallii3.3MbNoNo<br>Eubacterium_rectale3.4 MbNoNo<br>Clostridium_methylpentosum3.5MbNoNo<br>Eggerthella_lenta3.6MbYesYes<br>Holdemania_filiformis3.6MbNoNo<br>Alistipes_finegoldii3.7MbYesYes<br>Clostridium_hylemonae3.8MbNoNo<br>Roseburia_inulinivorans4.2MbNoNo<br>Bacteroides_coprocola4.3MbNoNo<br>Odoribacter_splanchnicus4.4MbYesYes<br>Lacrimispora_saccharolytica4.6MbYesYes<br>Parabacteroides_distasonis4.7 MbYesYes<br>Phocaeicola_vulgatus5.2 MbNoNo<br>Bacteroides_xylanisolvens6.0MbNoNo<br><br>Kneaddata uses Bowtie2 to do the alignments, what is the difference between the Bwotie2 approach presented here and the alignment step performed by Bowtie2 in Kneaddata?<br>Response: Thank you for your question. In our software evaluation, we used the default or recommended parameters for each tool. While both Bowtie2 and KneadData use the Bowtie2 alignment algorithm, there are differences in how they handle host contamination removal.<br>For example, KneadData is an integrated pipeline that automates various preprocessing steps for metagenomic data, including quality control (such as trimming low-quality reads) and decontamination (aligning reads to a host genome). In our evaluation, we used the --bypass-trim and --bypass-trf options to skip these preprocessing steps, but our results showed that the outcomes of KneadData and Bowtie2 were not completely identical. Upon further investigation, we found that the default parameters differ between the two: Bowtie2 uses the --sensitive-local setting [-D 15 -R 2 -N 0 -L 22 -i S,1,1.15], while KneadData applies the --very-sensitive-local setting [-D 20 -R 3 -N 0 -L 20 -i S,1,0.5]. This could be one of the reasons for the observed differences. Therefore, we still presented their differences in our work to highlight their variation.<br>As we have already discussed the parameter settings for different software in the background section, we do not delve into a detailed comparison of parameters in the main body of the manuscript.<br><br>Minor Issues<br>Throughout: the authors switch between the terms "host-depleted" and "Remove" to refer to the data which has been decontaminated. I suggest sticking to one, the former is more descriptive and tends to work better in context, but it is up to the authors.<br>Response: Thank you for your suggestion. Apart from the section where we compare Raw data, Remove data, and Microbiome data, we have standardized the term ‘host-removal’ throughout the rest of the manuscript to ensure consistency and improve readability for the readers.<br><br>L49: I realise this is not the aim of the manuscript, but could the authors briefly mention the different methods that exist for microbial DNA enrichment (or host DNA depletion) before library preparation and sequencing.<br>Response: We appreciate your suggestion. A postdoc of our team, who specializes in this area, has written a review on microbial DNA enrichment and host DNA depletion methods during experiments. This review is currently being prepared for submission and we hope it will provide valuable insights to the field on this topic.<br><br>L51-52: I agree that this is a concern. Can the authors provide a reference to any manuscripts which have evaluated this? If they exist. It would be important context for the reader and might also address my previous point.

Powered by Editorial Manager® and ProduXion Manager® from Aries Systems Corporation

	Response: Thank you for your insightful question. As mentioned earlier, the review will also address this concern, summarizing the different methods and their evaluations, which can provide important context for the reader.<br><br>L63: can the authors hypothesise about a particular reason for the increase began in 2015 - is this due to a paper or commentary highlighting the importance? Or is this an artefact of the increased quantity of microbiome papers published over this period?<br>Response: Thank you for your question. It's hard to pinpoint a specific reason for the increase beginning in 2015. Our survey indicates that the number of research articles employing metagenomic analysis (excluding amplicon studies) gradually increased from 2012 to 2014. However, we cannot definitively say whether this rise correlates with specific events, such as the release of Kraken in 2014.<br>Moreover, in our opinion, it's possible that authors may have employed these steps but chose not to include details in their descriptions. While we apologize for not being able to provide more specific information, it is clear that an increasing number of articles are beginning to recognize the importance of detailing host removal procedures.<br><br>L130: I think the description is incorrect here. The even split would be SinBac 60-2, while the 90% host and 10% community would be SynCom 60-1?<br>Response: Thank you so much for pointing out this issue. Here, it should be 'SynCom 60-3' instead of 'SinBac 60-3'. We have reviewed the entire manuscript for consistency and made the necessary corrections.<br><br>L182- L188 - can the authors please comment on a) whether there were specific microbial species, genes, or sets thereof which were commonly erroneously identified as host, and b) were these erroneously unmapped reads subsequently assigned taxonomy by Kraken2. These are important considerations as they may impact the downstream microbiome profile.<br>Response: Thank you very much for your insightful question. Indeed, we encountered instances in our study where some reads were detected by Kraken2 after being filtered for host contamination using Bowtie2 or KneadData. We believe this discrepancy arises from the differences between alignment-based algorithms and k-mer algorithms, as well as our inability to provide precise host reference genomes.<br>As for whether specific microbial species, genes, or sets of reads were commonly misidentified as host, we do not have definitive conclusions at this time. However, identifying these specific data types will be a key focus of our future work. We appreciate your valuable suggestions.<br><br>Discussion: generally, journals prefer that you do not refer to Figures and Tables in the discussion section. They should already be introduced in the results and methods section. Check that this is allowed by Gigascience.<br>Response: Thank you for your advice. We checked the formatting guidelines of GigaScience, and while we did not find specific requirements regarding the referencing of figures and tables in the discussion section, we all agree that your suggestion is reasonable. Therefore, we have removed the relevant references from the discussion. Thank you for your helpful feedback.<br><br>L369: "equal"<br>Response: We appreciate your feedback and have made the necessary changes.<br><br>L387-388: This could be tested by including reference genomes from other genera in the same family, if available.<br>Response: Thank you very much for your suggestion. We plan to implement this work in the future development of the HostPurge pipeline https://github.com/HaoLuo-leo/HostPurge. In our upcoming research, we will investigate whether our software performs better with reference genomes from the same genera or family, or even in cases where no reference genomes are available.
**Additional Information:**	
**Question**	**Response**
Are you submitting this manuscript to a special series or article collection?	No

**Experimental design and statistics**<br><br>Full details of the experimental design and statistical methods used should be given in the Methods section, as detailed in our Minimum Standards Reporting Checklist. Information essential to interpreting the data presented should be made available in the figure legends.<br><br>Have you included all the information requested in your manuscript?	Yes
**Resources**<br><br>A description of all resources used, including antibodies, cell lines, animals and software tools, with enough information to allow them to be uniquely identified, should be included in the Methods section. Authors are strongly encouraged to cite Research Resource Identifiers (RRIDs) for antibodies, model organisms and tools, where possible.<br><br>Have you included the information requested as detailed in our Minimum Standards Reporting Checklist?	Yes
**Availability of data and materials**<br><br>All datasets and code on which the conclusions of the paper rely must be either included in your submission or deposited in publicly available repositories (where available and ethically appropriate), referencing such data using a unique identifier in the references and in the "Availability of Data and Materials" section of your manuscript.<br><br>Have you have met the above requirement as detailed in our Minimum Standards Reporting Checklist?	Yes

Powered by Editorial Manager® and ProduXion Manager® from Aries Systems Corporation

Manuscript Click here to access/download;Manuscript;Manuscript.docx

Benchmarking short-read metagenomics tools for removing host contamination

Yunyun Gao[1*], Hao Luo[1*], Hujie Lyu[2], Haifei Yang[1,3], Salsabeel Yousuf[1], Shi Huang[4], Yong-Xin Liu[1#]

[1]Shenzhen Branch, Guangdong Laboratory of Lingnan Modern Agriculture, Genome Analysis Laboratory of the Ministry of Agriculture and Rural Affairs, Agricultural Genomics Institute at Shenzhen, Chinese Academy of Agricultural Sciences, Shenzhen 518120, China

[2]Department of Life Sciences, Imperial College of London, London SW7 2AZ, UK

[3]College of Life Sciences, Qingdao Agricultural University, Qingdao 266000, China

[4]Faculty of Dentistry, The University of Hong Kong, Hong Kong SAR, China

[#] Correspondence: liuyongxin@caas.cn (Yong-Xin Liu)

* These two authors contributed equally to this work.

ORCIDs

Yunyun Gao: https://orcid.org/0000-0003-3389-9385

Hao Luo: https://orcid.org/0009-0005-3391-8576

Yong-Xin Liu: https://orcid.org/0000-0003-1832-9835

Abstract

The rapid evolution of metagenomic sequencing technology offers remarkable opportunities to explore the intricate roles of microbiome in host health and disease, as well as to uncover the unknown structure and functions of microbial communities. However, the swift accumulation of metagenomic data poses substantial challenges for data analysis. Contamination from host DNA can substantially compromise result accuracy, and increase additional computational resources by including non-target sequences. In this study, we assessed the impact of computational host-DNA decontamination on downstream analyses, highlighting its importance in producing accurate results efficiently. We also evaluated the performance of conventional tools like KneadData, Bowtie2, BWA, KMCP, Kraken2, and KrakenUniq, each offering unique advantages for different applications. Furthermore, we highlighted the importance of an accurate host reference genome, noting that its absence negatively affected the decontamination performance across all tools. Our findings underscore the need for careful selection of decontamination tools and reference genomes to enhance the accuracy of metagenomic analyses.

Keywords

Metagenome, microbiome, host removal, microbial enrichment

Background

The advancement of second-generation sequencing technology and data analysis methods has greatly facilitated microbiome research, broadening our horizons in the widespread influences of microbiome on their host [1-3]. Compared to amplicon sequencing, shotgun metagenomic sequencing offers comprehensive assessments of bacterial communities with less bias, and improved resolution in identifying profiles at the species, strain, or functional levels [4]. As sequencing technology costs rapidly decreased and sequencing depth continues to expand, the volume of metagenomic data is growing exponentially [5]. Some studies even producing over 100 giga base pairs (Gbps) per sample to characterize the dark matter of the microbiome in human gut [6]. However, a major challenge exists when analyzing the metagenome data from complex host-associated microbiomes, such as those found in saliva, throat, and vaginal swabs. These samples often, contain over 90% human-aligned reads [4, 7, 8] due to the high contamination of host DNA. This contamination undermines the characterization of microbiomes, especially for low abundant species [7], leading to biased observations of the true underlying microbial composition. Additionally, privacy concerns have become particularly significant when the host is human [9], highlighting the importance of removing host contamination.

Despite efforts to remove host contamination during the experimental stage [8, 10-13], particularly in DNA isolation, residual DNA remains polluted with numerous host DNA fragments. The overall efficacy of different experimental protocols varies, and potential biases for the preferential enrichment of specific microbial taxa remain a concern. Even after significant host DNA reduction, high biomass samples like mucosal microbiome can still exhibit up to 90% host contamination in metagenomic data [8, 10]. This persistence is due to differences in cell and genome size between animals and microbiomes. Similarly, in low biomass samples like endophytic microorganisms, around 70% of host sequencing reads may still be present despite efforts to collect and concentrate the microbiota [14]. This high level of contamination often necessitates deeper sequencing to adequately capture the microbial reads of interest. Sequencing these unwanted host DNA reads, followed by computational removal from large NGS datasets, is both wasteful and time-consuming [15]. It compromises the accuracy of downstream analyses and consumes valuable research time and computing resources. This underscores the importance of devising a host contamination removing tool in the data analysis stage that is both accurate and efficient.

After searching 2,853 publications using the keywords ‘metagenome’ and ‘microbiome’ (Figure S1A, Table S1), we found that 57.94% of the studies addressed the removal of host contamination, with a discernible increasing trend from 2015 to 2024. The absence of standardized criteria for selecting host decontamination software has led to the use of 51 different tools, many relying on alignment and *k*-mer strategies. Among these, ten tools exhibited notable popularity, generally employ two main strategies: alignment-based and the *k*-mer approaches [9, 16, 17]. The alignment software, such as Bowtie2 [18], BWA [19], aligning sequencing reads to reference genomes. And

Kraken2 [20], KMCP [21] are popular *k*-mer based software that identify exact matches between small substrings (*k*-mers) from the reads in the reference database. Besides, some host contamination removal pipelines integrate these modules. For instance, DeconSeq [22] integrates a modified version of BWA, while KneadData (http://huttenhower.sph.harvard.edu/kneaddata) integrates Bowtie2. Several new tools, like Hostile [9], HoCoRT [16] have also been developed to enhance the accuracy of host decontamination process. Although some studies have explore the impact of varying amounts of host DNA on microbiomes [23, 24], the impact of removing host DNA contamination on the bioinformatic downstream analysis and the microbial genome assembly remains unclear [12].

In this study, we will compare the efficiency of metagenomic sequencing on microbiome by removing host contamination, and thoroughly evaluate the accuracy and speed of state-of-the-arts in computational host DNA decontamination. These results will serve as a guide for researchers in rationally selecting suitable tools for processing various metagenomic datasets.

Results

High host contamination increased the processing time and skewed interpretation the microbiome results

Here we simulated three groups (S1, S2, S3) of data using CAMISIM with 90% host contamination (Table S1-2), incorporating microbial reads from 30 species, each represented at equal abundance, along with human reads from *Homo sapiens* (GRCh38). We used KneadData, a popular host decontamination software in recent years, to remove host contamination from the raw data (Raw), resulting in host-removed data (Remove), while the 30 microbial groups served as a negative control (Microbiome). Our metagenomic analysis involved key steps such as species composition, diversity analysis, functional analysis, and metagenome-assembled genome (MAG) evaluations (Figure 1A). Based on these simulations, we evaluated the impact of host contamination removal on metagenomic analysis of the microbiome (Figure 1A), focusing on memory usage, processing time, and the effects on the accuracy of the results.

Throughout the analysis, no significant differences in memory usage were observed among the Raw data, Removed data, and Microbiome data during high-memory steps exceeding 100 gigabytes (GBs), such as species-level taxonomic annotation (Kraken2), de-replication (drep), and MAG annotation (GTDBtk). However, compared to the Raw data, the host-read-removed data significantly reduced the run time of downstream analyses (Figure 1B). Specifically, processing the host removal data took 5.98 times shorter for binning (MetaWRAP), 7.63 times shorter for function annotation (HUMAnN3), and 20.55 times shorter for assembly (MEGAHIT). And the average processing time for Remove data was 139.14 minutes (min) for MetaWRAP compared to 832.64 min for Raw data, 308.92 min for HUMAnN3 compared to 2357.95 min for Raw data, and 106.59 min for MEGAHIT compared to 2190.27 min for Raw data. Additionally, handling the negative control data (Microbiome) required similar resources to the host removal data in terms of both

memory usage and time consumption.

Compared to Microbiome data, Raw data altered the relative abundance of microbiota community, while Remove data showed a similar composition to that annotated by Kraken2 (Figure 1C). Interestingly, the remaining taxa were similar to the Microbiome data, after removing the Chordata (the phylum of *Homo sapiens*) from the Raw data (Figure 1C, de-chordata group). There was no difference in richness index between Microbiome data and Remove data, whereas Raw data showed a significantly lower richness index than both (Figure S2A). And principal coordinates analysis (PCoA) was performed to visualize changes in community composition, revealing that the first axes of PCoA explained 100% of the overall variations. This observation suggested low dimensionality and distinct separation of sample groups. Specifically, samples from Raw data were clearly separated from those of Microbiome and the Removed data along PCo1 (Figure S2B). This finding underscores the effectiveness of host decontamination in highlighting the underlying microbial community structure.

Despite simulating Metagenomic data from 30 microbiota, with each species having 0.1 million reads, only 14 metagenome-assembled genomes (MAGs) were obtained. No significant differences were detected in completeness rate and contamination rates among the Microbiome data, Raw data and Removed data (Figure S2C). However, the number of MAGs was much more in Microbiome and Removed data compared to Raw data, except for *Bifidobacterium breve*, which was detected in all three groups (S1, S2, S3) in Raw data, but only in S2 in Microbiome and Removed data (Figure 1D). Next, we compared the gene ontology (GO) terms to Microbiome data. We found a stronger correlation in GO terms between Removed data and Microbiome data than that between Raw data and Microbiome data (Figure 1E, Figure S2D), indicating that the host removal process results in more specific gene function annotation.

Kraken2 was fast and low-resource tool for host removal.

To further compare the differences in host removal among various software tools, we obtained 1080 simulated metagenomic datasets, which include single bacterium (SinBac) and synthetic community (SynCom) across various sizes (10 Gbps, 30 Gbps, and 60 Gbps). The simulations were conducted separately for human (*Homo sapiens*) and rice (*Oryza sativa indica*) hosts, each with 90%, 50%, and 10% levels of host contamination (Figure 2A, see Methods for more details). For convenience, we have assigned abbreviations to various datasets. For example, SinBac10-1 refers to a 10 Gbps dataset with 90% host genome reads and 10% reads from a single bacterium genome. Similarly, SynCom 30-2, represented a 30Gbps dataset with 50% host genome reads and 50% reads from the synthetic community genome. SynCom 60-3, refers to a 60Gbps dataset with 10% host genome reads and 90% reads from the synthetic community genome (Figure 2A). Based on these simulated data, we compared the computational resources required and host decontamination performance of six existing tools: KneadData, Bowtie2, and BWA (for alignment-based software), and KMCP, Kraken2, and KrakenUniq (for *k*-mer strategy software).

Before removing host contamination, indexing of host reference genomes is crucial. In this study, we constructed reference genomes for *Homo sapiens* (GRCh38) and *Oryza sativa indica* (GWHBFPX00000000) with sizes approximately 3.1 Gbps and 373.8 Megabase pairs (Mbps), respectively. Kraken2 utilized minimal computational resources for indexing both human and rice genomes, requiring only 0.3 gigabyte (Gb) memory and taking 6.94 minutes (min) to create a custom database for the human genome (Figure 2B). In contrast, the other five tools required an average of 18.05 Gb memory and 117.98 min.

We then compared the resource consumption during the host contamination removal process across six software. In summary, Bowtie2 (1.95 Gb (0.410, 3.42)), and Kraken2 (2.47 Gb (0.710, 4.12)) demonstrated the lowest maximum memory usage across all simulated datasets for alignment and *k*-mer based software, respectively (Figure 2C, Figure S3). These values were significantly lower than that of the other four tools, with BWA requiring 3.995 Gb (1.40, 6.74), KneadData requiring 15.17 Gb (6.47, 30.27), KMCP requiring 14.45 Gb (4.27, 25.110), KrakenUniq requiring 22.410 Gb (11.13, 33.67). Regarding data size, handling 60 Gbps data consumed significantly more resources than 10 Gbps data in KneadData and Kraken2. Different host types also significantly influenced resource consumption across all software (Table S3-1), with human data requiring notably more resources than rice data ($P < 0.05$). Whereas, no significant difference between the different microbiome types for all software, except for KrakenUniq (Table S3-1).

For time usage, the *k*-mer software (KMCP, 156.10 min (90.47, 231.16), Kraken2, 29.34 min (13.42, 55.45), and KrakenUniq, 59.23 min (26.66, 98.14), required less time than the alignment-based software (BWA 582.26 min (300.15, 1065.64), Bowtie2 209.00 min (111.66, 512.61), KneadData 501.38 mins (287.41, 1177.06)), with Kraken2 demonstrating significantly shorter execution times compared to other tools (Figure 2E, Table S3-2). The diversity of microbiomes exhibited no impact on processing time across all six software (Table S3-2). But, the size of the metagenomic data significantly influenced execution time (Figure 2D, 2E), this emphasized the importance of utilizing fast software for efficient processing, especially when dealing with large metagenomic datasets. Simulated metagenomic data from human took more time to process than data from rice across all tools, indicating that host genome complexity leads to increased processing time (Figure S4, Table S3). Noticeably, a high proportion of host genome contamination significantly reduced the speed of alignment-based tools like BWA, Bowtie2, KneadData, and KrakenUniq (Figure S4, Table S3-2). For instance, processing a large 60 Gbps metagenomic dataset containing 90% human genome contamination resulted in a significant increase in processing time (1.35-fold in KrakenUniq, 2.59-fold in BWA, 5.36-fold in KneadData, and 6.76-fold in Bowtie2) compared to the same dataset with only 10% contamination. This suggested that these tools may be less suitable for datasets with substantial host contamination. Nevertheless, this significant slowdown was not observed in KMCP and Kraken2.

Performance in host decontamination accuracy of six software

Then four metrics (accuracy, recall, precision, and F1-score) were calculated to evaluate the performance of host decontamination accuracy in six software based on with the 1080 simulated data generated according to the rule in Figure 2A. We observed significant differences among the six software ($P < 0.05$) in the accuracy, recall, precision, and F1-score (Table S3-4, S3-5, S3-6, S3-7). In terms of accuracy, the alignment-based software (BWA, 0.9989 (0.9966, 0.9998), Bowtie2, 0.9997 (0.9988, 0.9998), and KneadData, 0.9997 (0.9989, 0.9998)) outperformed the *k*-mer software (KMCP, 0.8947 (0.8133, 0.9748); Kraken2, 0.9891 (0.9832, 0.9974)), with the exception of KrakenUniq (0.9998 (0.9994, 0.9999)), which consistently exhibited a high and stable performance (Figure 3A). However, the alignment-based software exhibited lower precision performance (BWA, 0.9980 (0.9853, 0.9996), Bowtie2, 0.9999 (0.9999, 0.9999), and KneadData, 0.9981 (0.9971, 0.9998)), potentially leading to an increased number of false positives associated with the host genome. This implied that some microbiome reads may be erroneously mapped as part of the host genome and subsequently was removed as contamination. Conversely, *k*-mer software (KMCP, 0.7686 (0.7477, 0.7925), Kraken2 0.9787 (0.9787, 0.9823), and KrakenUniq 0.9999 (0.9999, 1)) showed lower recall performance, leading to an increased number of false negatives associated with the host genome. This suggested that some host reads may be erroneously unmapped, thereby retaining some host contamination in the downstream analyses (Figure 3B). For F1-score (2 * Precision * Recall/ (Precision + Recall)), the type of microbiome, host type, and the proportion of host genome all influenced the performance on these tools. Notably, BWA, KneadData, and KrakenUniq performed significantly better on human datasets compared to rice datasets. Conversely, Bowtie2 showed great performance with rice dataset (Figure 3C).

We compared the composition of the metagenomic dataset with a synthetic community (SynCom) after host decontamination using the abovementioned six software (Figure 2A). The classes Magnoliopsida and Mammalia displayed low values (i.e., the $\log_{10}$-transformed relative abundance) in BWA, KneadData, and KrakenUniq (Figure 3D). Among these, BWA and KneadData, both alignment-based tools, demonstrate superior performance in removing host contamination compared to KrakenUniq, which, as a *k*-mer based tool, tends to be less effective. All tools, except for KMCP, identified some few groups, which belongs to Actinomycetes, Clostridia, Negativicutes, as host contamination, and removed them from the raw data (Figure 3D). For the integrated comparison of resource consumption and the performance of host decontamination across software, we normalized all data using min-max normalization. This enables a comparative analysis of computational efficiency and host contamination removal effectiveness across simulated datasets (Figure 3E, Figure S5). Based on the summarized normalized data, Kraken2 showed significant excellence ($P < 0.05$) under the high levels (90%) of host contamination, in both human (7.8093 (7.7236, 7.8215)) and rice (7.8268 (7.7109, 7.8330)) datasets through comprehensive comparisons. Additionally, when comparing the host removal performance among alignment-based software,

focusing on normalized accuracy (NA), normalized precision (NP), normalized recall (NR), and normalized F1-score (NF1) under the high levels (90%) of host contamination, KneadData demonstrated significant superiority ($P < 0.05$) in human (3.9996 (3.9996, 3.9997)).

The absence of accurate host reference genome affected the decontamination performance

Next, we assessed the impact of lacking a host reference genome on the effectiveness of existing host decontamination software. Three *Oryza* species—*Oryza sativa japonica* (GWHBFOO00000000, Osj), *Oryza sativa indica* (GWHBFPT00000000, Osi), and *Oryza rufipogon* (GWHBFHN00000000, Or)—were selected as the resource of host metagenomic reads to generate simulated datasets. *Oryza sativa indica* (GWHBFPX00000000, Refer) was chosen as the reference genome (Figure 4A). The average nucleotide identity (ANI) between the three species (Osj, Osi, Or) and the reference genome, showed that Osi had the highest similarity (99.01%) to the reference genome, followed by Osj at 97.94% and Or at 97.49% (Figure 4B). The simulated metagenomic reads derived from a single bacterium (SinBac) was generated as before, resulting in three datasets (OsjSinBac, OsiSinBac, OrSinBac) contained varying levels of host DNA contamination (10%, 50%, and 90%) and each 10 Gbps in size. Subsquently, the indexing databases for the six tools were built using the reference genome, and we compared the performance of host decontamination tools (BWA, Bowtie2, KneadData, KMCP, Kraken2 and KrakenUniq) under these conditions (Figure 4A).

In terms of time and memory consumption, KneadData and KrakenUniq used more memory (Figure S6A), while alignment software took more time than *k*-mer software (Figure S6B), as previously described. When comparing with reference metagenomic data, processing OsjSinBac, OsiSinBac, OrSinBac data resulted in all software requiring significantly more time (Figure S6B). Notably, the absence of a closely aligned and accurate host reference genome negatively impacted the decontamination performance of all tools (Figure 4C). Specifically, accuracy, recall, and F1-score were significantly lower for datasets representing OsjSinBac, OsiSinBac, and OrSinBac compared to reference data aligned to the indexing database created with the reference genome. Precision, however, did not show significant differences, indicating that decontaminated datasets still contained some residual host reads.

Then we compared the comprehensive performance for all software during the absence of accurate host reference genome. Based on the summarized normalized data of resource consumption during running and host decontamination metrics (accuracy, precision, recall, and F1-score). All software performed better with reference metagenomic data (5.41 (5.15, 5.91)) than with OsjSinBac (4.93 (4.32, 5.50)), OsiSinBac (4.54 (3.91, 5.35)), OrSinBac (4.64 (4.08, 5.19)) datasets, whereas less difference across these datasets was observed with Kraken2 and KrakenUniq (Figure S6C). Moreover, while different tools performed better on high-host contamination samples with a reference genome, this advantage was not evident in the absence of a reference genome. In the presence of 90% host contamination, all tools showed a significant reduction in accuracy (Figure

4D, Table S4-1), emphasizing the importance of a host reference genome for high contamination metagenomic data.

Methods

Literature searches and data collection

A literature search was conducted in the Web of Science Core Collection Database on April 18th, 2024, using the search terms ‘metagenome’ and ‘microbiome’ (Table S1-1). Only research articles were utilized to gather information on software usage (Figure S1A). We excluded publications solely focused on amplicon sequencing data, long-read metagenomes, environmental samples or food samples. Subsequently, we compiled the percentage of publications mentioning host contamination removing and the number of publications for each software.

Simulated dataset description for the downstream analysis

Three groups of data (S1, S2, S3) with 90% host contamination were simulated, including genomes from 30 microbiota species and *Homo sapiens* (GRCh38). The 30 microbial species were randomly selected based on a previously published human-associated microbial community [25]. Here we used CAMISIM to generate metagenomic data for 30 microbial genomes, simulating paired-end reads (PE150) with three replicates for each microbial species (number_of_samples = 3). Each dataset comprised 3 million microbial reads, with 0.1 million reads per microbial species, combined with 27 million reads from Homo sapiens. The reads were labeled accordingly before mixing (Table S1-2). Among the microbiome species, six belonged to Actinomycetota, 15 to Bacillota, six to Bacteroidota, and one each to Pseudomonadota, Thermodesulfobacteriota, and Verrucomicrobiota. To remove host contamination from the raw data (Raw), we used KneadData, a bioinformatics tool specifically designed for this purpose. The output from KneadData processing constituted the host removal data (Remove). And the 30 microbial groups served as a negative control (Microbiome) to evaluate the accuracy and effectiveness of the host contamination removal process.

Metagenomic analysis

In order to compare the difference of analysis in direct data (Raw data) and the host contamination removing data (Remove data), we selected 10 Gbps synthetic community datasets of human with varying host contamination levels (Figure 1A). The resource consumption was tested as following description, and metagenomic analysis directly refer to the steps of EasyMetagenome 1.10 pipeline [26]. Briefly, taxonomic profiling was performed using Kraken2 [27], with PlusPFP database, and relative abundances were obtained using Bracken. Functional profiling was preformed via HUMAnN3 [28] using Uniref90 gene families. After assembling the metagenomic data using Megahit 1.0. [29], metagenomic binning and bin refinement were conducted using MetaWRAP [30]. The MetaWRAP refinement aimed to enhance the quality of our MAG binning, utilizing the parameters -c 50 -x 10, which retained only bins with completeness greater than 50% and contamination less than 10%. Redundancies in the metagenome-assembled genomes (MAGs) were removed with dRep v2.6.2. MAGs were annotated using GTDBtk v2.3.2 [31], and their quality was

evaluated using CheckM2 v1.0.1 [32]. Gene prediction was performed using Prodigal v2.6.3 [33], clustering of genes with CD-HIT v4.8.1 [34], quantification of genes with salmon v1.8.0, and gene annotation with emapper v2.1.6. Then alpha diversity and beta diversity analyses was analyzed using R 4.2.3 as described in EasyAmplicon [35]. The completeness and contamination rate of metagenome assembly genomes (MAGs) were normal measurement data, thus they were presented as median (*P25*, *P75*). And we also annotated the GO terms with eggnog-mapper, and calculated their correlation with microbiome data, that only retained microbiome from raw data, using Spearman. The pipeline could be found in https://github.com/YunyunGao374/HostPurge/blob/main/0HostDecontaminationImpactiononDownstreamAnalysis.sh.

Simulated dataset description for the comparison of six tools using human and rice data

We selected human and rice, both of significant economic and medical importance, and with well-characterized genomes, as the focus of our study. Six tools were selected for analysis, three of which are alignment-based software (BWA, Bowtie2, and KneadData), while the others are *k*-mer based (KMCP, Kraken2, KrakenUniq). Simulated datasets were generated using CAMISIM, and analyses were conducted using default or author-recommended parameters. To ensure comparability and reliability, each dataset comprised five replicates. These datasets covered various data sizes (10 Gbps, 30 Gbps, 60 Gbps), different level of host DNA contamination (90%, 50%, 10%), and diverse microbial complexities (single bacterium, SinBac, or synthetic community, SynCom) from both human and rice samples (Figure 2A). Each dataset had five replicates per condition. For the rice SynCom, we selected 14 commonly reported species, chosen randomly from known rice-associated microbes. The human SynCom was constructed with 35 species (Table S2-1), based on a previously published human-associated microbial community [25]. For the SinBac simulations, we utilized default parameters in CAMISIM to generate paired-end reads (PE150) for both the host and a single bacterial genome, conducting five replicates for each simulation (number_of_samples = 5). We then mixed reads from the host and microbial genomes in varying proportions of host DNA contamination. In the case of the SynCom simulations, we employed the same ways as in the SinBac for generating the host's metagenomic data. However, for the microbial metagenomic data, we used the differential mode in CAIMISM. The resulting data were then mixed according to different level of host DNA contamination. The code for the generation of simulated data can be found in https://github.com/YunyunGao374/HostPurge/blob/main/1HostDecontaminationSoftwareComparison.sh. Taxonomy information and their genome IDs are provided in Table S2-1.

To ensure comprehensive evaluation, we generated 1080 simulated datasets using CAMISIM. These datasets encompass three distinct sizes (10 Gbps, 30 Gbps, and 60 Gbps) and representing both simple (SinBac) and complex (SynCom) microbiomes. The simulations were conducted separately for human (*Homo sapiens*, GRCh38) and rice (*Oryza sativa indica*, GWHBFPX00000000) hosts, each with three levels of host contamination (10%, 50%, and 90%), enabling a nuanced exploration

of host genome contamination removal across various conditions (Figure 1A). For the species which contains multiple chromosomes, we just download all of them and stimulated the information, and all of reference and fasta information have been attached in Table S2-1. Here, for convenience, we have assigned abbreviations to various datasets. For example, SinBac10-1 denoted a 10 Gbps dataset with 90% of reads originating from the host genome and 10% from single bacteria genome. Similarly, SynCom 30-2, represented a 30 Gbps dataset with an even split of 50% reads from the host genome and 50% from the synthetic community genome. Based on these simulated data, we assessed the impact of metagenomic sequencing on microbiome by removing host contamination.

Simulated dataset description for the comparison of six tools within a genus level

To assess the performance of host decontamination tools at the genus level, we utilized three rice species and simple microbiomes separately: *Oryza sativa japonica* (GWHBFOO00000000, Osj), and *Oryza sativa indica* (GWHBFPT00000000, Osi), *Oryza rufipogon* (GWHBFHN00000000, Or). For each species, we generated datasets by combining the host genome with a simple bacterial genome, resulting in three separate datasets: Or with simple bacteria (OrSinBac), Osj with simple bacteria (OsjSinBac), and Osi with simple bacteria (OsiSinBac). Each dataset was simulated using CAMISIM with different levels of host DNA contamination (10%, 50%, and 90%) for 10 Gbps datasets, with five replicates per condition.

Oryza sativa indica served as the reference genome (GWHBFPX00000000, Refer), and indices were created for six tools (BWA, Bowtie2, KneadData, KMCP, Kraken2, KrakenUniq), as described above. Detailed fasta information is provided in Table S2-1. These simulated datasets were analyzed to compare the performance of the six tools in removing host contamination within the genus. The comparison involved evaluating computational resources and the effectiveness of host decontamination. Genome similarity was calculated using fastANI v1.34 [36], and the impact of metagenomic sequencing on microbiome have been assessed with the tested of following performance metrics. Based on these datasets, we aimed to determine how each tool performs in removing host contamination when genome data is limited within the same genus.

Performance metrics tests

All software utilized eight threads for building the database and running the processes across simulated datasets, and the running code of different software can be found at https://github.com/YunyunGao374/HostPurge/blob/main/1HostDecontaminationSoftwareComparison.sh. We assessed a comparative analysis of resource consumption, focusing on maximum RAM usage and processing time. Additionally, we evaluated their performance metrics including true positive (TP), true negative (TN), false positive (FP), and false negative (FN). And the precision, recall and F1-score were also calculated, with the following formulas: Accuracy = (TP + TN) / (TP + FN + FP + TN), Precision = (TP / (TP + FP)), Recall = (TP / (TP + FN)), F1-score = 2 * Precision * Recall / (Precision + Recall) [37]. Then all bioinformatics analysis were all analyzed within R 4.2.3. We conducted normality and homogeneity tests on all data. Measurement data were expressed

as mean ±SE for normally distributed data and as median (*P25, P75*) for non-normally distributed data. For normally distributed data with homogeneous variances in two-group comparisons, we used paired t-tests. Non-normally distributed or non-homogeneous data were analyzed using non-parametric Wilcoxon tests. In multiple group comparisons, normally distributed data with homogeneous variances were analyzed with ANOVA, while non-normally distributed or non-homogeneous data were analyzed with non-parametric Kruskal-Wallis tests [38]. Bonferroni post-hoc tests were performed on the data within each group to analyze the differences between different datasets [39]. For the average species composition plot, when dealing with data containing zeros, we adopted the straightforward method of adding a positive constant (0.001) to all leaf trait values [40], and then taking the logarithm of the resulting average relative abundance. For the integrated comparison of resource consumption and the performance of host purge in each software, we normalized all data use min-max normalization [41], which is a technique that performs a linear transformation of the original data. Data visualization was done using ggplot2 package, and $P \leqslant 0.05$ was regarded as statistically significant. All relevant data and plot code can be found at https://github.com/YunyunGao374/HostPurge.

Discussion

While 57.94% of publications eliminated host contamination before analyzing their data, while the remaining opted to analyze their data directly, potentially sacrificing valuable microbiome information in the process. Some software was developed to decontaminate the host genome [9, 18-22], but limited research provided the distinctions among them. For accelerating the reproducibility, comparability, and standardization of metagenomic data, it is important to understand how these different tools, broadly referred to downstream analyses [11]. And the significant influence of function annotation, species annotation, reads assembly and binning in the consumption of computational resources, exists the necessity to remove host contamination, particularly in ultra-high-depth sequencing data.

In our study, we generated a simulated dataset with 0.1 million reads of 30 microbiome species, and 27 million reads of *Homo sapiens*. Among the microbiome species, six belonged to Actinomycetota, 15 to Bacillota, six to Bacteroidota, one each to Pseudomonadota, Thermodesulfobacteriota, and Verrucomicrobiota. However, the proportion of the relative abundance were not equal, and two phyla not included in the simulation (Streptophyta and Campylobacterota) were annotated. This discrepancy may be attributed to the limitations of the Kraken2 annotation process, despite our use of the most comprehensive database (PlusPFP), as well as the the heterogeneity of our simulated data. In addition, although each of simulated data comprises 30 microbial groups, we only obtained 14 MAGs. The strict bin refinement parameters (-c 50, -x 10) may have further contributed to this outcome, highlighting the potential need for further improvement of the metagenomic analysis software. Nonetheless, the importance of the host decontamination was still being demonstrated in terms of gene functional annotation. Meanwhile, the significant difference in the Shannon index

between microbiome (negative control) data and raw or removed data highlights the importance of establishing a ‘gold standard’ for host removal.

It is noteworthy that we observed significantly higher accuracy, precision, and F1-score in datasets with high (90%) compared to low (10%) levels of host contamination in KneadData. Similar trends were also observed in other alignment software. This phenomenon may be attributed to two factors. Firstly, our simulated data, derived from the host genome used as the indexing reference database, resulted in a higher proportion of host reads for high host contamination data (90%) aligning to the host reference in alignment software (BWA, Bowtie2, KneadData). Secondly, reads from the microbiome may be challenging to distinguish from reference genomes, leading to a higher number of microbiome reads being discarded in datasets with lower host contamination (10%).

Additionally, the challenge of predicting the host genome when it’s unavailable, potentially down to the genus, family, or order level, needs consideration. Interestingly, despite Osi having a closer ANI to the reference genome compared to Osj, the decontamination performance for OsiSinBac was not as effective as for OsjSinBac. And the high host contamination showed a significant reduction of accuracy in all software, emphasizing the importance of a host reference genome for high contamination metagenomic data [42]. The absence of an accurate host reference genome can lead to residual host sequences, which in turn reduces the precision of subsequent functional annotations. One possible solution might be the combination of alignment-based and *k*-mer methods for more accurate host contamination removal. However, a unique challenge remains in distinguishing microbial sequences resulting from horizontal gene transfer (HGT) rather than host contamination. HGT between the microbiome and host genomes, often involving mobile genetic elements (MGEs), plays a crucial role in microbial adaptation to diverse environments. Metagenomic sequencing, particularly with short-read technologies, faces significant difficulties in accurately identifying these horizontally transferred gene regions [43]. In the future, advances in artificial intelligence algorithms [44] and long-read sequencing may help overcome not only host contamination but also the problem of horizontal transfer of bacteria.

Conclusion

In conclusion, host decontamination not only speeds up downstream analysis but also enhances the accuracy of gene function annotation, particularly in ultra-high-depth sequencing data. And each of these tools (BWA, Bowtie2, KneadData, Krakne2, KMCP and KrakenUniq) offers unique strengths that can be harnessed based on the specific requirements of a research study. Briefly, Bowtie2 and KneadData provides more accurate removal capabilities, albeit with increased computational demands. Kraken2 and KrakenUniq offer fast and user-friendly solution, while KMCP can retain more low-abundance taxa. When reference genomes are lacking, BWA and KneadData are less impacted among alignment software, and Kraken2 and KrakenUniq are less affected among *k*-mer software.

Understanding the trade-offs between speed, accuracy, and computational resources is crucial for

selecting the most suitable tool for host DNA removal in metagenomic analyses. As research increasingly focuses on understanding the impact of host contamination on microbiome annotation, particularly for low abundance taxa [42], this study provides a comprehensive evaluation that lays the groundwork for refining tools and methodologies. Ultimately, these advancements will empower researchers to derive meaningful biological insights from complex metagenomic datasets.

Acknowledgments

The work was financially supported by the China Postdoctoral Science Foundation (2024M753580), National Natural Science Foundation of China (U23A20148, 32470055), and the Agricultural Science and Technology Innovation Program (CAAS-ZDRW202308). We would like to acknowledge the support of the Prof. Wei Shen (Institute for Viral Hepatitis, The Second Affiliated Hospital, Chongqing Medical University, China) for any help during running the KMCP. And we sincerely thank Dr. Yao Wang (Agricultural Genomics Institute at Shenzhen, Chinese Academy of Agricultural Sciences, China) for her help in data analyses, Dr. Kai Peng (Yangzhou University, China) for his help in drawing the spider diagram.

Authors' contributions

Yong-Xin Liu, Shi Huang and Yunyun Gao conceived and coordinated the study. Yunyun Gao, Hao Luo, and Haifei Yang collected and analyzed the simulated data. Yunyun Gao and Hujie Lyu did the literature searches and data collection. Yong-Xin Liu, Shi Huang, Salsabeel Yousuf and Yunyun Gao revised the manuscript. All authors have read the final manuscript and approved it for publication.

Conflict of interest

The authors declare that they have no conflicts of interest.

Data availability

The raw data of simulated metagenomic sequencing reads have been deposited at the Genome Warehouse (GWH) (https://bigd.big.ac.cn/gwh/) under PRJCA028271. All pipelines, data analyses, and plotting code can be found at https://github.com/YunyunGao374/HostPurge.

References

1. Rooks MG and Garrett WS. Gut microbiota, metabolites and host immunity. *Nature reviews immunology* 2016;6:341-52. https://doi.org/10.1038/nri.2016.42.

2. Gao Y, Li D and Liu Y-X. Microbiome research outlook: past, present, and future. *Protein & Cell* 2023;10:709-12. https://doi.org/10.1093/procel/pwad031.

3. Xia H, Zhang Z, Luo C, et al. MultiPrime: A reliable and efficient tool for targeted next‐generation sequencing. *iMeta* 2023;e143:1-16. https://doi.org/10.1002/imt2.143.

4. Saheb Kashaf S, Almeida A, Segre JA, et al. Recovering prokaryotic genomes from host-associated, short-read shotgun metagenomic sequencing data. *Nature protocols* 2021;5:2520-41. https://doi.org/10.1038/s41596-021-00508-2.

5. Tremblay J, Schreiber L and Greer CW. High-resolution shotgun metagenomics: the more data, the better? *Briefings in Bioinformatics* 2022;6:bbac443. https://doi.org/10.1093/bib/bbac443.

6. Jin H, Quan K, He Q, et al. A high-quality genome compendium of the human gut microbiome of Inner Mongolians. *Nature Microbiology* 2023;1:150-61. https://doi.org/10.1038/s41564-022-01270-1.

7. Pereira-Marques J, Hout A, Ferreira RM, et al. Impact of host DNA and sequencing depth on the taxonomic resolution of whole metagenome sequencing for microbiome analysis. *Frontiers in microbiology* 2019:1277. https://doi.org/10.3389/fmicb.2019.01277.
8. Wu-Woods NJ, Barlow JT, Trigodet F, et al. Microbial-enrichment method enables high-throughput metagenomic characterization from host-rich samples. *Nature Methods* 2023;11:1672-82. https://doi.org/10.1038/s41592-023-02025-4.
9. Constantinides B, Hunt M and Crook DW. Hostile: accurate decontamination of microbial host sequences. *Bioinformatics* 2023;12:btad728. https://doi.org/10.1093/bioinformatics/btad728.
10. Heravi FS, Zakrzewski M, Vickery K, et al. Host DNA depletion efficiency of microbiome DNA enrichment methods in infected tissue samples. *Journal of microbiological methods* 2020:105856. https://doi.org/10.1016/j.mimet.2020.105856.
11. Marchukov D, Li J, Juillerat P, et al. Benchmarking microbial DNA enrichment protocols from human intestinal biopsies. *Frontiers in genetics* 2023:1184473. https://doi.org/10.3389/fgene.2023.1184473.
12. Cheng WY, Liu W-X, Ding Y, et al. High sensitivity of shotgun metagenomic sequencing in colon tissue biopsy by host DNA depletion. *Genomics, Proteomics & Bioinformatics* 2022; https://doi.org/10.1016/j.gpb.2022.09.003.
13. Feehery GR, Yigit E, Oyola SO, et al. A method for selectively enriching microbial DNA from contaminating vertebrate host DNA. *PloS one* 2013;10:e76096. https://doi.org/10.1371/journal.pone.0076096.
14. Soto-Giron MJ, Kim J-N, Schott E, et al. The edible plant microbiome represents a diverse genetic reservoir with functional potential in the human host. *Scientific Reports* 2021;1:24017. https://doi.org/10.1038/s41598-021-03334-4.
15. Diao Z, Han D, Zhang R, et al. Metagenomics next-generation sequencing tests take the stage in the diagnosis of lower respiratory tract infections. *Journal of advanced research* 2022:201-12.
16. Rumbavicius I, Rounge TB and Rognes T. HoCoRT: host contamination removal tool. *BMC bioinformatics* 2023;1:371. https://doi.org/10.1186/s12859-023-05492-w.
17. Liu Y, Ghaffari MH, Ma T, et al. Impact of database choice and confidence score on the performance of taxonomic classification using Kraken2. *aBIOTECH* 2024:1-11. https://doi.org/10.1007/s42994-024-00178-0.
18. Langmead B and Salzberg SL. Fast gapped-read alignment with Bowtie 2. *Nature methods* 2012;4:357-9. https://doi.org/10.1038/nmeth.1923.
19. Li H and Durbin R. Fast and accurate short read alignment with Burrows–Wheeler transform. *bioinformatics* 2009;14:1754-60. https://doi.org/10.1093/bioinformatics/btp324.
20. Lu J, Rincon N, Wood DE, et al. Metagenome analysis using the Kraken software suite. *Nature protocols* 2022;12:2815-39. https://doi.org/10.1038/s41596-022-00738-y.
21. Shen W, Xiang H, Huang T, et al. KMCP: accurate metagenomic profiling of both prokaryotic and viral populations by pseudo-mapping. *Bioinformatics* 2023;1:btac845. https://doi.org/10.1093/bioinformatics/btac845.
22. Schmieder R and Edwards R. Fast identification and removal of sequence contamination from genomic and metagenomic datasets. *PloS one* 2011;3:e17288. https://doi.org/10.1371/journal.pone.0017288.

23. Nearing JT, Comeau AM and Langille MG. Identifying biases and their potential solutions in human microbiome studies. *Microbiome* 2021;1:113. https://doi.org/10.1186/s40168-021-01059-0.

24. McArdle AJ and Kaforou M. Sensitivity of shotgun metagenomics to host DNA: abundance estimates depend on bioinformatic tools and contamination is the main issue. *Access microbiology* 2020;4:e000104. https://doi.org/10.1099/acmi.0.000104.

25. Cheng AG, Ho P-Y, Aranda-Díaz A, et al. Design, construction, and in vivo augmentation of a complex gut microbiome. *Cell* 2022;19:3617-36. https://doi.org/10.1016/j.cell.2022.08.003.

26. Liu Y-X, Qin Y, Chen T, et al. A practical guide to amplicon and metagenomic analysis of microbiome data. *Protein & cell* 2021;5:315-30. https://doi.org/10.1007/s13238-020-00724-8.

27. Wood DE, Lu J and Langmead B. Improved metagenomic analysis with Kraken 2. *Genome biology* 2019:1-13. https://doi.org/10.1186/s13059-019-1891-0.

28. Abubucker S, Segata N, Goll J, et al. Metabolic reconstruction for metagenomic data and its application to the human microbiome. *PLoS computational biology* 2012;6:e1002358. https://doi.org/10.1371/journal.pcbi.1002358.

29. Li D, Liu C-M, Luo R, et al. MEGAHIT: an ultra-fast single-node solution for large and complex metagenomics assembly via succinct de Bruijn graph. *Bioinformatics* 2015;10:1674-6. https://doi.org/10.1093/bioinformatics/btv033.

30. Uritskiy GV, DiRuggiero J and Taylor J. MetaWRAP—a flexible pipeline for genome-resolved metagenomic data analysis. *Microbiome* 2018:1-13. https://doi.org/10.1186/s40168-018-0541-1.

31. Chaumeil P-A, Mussig AJ, Hugenholtz P, et al. GTDB-Tk v2: memory friendly classification with the genome taxonomy database. *Bioinformatics* 2022;23:5315-6. https://doi.org/10.1093/bioinformatics/btac672.

32. Chklovski A, Parks DH, Woodcroft BJ, et al. CheckM2: a rapid, scalable and accurate tool for assessing microbial genome quality using machine learning. *Nature Methods* 2023;8:1203-12. https://doi.org/10.1038/s41592-023-01940-w.

33. Hyatt D, Chen G-L, LoCascio PF, et al. Prodigal: prokaryotic gene recognition and translation initiation site identification. *BMC bioinformatics* 2010:1-11. 10.1186/1471-2105-11-119.

34. Fu L, Niu B, Zhu Z, et al. CD-HIT: accelerated for clustering the next-generation sequencing data. *Bioinformatics* 2012;23:3150-2. https://doi.org/10.1093/bioinformatics/bts565.

35. Liu YX, Chen L, Ma T, et al. EasyAmplicon: An easy‐to‐use, open‐source, reproducible, and community‐based pipeline for amplicon data analysis in microbiome research. *iMeta* 2023;1:e83. https://doi.org/10.1002/imt2.83.

36. Jain C, Rodriguez-R LM, Phillippy AM, et al. High throughput ANI analysis of 90K prokaryotic genomes reveals clear species boundaries. *Nature communications* 2018;1:5114. https://doi.org/10.1038/s41467-018-07641-9.

37. Qin H, Ou L, Gao J, et al. DENA: training an authentic neural network model using Nanopore sequencing data of Arabidopsis transcripts for detection and quantification of N 6-methyladenosine on RNA. *Genome Biology* 2022;1:25. https://doi.org/10.1186/s13059-021-02598-3.

38. McKight PE and Najab J. Kruskal‐wallis test. *The corsini encyclopedia of psychology* 2010:1-. https://doi.org/10.1002/9780470479216.corpsy0491.

39. Duque M, Lee-Kubli CA, Tufail Y, et al. Sonogenetic control of mammalian cells using exogenous Transient Receptor Potential A1 channels. *Nature communications* 2022;1:600.

https://doi.org/10.1038/s41467-022-28205-y.

40. Harris T, Ottaviani G, Mulligan M, et al. Trait hypervolumes based on natural history collections can detect ecological strategies that are distinct to biogeographic regions. *Journal of Ecology* 2023;2:314-26. https://doi.org/10.1111/1365-2745.14005.

41. Song Q, Lee J, Akter S, et al. Prediction of condition-specific regulatory genes using machine learning. *Nucleic Acids Research* 2020;11:e62-e. https://doi.org/10.1093/nar/gkaa264.

42. Gihawi A, Ge Y, Lu J, et al. Major data analysis errors invalidate cancer microbiome findings. *MBio* 2023;5:e01607-23. https://doi.org/10.1128/mbio.01607-23.

43. Brito IL. Examining horizontal gene transfer in microbial communities. *Nature Reviews Microbiology* 2021;7:442-53. https://doi.org/10.1038/s41579-021-00534-7.

44. Gao Y, Peng K, Bai D, et al. The Microbiome Protocols eBook initiative: Building a bridge to microbiome research. *iMeta* 2024;e182:1-7. https://doi.org/10.1002/imt2.182.

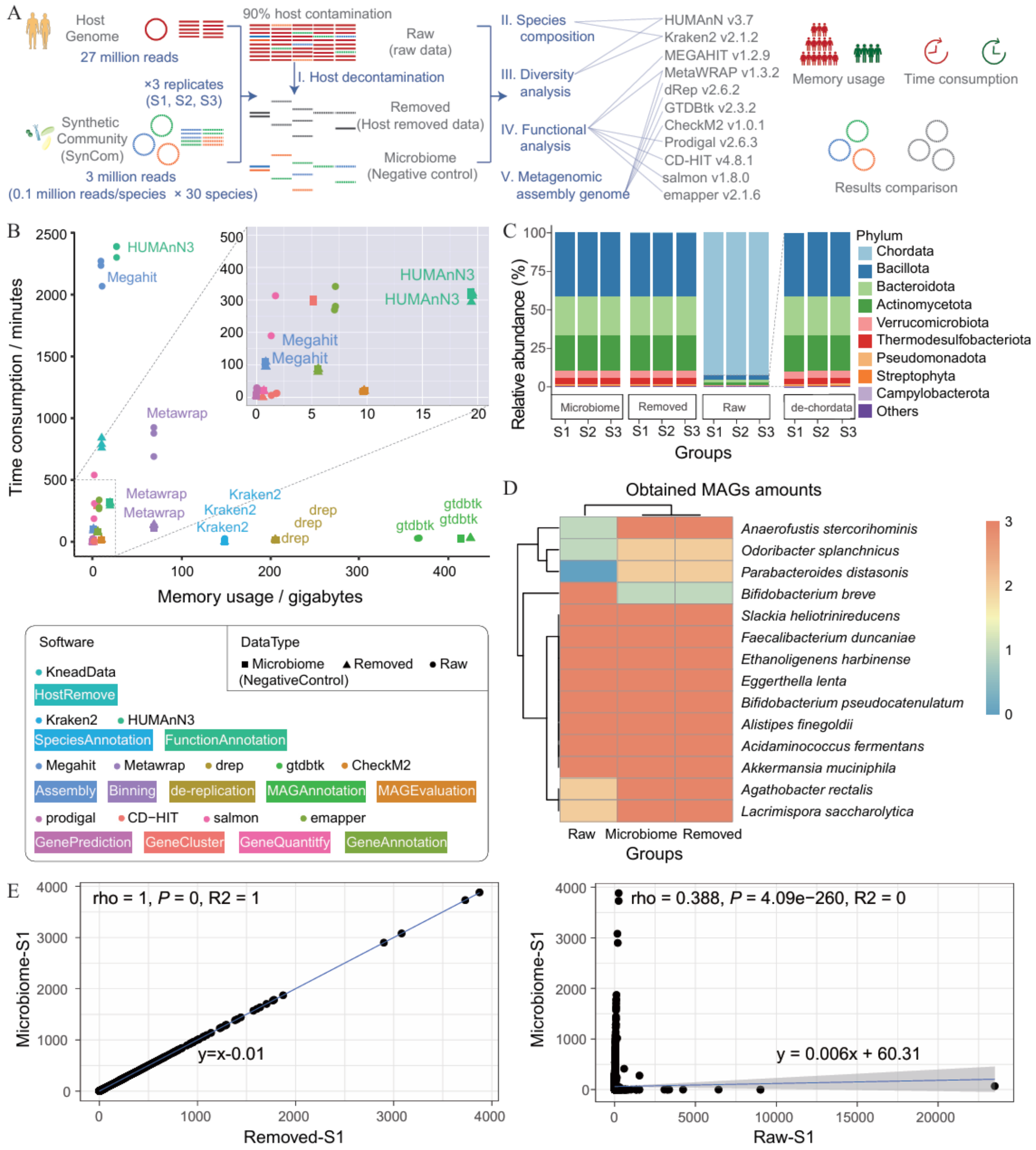

Figure 1. Host contamination consumed extra computing resources and affected the accuracy of the results in metagenomic analysis.

A. Simulated data design and the main downstream pipeline. Three samples (S1, S2, S3) with 90% host contamination were generated from the genomes of 30 bacteria and *Homo sapiens*. The raw data (Raw) underwent host decontamination to produce the removed data (Removed). The microbiome data was used as a negative control (Microbiome). Subsequent downstream analyses included host decontamination, species composition, diversity analysis, functional analysis, and metagenomic assembly genome evaluations. B. Host contamination increased computing resource consumption by 7.63 to 20.55 times in Megahit and HUMAnN3. The performance in terms of time and memory usage during downstream analyses was assessed on three samples (~9 GB per sample). C. The relative abundance at the phylum level. We also displayed the composition of raw data without chordata (de-chordata) to demonstrate that host removal can accurately reflect the true

microbiota composition. D. Evaluation of metagenomic assembly genome (MAG) amounts. Removed data can generate more MAGs than raw data during binning in all samples. E. Correlation assessment of gene ontology (GO) terms between microbiome data and removed data (left) or raw data (right) in S1 group. Each step of analysis was based on three sample replicates, with each replicate consisting of 30 million paired-end 150 bp reads (~ 9 GB).

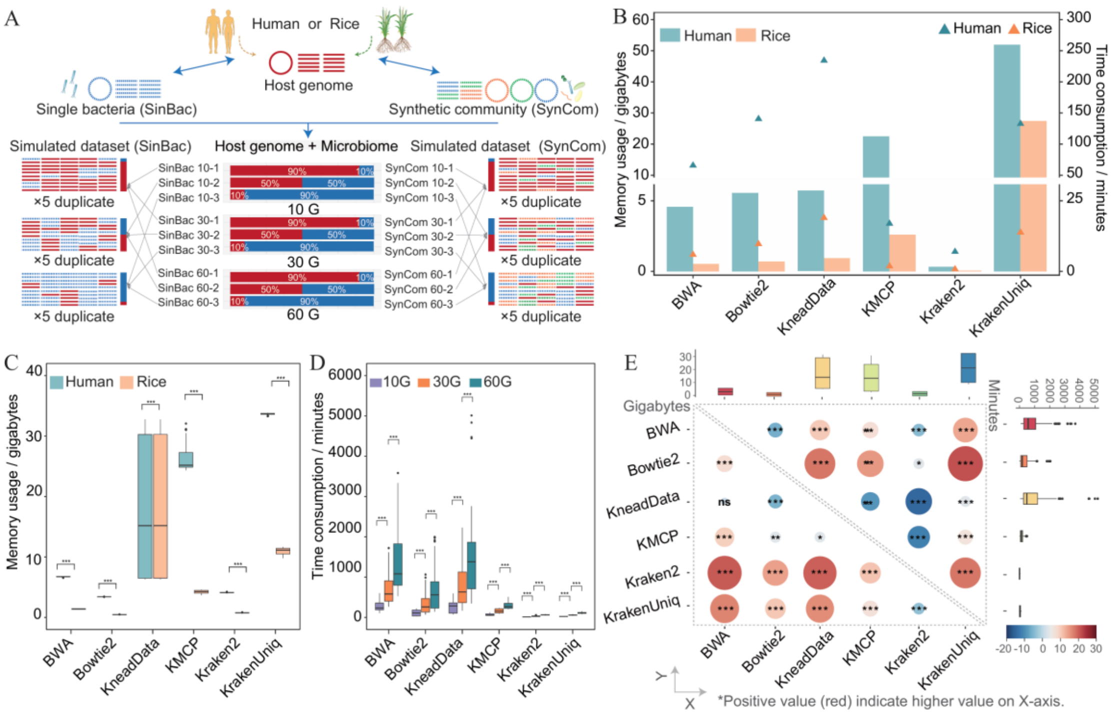

Figure 2. Benchmarking calculates resources of six host removal software on simulated human and rice metagenomic data.

The computational resources of all software were mostly influenced by the host reference genome size and the metagenomic data size, with Kraken2 consistently utilizing minimal computational resources.

A. Simulating metagenome datasets using CAMISIM. The datasets were designed to encompass diverse scenarios, featuring varying proportions of host genome contamination. Derived from human or rice genomes, the datasets come in three different sizes, each containing either a single bacterium (SinBac) or a synthetic community (SynCom, detail in Table S2-1). B. Comparison of time and memory usage in software for indexing host reference genome. The size of reference genome affects the resource consumption, with Kraken2 utilizing the fewest resources during the indexing step. C. Memory usage for different software, measured in giga bytes (Gb). The maximum computational memory usage is influenced by the host reference genome, except in KneadData. D. Running time of different software, showing in minutes. The decontamination process for large datasets requires more time. E. Memory usage (top-right diagonal) and execution time (bottom-left diagonal) among different software based on Kruskal-Wallis test. Positive values (red circle) indicate higher time or memory requirements for the software on the X-axis. The size of the circles

represents the Z-value, which is the standardized scores corresponding to each pairwise comparison. Kraken2 was observed to use significantly lower time and memory usage compared to others. '*' is shown as significant difference. (ns, not significant; *, $P \leqslant 0.05$; **, $P \leqslant 0.01$; ***, $P \leqslant 0.001$.)

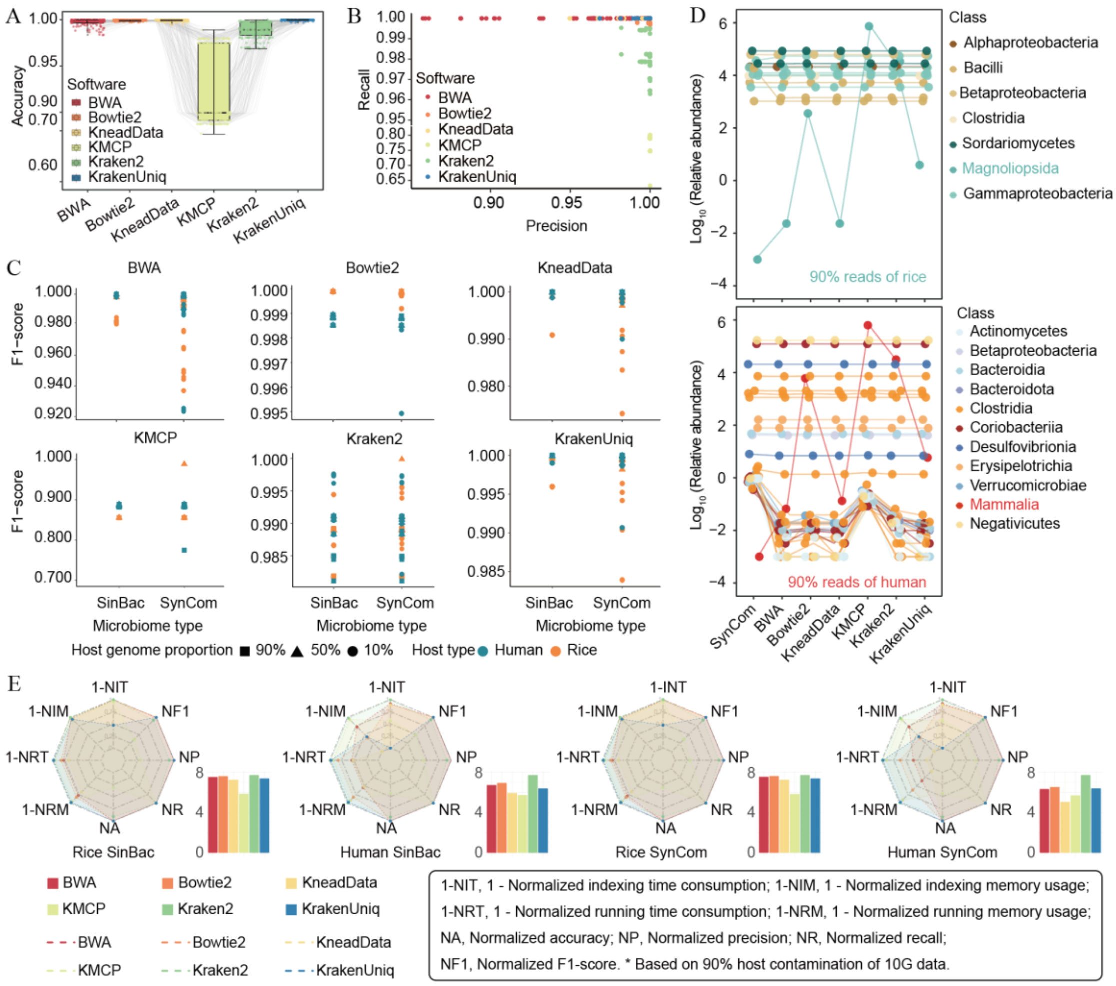

Figure 3. Assessing the accuracy of host contamination removal across various software. The alignment-based software displayed a higher rate of false positives, consequently diminishing the accuracy of microbiome information, while *k*-mer software exhibited an elevated occurrence of false negatives, thereby contributing to contamination of the data with host genome sequences. A. Accuracy among six software, showing BWA, Bowtie2, KneadData and KrakenUniq perform well. Accuracy = (True positive + True negative) / (True positive + True negative + False positive + False negative). B. Precision-recall of software. The alignment-based software (BWA, Bowtie2 and KneadData) exhibited higher false positive (some microbiota reads misaligned as host genome for removal), resulting in reduced microbiome information. However, the *k*-mer software (KMCP, Kraken2 and KrakenUniq) showed increased false negative (some host reads not be found), leading to the host genome contamination. Precision = Ture positive / (Ture positive+ False positive), Recall = True positive/ (True positive + False negative). C. High host contamination rate and microbiome complex rate reduce F1-score in six software. F1-score = 2 * Precision * Recall/ (Precision + Recall). D. Composition of the metagenomic dataset with a synthetic community after host contamination

removal using six software based on 90% host contamination. BWA and KneadData retained lower host contamination in alignment-based software, and KrakenUniq and Kraken2 retained lower host contamination in *k*-mer based software. E. Comparative analysis of computational efficiency and host contamination removal performance across simulated 60 Gbps datasets with 90% host contamination. The bar plot presented summary values for all indicators, highlighting Kraken2's excellence in comprehensive comparisons. The abbreviations for the indicators are as follows: 1-NIT, 1 - Normalized indexing time consumption; 1-NIM, 1 - Normalized indexing memory usage; 1-NRT, 1 - Normalized running time consumption; 1-NRM, 1 - Normalized running memory usage; NA, Normalized accuracy; NP, Normalized precision; NR, Normalized recall; NF1, Normalized F1-score. SinBac, Single bacterium; SynCom, Synthetic community.

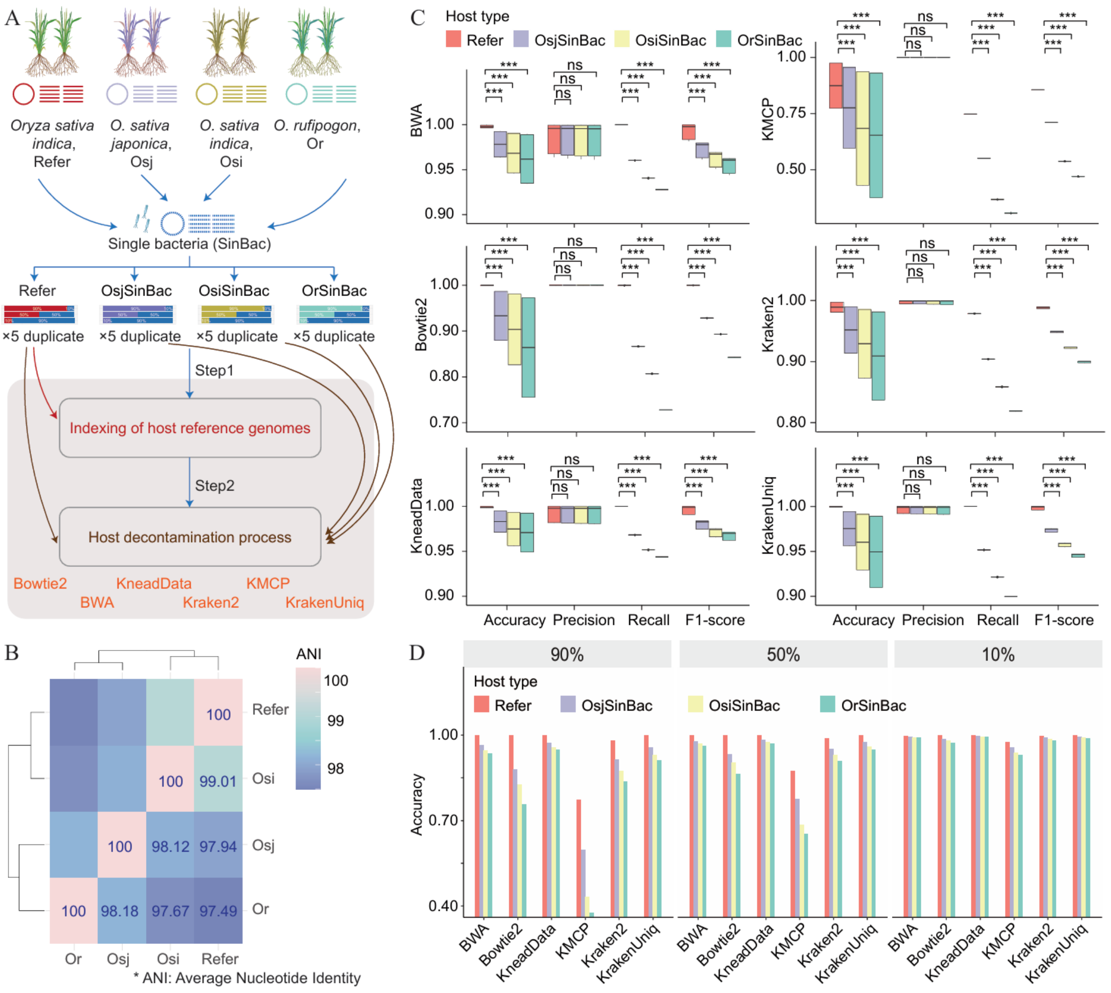

Figure 4. Impact of lacking a host reference genome on the performance of host decontamination tools

A. Impact of the absence of host reference genome on host decontamination tools. Simulated datasets were derived from three *Oryza* species (*Oryza sativa japonica*, Osj; *Oryza sativa indica*, Osi, *Oryza rufipogon*, Or) as hosts and a single bacterium. Each dataset contained varying levels of host DNA contamination (10%, 50%, and 90%) and was 10 Gbps in size, with five replicates per condition. The reference genome of *Oryza sativa indica* (refer) was used to create the indexing database for various host removal tools. All simulated data were aligned to this reference database to evaluate the performance of these tools in the absence of a specific host reference genome. B. Average nucleotide identity (ANI) analysis using FastANI. AIN values for *Oryza sativa japonica* (Osj), *Oryza sativa indica* (Osi), *Oryza rufipogon* (Or) and reference genome (*Oryza sativa indica*, refer) were shown. Osi (99.01%) showed the highest similarity to the reference genome, followed by Osj (97.94%) and Or (97.49%). C. Accuracy, precision, recall and F1-score of six tools on the simulated metagenomic data from the *Oryza* genus. All tools demonstrated significantly lower accuracy, recall, and F1-score for OsjSinBac, OsiSinBac, and OrSinBac compared to the reference data when aligned to the indexing database, which was created using the reference genome. D. Accuracy index of different software across various host genome proportion (90%, 50%, 10%). High host contamination of metagenomic data in the absence of host reference genome notably

affected the performance of existing host removal tools.

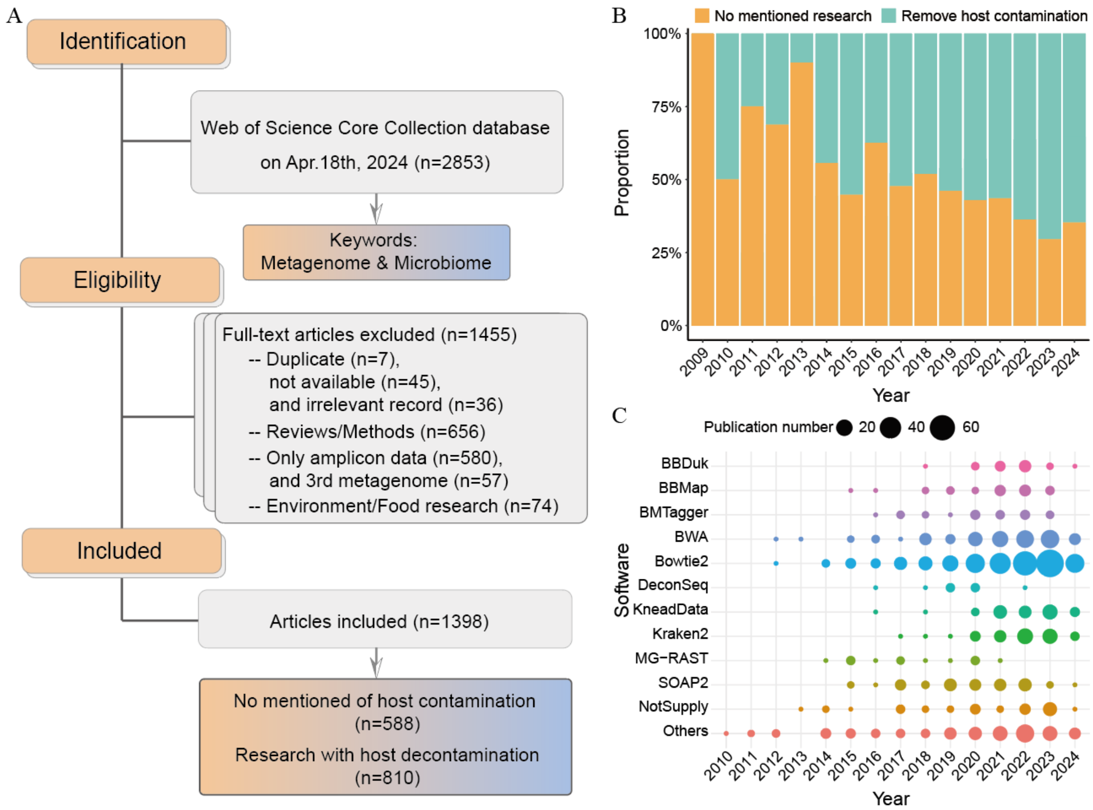

Figure S1. Literature search for existing studies addressing host contamination.

A. Literature search criteria. B. The proportion of publications mentioning the removal of host contamination. C. The existing software utilized for removing host genomes.

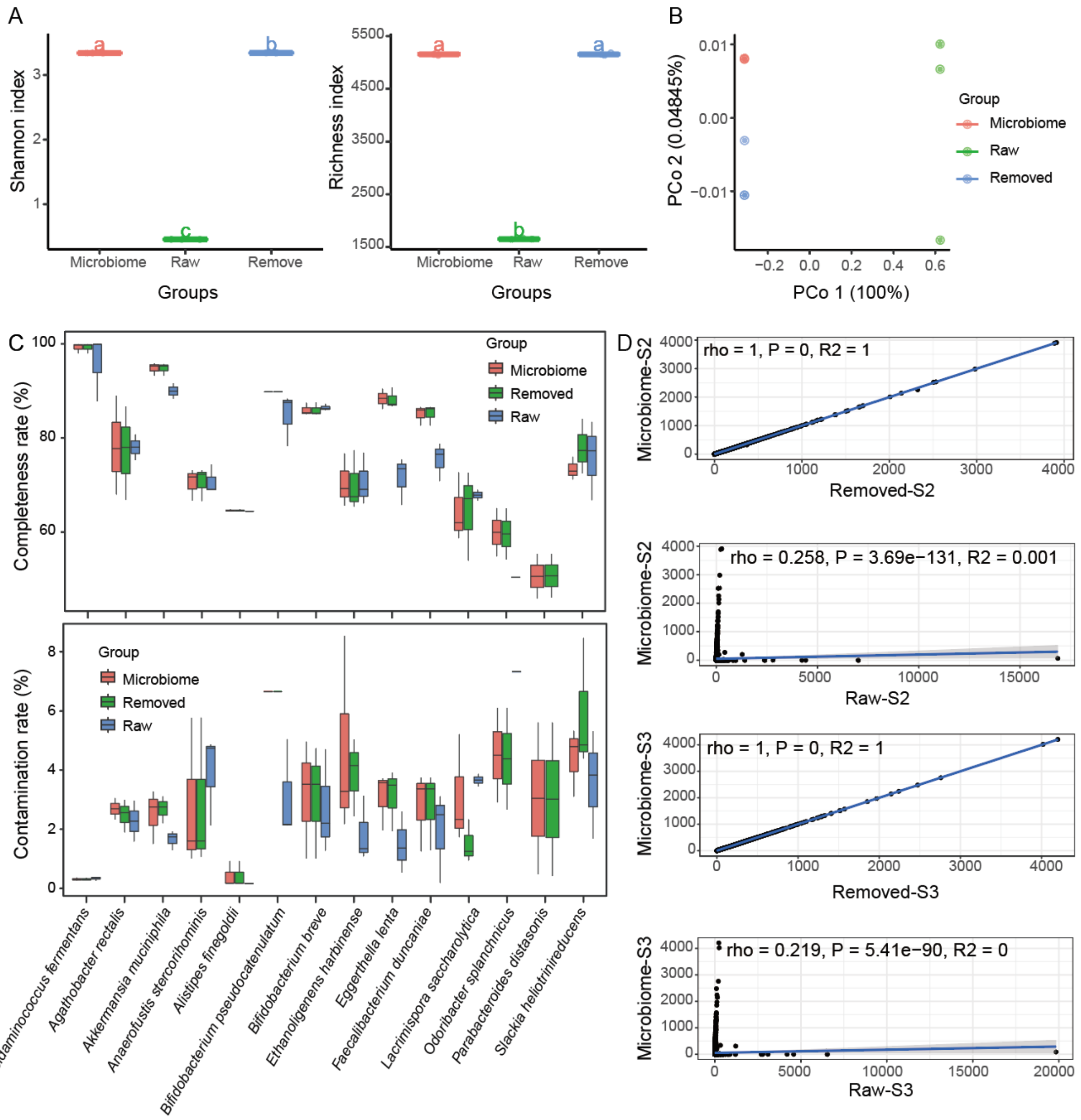

Figure S2. Comparison of host removal performance on the accuracy of the results in metagenomic analysis.

A. Shannon index, richness index, and principal coordinates analysis (PCoA) across microbiome removed and raw data. B. The completeness and contamination rates of MAGs (metagenome-assembled genomes) were assessed in microbiome, removed and raw data. C. Correlation assessment of gene ontology (GO) terms between microbiome data and removed data or raw data in S2 and S3 samples.

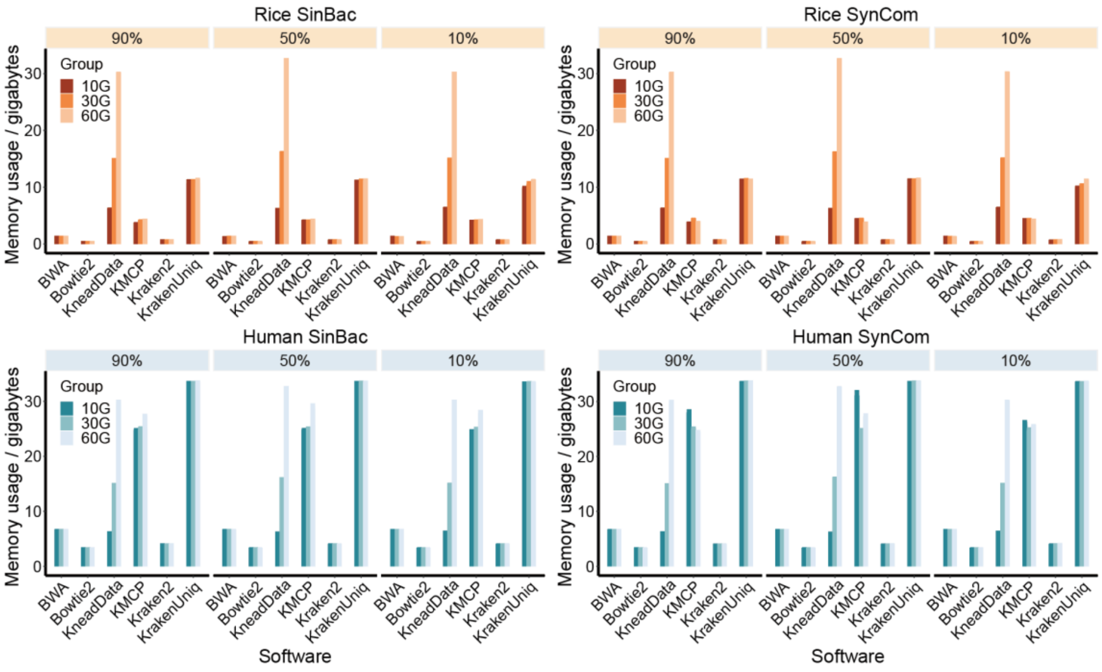

Figure S3. Memory usage during the host removing process of BWA, Bowtie2, KneadData, KMCP, Kraken2, KrakenUniq in simulation rice and human metagenome. SinBac, Single Bacteria; SynCom, Synthetic Community.

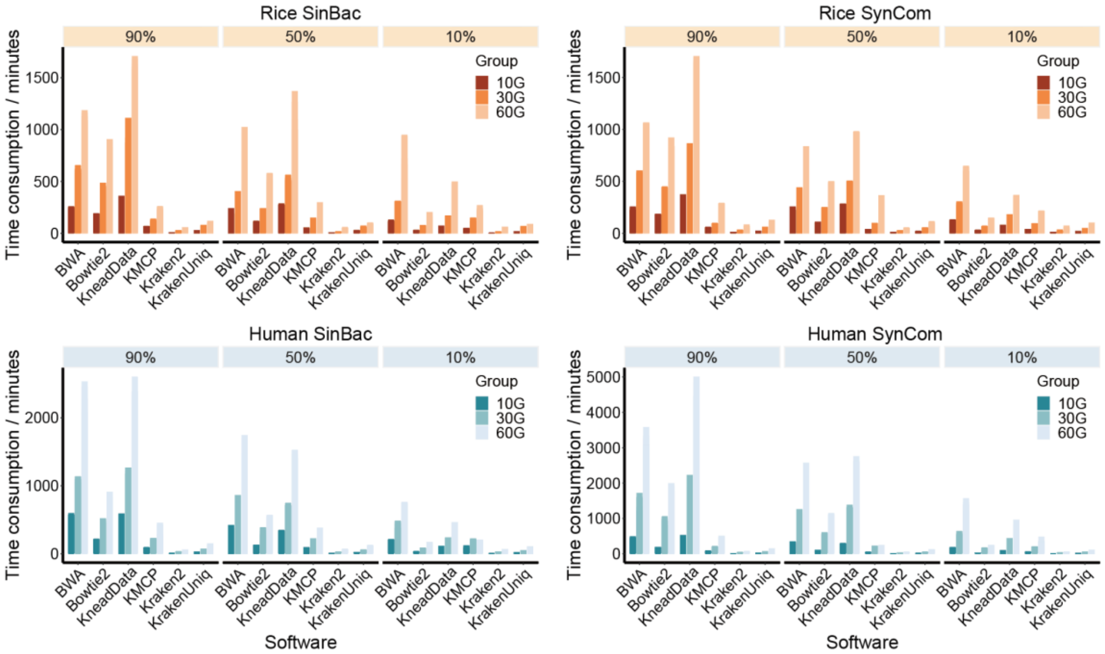

Figure S4. Time consumption during the host removing process of BWA, Bowtie2, KneadData, KMCP, Kraken2, KrakenUniq. SinBac, Single bacterium; SynCom, Synthetic community.

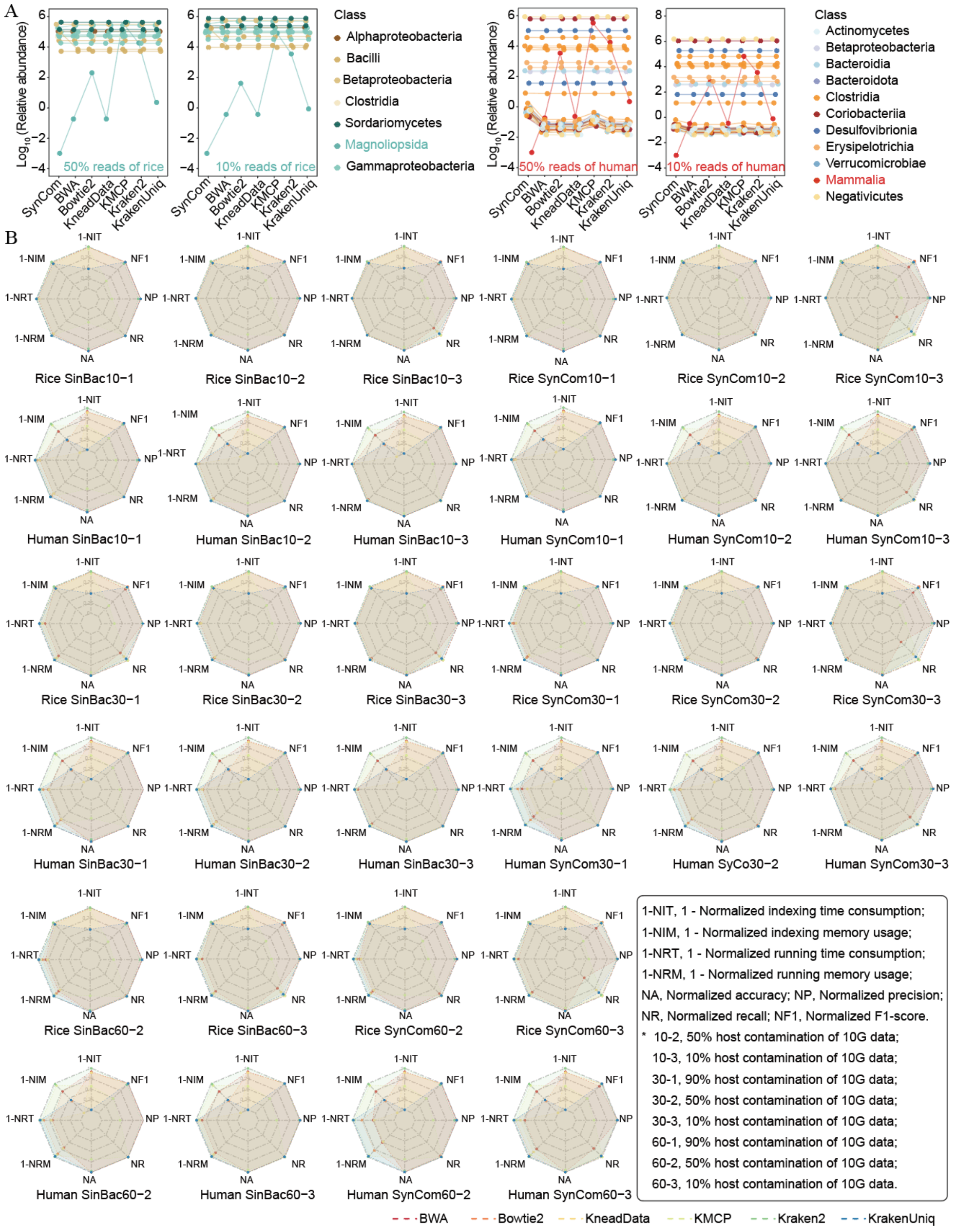

Figure S5. Comparative analysis of composition, computational efficiency and host contamination removal performance across six software.

A. Composition of the metagenomic dataset with a synthetic community after host contamination removal using six software. B. Computational efficiency and host contamination removal performance. 1-NIT, 1 - Normalized indexing time consumption; 1-NIM, 1 - Normalized indexing memory usage; 1-NRT, 1 - Normalized running time consumption; 1-NRM, 1 - Normalized running memory usage; NA, Normalized accuracy; NP, Normalized precision; NR, Normalized recall; NF1, Normalized F1-score. SinBac, Single bacterium; SynCom, Synthetic community.

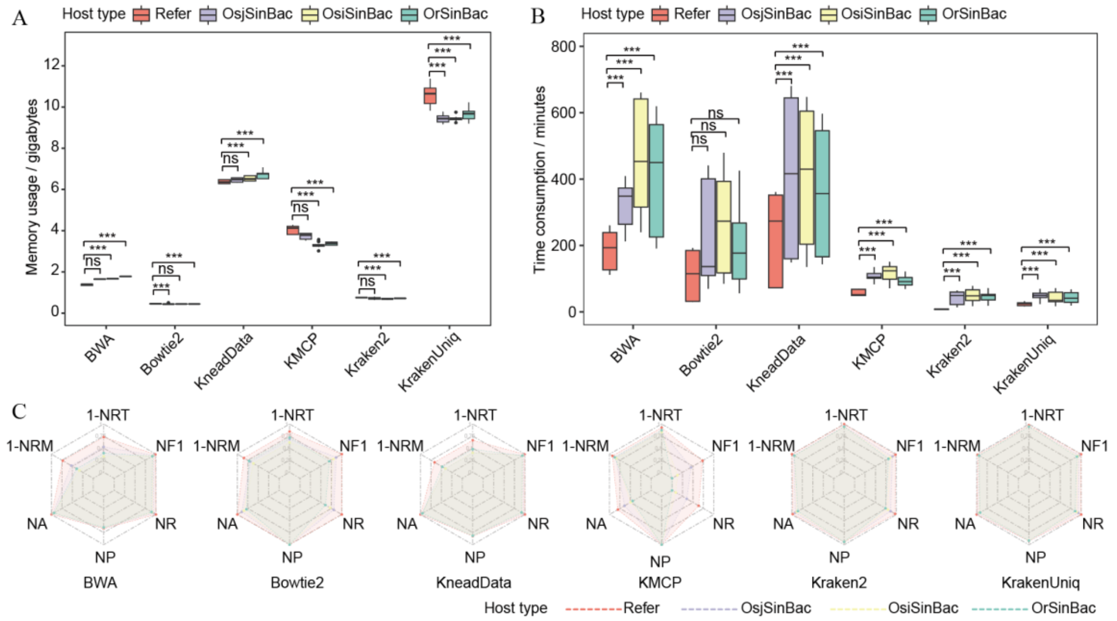

Figure S6. Assessing the performance of six tools at the genus level.

A. Memory usage during the host removing process for six tools across OsjSinBac, OsiSinBac, OrSinBac and reference metagenomic data. B. Time consumption during the host removing process for six tools across OsjSinBac, OsiSinBac, OrSinBac and reference metagenomic data. C. Computational efficiency and host contamination removal performance for six tools across OsjSinBac, OsiSinBac, OrSinBac and reference metagenomic data. 1-NRT, 1 - Normalized running time consumption; 1-NRM, 1 - Normalized running memory usage; NA, Normalized accuracy; NP, Normalized precision; NR, Normalized recall; NF1, Normalized F1-score. OsjSinBac, *Oryza sativa japonica* with a single bacterium; OsiSinBac, *Oryza sativa indica* with a single bacterium; OrSinBac, *Oryza rufipogon* with a single bacterium.

Manuscript-trackedchange

Click here to access/download;Manuscript;Manuscript-trackedchange.docx

~~Benchmarking metagenomics tools for removing host contamination~~Benchmarking short-read metagenomics tools for removing host contamination

Formatted: Font: 10.5 pt, Not Bold, Font color: Red

Yunyun Gao[1*], Hao Luo[1*], Hujie Lyu[2], Haifei Yang[1,3], Salsabeel Yousuf[1], Shi Huang[4], Yong-Xin Liu[1#]

[1]Shenzhen Branch, Guangdong Laboratory of Lingnan Modern Agriculture, Genome Analysis Laboratory of the Ministry of Agriculture and Rural Affairs, Agricultural Genomics Institute at Shenzhen, Chinese Academy of Agricultural Sciences, Shenzhen 518120, China

[2]Department of Life Sciences, Imperial College of London, London SW7 2AZ, UK

[3]College of Life Sciences, Qingdao Agricultural University, Qingdao 266000, China

[4]Faculty of Dentistry, The University of Hong Kong, Hong Kong SAR, China

[#] Correspondence: liuyongxin@caas.cn (Yong-Xin Liu)

* These two authors contributed equally to this work.

ORCIDs

Yunyun Gao: https://orcid.org/0000-0003-3389-9385

Hao Luo: https://orcid.org/0009-0005-3391-8576

Yong-Xin Liu: https://orcid.org/0000-0003-1832-9835

Abstract

The rapid evolution of metagenomic sequencing technology offers remarkable opportunities to explore the intricate roles of microbiome in host health and disease, as well as to uncover the unknown structure and functions of microbial communities. However, the swift accumulation of metagenomic data poses substantial challenges for data analysis. Contamination from host DNA can substantially compromise result accuracy, and increase additional computational resources by including non-target sequences. In this study, we assessed the impact of computational host-DNA decontamination on downstream analyses, highlighting its importance in producing accurate results efficiently. We also evaluated the performance of conventional tools like KneadData, Bowtie2, BWA, KMCP, Kraken2, and KrakenUniq, each offering unique advantages for different applications. Furthermore, we highlighted the importance of an accurate host reference genome, noting that its absence negatively affected the decontamination performance across all tools. Our findings underscore the need for careful selection of decontamination tools and reference genomes to enhance the accuracy of metagenomic analyses.

Keywords

Metagenome, microbiome, host removal, microbial enrichment

Background

The advancement of second-generation sequencing technology and data analysis methods has greatly facilitated microbiome research, broadening our horizons in the widespread influences of microbiome on their host [1-3]. Compared to amplicon sequencing, shotgun metagenomic sequencing offers comprehensive assessments of bacterial communities with less bias, and improved resolution in identifying profiles at the species, strain, or functional levels [4]. As sequencing technology costs rapidly decreased and sequencing depth continues to expand, the volume of metagenomic data is growing exponentially [5]. Some studies even producing over 100 giga base pairs (Gbps) per sample to characterize the dark matter of the microbiome in human gut [6]. However, a major challenge exists when analyzing the metagenome data from complex host-associated microbiomes, such as those found in saliva, throat, and vaginal swabs. These samples often, contain over 90% human-aligned reads [4, 7, 8] due to the high contamination of host DNA. This contamination undermines the characterization of microbiomes, especially for low abundant species [7], leading to biased observations of the true underlying microbial composition. Additionally, privacy concerns have become particularly significant when the host is human [9], highlighting the importance of removing host contamination.

Despite efforts to remove host contamination during the experimental stage [8, 10-13], particularly in DNA isolation, residual DNA remains polluted with numerous host DNA fragments. The overall efficacy of different experimental protocols varies, and potential biases for the preferential enrichment of specific microbial taxa remain a concern. Even after significant host DNA reduction, high biomass samples like mucosal microbiome can still exhibit up to 90% host contamination in metagenomic data [8, 10]. This persistence is due to differences in cell and genome size between animals and microbiomes. Similarly, in low biomass samples like endophytic microorganisms, around 70% of host sequencing reads may still be present despite efforts to collect and concentrate the microbiota [14]. This high level of contamination often necessitates deeper sequencing to adequately capture the microbial reads of interest. Sequencing these unwanted host DNA reads, followed by computational removal from large NGS datasets, is both wasteful and time-consuming [15]. It These limitations not only compromiscompromisese the accuracy of downstream analyses and consumes valuablebut also waste research timeer's efforts and computing resources. This underscores the importance of devising a host contamination removing tool in the data analysis stage that is both accurate and efficient.

After searching 2,853 publications using the keywords 'metagenome' and 'microbiome' (Figure S1A, Table S1), we found that 57.94% of the studies addressed the removal of host contamination, with a discernible increasing trend from 2015 to 2024. The absence of standardized criteria for selecting host decontamination software has led to the use of 51 different tools, many relying on alignment and *k*-mer strategies. Among these, ten tools exhibited notable popularity, generally employ two main strategies: alignment-based and the *k*-mer approaches [9, 16, 17]. The alignment

software, such as Bowtie2 [18], BWA [19], aligning sequencing reads to reference genomes. And Kraken2 [20], KMCP [21] are popular *k*-mer based software that identify exact matches between small substrings (*k*-mers) from the reads in the reference database. Besides, some host contamination removal pipelines integrate these modules. For instance, DeconSeq [22] integrates a modified version of BWA, while KneadData (http://huttenhower.sph.harvard.edu/kneaddata) integrates Bowtie2. Several new tools, like Hostile [9], HoCoRT [16] have also been developed to enhance the accuracy of host decontamination process. Although some studies have explore the impact of varying amounts of host DNA on microbiomes [23, 24], the impact of ~~depleting~~ removing host DNA contamination on the bioinformatic downstream analysis and the microbial genome assembly remains unclear [12].

In this study, we will compare the efficiency of metagenomic sequencing on microbiome by removing host contamination, and thoroughly evaluate the accuracy and speed of state-of-the-arts in computational host DNA decontamination. These results will serve as a guide for researchers in rationally selecting suitable tools for processing various metagenomic datasets.

Results

High host contamination increased the processing time and skewed interpretation the microbiome results

Here we simulated three groups (S1, S2, S3) of data using CAMISIM with 90% host contamination (Table S1-2), incorporating ~~genomes~~ microbial reads from 30 species, each represented at equal abundance, along with~~microbiota and~~ human reads from *Homo sapiens* (GRCh38). We used KneadData, a popular host decontamination software in recent years, to remove host contamination from the raw data (Raw), resulting in host-removed data (Remove), while the 30 microbial groups served as a negative control (Microbiome). ~~Based on these simulations, we evaluated the impact of host contamination removal on metagenomic analysis of the microbiome (Figure 1A).~~

Our metagenomic analysis involved key steps such as species composition, diversity analysis, functional analysis, and metagenome-assembled genome (MAG) evaluations (Figure 1A). Based on these simulations, we evaluated the impact of host contamination removal on metagenomic analysis of the microbiome (Figure 1A), focusing on memory usage, processing time, and the effects on the accuracy of the results.

Throughout the analysis, ~~During the metagenomic analysis,~~ no significant differences in memory usage were observed among the R~~r~~aw data, R~~r~~emoved data, and M~~m~~icrobiome data during high-memory steps exceeding 100 gigabytes (GBs), such as species-level taxonomic annotation (Kraken2), de-replication (drep), and MAG annotation (GTDBtk). However, compared to the R~~r~~aw data, the host-read-~~depleted~~removed data significantly reduced the run time of downstream analyses (Figure 1B). Specifically, processing the host-~~depleted~~ removal data took 5.98 times shorter for binning (MetaWRAP), 7.63 times shorter for function annotation (HUMAnN3), and 20.55 times shorter for assembly (MEGAHIT). And the average processing time for Remove data was 139.14

minutes (min) for MetaWRAP compared to 832.64 min for rRaw data, 308.92 min for HUMAnN3 compared to 2357.95 min for Rraw data, and 106.59 min for MEGAHIT compared to 2190.27 min for Rraw data. Additionally, handling the negative control data (Microbiome) required similar resources to the host-depleted removal data in terms of both memory usage and time consumption.

Compared to mMicrobiome data, rRaw data altered the relative abundance of microbiota community, while Rremove data showed a similar composition to that annotated by Kraken2 (Figure 1C). Interestingly, the released remaining taxa were similar to the microbiome Microbiome data, after removing the Chordata (the phylum of *Homo sapiens*) from the rRaw data (Figure 1C, de-chordata group). There was no difference in richness index between microbiome Microbiome data and Rremove data, whereas Rraw data showed a significantly lower richness index than both (Figure S2A). And principal coordinates analysis (PCoA) was performed to visualize changes in community composition, revealing that the first axes of PCoA explained 100% of the overall variations,. This observation –suggested low dimensionality and distinct separation of sample groups. Specifically, and samples from rRaw data were clearly separated from those of Mmicrobiome and the rRemoved data along PCo1 (Figure S2B). This finding underscores the effectiveness of host decontamination in highlighting the underlying microbial community structure.

Despite simulating Mmetagenomic data from 30 microbiota, with each species having 0.1 million reads, only 14 metagenome-assembled genomes (MAGs) were obtained. No significant differences were detected in completeness rate and contamination rates among the microbiome Microbiome data, raw Raw data and rRemoved data (Figure S2C). However, the number of MAGs was much more in Mmicrobiome and rRemoved data compared to rRaw data, except for *Bifidobacterium breve*, which was detected in all three groups (S1, S2, S3) in Rraw data, but only in S2 in Mmicrobiome and Rremoved data (Figure 1D). Next, we compared the gene ontology (GO) terms to mMicrobiome data. We found a stronger higher correlation similarity in GO terms between Rremoved data and Mmicrobiome data than that between rRaw data and microbiome Microbiome data (Figure 1E, Figure S2D), indicating that the host removal process results in more specific gene function annotation.

Kraken2 was fast and low-resource tool for host removal.

To further compare the differences in host removal among various software toolsTo ensure a comprehensive evaluation, we obtained generated 1080 simulated metagenomic datasets, using CAMISIM. These datasets which include single bacterium (SinBac) and synthetic community (SynCom) across various sizes (10 Gbps, 30 Gbps, and 60 Gbps)encompass three distinct sizes (10 Gbps, 30 Gbps, and 60 Gbps) and representing both single bacterium (SinBac) and synthetic community (SynCom) of microbiomes. The simulations were conducted separately for human (*Homo sapiens*) and rice (*Oryza sativa indica*) hosts, each with three 90%, 50%, and 10% levelslevels of host contamination (10%, 50%, and 90%), enabling a nuanced exploration of host genome contamination removal across various conditions (Figure 2A, see Methods for more details).

For convenience, we have assigned abbreviations to various datasets. For example, SinBac10-1 refers to a ~~denoted a~~ 10 Gbps dataset with 90% host genome reads ~~of reads originating from the host genome~~ and 10% reads from a single bacterium genome. Similarly, SynCom 30-2, represented a 30Gbps dataset with 50% host genome reads and 50% reads from the synthetic community genome. ~~Similarly,~~ SynCom ~~SinBac~~ 60-3, refers to a ~~represented a~~ 60Gbps dataset ~~with an even split~~ with ~~of~~ 1~~9~~0% host genome reads ~~reads from the host genome~~ and ~~10~~90% reads from the synthetic community genome (Figure 2A). Based on these simulated data, we compared the computational resources required and host decontamination performance of six existing tools: KneadData, Bowtie2, and BWA (for alignment-based software), and KMCP, Kraken2, and KrakenUniq (for *k*-mer strategy software).

Before removing host contamination, indexing of host reference genomes is crucial. In this study, we constructed reference genomes for *Homo sapiens* (GRCh38) and *Oryza sativa indica* (GWHBFPX00000000) with sizes approximately 3.1 Gbps and 373.8 Megabase pairs (Mbps), respectively. Kraken2 utilized minimal computational resources for indexing both human and rice genomes, requiring only 0.3 gigabyte (Gb) memory and taking 6.94 minutes (min) to create a custom database for the human genome (Figure 2B). In contrast, the other five tools required an average of 18.05 Gb memory and 117.98 min.

We then compared the resource consumption during the host contamination removal process across six software. In summary, Bowtie2 (1.95 Gb (0.410, 3.42)), and Kraken2 (2.47 Gb (0.710, 4.12)) demonstrated the lowest maximum memory usage across all simulated datasets for alignment and *k*-mer based software, respectively (Figure 2C, Figure S3). These values were significantly lower than that of the other four tools, with BWA requiring 3.995 Gb (1.40, 6.74), KneadData requiring 15.17 Gb (6.47, 30.27), KMCP requiring 14.45 Gb (4.27, 25.110), KrakenUniq requiring 22.410 Gb (11.13, 33.67). Regarding data size, handling 60 Gbps data consumed significantly more resources than 10 Gbps data in KneadData and Kraken2. Different host types also significantly influenced resource consumption across all software (Table S3-1), with human data requiring notably more resources than rice data ($P < 0.05$). Whereas, no significant difference between the different microbiome types for all software, except for KrakenUniq (Table S3-1).

For time usage, the *k*-mer software (KMCP, 156.10 min (90.47, 231.16), Kraken2, 29.34 min (13.42, 55.45), and KrakenUniq, 59.23 min (26.66, 98.14), required less time than the alignment-based software (BWA 582.26 min (300.15, 1065.64), Bowtie2 209.00 min (111.66, 512.61), KneadData 501.38 mins (287.41, 1177.06)), with Kraken2 demonstrating significantly shorter execution times compared to other tools (Figure 2E, Table S3-2). The diversity of microbiomes exhibited no impact on processing time across all six software (Table S3-2). But, the size of the metagenomic data significantly influenced execution time (Figure 2D, 2E), this emphasized the importance of utilizing fast software for efficient processing, especially when dealing with large metagenomic datasets.

Formatted: Not Highlight

Formatted: Not Highlight

Formatted: Not Highlight

Formatted: Not Highlight

Formatted: Not Highlight

Simulated metagenomic data from human took more time to process than data from rice across all tools, indicating that host genome complexity leads to increased processing time (Figure S4, Table S3). Noticeably, a high proportion of host genome contamination significantly reduced the speed of alignment-based tools like BWA, Bowtie2, KneadData, and KrakenUniq (Figure S4, Table S3-2). For instance, processing a large 60 Gbps metagenomic dataset containing 90% human genome contamination resulted in a significant increase in processing time (1.35-fold in KrakenUniq, 2.59-fold in BWA, 5.36-fold in KneadData, and 6.76-fold in Bowtie2) compared to the same dataset with only 10% contamination. This suggested that these tools may be less suitable for datasets with substantial host contamination. Nevertheless, this significant slowdown was not observed in KMCP and Kraken2.

Performance in host decontamination accuracy of six software

Then four metrics (accuracy, recall, precision, and F1-score) were calculated to evaluate the performance of host decontamination accuracy in six software based on with the 1080 simulated data generated according to the rule in Figure 2A. We observed significant differences among the six software ($P < 0.05$) in the accuracy, recall, precision, and F1-score (Table S3-4, S3-5, S3-6, S3-7). In terms of accuracy, the alignment-based software (BWA, 0.9989 (0.9966, 0.9998), Bowtie2, 0.9997 (0.9988, 0.9998), and KneadData, 0.9997 (0.9989, 0.9998)) outperformed the *k*-mer software (KMCP, 0.8947 (0.8133, 0.9748); Kraken2, 0.9891 (0.9832, 0.9974)), with the exception of KrakenUniq (0.9998 (0.9994, 0.9999)), which consistently exhibited a high and stable performance (Figure 3A). However, the alignment-based software exhibited lower precision performance (BWA, 0.9980 (0.9853, 0.9996), Bowtie2, 0.9999 (0.9999, 0.9999), and KneadData, 0.9981 (0.9971, 0.9998)), potentially leading to an increased number of false positives associated with the host genome. This implied that some microbiome reads may be erroneously mapped as part of the host genome and subsequently was removed as contamination. Conversely, *k*-mer software (KMCP, 0.7686 (0.7477, 0.7925), Kraken2 0.9787 (0.9787, 0.9823), and KrakenUniq 0.9999 (0.9999, 1)) showed lower recall performance, leading to an increased number of false negatives associated with the host genome. This suggested that some host reads may be erroneously unmapped, thereby retaining some host contamination in the downstream analyses (Figure 3B). For F1-score (2 * Precision * Recall/ (Precision + Recall)), the type of microbiome, host type, and the proportion of host genome all influenced the performance on these tools. Notably, BWA, KneadData, and KrakenUniq performed significantly better on human datasets compared to rice datasets. Conversely, Bowtie2 showed great performance with rice dataset (Figure 3C).

We compared the composition of the metagenomic dataset with a synthetic community (SynCom) after host decontamination using the abovementioned six software (Figure 2A). The classes Magnoliopsida and Mammalia displayed low values (i.e., the $\log_{10}$-transformed relative abundance) in BWA, KneadData, and KrakenUniq (Figure 3D). Among these, BWA and KneadData, both alignment-based tools, demonstrate superior performance in removing host contamination

compared to KrakenUniq, which, as a *k*-mer based tool, tends to be less effective.~~This suggested that BWA and KneadData that belong to the alignment-based software category exhibit superior performance in host contamination removal performance, and KrakenUniq that falls into the *k*-mer based software category tends to remove less host contamination.~~ All tools, except for KMCP, identified some few groups, which belongs to Actinomycetes, Clostridia, Negativicutes, as host contamination, and removed them from the raw data (Figure 3D). For the integrated comparison of resource consumption and the performance of host decontamination across software, we normalized all data using min-max normalization. This enables a comparative analysis of computational efficiency and host contamination removal effectiveness across simulated datasets (Figure 3E, Figure S5). Based on the summarized normalized data, Kraken2 showed significant excellence ($P < 0.05$) under the high levels (90%) of host contamination, in both human (7.8093 (7.7236, 7.8215)) and rice (7.8268 (7.7109, 7.8330)) datasets through comprehensive comparisons. Additionally, when comparing the host removal performance among alignment-based software, focusing on normalized accuracy (NA), normalized precision (NP), normalized recall (NR), and normalized F1-score (NF1) under the high levels (90%) of host contamination, KneadData demonstrated significant superiority ($P < 0.05$) in human (3.9996 (3.9996, 3.9997)).

The absence of accurate host reference genome affected the decontamination performance

Next, we assessed the impact of lacking a host reference genome on the effectiveness of existing host decontamination software. Three *Oryza* species—*Oryza sativa japonica* (GWHBFOO00000000, Osj), *Oryza sativa indica* (GWHBFPT00000000, Osi), and *Oryza rufipogon* (GWHBFHN00000000, Or)—were selected as the resource of host metagenomic reads to generate simulated datasets. *Oryza sativa indica* (GWHBFPX00000000, Refer) was chosen as the reference genome (Figure 4A). The average nucleotide identity (ANI) between the three species (Osj, Osi, Or) and the reference genome, showed that Osi had the highest similarity (99.01%) to the reference genome, followed by Osj at 97.94% and Or at 97.49% (Figure 4B). The simulated metagenomic reads derived from a single bacterium (SinBac) was generated as before, resulting in three datasets (OsjSinBac, OsiSinBac, OrSinBac) contained varying levels of host DNA contamination (10%, 50%, and 90%) and each 10 Gbps in size. Subsequently, the indexing databases for the six tools were built using the reference genome, and we compared the performance of host decontamination tools (BWA, Bowtie2, KneadData, KMCP, Kraken2 and KrakenUniq) under these conditions (Figure 4A).

In terms of time and memory consumption, KneadData and KrakenUniq used more memory (Figure S6A), while alignment software took more time than *k*-mer software (Figure S6B), as previously described. When comparing with reference metagenomic data, processing OsjSinBac, OsiSinBac, OrSinBac data resulted in all software requiring significantly more time (Figure S6B). Notably, the absence of a closely aligned and accurate ~~a~~ host reference genome negatively impacted the decontamination performance of all tools (Figure 4C). Specifically, accuracy, recall, and F1-score

Formatted: Font: Italic

were significantly lower for datasets representing OsjSinBac, OsiSinBac, and OrSinBac compared to reference data aligned to the indexing database created with the reference genome. Precision, however, did not show significant differences, indicating that decontaminated datasets still contained some residual host reads.

Then we compared the comprehensive performance for all software during the absence of accurate host reference genome. Based on the summarized normalized data of resource consumption during running and host decontamination metrics (accuracy, precision, recall, and F1-score). All software performed better with reference metagenomic data (5.41 (5.15, 5.91)) than with OsjSinBac (4.93 (4.32, 5.50)), OsiSinBac (4.54 (3.91, 5.35)), OrSinBac (4.64 (4.08, 5.19)) datasets, whereas less difference across these datasets was observed with Kraken2 and KrakenUniq (Figure S6C). Moreover, while different tools performed better on high-host contamination samples with a reference genome, this advantage was not evident in the absence of a reference genome. In the presence of 90% host contamination, all tools showed a significant reduction in accuracy (Figure 4D, Table S4-1), emphasizing the importance of a host reference genome for high contamination metagenomic data.

Methods

Literature searches and data collection

A literature search was conducted in the Web of Science Core Collection Database on April 18th, 2024, using the search terms ‘metagenome’ and ‘microbiome’ (Table S1-1). Only research articles were utilized to gather information on software usage (Figure S1A). We excluded publications solely focused on amplicon sequencing data, long-read metagenomes, environmental samples or food samples. Subsequently, we compiled the percentage of publications mentioning host contamination removing and the number of publications for each software.

Simulated dataset description for the downstream analysis

Three groups of data (S1, S2, S3) with 90% host contamination were simulated, including genomes from 30 microbiota species and *Homo sapiens* (GRCh38). The 30 microbial species were randomly selected based on a previously published human-associated microbial community [25]. Here we used CAMISIM to generate metagenomic data for 30 microbial genomes, simulating paired-end reads (PE150) with three replicates for each microbial species (number_of_samples = 3). ~~E~~Each dataset comprised 3 million microbial reads, with 0.1 million reads per microbial species, combined with 27 million reads from Homo sapiens. The reads were labeled accordingly before mixing ~~ach dataset contained 0.1 million reads of microbiome data and 27 million reads of *Homo sapiens*~~ (Table S1-2). Among the microbiome species, six belonged to Actinomycetota, 15 to Bacillota, six to Bacteroidota, and one each to Pseudomonadota, Thermodesulfobacteriota, and Verrucomicrobiota. To remove host contamination from the raw data (Raw), we used KneadData, a bioinformatics tool specifically designed for this purpose. The output from KneadData processing constituted the host removal ~~depleted~~ data (Remove). And the 30 microbial groups served as a negative control

(Microbiome) to evaluate the accuracy and effectiveness of the host contamination removal process.

Metagenomic analysis

In order to compare the difference of analysis in direct data (Raw data) and the host contamination removing data (Remove data), we selected 10 Gbps synthetic community datasets of human with varying host contamination levels (Figure 1A). The resource consumption was tested as following description, and metagenomic analysis directly refer to the steps of EasyMetagenome 1.10 pipeline [26]. Briefly, taxonomic profiling was performed using Kraken2 [27], with PlusPFP database, and relative abundances were obtained using Bracken. Functional profiling was preformed via HUMAnN3 [28] using Uniref90 gene families. After assembling the metagenomic data using Megahit 1.0. [29], metagenomic binning and bin refinement were conducted using MetaWRAP [30]. The MetaWRAP refinement aimed to enhance the quality of our MAG binning, utilizing the parameters -c 50 -x 10, which retained only bins with completeness greater than 50% and contamination less than 10%. Redundancies in the metagenome-assembled genomes (MAGs) were removed with dRep v2.6.2. MAGs were annotated using GTDBtk v2.3.2 [31], and their quality was evaluated using CheckM2 v1.0.1 [32]. Gene prediction was performed using Prodigal v2.6.3 [33], clustering of genes with CD-HIT v4.8.1 [34], quantification of genes with salmon v1.8.0, and gene annotation with emapper v2.1.6. Then alphaα diversity and beta d β dddiversity analyses was analyzed using R 4.2.3 as descripted in EasyAmplicon [35]. The completeness and contamination rate of metagenome assembly genomes (MAGs) were normal measurement data, thus they were presented as median (*P25*, *P75*). And we also annotated the GO terms with eggnog-mapper, and calculated their correlation with microbiome data, that only retained microbiome from raw data, using Spearman. The pipeline could be found in https://github.com/YunyunGao374/HostPurge/blob/main/0HostDecontaminationImpactiononDownstreamAnalysis.sh.

Simulated dataset description for the comparison of six tools using human and rice data

We selected human and rice, both of significant economic and medical importance, and with well-characterized genomes, as the focus of our study. Six tools were selected for analysis, three of which are alignment-based software (BWA, Bowtie2, and KneadData), while the others are *k*-mer based (KMCP, Kraken2, KrakenUniq). Simulated datasets were generated using CAMISIM, and analyses were conducted using default or author-recommended parameters. To ensure comparability and reliability, each dataset comprised five replicates. These datasets covered various data sizes (10 Gbps, 30 Gbps, 60 Gbps), different level of host DNA contamination (910%, 50%, 190%), and diverse microbial complexities (single bacterium, SinBac, or synthetic community, SynCom) from both human and rice samples (Figure 2A). Each dataset had five replicates per condition. For the rice SynCom, we selected 14 commonly reported species, chosen randomly from known rice-associated microbes. The human SynCom was constructed with 35 species (Table S2-1), based on a previously published human-associated microbial community [25]., For the SinBac simulations,

Formatted: Font: SimSun

Formatted: Not Highlight

we utilized default parameters in CAMISIM to generate paired-end reads (PE150) for both the host and a single bacterial genome, conducting five replicates for each simulation (number_of_samples = 5). We then mixed reads from the host and microbial genomes in varying proportions of host DNA contamination.~~ with differential mode in CAMISIM~~ In the case of the SynCom simulations, we employed the same ways as in the SinBac for generating the host's metagenomic data. However, for the microbial metagenomic data, we used the differential mode in CAIMISM. The resulting data were then mixed according to different level of host DNA contamination. ~~The synthetic rice community consists of common microorganisms randomly selected from widely reported strains, while the synthetic human community refers to a microbial community previously published [25].~~ The code for the generation of simulated data can be found in https://github.com/YunyunGao374/HostPurge/blob/main/1HostDecontaminationSoftwareComparison.sh. Taxonomy information and their genome IDs are provided in Table S2-1.

To ensure comprehensive evaluation, we generated 1080 simulated datasets using CAMISIM. These datasets encompass three distinct sizes (10 Gbps, 30 Gbps, and 60 Gbps) and representing both simple (SinBac) and complex (SynCom) microbiomes. The simulations were conducted separately for human (*Homo sapiens*, GRCh38) and rice (*Oryza sativa indica*, GWHBFPX00000000) hosts, each with three levels of host contamination (10%, 50%, and 90%), enabling a nuanced exploration of host genome contamination removal across various conditions (Figure 1A). For the species which contains multiple chromosomes, we just download all of them and stimulated the information, and all of reference and fasta information have been attached in Table S2-1. Here, for convenience, we have assigned abbreviations to various datasets. For example, SinBac10-1 denoted a 10 Gbps dataset with 90% of reads originating from the host genome and 10% from single bacteria genome. Similarly, SynCom 30-2, represented a 30 Gbps dataset with an even split of 50% reads from the host genome and 50% from the synthetic community genome. Based on these simulated data, we assessed the impact of metagenomic sequencing on microbiome by removing host contamination.

Simulated dataset description for the comparison of six tools within a genus level

To assess the performance of host decontamination tools at the genus level, we utilized three rice species and simple microbiomes separately: *Oryza sativa japonica* (GWHBFOO00000000, Osj), and *Oryza sativa indica* (GWHBFPT00000000, Osi), *Oryza rufipogon* (GWHBFHN00000000, Or). For each species, we generated datasets by combining the host genome with a simple bacterial genome, resulting in three separate datasets: Or with simple bacteria (OrSinBac), Osj with simple bacteria (OsjSinBac), and Osi with simple bacteria (OsiSinBac). Each dataset was simulated using CAMISIM with different levels of host DNA contamination (10%, 50%, and 90%) for 10 Gbps datasets, with five replicates per condition.

Oryza sativa indica served as the reference genome (GWHBFPX00000000, Refer), and indices were created for six tools (BWA, Bowtie2, KneadData, KMCP, Kraken2, KrakenUniq), as described above. Detailed fasta information is provided in Table S2-1. These simulated datasets were analyzed

to compare the performance of the six tools in removing host contamination within the genus. The comparison involved evaluating computational resources and the effectiveness of host decontamination. Genome similarity was calculated using fastANI v1.34 [36], and the impact of metagenomic sequencing on microbiome have been assessed with the tested of following performance metrics. Based on these datasets, we aimed to determine how each tool performs in removing host contamination when genome data is limited within the same genus.

Performance metrics tests

All software utilized eight threads for building the database and running the processes across simulated datasets, and the running code of different software can be found at https://github.com/YunyunGao374/HostPurge/blob/main/1HostDecontaminationSoftwareComparison.sh. We assessed a comparative analysis of resource consumption, focusing on maximum RAM usage and processing time. Additionally, we evaluated their performance metrics including true positive (TP), true negative (TN), false positive (FP), and false negative (FN). And the precision, recall and F1-score were also calculated, with the following formulas: Accuracy = (TP + TN) / (TP + FN + FP + TN), Precision = (TP / (TP + FP)), Recall = (TP / (TP + FN)), F1-score = 2 * Precision * Recall / (Precision + Recall) [37]. Then all bioinformatics analysis were all analyzed within R 4.2.3. We conducted normality and homogeneity tests on all data. Measurement data were expressed as mean ±SE for normally distributed data and as median (*P25, P75*) for non-normally distributed data. For normally distributed data with homogeneous variances in two-group comparisons, we used paired t-tests. Non-normally distributed or non-homogeneous data were analyzed using non-parametric Wilcoxon tests. In multiple group comparisons, normally distributed data with homogeneous variances were analyzed with ANOVA, while non-normally distributed or non-homogeneous data were analyzed with non-parametric Kruskal-Wallis tests [38]. Bonferroni post-hoc tests were performed on the data within each group to analyze the differences between different datasets [39]. For the average species composition plot, when dealing with data containing zeros, we adopted the straightforward method of adding a positive constant (0.001) to all leaf trait values [40], and then taking the logarithm of the resulting average relative abundance. For the integrated comparison of resource consumption and the performance of host purge in each software, we normalized all data use min-max normalization [41], which is a technique that performs a linear transformation of the original data. Data visualization was done using ggplot2 package, and *P*~~p~~ ≤ 0.05 was regarded as statistically significant. All relevant data and plot code can be found at https://github.com/YunyunGao374/HostPurge.

Discussion

While 57.94% of publications eliminated host contamination before analyzing their data, while the remaining opted to analyze their data directly~~ (Figure S1B)~~, potentially sacrificing valuable microbiome information in the process. Some software was developed to decontaminate the host genome [9, 18-22], but limited research provided the distinctions among them. For accelerating the

reproducibility, comparability, and standardization of metagenomic data, it is important to understand how these different tools, broadly referred to downstream analyses [11]. And the significant influence of function annotation, species annotation, reads assembly and binning in the consumption of computational resources, exists the necessity to remove host contamination, particularly in ultra-high-depth sequencing data.

In our study, we generated a simulated dataset with 0.1 million reads of 30 microbiome species, and 27 million reads of *Homo sapiens*. Among the microbiome species, six belonged to Actinomycetota, 15 to Bacillota, six to Bacteroidota, one each to Pseudomonadota, Thermodesulfobacteriota, and Verrucomicrobiota ~~(Table S1-2)~~. However, the proportion of the relative abundance were not equal, and two phyla not included in the simulation (Streptophyta and Campylobacterota) were annotated ~~(Figure 1C)~~. This discrepancy may be attributed to the limitations of the Kraken2 annotation process, despite our use of the most comprehensive database (PlusPFP), as well as the the heterogeneity of our simulated data. In addition, although each of simulated data comprises 30 microbial groups, we only obtained 14 MAGs. ~~These may be attributed to the heterogeneity of our simulated data~~The strict bin refinement parameters (-c 50, -x 10) may have further contributed to this outcome, highlighting ~~or~~ the potential need for further improvement of the ~~assembly and binning~~ metagenomic analysis software. Nonetheless, the importance of the host decontamination was still being demonstrated in terms of gene functional annotation. Meanwhile, the significant difference in the Shannon index between microbiome (negative control) data and raw or removed data highlights the importance of establishing a "gold standard" for host removal.

It is noteworthy that we observed significantly higher accuracy, precision, and F1-score in datasets with high (90%) compared to low (10%) levels of host contamination in KneadData. Similar trends were also observed in other alignment software ~~(Tables S3-5, S3-7)~~. This phenomenon may be attributed to two factors. Firstly, our simulated data, derived from the host genome used as the indexing reference database, resulted in a higher proportion of host reads for high host contamination data (90%) aligning to the host reference in alignment software (BWA, Bowtie2, KneadData). Secondly, reads from the microbiome may be challenging to distinguish from reference genomes, leading to a higher number of microbiome reads being discarded in datasets with lower host contamination (10%).

Additionally, the challenge of predicting the host genome when it's unavailable, potentially down to the genus, family, or order level, needs consideration. Interestingly, despite Osi having a closer ANI to the reference genome compared to Osj, the decontamination performance for OsiSinBac was not as effective as for OsjSinBac ~~(Figure 4C)~~. And the high host contamination showed a significant reduction of accuracy in all software ~~(Figure 4D)~~, emphasizing the importance of a host reference genome for high contamination metagenomic data [42]. The absence of an accurate host reference genome can lead to residual host sequences, which in turn reduces the precision of subsequent functional annotations. One possible solution might be the combination of alignment-based and *k*-

Formatted: Font: Italic

mer methods for more accurate host contamination removal. However, a unique challenge remains in distinguishing microbial sequences resulting from horizontal gene transfer (HGT) rather than host contamination. HGT between the microbiome and host genomes, often involving mobile genetic elements (MGEs), plays a crucial role in microbial adaptation to diverse environments. Metagenomic sequencing, particularly with short-read technologies, faces significant difficulties in accurately identifying these horizontally transferred gene regions [43]. In the future, advances in artificial intelligence algorithms [44] and long-read sequencing may help overcome not only host contamination but also the problem of horizontal transfer of bacteria.

Conclusion

In conclusion, host decontamination not only speeds up downstream analysis but also enhances the accuracy of gene function annotation, particularly in ultra-high-depth sequencing data. And each of these tools (BWA, Bowtie2, KneadData, Krakne2, KMCP and KrakenUniq) offers unique strengths that can be harnessed based on the specific requirements of a research study. Briefly, Bowtie2 and KneadData provides more accurate removal capabilities, albeit with increased computational demands. Kraken2 and KrakenUniq offer fast and user-friendly solution, while KMCP can retain more low-abundance taxa. When reference genomes are lacking, BWA and KneadData are less impacted among alignment software, and Kraken2 and KrakenUniq are less affected among *k*-mer software.

Understanding the trade-offs between speed, accuracy, and computational resources is crucial for selecting the most suitable tool for host DNA removal in metagenomic analyses. As research increasingly focuses on understanding the impact of host contamination on microbiome annotation, particularly for low abundance taxa [42], this study provides a comprehensive evaluation that lays the groundwork for refining tools and methodologies. Ultimately, these advancements will empower researchers to derive meaningful biological insights from complex metagenomic datasets.

Acknowledgments

The work was financially supported by the China Postdoctoral Science Foundation (2024M753580) ~~and~~ , National Natural Science Foundation of China (U23A20148, 32470055), the Agricultural Science and Technology Innovation Program (CAAS-ZDRW202308). We would like to acknowledge the support of the Prof. Wei Shen (Institute for Viral Hepatitis, The Second Affiliated Hospital, Chongqing Medical University, China) for any help during running the KMCP. And we sincerely thank Dr. Yao Wang (Agricultural Genomics Institute at Shenzhen, Chinese Academy of Agricultural Sciences, China) for her help in data analyses, Dr. Kai Peng (Yangzhou University, China) for his help in drawing the spider diagram.

Authors' contributions

Yong-Xin Liu, Shi Huang and Yunyun Gao conceived and coordinated the study. Yunyun Gao, Hao Luo, and Haifei Yang collected and analyzed the simulated data. Yunyun Gao and Hujie Lyu did the literature searches and data collection. Yong-Xin Liu, Shi Huang, Salsabeel Yousuf and Yunyun Gao

revised the manuscript. All authors have read the final manuscript and approved it for publication.

Conflict of interest

The authors declare that they have no conflicts of interest.

Data availability

The raw data of simulated metagenomic sequencing reads have been deposited at the Genome Warehouse (GWH) (https://bigd.big.ac.cn/gwh/) under PRJCA028271. All pipelines, data analyses, and plotting code can be found at https://github.com/YunyunGao374/HostPurge.

References

1. Rooks MG and Garrett WS. Gut microbiota, metabolites and host immunity. *Nature reviews immunology* 2016;6:341-52. https://doi.org/10.1038/nri.2016.42.

2. Gao Y, Li D and Liu Y-X. Microbiome research outlook: past, present, and future. *Protein & Cell* 2023;10:709-12. https://doi.org/10.1093/procel/pwad031.

3. Xia H, Zhang Z, Luo C, et al. MultiPrime: A reliable and efficient tool for targeted next‐generation sequencing. *iMeta* 2023;e143:1-16. https://doi.org/10.1002/imt2.143.

4. Saheb Kashaf S, Almeida A, Segre JA, et al. Recovering prokaryotic genomes from host-associated, short-read shotgun metagenomic sequencing data. *Nature protocols* 2021;5:2520-41. https://doi.org/10.1038/s41596-021-00508-2.

5. Tremblay J, Schreiber L and Greer CW. High-resolution shotgun metagenomics: the more data, the better? *Briefings in Bioinformatics* 2022;6:bbac443. https://doi.org/10.1093/bib/bbac443.

6. Jin H, Quan K, He Q, et al. A high-quality genome compendium of the human gut microbiome of Inner Mongolians. *Nature Microbiology* 2023;1:150-61. https://doi.org/10.1038/s41564-022-01270-1.

7. Pereira-Marques J, Hout A, Ferreira RM, et al. Impact of host DNA and sequencing depth on the taxonomic resolution of whole metagenome sequencing for microbiome analysis. *Frontiers in microbiology* 2019:1277. https://doi.org/10.3389/fmicb.2019.01277.

8. Wu-Woods NJ, Barlow JT, Trigodet F, et al. Microbial-enrichment method enables high-throughput metagenomic characterization from host-rich samples. *Nature Methods* 2023;11:1672-82. https://doi.org/10.1038/s41592-023-02025-4.

9. Constantinides B, Hunt M and Crook DW. Hostile: accurate decontamination of microbial host sequences. *Bioinformatics* 2023;12:btad728. https://doi.org/10.1093/bioinformatics/btad728.

10. Heravi FS, Zakrzewski M, Vickery K, et al. Host DNA depletion efficiency of microbiome DNA enrichment methods in infected tissue samples. *Journal of microbiological methods* 2020:105856. https://doi.org/10.1016/j.mimet.2020.105856.

11. Marchukov D, Li J, Juillerat P, et al. Benchmarking microbial DNA enrichment protocols from human intestinal biopsies. *Frontiers in genetics* 2023:1184473. https://doi.org/10.3389/fgene.2023.1184473.

12. Cheng WY, Liu W-X, Ding Y, et al. High sensitivity of shotgun metagenomic sequencing in colon tissue biopsy by host DNA depletion. *Genomics, Proteomics & Bioinformatics* 2022; https://doi.org/10.1016/j.gpb.2022.09.003.

13. Feehery GR, Yigit E, Oyola SO, et al. A method for selectively enriching microbial DNA from contaminating vertebrate host DNA. *PloS one* 2013;10:e76096. https://doi.org/10.1371/journal.pone.0076096.

14. Soto-Giron MJ, Kim J-N, Schott E, et al. The edible plant microbiome represents a diverse genetic reservoir with functional potential in the human host. *Scientific Reports* 2021;1:24017. https://doi.org/10.1038/s41598-021-03334-4.

15. Diao Z, Han D, Zhang R, et al. Metagenomics next-generation sequencing tests take the stage in the diagnosis of lower respiratory tract infections. *Journal of advanced research* 2022:201-12.

16. Rumbavicius I, Rounge TB and Rognes T. HoCoRT: host contamination removal tool. *BMC bioinformatics* 2023;1:371. https://doi.org/10.1186/s12859-023-05492-w.

17. Liu Y, Ghaffari MH, Ma T, et al. Impact of database choice and confidence score on the performance of taxonomic classification using Kraken2. *aBIOTECH* 2024:1-11. https://doi.org/10.1007/s42994-024-00178-0.

18. Langmead B and Salzberg SL. Fast gapped-read alignment with Bowtie 2. *Nature methods* 2012;4:357-9. https://doi.org/10.1038/nmeth.1923.

19. Li H and Durbin R. Fast and accurate short read alignment with Burrows–Wheeler transform. *bioinformatics* 2009;14:1754-60. https://doi.org/10.1093/bioinformatics/btp324.

20. Lu J, Rincon N, Wood DE, et al. Metagenome analysis using the Kraken software suite. *Nature protocols* 2022;12:2815-39. https://doi.org/10.1038/s41596-022-00738-y.

21. Shen W, Xiang H, Huang T, et al. KMCP: accurate metagenomic profiling of both prokaryotic and viral populations by pseudo-mapping. *Bioinformatics* 2023;1:btac845. https://doi.org/10.1093/bioinformatics/btac845.

22. Schmieder R and Edwards R. Fast identification and removal of sequence contamination from genomic and metagenomic datasets. *PloS one* 2011;3:e17288. https://doi.org/10.1371/journal.pone.0017288.

23. Nearing JT, Comeau AM and Langille MG. Identifying biases and their potential solutions in human microbiome studies. *Microbiome* 2021;1:113. https://doi.org/10.1186/s40168-021-01059-0.

24. McArdle AJ and Kaforou M. Sensitivity of shotgun metagenomics to host DNA: abundance estimates depend on bioinformatic tools and contamination is the main issue. *Access microbiology* 2020;4:e000104. https://doi.org/10.1099/acmi.0.000104.

25. Cheng AG, Ho P-Y, Aranda-Díaz A, et al. Design, construction, and in vivo augmentation of a complex gut microbiome. *Cell* 2022;19:3617-36. https://doi.org/10.1016/j.cell.2022.08.003.

26. Liu Y-X, Qin Y, Chen T, et al. A practical guide to amplicon and metagenomic analysis of microbiome data. *Protein & cell* 2021;5:315-30. https://doi.org/10.1007/s13238-020-00724-8.

27. Wood DE, Lu J and Langmead B. Improved metagenomic analysis with Kraken 2. *Genome biology* 2019:1-13. https://doi.org/10.1186/s13059-019-1891-0.

28. Abubucker S, Segata N, Goll J, et al. Metabolic reconstruction for metagenomic data and its application to the human microbiome. *PLoS computational biology* 2012;6:e1002358. https://doi.org/10.1371/journal.pcbi.1002358.

29. Li D, Liu C-M, Luo R, et al. MEGAHIT: an ultra-fast single-node solution for large and complex metagenomics assembly via succinct de Bruijn graph. *Bioinformatics* 2015;10:1674-6. https://doi.org/10.1093/bioinformatics/btv033.

30. Uritskiy GV, DiRuggiero J and Taylor J. MetaWRAP—a flexible pipeline for genome-resolved metagenomic data analysis. *Microbiome* 2018:1-13. https://doi.org/10.1186/s40168-018-0541-1.

31. Chaumeil P-A, Mussig AJ, Hugenholtz P, et al. GTDB-Tk v2: memory friendly classification with

the genome taxonomy database. *Bioinformatics* 2022;23:5315-6. https://doi.org/10.1093/bioinformatics/btac672.

32. Chklovski A, Parks DH, Woodcroft BJ, et al. CheckM2: a rapid, scalable and accurate tool for assessing microbial genome quality using machine learning. *Nature Methods* 2023;8:1203-12. https://doi.org/10.1038/s41592-023-01940-w.

33. Hyatt D, Chen G-L, LoCascio PF, et al. Prodigal: prokaryotic gene recognition and translation initiation site identification. *BMC bioinformatics* 2010:1-11. 10.1186/1471-2105-11-119.

34. Fu L, Niu B, Zhu Z, et al. CD-HIT: accelerated for clustering the next-generation sequencing data. *Bioinformatics* 2012;23:3150-2. https://doi.org/10.1093/bioinformatics/bts565.

35. Liu YX, Chen L, Ma T, et al. EasyAmplicon: An easy‐to‐use, open‐source, reproducible, and community‐based pipeline for amplicon data analysis in microbiome research. *iMeta* 2023;1:e83. https://doi.org/10.1002/imt2.83.

36. Jain C, Rodriguez-R LM, Phillippy AM, et al. High throughput ANI analysis of 90K prokaryotic genomes reveals clear species boundaries. *Nature communications* 2018;1:5114. https://doi.org/10.1038/s41467-018-07641-9.

37. Qin H, Ou L, Gao J, et al. DENA: training an authentic neural network model using Nanopore sequencing data of Arabidopsis transcripts for detection and quantification of N 6-methyladenosine on RNA. *Genome Biology* 2022;1:25. https://doi.org/10.1186/s13059-021-02598-3.

38. McKight PE and Najab J. Kruskal‐wallis test. *The corsini encyclopedia of psychology* 2010:1-. https://doi.org/10.1002/9780470479216.corpsy0491.

39. Duque M, Lee-Kubli CA, Tufail Y, et al. Sonogenetic control of mammalian cells using exogenous Transient Receptor Potential A1 channels. *Nature communications* 2022;1:600. https://doi.org/10.1038/s41467-022-28205-y.

40. Harris T, Ottaviani G, Mulligan M, et al. Trait hypervolumes based on natural history collections can detect ecological strategies that are distinct to biogeographic regions. *Journal of Ecology* 2023;2:314-26. https://doi.org/10.1111/1365-2745.14005.

41. Song Q, Lee J, Akter S, et al. Prediction of condition-specific regulatory genes using machine learning. *Nucleic Acids Research* 2020;11:e62-e. https://doi.org/10.1093/nar/gkaa264.

42. Gihawi A, Ge Y, Lu J, et al. Major data analysis errors invalidate cancer microbiome findings. *MBio* 2023;5:e01607-23. https://doi.org/10.1128/mbio.01607-23.

43. Brito IL. Examining horizontal gene transfer in microbial communities. *Nature Reviews Microbiology* 2021;7:442-53. https://doi.org/10.1038/s41579-021-00534-7.

44. Gao Y, Peng K, Bai D, et al. The Microbiome Protocols eBook initiative: Building a bridge to microbiome research. *iMeta* 2024;e182:1-7. https://doi.org/10.1002/imt2.182.

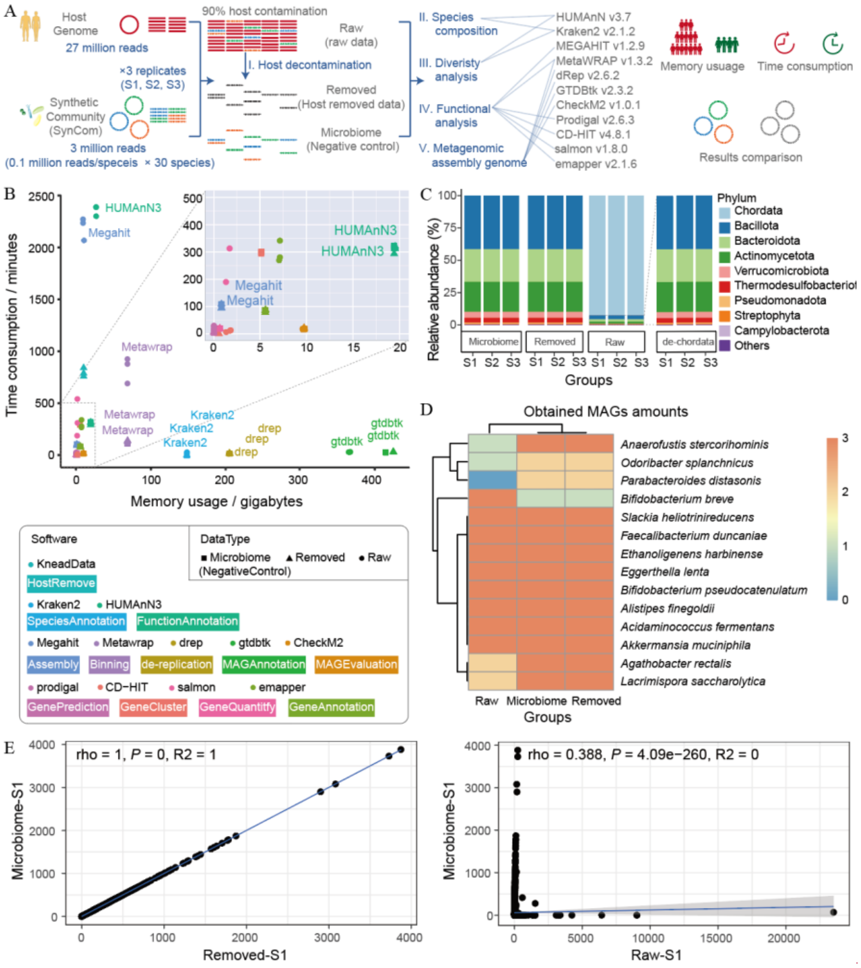

A
Host Genome
27 million reads
×3 replicates (S1, S2, S3)
Synthetic Community (SynCom)
3 million reads
(0.1 million reads/speceis × 30 species)
90% host contamination
Raw (raw data)
I. Host decontamination
Removed (Host removed data)
Microbiome (Negative control)
II. Species composition
III. Diveristy analysis
IV. Functional analysis
V. Metagenomic assembly genome
HUMAnN v3.7
Kraken2 v2.1.2
MEGAHIT v1.2.9
MetaWRAP v1.3.2
dRep v2.6.2
GTDBtk v2.3.2
CheckM2 v1.0.1
Prodigal v2.6.3
CD-HIT v4.8.1
salmon v1.8.0
emapper v2.1.6
Memory usage
Time consumption
Results comparison
B
Time consumption / minutes
Memory usage / gigabytes
HUMAnN3
Megahit
Metawrap
Kraken2
drep
gtdbtk
Software
KneadData
HostRemove
Kraken2
HUMAnN3
SpeciesAnnotation
FunctionAnnotation
Megahit
Metawrap
drep
gtdbtk
CheckM2
Assembly
Binning
de-replication
MAGAnnotation
MAGEvaluation
prodigal
CD-HIT
salmon
emapper
GenePrediction
GeneCluster
GeneQuantity
GeneAnnotation
DataType
Microbiome (NegativeControl)
Removed
Raw
C
Relative abundance (%)
Microbiome
Removed
Raw
de-chordata
S1 S2 S3
Groups
Phylum
Chordata
Bacillota
Bacteroidota
Actinomycetota
Verrucomicrobiota
Thermodesulfobacteriot
Pseudomonadota
Streptophyta
Campylobacterota
Others
D
Obtained MAGs amounts
Anaerofustis stercorihominis
Odoribacter splanchnicus
Parabacteroides distasonis
Bifidobacterium breve
Slackia heliotrinireducens
Faecalibacterium duncaniae
Ethanoligenens harbinense
Eggerthella lenta
Bifidobacterium pseudocatenulatum
Alistipes finegoldii
Acidaminococcus fermentans
Akkermansia muciniphila
Agathobacter rectalis
Lacrimispora saccharolytica
Raw
Microbiome
Removed
Groups
E
rho = 1, P = 0, R2 = 1
Microbiome-S1
Removed-S1
rho = 0.388, P = 4.09e−260, R2 = 0
Raw-S1

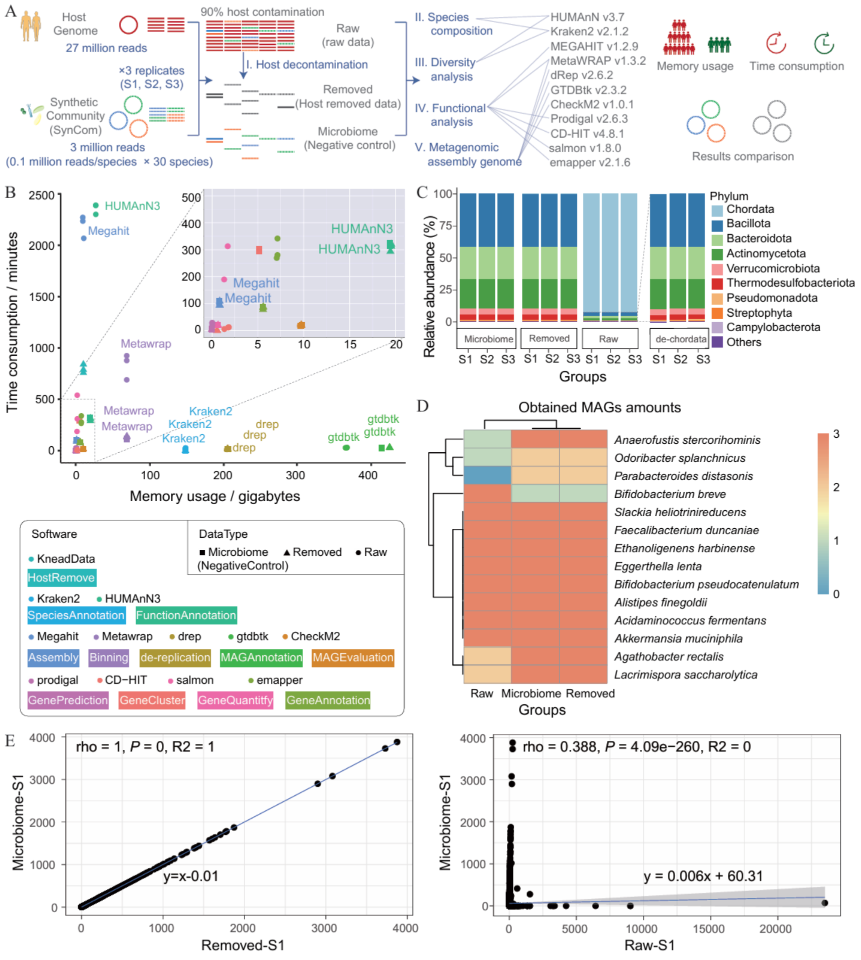

Figure 1. Host contamination consumed extra computing resources and affected the accuracy of the results in metagenomic analysis.

A. Simulated data design and the main downstream pipeline. Three samples (S1, S2, S3) with 90% host contamination were generated from the genomes of 30 bacteria and *Homo sapiens*. The raw data (Raw) underwent host decontamination to produce the removed data (Removed). The microbiome data was used as a negative control (Microbiome). Subsequent downstream analyses included host decontamination, species composition, diversity analysis, functional analysis, and metagenomic assembly genome evaluations. B. Host contamination increased computing resource consumption by 7.63 to 20.55 times in Megahit and HUMAnN3. The performance in terms of time and memory usage during downstream analyses was assessed on three samples (~9 GB per sample). C. The relative abundance at the phylum level. We also displayed the composition of raw data without chordata (de-chordata) to demonstrate that host removal can accurately reflect the true

microbiota composition. D. Evaluation of metagenomic assembly genome (MAG) amounts. Removed data can generate more MAGs than raw data during binning in all samples. E. Correlation assessment of gene ontology (GO) terms between microbiome data and removed data (left) or raw data (right) in S1 group. Each step of analysis was based on three sample replicates, with each replicate consisting of 30 million paired-end 150 bp reads (~ 9 GB).

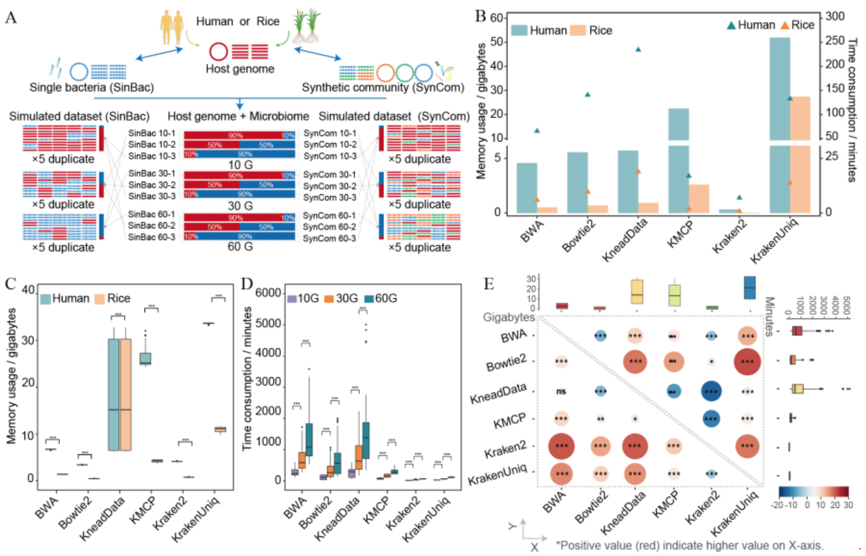

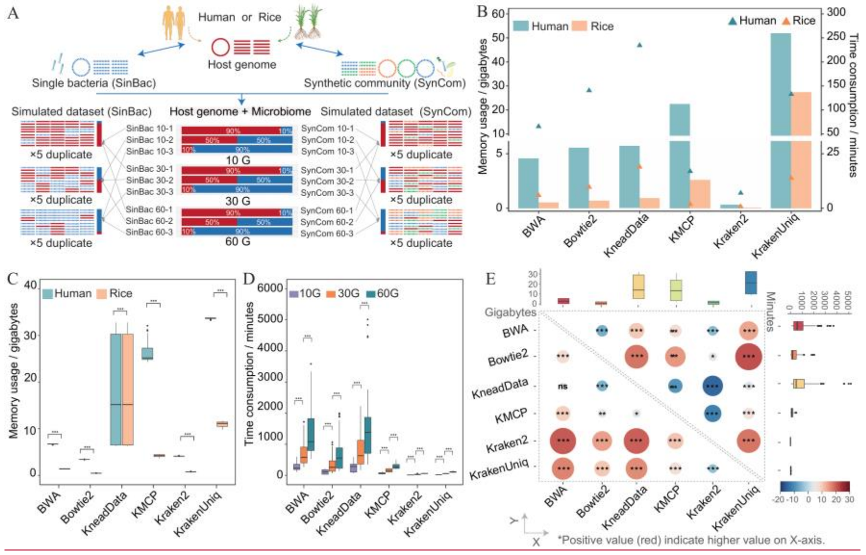

Figure 2. Benchmarking calculates resources of six host removal software on simulated human and rice metagenomic data.

The computational resources of all software were mostly influenced by the host reference genome size and the metagenomic data size, with Kraken2 consistently utilizing minimal computational resources.

A. Simulating metagenome datasets using CAMISIM. The datasets were designed to encompass diverse scenarios, featuring varying proportions of host genome contamination. Derived from human or rice genomes, the datasets come in three different sizes, each containing either a single bacterium (SinBac) or a synthetic community (SynCom, detail in Table S2-1). B. Comparison of time and memory usage in software for indexing host reference genome. The size of reference genome affects the resource consumption, with Kraken2 utilizing the fewest resources during the indexing step. C. Memory usage for different software, measured in giga bytes (Gb). The maximum computational memory usage is influenced by the host reference genome, except in KneadData. D. Running time of different software, showing in minutes. The decontamination process for large datasets requires more time. E. Memory usage (top-right diagonal) and execution time (bottom-left diagonal) among different software based on Kruskal-Wallis test. Positive values (red circle) indicate higher time or memory requirements for the software on the X-axis. The size of the circles represents the Z-value, which is the standardized scores corresponding to each pairwise comparison. Kraken2 was observed to use significantly lower time and memory usage compared to others. '*' is shown as significant difference. (ns, not significant; *, $P \leqslant 0.05$; **, $P \leqslant 0.01$; ***, $P \leqslant 0.001$.)

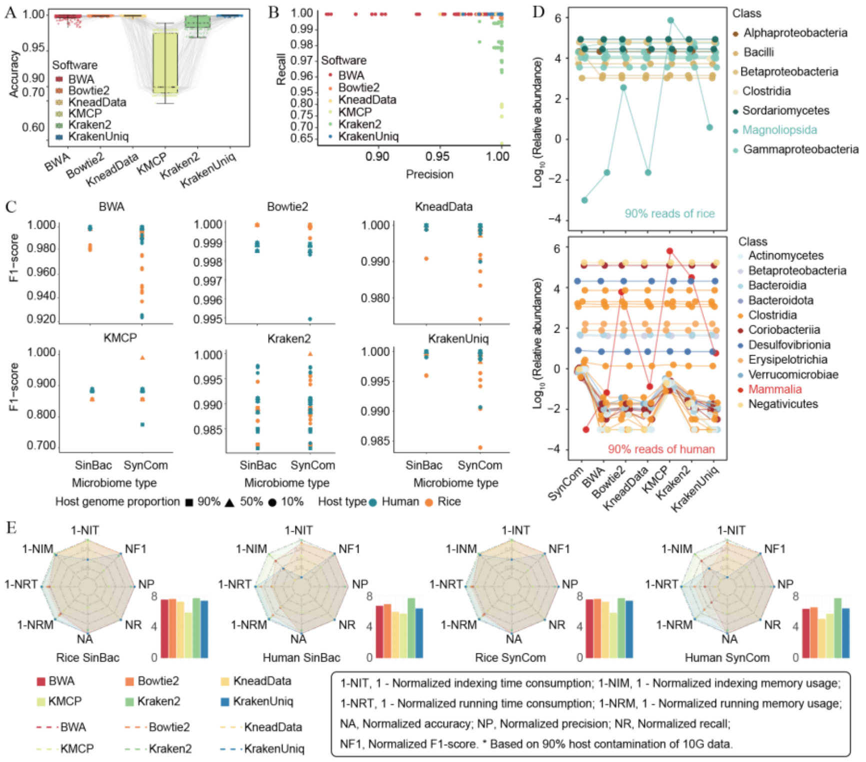

Figure 3. Assessing the accuracy of host contamination removal across various software. The alignment-based software displayed a higher rate of false positives, consequently diminishing the accuracy of microbiome information, while *k*-mer software exhibited an elevated occurrence of false negatives, thereby contributing to contamination of the data with host genome sequences. A. Accuracy among six software, showing BWA, Bowtie2, KneadData and KrakenUniq perform well. Accuracy = (True positive + True negative) / (True positive + True negative + False positive + False negative). B. Precision-recall of software. The alignment-based software (BWA, Bowtie2 and KneadData) exhibited higher false positive (some microbiota reads misaligned as host genome for removal), resulting in reduced microbiome information. However, the *k*-mer software (KMCP, Kraken2 and KrakenUniq) showed increased false negative (some host reads not be found), leading to the host genome contamination. Precision = Ture positive / (Ture positive+ False positive), Recall = True positive/ (True positive + False negative). C. High host contamination rate and microbiome complex rate reduce F1-score in six software. F1-score = 2 * Precision * Recall/ (Precision + Recall). D. Composition of the metagenomic dataset with a synthetic community after host contamination removal using six software based on 90% host contamination. BWA and KneadData retained lower host contamination in alignment-based software, and KrakenUniq and Kraken2 retained lower host contamination in *k*-mer based software. E. Comparative analysis of computational efficiency and host contamination removal performance across simulated 60 Gbps datasets with 90% host

contamination. The bar plot presented summary values for all indicators, highlighting Kraken2's excellence in comprehensive comparisons. The abbreviations for the indicators are as follows: 1-NIT, 1 - Normalized indexing time consumption; 1-NIM, 1 - Normalized indexing memory usage; 1-NRT, 1 - Normalized running time consumption; 1-NRM, 1 - Normalized running memory usage; NA, Normalized accuracy; NP, Normalized precision; NR, Normalized recall; NF1, Normalized F1-score. SinBac, Single bacterium; SynCom, Synthetic community.

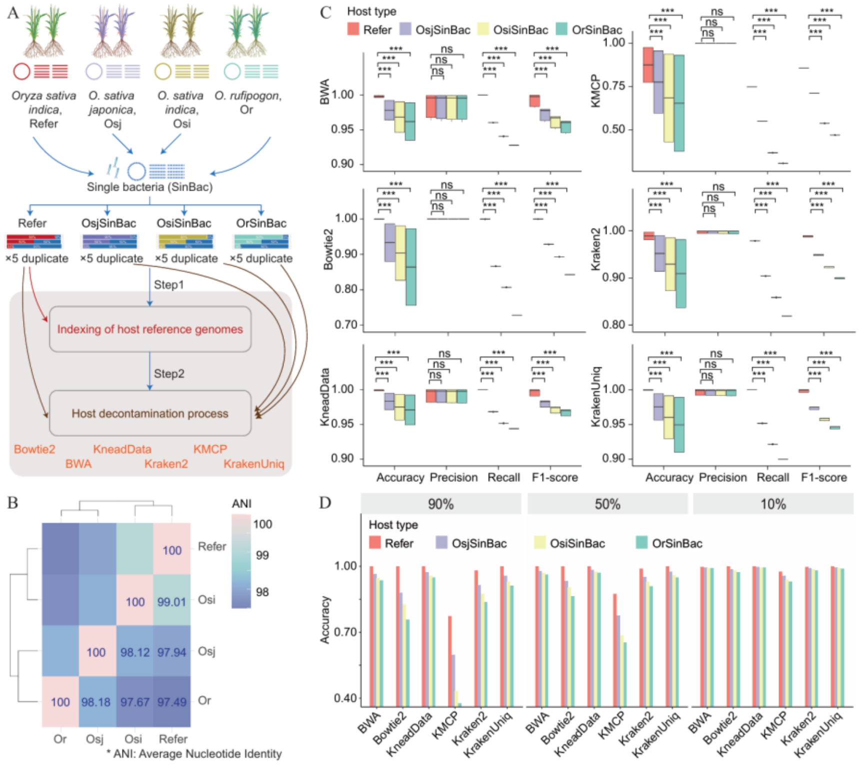

Figure 4. Impact of lacking a host reference genome on the performance of host decontamination tools

A. Impact of the absence of host reference genome on host decontamination tools. Simulated datasets were derived from three *Oryza* species (*Oryza sativa japonica*, Osj; *Oryza sativa indica*, Osi, *Oryza rufipogon*, Or) as hosts and a single bacterium. Each dataset contained varying levels of host DNA contamination (10%, 50%, and 90%) and was 10 Gbps in size, with five replicates per condition. The reference genome of *Oryza sativa indica* (refer) was used to create the indexing database for various host removal tools. All simulated data were aligned to this reference database to evaluate the performance of these tools in the absence of a specific host reference genome. B. Average nucleotide identity (ANI) analysis using FastANI. AIN values for *Oryza sativa japonica* (Osj), *Oryza sativa indica* (Osi), *Oryza rufipogon* (Or) and reference genome (*Oryza sativa indica*, refer) were shown. Osi (99.01%) showed the highest similarity to the reference genome, followed by Osj (97.94%) and Or (97.49%). C. Accuracy, precision, recall and F1-score of six tools on the simulated metagenomic data from the *Oryza* genus. All tools demonstrated significantly lower accuracy, recall, and F1-score for OsjSinBac, OsiSinBac, and OrSinBac compared to the reference data when aligned to the indexing database, which was created using the reference genome. D. Accuracy index of different software across various host genome proportion (90%, 50%, 10%). High host contamination of metagenomic data in the absence of host reference genome notably

affected the performance of existing host removal tools.

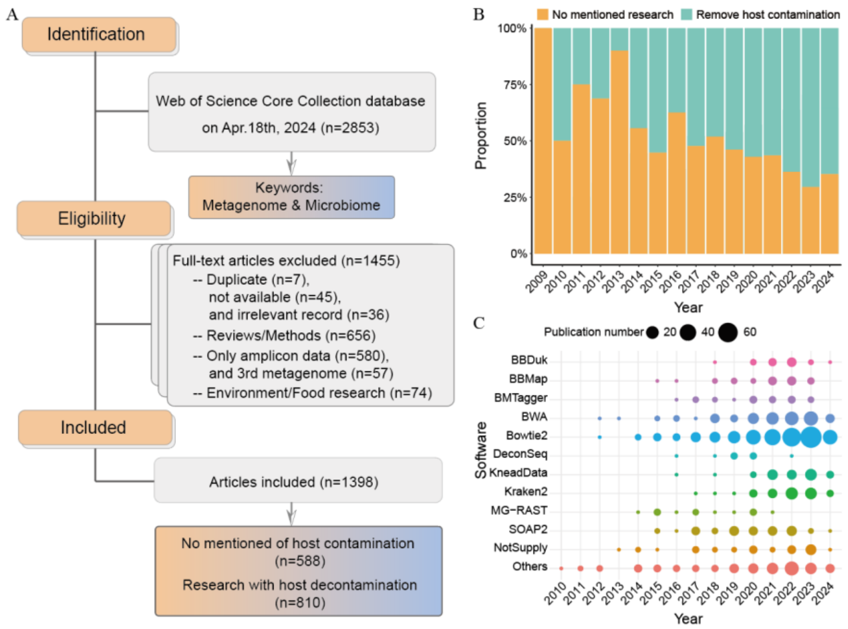

Figure S1. Literature search for existing studies addressing host contamination.

A. Literature search criteria. B. The proportion of publications mentioning the removal of host contamination. C. The existing software utilized for removing host genomes.

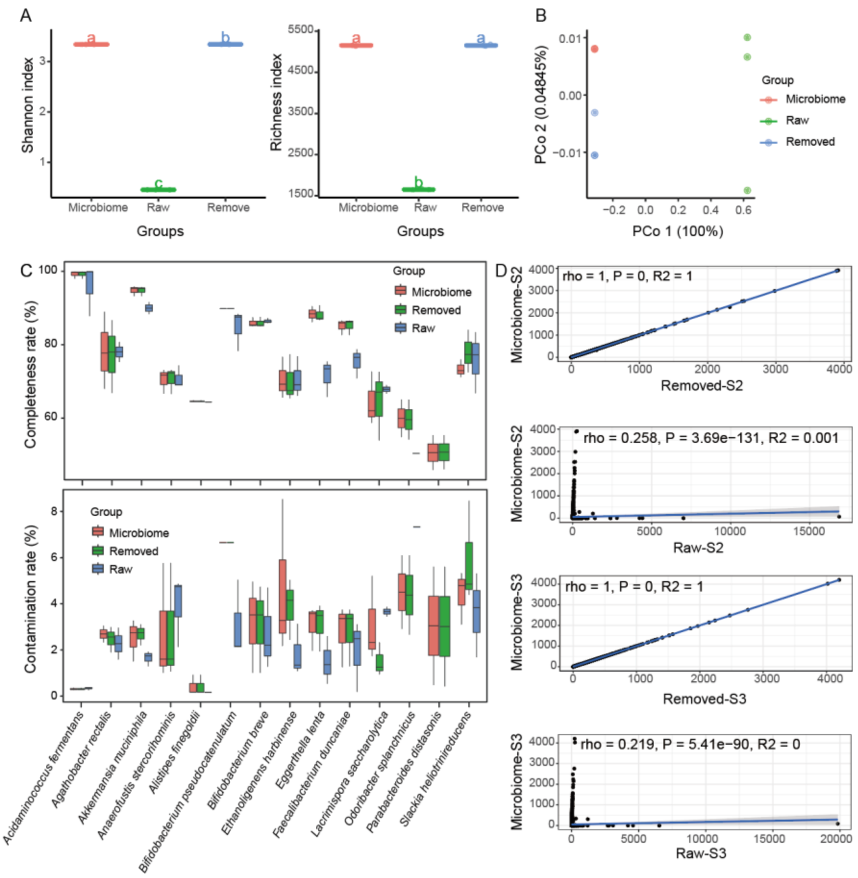

Figure S2. Comparison of host removal performance on the accuracy of the results in metagenomic analysis.

A. Shannon index, richness index, and principal coordinates analysis (PCoA) across microbiome removed and raw data. B. The completeness and contamination rates of MAGs (metagenome-assembled genomes) were assessed in microbiome, removed and raw data. C. Correlation assessment of gene ontology (GO) terms between microbiome data and removed data or raw data in S2 and S3 samples.

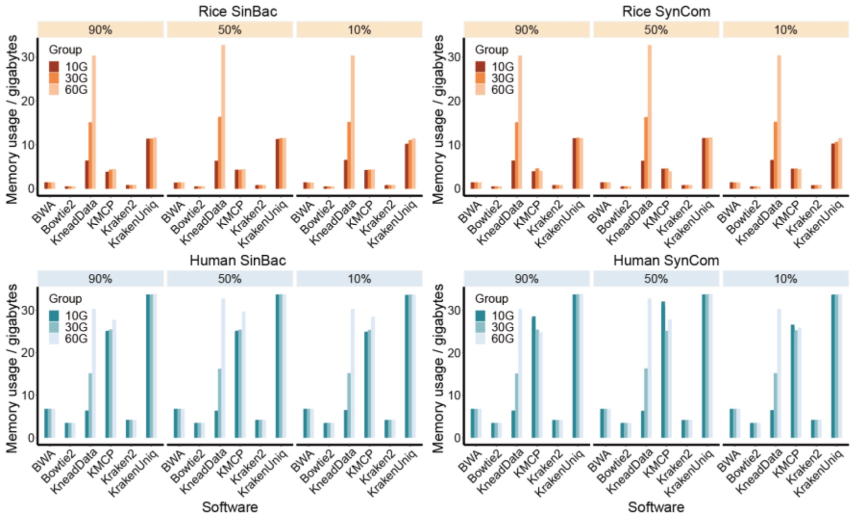

Figure S3. Memory usage during the host removing process of BWA, Bowtie2, KneadData, KMCP, Kraken2, KrakenUniq in simulation rice and human metagenome. SinBac, Single Bacteria; SynCom, Synthetic Community.

Formatted: Font: Not Bold

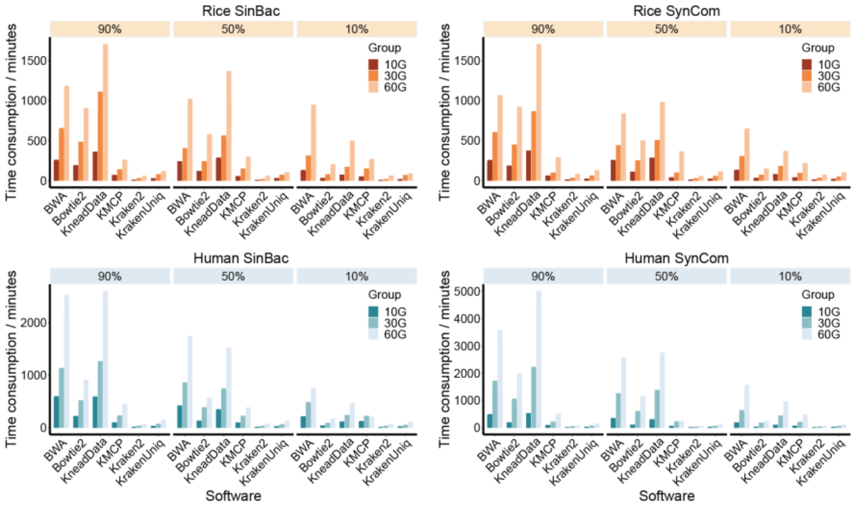

Figure S4. Time consumption during the host removing process of BWA, Bowtie2, KneadData, KMCP, Kraken2, KrakenUniq. SinBac, Single bacterium; SynCom, Synthetic community.

Formatted: Font: Bold

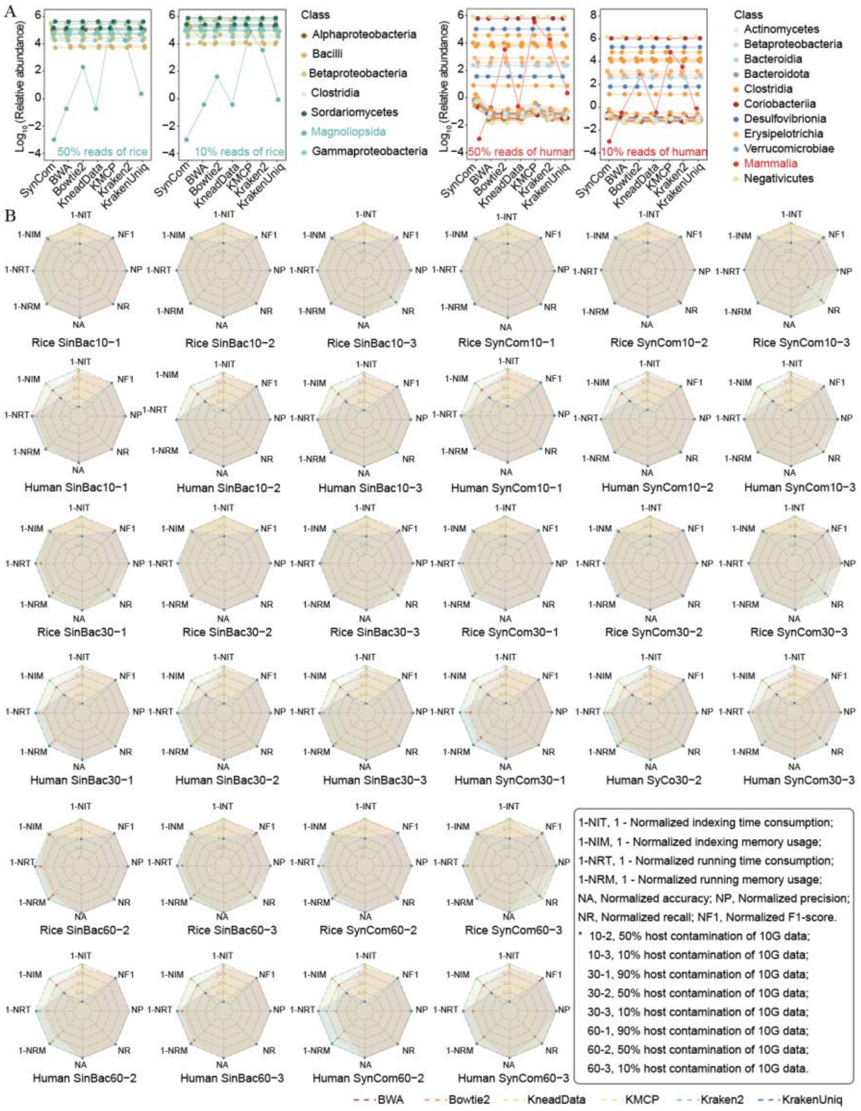

Figure S5. Comparative analysis of composition, computational efficiency and host contamination removal performance across six software.

A. Composition of the metagenomic dataset with a synthetic community after host contamination removal using six software. B. Computational efficiency and host contamination removal performance. 1-NIT, 1 - Normalized indexing time consumption; 1-NIM, 1 - Normalized indexing memory usage; 1-NRT, 1 - Normalized running time consumption; 1-NRM, 1 - Normalized running memory usage; NA, Normalized accuracy; NP, Normalized precision; NR, Normalized recall; NF1, Normalized F1-score. SinBac, Single bacterium; SynCom, Synthetic community.

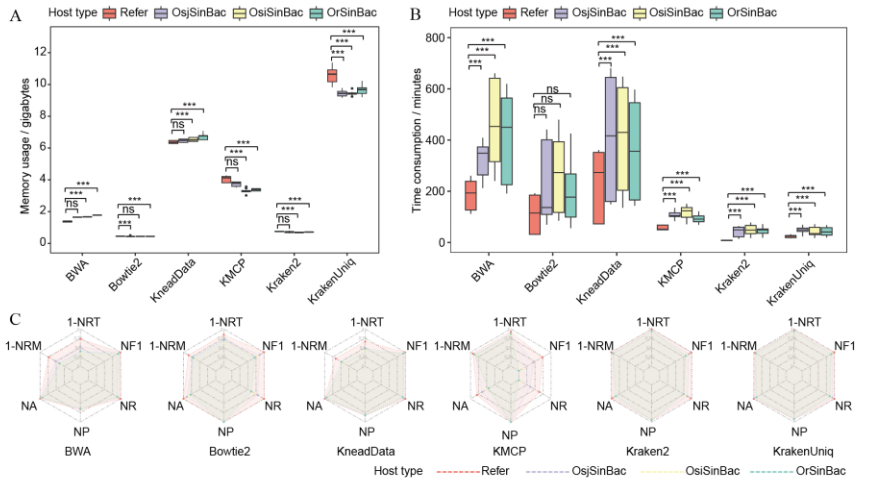

Figure S6. Assessing the performance of six tools at the genus level.

A. Memory usage during the host removing process for six tools across OsjSinBac, OsiSinBac, OrSinBac and reference metagenomic data. B. Time consumption during the host removing process for six tools across OsjSinBac, OsiSinBac, OrSinBac and reference metagenomic data. C. Computational efficiency and host contamination removal performance for six tools across OsjSinBac, OsiSinBac, OrSinBac and reference metagenomic data. 1-NRT, 1 - Normalized running time consumption; 1-NRM, 1 - Normalized running memory usage; NA, Normalized accuracy; NP, Normalized precision; NR, Normalized recall; NF1, Normalized F1-score. OsjSinBac, *Oryza sativa japonica* with a single bacterium; OsiSinBac, *Oryza sativa indica* with a single bacterium; OrSinBac, *Oryza rufipogon* with a single bacterium.

LetterToEditor&Reviewer Click here to access/download;Manuscript;LetterToEditor&Reviewer.docx

Dear Editor,

We appreciate the thorough review and constructive feedback provided by you and the reviewers. The suggestions have been instrumental in enhancing our manuscript. Here is a summary of the major suggestions and our responses:

1) Introduction & Methods:

We have expanded the introduction to include a brief description of the parameters analyzed and the rationale for selecting human and rice as hosts. Additionally, we have clarified the details regarding the synthetic community design and the simulations (Lines 300–317).

2) Discussion of Limitations:

We have added a section discussing the limitations of our analysis, especially the challenges of applying these tools to real-world data. We have also outlined future directions, including plans to incorporate real data simulations as suggested by the reviewer (Lines 414–422).

3) Terminology Standardization:

We have ensured consistent use of key terms such as *Raw*, *Removed*, *Microbiome*, and *Host Removal* across the entire manuscript. These changes aim to enhance clarity and avoid any potential confusion for the readers.

4) Research Title:

We have changed our title as 'Benchmarking short-read metagenomics tools for removing host contamination'.

5) Figures Issues:

We thoroughly reviewed all figures and addressed issues such as adding simulation line functions and correcting typographical errors.

All the detailed answer are attached below, with our responses in blue. We believe these revisions address the reviewers' concerns and significantly improve our manuscript. Your feedback has been invaluable, and we are committed to further refining our work.

Sincerely,

Yong-Xin Liu
Agricultural Genomics Institute at Shenzhen, Chinese Academy of Agricultural Sciences
liuyongxin@caas.cn
Google Scholar: https://scholar.google.com/citations?user=NoxNy_IAAAAJ

Authors response to Reviewers

Reviewer reports:

Reviewer #1: The authors present the importance of host decontamination for accurate metagenomic analyses and benchmarked six bioinformatics tools for the removal of host reads. The analyses presented is comprehensive and statistically rigorous. This would be a useful resource for other

researchers in the field of metagenomics to guide selection of appropriate tools for host read removal. Nevertheless, the introduction of the paper is missing some foundational information that makes it difficult to follow. This includes a brief introduction of the parameters that are being analyzed and the strategy for designing the synthetic communities as well as the motivation of specifically looking at human and rice as host in this analysis. The methods are lacking clarity into how the simulations were carried out and the type of sequencing technology that is being simulated. The paper also lacks discussion on limitations of the analyses, its broader implication in real data and elaboration on the biological relevance of the analysis. How would real samples be reflected/represented by the different percent of host contaminated reads as presented in this analysis. ie is 90% typical when studying skin surfaces? Given artifacts derived from sequencing, the impact of this work can be benefited by including an analysis of real-world data - ie known species spike-ins into known host background that is subsequently sequenced and compared to expected abundances.

Response: Thank you very much for your detailed and thoughtful feedback. We greatly appreciate your positive comments regarding the comprehensiveness and statistical rigor of our analysis. We have carefully considered your suggestions and have made several revisions to enhance the clarity and depth of the manuscript.

1) For the missing foundational information, we have expanded them to include more essential background information. Specifically:

- A brief description of the parameters used for synthetic community design has been added in the 'Methods' to improve clarity on the approach used. We also provide more details on how the simulations were conducted, including descriptions of the SynCom and SinBac that were simulated, to clarify this aspect of the methods (Line 309-317).
- We have explained the rationale behind selecting human and rice as hosts in our analysis (Line 300-301). Human samples are emphasized due to their critical importance in medical research, especially in the context of the human microbiome. Rice, on the other hand, serves as a key model organism in agricultural research. Additionally, both species have well-annotated and complete genomes, which meet our computational needs.

2) For the discussion of limitations and broader implication, we have added the discussion the limitations of our analysis, including the potential challenges when applying these tools to 'real-world data' (Line 414-422). We have also elaborated on the broader implications of our findings for metagenomic research. For example, in microbiome studies, contamination levels can vary significantly, and high contamination percentages are not uncommon. We greatly appreciate your suggestion regarding the inclusion of real sequencing data performance. In future work, we plan to conduct real data simulations and incorporate new algorithms to develop host removal tools suited for various scenarios. Thank you again for your valuable suggestions.

Specific comments:

- Line 58 - Consider adding that sequencing resources could also be wasted in the process of requiring deeper sequencing to capture non-host reads.

Response: Thanks for your advice, here we have addressed this by emphasizing the waste of sequencing resources caused by sequencing unwanted host DNA reads and the additional need for deeper sequencing to accurately capture non-host reads.

- Line 85 - What does "genomes from 30 microbiota: refer to? 30 microbiome samples? Or 30 microbial species?
- What is the rationale for selection of 30 genomes/species? Was this based on a specific environment? Were the abundance of each species equal?

Response: Thank you for your valuable feedback. The phrase "genomes from 30 microbiota" refers to the genomes of 30 microbial species, as detailed in Table S1-2 (Microbiome Composition). We selected these species based on the reference "*Cheng AG, Ho P-Y, Aranda-Díaz A, et al. Design, construction, and in vivo augmentation of a complex gut microbiome. Cell 2022; 19:3617-36*", where we randomly chose 30 common human-associated microbial species for simulation. Each species was represented by 0.1 million reads, resulting in a total of 3 million reads for the dataset, as described in the Methods section under "Simulated dataset description for the downstream analysis".

To avoid any confusion for the readers, we have revised the wording in the relevant sections. Specifically, in the Results section, we changed "incorporating genomes from 30 microbiota and *Homo sapiens* (GRCh38)" to "incorporating microbial reads from 30 species, each represented at equal abundance, along with human reads from *Homo sapiens* (GRCh38)". Additionally, in the Methods section, we provided the reference for the microbial community used in our simulations.

- Line 90 - what part of the metagenomic analysis? Does this refer to the classification task?
- More details can be provided here regarding the types of analyses that are being done downstream to evaluate efficacy of host decontamination, i.e. MAG assembly, functional annotation etc.

Response: Thank you for your insightful comments. We have revised the description of the metagenomic analysis to provide more details about the specific types of analyses performed to evaluate the efficacy of host decontamination, as well as clarifying what part of the analysis we are referring to.

- Line 102 - The different use of lower and uppercase to refer to the samples are confusing. Consistent use of the uppercase version "R"aw, "R"removed, "M"icrobiome would help with clarity.

Response: Thank you for your suggestion. We have standardized the terminology by consistently using uppercase for all sample references: 'Raw', 'Removed', and 'Microbiome'.

- Line 102 - More context can be given here as to why the relative abundance is "altered". It is obvious that the presence of host reads would skew the relative abundances. Would it be more informative to present the absolute counts?

Response: Thank you for your insightful suggestion. In Figure 1C, we observed that the Raw data

impacted the relative abundance due to the presence of host reads. However, after applying Kraken2's decontamination (removing Chordata), the relative abundance results aligned with those annotated for the Microbiome and Removed datasets. This is consistent with the rationale presented in our introduction, where we discussed the use of Kraken2 for host decontamination.

However, due to limitations in the reference database, the annotation results may not fully match our simulated data. Although we used the Kraken2 PlusPFP database—the most comprehensive available at 150 GB (as mentioned in the Methods section under 'Metagenomic Analysis')—we opted not to directly compare the classification data with true data to avoid potential misinterpretation by readers. Instead, we addressed this limitation in the Discussion section.

Regarding your suggestion to present absolute abundances, we found that in the Raw data, the majority of reads were classified as Chordata, but after decontamination, the remaining taxa annotations were consistent with those in the Microbiome and Removed datasets (the Table 1 was attached below). After discussion among all co-authors, we decided to retain the current presentation of relative abundance results.

Table 1. Absolute counts of the top 10 phyla.

Phylum	Microbiome data			Raw data			Remove data		
	S1	S2	S3	S1	S2	S3	S1	S2	S3
Bacillota	897000	896623	896190	897001	896623	896190	897000	896623	896190
Bacteroidota	547070	547173	547005	547070	547173	547006	547070	547173	547005
Actinomycetota	502823	502948	502800	502823	502948	502800	502823	502948	502800
Verrucomicrobiota	100065	100099	100105	100065	100099	100105	100065	100099	100105
Thermodesulfobacteriota	80667	80471	80450	80667	80471	80450	80667	80471	80450
Pseudomonadota	16747	16761	16742	16854	16761	16777	16747	16761	16742
Streptophyta	14428	14170	14492	14456	14250	14586	14428	14170	14492
Campylobacterota	1642	1642	1662	1642	1642	1662	1642	1642	1662
**Chordata**	**1343**	**1303**	**1316**	**27001112**	**27001115**	**27000944**	**2828**	**2855**	**2879**
Mycoplasmatota	736	775	765	736	775	765	736	775	765
Others	3337	3288	3246	3339	3291	3248	3337	3288	3246

- Line 104 - What does "released taxa" refer to? It seems that filtering out reads that align with Chordata achieved similar results as the Microbiome-only sample. If that is the case, what would

the benefit of host decontamination be here?

Response: Thank you for your question. By “released taxa”, we were referring to the microbial taxa that became visible after removing the Chordata (human) reads from the Raw data. This allowed us to better focus on the microbial community, resulting in similar outcomes to those observed in the Microbiome-only sample. To avoid any confusion, we have replaced “released taxa” with “remaining taxa” and added a reference to Figure 1C, de-chordata group.

Regarding the benefit of host decontamination, in the Raw data, Kraken2’s species annotation included a large amount of host information (Chordata), which affected the accuracy of microbial community profiling. In contrast, the Remove data, where host reads were filtered out using KneadData, showed a microbial community composition more similar to the Microbiome data. Although Kraken2 can be used both for species annotation and host removal, this highlights the importance of using dedicated host decontamination tools like KneadData to improve species annotation accuracy. This is also why we further evaluate different tools in the next section of the manuscript.

Additionally, due to limitations in the database of Kraken2, the annotated results differed from the actual simulated data, an issue we address in the Discussion section. We hope that by sharing our findings, we can contribute to the ongoing refinement of metagenomic analysis tools.

- Line 105 - What about the Shannon index results?

Response: Thank you for your question, and we are so sorry that we have included the Shannon index in Figure S2A, while not addressing the Shannon index results in our initial description. Our analysis showed a significant difference in the Shannon index across the Microbiome, Removed, and Raw data. The values for the Microbiome data were closely grouped (3.3397; 3.3390, 3.3402) and were higher than those for the Removed data (3.3425; 3.3419, 2.2431). In contrast, the Raw data had notably lower values (0.4549; 0.4549, 0.4565). This indicates that the removal of host reads improves the diversity estimates of the microbial community compared to the Raw data. After discussion, we decided to focus solely on the Richness index in our presentation, as it provides a clearer representation of microbial diversity and avoids potential confusion.

- Line 108 - It seems to be unusual that the first axis of the PCoA explains 100% of variation. Is this expected?

Response: Thank you for your insightful question. We acknowledge that it may seem unusual for the first axis of the Principal Coordinates Analysis (PCoA) to explain 100% of the variation. This result typically indicates that the data has low dimensionality, which can occur when the microbial community composition is dominated by a few taxa or when there is minimal variation among the samples.

In our case, this observation suggests that the removal of host reads led to a clearer distinction between the sample groups, resulting in a single axis capturing all the variability. While this is not the most common scenario in more complex datasets, it highlights the significant impact of host

decontamination on the observed microbial community structure. We appreciate your attention to this detail and will clarify this point in the manuscript to provide better context for our findings.

- Line 115 - There seems to be no further discussion about the recovery of B. breve from only the raw samples. Any hypotheses of why this could have been the case?

Response: Thank you for your question. To assess the recovery of *B. breve*, we compared the MAGs obtained from our analysis with the simulated *Bifidobacterium breve* data (GCF_001025175.1). The ANI similarity between the MAGs and the reference genome ranged from 99.8% to 99.9%. In our manuscript, we used MetaWRAP refinement to improve the quality of our MAGs binning, applying the parameters -c 50 -x 10, meaning we retained only bins with completeness over 50% and contamination below 10% (Table 2, MAGs in blue).

However, when we kept all binning data and adjusted the MetaWRAP refinement parameters to -c 0 -x 100, we indeed recovered additional low-quality MAGs (Table 2, MAGs in green). With this adjustment, *B. breve* was enriched in a greater number of Microbiome and Remove data, displaying a completeness of 85.42% to 85.89% and contamination of 12.91% to 14.46%. Additionally, we examined all enriched data and found no clear patterns, except that host removal generally improves the recovery of high- and medium-quality MAGs, though some specific groups may still be enriched in raw data.

To provide readers with more information, we have added our MAG filtering parameters to the Materials and Methods section and included this discussion in the discussion section.

Table2. Reconstruetd MAGs of *B. breve*.

Samples	fastANI	completeness	contamination	GC	N50	size	MAG quality
Microbiome1	99.45	85.42	13.6	0.587	15788	1898963	Low
Microbiome2	99.8024	94.81	8.156	0.587	18589	2083715	Medium
Microbiome3	99.6876	85.89	12.91	0.588	18766	1967196	Low
Remove1	99.4102	85.42	14.46	0.587	15444	1973302	Low
Remove2	99.8168	94.81	8.156	0.587	18589	2083715	Medium
Remove3	99.6882	85.89	12.91	0.588	18766	1965172	Low
Raw1	99.8909	83.18	1.109	0.588	16512	1695982	Medium
Raw2	99.8911	93.28	5.66	0.589	19278	2050916	Medium
Raw3	99.843	90.34	2.707	0.589	19070	1910714	High

* fastANI represents the comparison between reconstructed MAGs of different samples and reference genome (Bifidobacterium breve fasta data (GCF_001025175.1)).

High MAGs, completeness ≥ 90% and Contamination ≤ 5%; Medium MAGs, completeness ≥ 50% and Contamination < 10%; Low MAGs, completeness < 50% or Contamination > 10%

- Line 118 - In what aspect does “higher similarity” refer to here? What are the numbers in the x and y axis of Fig. 1E?

Response: Thank you for your insightful question. The term ‘higher similarity’ refers to the

Spearman correlation between the GO terms in different datasets. Specifically, we compared the GO term annotations between Microbiome data, Raw data, and Removed data. In Figure 1E, the x-axis represents the GO term counts in the Microbiome data, while the y-axis represents the GO term counts in the Raw and Removed data, respectively. To make this clearer, we have now added linear regression lines to the plot for better visualization of the relationship. And we have replaced the phrase 'higher similarity' with 'stronger correlation' in our manuscript to avoid any potential confusion.

- Line 124 - how many species are included in the SynCom and were the communities designed to reflect either a human-associated microbiome and a rice microbiome? This is later mentioned in the methods but stating in here could help with interpretability. Furthermore, what is the rationale for simulating equal abundances of each species?

Response: We apologize for not providing sufficient details regarding the SynCom. In our study, the SynCom included 14 species for the rice microbiome and 35 species for the human-associated microbiome. The simulated data were based on core microbiota reported in the literature (see Table S2-1). For further clarification, we have included a detailed description in the Methods section and noted in Line 124 that readers can find additional information there.

Regarding the rationale for simulating equal abundances of each species, we initially employed the differential mode from the CAMISIM software, which captures a greater degree of randomness and reflects natural samples more closely. However, in the first part of our results, we chose to use equal abundances to emphasize the impact of host decontamination on the recoverable microbial MAGs. In other parts of the manuscript, we utilized a differential approach that better represents natural sample dynamics.

- Line 130 - The description of the abbreviations are somewhat confusing. Is SinBac 60-3 supposed to be SynBac 60-3 here? In Fig. 2A the bar graphs in the center showing the proportions of host vs bacteria reads does not seem to be consistent with the vertical bar graphs in the same plot.

Response: Thank you so much for pointing out this issue. Here, it should be 'SynCom 60-3' instead of 'SinBac 60-3'. We have reviewed the entire manuscript for consistency and made the necessary corrections. Additionally, we have updated Figure 2A to ensure that the proportions of host versus bacterial reads in the bar graphs are consistent with the vertical bar graphs in the same plot.

- Line 182 - What were some of the false positives/ false negatives? Were these specific species that are consistently misclassified? What taxonomic levels were the analysis caried out at? Do these metrics differ at different taxonomic levels?

Response: Thank you for your question. In our study, we evaluated the effectiveness of host read removal using various software tools. True positives refer to host reads that were correctly identified and removed as host genome sequences. False positives represent microbiota reads that were mistakenly classified as host reads and thus removed, while false negatives refer to host reads that

were not identified and remained in the dataset.

Our analysis was conducted at the read level, as each simulated metagenomic read was labeled with its specific origin. Therefore, we did not focus on identifying consistently misclassified species at different taxonomic levels, since the classification was based on individual reads rather than specific taxa. However, the potential for misclassification of certain reads is an area of interest, and in future work, we plan to conduct a more detailed analysis of these misclassified reads to better understand patterns in distinguishing between host and microbial sequences more accurately.

- Lines 195-198 - If KrakenUniq also displays low Mammalia values, shouldn't it be considered as being able to remove more host contamination reads rather than less?

Response: Thank you for pointing this out. We appreciate your careful observation. What we intended to express here is that, among the three tools, BWA and KneadData demonstrate better host read removal performance compared to KrakenUniq. We have revised the original text to clarify this point.

- Line 212 - How does different versions of references genomes impact host removal? (ie GRCh38, telomere-to-telomere gapless sequence of the human genome). Can a similar evaluation be carried out to assess the impacts of these versions?

Response: Thanks so much for your suggestion, we acknowledge that different versions of reference genomes may have varying impacts on host removal efficiency. In our future work, we plan to evaluate these differences.

- Line 231 - Does "the absence of a host reference genome" mean absence of a host reference genome that is closely aligned with the host whereby samples/sequences were derived from? If yes, this might have to be reworded for clarity.

- It is not always possible to obtain host genomes that are specific to a given sample type - are there recommendations for what can be done to select a suitable host genome?

Response: We appreciate your suggestion. We have revised our wording to clarify that 'the absence of a host reference genome' now reads as 'the absence of a closely aligned and accurate reference genome'. Additionally, we have thoroughly reviewed and updated the relevant descriptions throughout the manuscript. Regarding the selection of a suitable host genome, our experience suggests that using a pangenome approach during database construction can be effective. We plan to validate this approach in our upcoming research. Additionally, we are exploring the integration of deep learning algorithms to address the challenges posed by the absence of precise reference genomes.

- Line 245 - How were the simulations carried out in CAMISIM? What were the simulation parameters?

Response: Thank you for your question. We have expanded the Methods section to provide a clearer

understanding of how the simulations were conducted in CAMISIM. This includes a detailed description of the simulation parameters used, ensuring readers can better grasp how we obtained the simulated data.

- Lines 299-304 - These text appears to be repeated from the results section above (line 122-127).

Response: Thank you for pointing this out. Our initial intention was to maintain consistency to avoid confusion for readers. We have now streamlined the description in lines 122-127 to allow readers to focus more on the results section.

Please correct all spelling mistakes in the text, figures and figure legends.

For example:

- Figure 1A 0.1million reads/species, Diversity analysis, Memory usage
- Line 367 - 27 million "reads"

Line 369 - "equal"

Response: Thank you so much for your thorough review. We have carefully checked and revised our entire manuscript.

Reviewer #2: Overall Impressions

The authors present a nice comparison of different methods for removing host data from metagenomic sequencing data. As this focuses entirely on short-read metagenomic data, I suggest altering the title to communicate this.

Response: Thank you for your suggestion. We have changed our title as 'Benchmarking short-read metagenomics tools for removing host contamination'.

Major Issues

Can the authors comment on whether a two stage-approach would be beneficial for data from hosts with a good-quality reference genome (eg. human or mouse). I have noticed in my own data that, after decontamination by Bowtie2 alignment against the human reference genome, some reads are still classified by Kraken2 as Homo sapiens - which I then remove. Would implementing this as standard be beneficial or offer only a marginal increase in data quality in return for the additional processing time and resources. The authors highlight that Kraken2 seems to be suitable for identifying which reads are from Homo sapiens (L104, Fig 1C) - they could use this info to add another decontamination method to the evaluation (Kraken2 classification of Raw data followed by extracting non-chordata reads using the extract_kraken_reads.py script in KrakenTools).

Response: Thank you for your insightful question. In metagenomic studies, we often work with data from numerous samples, while accurate reference genomes for each host (human) are hardly

available. Thus, we typically construct a reference index based on a publicly available host genome and perform host removal analyses using tools like Bowtie2 or KneadData. And it is also common for Kraken2 to annotate additional host reads. Based on our team's experience, we also directly remove these extra host reads use the extract_kraken_reads.py script.

The issue of residual host contamination due to the lack of an accurate reference genome is evident in our data. For example, when using Bowtie2 with a precise host reference genome, we achieve data accuracies ranging from 0.9973 to 0.9999 (Figure 3A). In contrast, when a precise reference genome is absent, the accuracy of our data drops to a range of 0.7552 to 0.9999 (Figure 4C).

Regarding the potential benefits of a two-step approach for host contamination removal, we strongly support this method. Host contamination can significantly affect the accuracy of downstream functional annotations (Figure 1E). In our original manuscript, we discussed various host removal strategies that balance speed and accuracy, and we developed a workflow called HostPurge (https://github.com/HaoLuo-leo/HostPurge). In our future work, we will further optimize the HostPurge workflow to provide a precise and efficient solution for host contamination removal.

Why was the number of reads chosen for the microbial species included? The authors focus on the reconstruction of MAGs and note that some species were not recovered in some groups - it is likely that increasing the number of reads overall, without adjusting the % of reads from microbiome and host, will impact this.

Response: Thank you for your question. In our study, we aimed to assess the impact of host reads on the generation of Metagenome-Assembled Genomes (MAGs) from microbial communities, particularly in samples with high host proportions (such as 90%). Typically, metagenomic datasets are around 10 Gb in size. If we were to increase the number of reads from the microbial species, the overall sequencing coverage would also need to be increased, which would significantly raise the costs associated with sequencing and computational analysis.

Our focus was to investigate the effects of high host read proportions on the recoverability of MAGs under standard sequencing conditions. In our simulations, the 30 million reads generated approximately 8 Gb of data, which aligns with the typical sequencing output in routine studies. This design allows us to explore the dynamics of host contamination in a realistic context while keeping the analysis feasible.

Were the MAGs that were not recovered from species with larger genomes? The same number of reads from a larger genome will likely return a smaller MAGs due to a lower breath and depth of coverage. Also, can the authors state why MAGs were reconstructed in the first place? Was it for data quality purposes?

Response: Thank you for your question. The quality of the data is indeed crucial for recovering high-quality MAGs, which serve as a foundation for further investigation into the functionality, metabolism, and evolution of specific species or strains. Reconstructing MAGs is a vital step in metagenomic data analysis, as it enables functional validation of microbial species or strains

identified in the sample.

In our study, each microbial species was represented by 0.1 million reads, with genome sizes ranging from approximately 2 to 6 Mb (Table S1-2, S2-1). This resulted in a coverage depth of 5~15× for each genome. To determine whether insufficient coverage might explain the failure to reconstruct certain MAGs, we checked the genomes of reconstructed and unreconstructed MAGs. Here two reconstructed MAGs did not match our reference data, but we did not observe a strong correlation between genome size and the ability to recover MAGs (Table 1).

In this work, we used MetaWRAP refinement to improve the binning of our MAGs with parameters -c 50 -x 10, meaning we retained only those bins with completeness greater than 50% and contamination less than 10%. However, when we retained all binning data and adjusted the MetaWRAP refinement parameters to -c 0 -x 100, we indeed recovered additional low-quality MAGs, while we still did not see the strong correlation

We hypothesize that, in addition to genome size, factors such as the heterogeneity of the simulated data and the presence of specific bacterial genes might influence both the quantity or quality of the MAGs that can be recovered.

Table 1. The genome size of simulated microbiota.

Species	Genome_Size	Reconstructed MAGs this work	Adjusted parameter (-c 0 -x 100)
Veillonella_dispar	2.1Mb	No	No
Absiella_dolichum	2.2Mb	No	No
Ruminococcus_bromii	2.2Mb	No	No
Acidaminococcus_fermentans	2.3Mb	**Yes**	**Yes**
Anaerofustis_stercorihominis	2.3Mb	**Yes**	**Yes**
Collinsella_aerofaciens	2.3Mb	No	**Yes**
Bifidobacterium_breve	2.3Mb	**Yes**	**Yes**
Bifidobacterium_pseudocatenulatum	2.3Mb	**Yes**	**Yes**
Holdemanella_biformis	2.5Mb	No	**Yes**
Burkholderiales_bacterium	2.6Mb	No	No
Akkermansia_muciniphila	2.7 Mb	**Yes**	**Yes**
Desulfovibrio_piger	2.9Mb	No	No
Adlercreutzia_equolifaciens	2.9Mb	No	No
Ethanoligenens_harbinense	3.0Mb	**Yes**	**Yes**
Slackia_heliotrinireducens	3.1Mb	**Yes**	**Yes**
Clostridiales_bacterium	3.2Mb	No	No
Anaerobutyricum_hallii	3.3Mb	No	No
Eubacterium_rectale	3.4 Mb	No	No
Clostridium_methylpentosum	3.5Mb	No	No
Eggerthella_lenta	3.6Mb	**Yes**	**Yes**

Holdemania_filiformis	3.6Mb	No	No
Alistipes_finegoldii	3.7Mb	**Yes**	**Yes**
Clostridium_hylemonae	3.8Mb	No	No
Roseburia_inulinivorans	4.2Mb	No	No
Bacteroides_coprocola	4.3Mb	No	No
Odoribacter_splanchnicus	4.4Mb	**Yes**	**Yes**
Lacrimispora_saccharolytica	4.6Mb	**Yes**	**Yes**
Parabacteroides_distasonis	4.7 Mb	**Yes**	**Yes**
Phocaeicola_vulgatus	5.2 Mb	No	No
Bacteroides_xylanisolvens	6.0Mb	No	No

Kneaddata uses Bowtie2 to do the alignments, what is the difference between the Bwotie2 approach presented here and the alignment step performed by Bowtie2 in Kneaddata?

Response: Thank you for your question. In our software evaluation, we used the default or recommended parameters for each tool. While both Bowtie2 and KneadData use the Bowtie2 alignment algorithm, there are differences in how they handle host contamination removal.

For example, KneadData is an integrated pipeline that automates various preprocessing steps for metagenomic data, including quality control (such as trimming low-quality reads) and decontamination (aligning reads to a host genome). In our evaluation, we used the --bypass-trim and --bypass-trf options to skip these preprocessing steps, but our results showed that the outcomes of KneadData and Bowtie2 were not completely identical. Upon further investigation, we found that the default parameters differ between the two: Bowtie2 uses the --sensitive-local setting [-D 15 -R 2 -N 0 -L 22 -i S,1,1.15], while KneadData applies the --very-sensitive-local setting [-D 20 -R 3 -N 0 -L 20 -i S,1,0.5]. This could be one of the reasons for the observed differences. Therefore, we still presented their differences in our work to highlight their variation.

As we have already discussed the parameter settings for different software in the background section, we do not delve into a detailed comparison of parameters in the main body of the manuscript.

Minor Issues

Throughout: the authors switch between the terms "host-depleted" and "Remove" to refer to the data which has been decontaminated. I suggest sticking to one, the former is more descriptive and tends to work better in context, but it is up to the authors.

Response: Thank you for your suggestion. Apart from the section where we compare Raw data, Remove data, and Microbiome data, we have standardized the term ‘host-removal’ throughout the rest of the manuscript to ensure consistency and improve readability for the readers.

L49: I realise this is not the aim of the manuscript, but could the authors briefly mention the different methods that exist for microbial DNA enrichment (or host DNA depletion) before library preparation and sequencing.

Response: We appreciate your suggestion. A postdoc of our team, who specializes in this area, has written a review on microbial DNA enrichment and host DNA depletion methods during experiments. This review is currently being prepared for submission and we hope it will provide valuable insights to the field on this topic.

L51-52: I agree that this is a concern. Can the authors provide a reference to any manuscripts which have evaluated this? If they exist. It would be important context for the reader and might also address my previous point.

Response: Thank you for your insightful question. As mentioned earlier, the review will also address this concern, summarizing the different methods and their evaluations, which can provide important context for the reader.

L63: can the authors hypothesise about a particular reason for the increase began in 2015 - is this due to a paper or commentary highlighting the importance? Or is this an artefact of the increased quantity of microbiome papers published over this period?

Response: Thank you for your question. It's hard to pinpoint a specific reason for the increase beginning in 2015. Our survey indicates that the number of research articles employing metagenomic analysis (excluding amplicon studies) gradually increased from 2012 to 2014. However, we cannot definitively say whether this rise correlates with specific events, such as the release of Kraken in 2014.

Moreover, in our opinion, it's possible that authors may have employed these steps but chose not to include details in their descriptions. While we apologize for not being able to provide more specific information, it is clear that an increasing number of articles are beginning to recognize the importance of detailing host removal procedures.

L130: I think the description is incorrect here. The even split would be SinBac 60-2, while the 90% host and 10% community would be SynCom 60-1?

Response: Thank you so much for pointing out this issue. Here, it should be 'SynCom 60-3' instead of 'SinBac 60-3'. We have reviewed the entire manuscript for consistency and made the necessary corrections.

L182- L188 - can the authors please comment on a) whether there were specific microbial species, genes, or sets thereof which were commonly erroneously identified as host, and b) were these erroneously unmapped reads subsequently assigned taxonomy by Kraken2. These are important considerations as they may impact the downstream microbiome profile.

Response: Thank you very much for your insightful question. Indeed, we encountered instances in our study where some reads were detected by Kraken2 after being filtered for host contamination using Bowtie2 or KneadData. We believe this discrepancy arises from the differences between alignment-based algorithms and *k*-mer algorithms, as well as our inability to provide precise host

reference genomes.

As for whether specific microbial species, genes, or sets of reads were commonly misidentified as host, we do not have definitive conclusions at this time. However, identifying these specific data types will be a key focus of our future work. We appreciate your valuable suggestions.

Discussion: generally, journals prefer that you do not refer to Figures and Tables in the discussion section. They should already be introduced in the results and methods section. Check that this is allowed by Gigascience.

Response: Thank you for your advice. We checked the formatting guidelines of *GigaScience*, and while we did not find specific requirements regarding the referencing of figures and tables in the discussion section, we all agree that your suggestion is reasonable. Therefore, we have removed the relevant references from the discussion. Thank you for your helpful feedback.

L369: "equal"

Response: We appreciate your feedback and have made the necessary changes.

L387-388: This could be tested by including reference genomes from other genera in the same family, if available.

Response: Thank you very much for your suggestion. We plan to implement this work in the future development of the HostPurge pipeline https://github.com/HaoLuo-leo/HostPurge. In our upcoming research, we will investigate whether our software performs better with reference genomes from the same genera or family, or even in cases where no reference genomes are available.

Figure1

Click here to access/download;Figure;Figure1-F.pdf

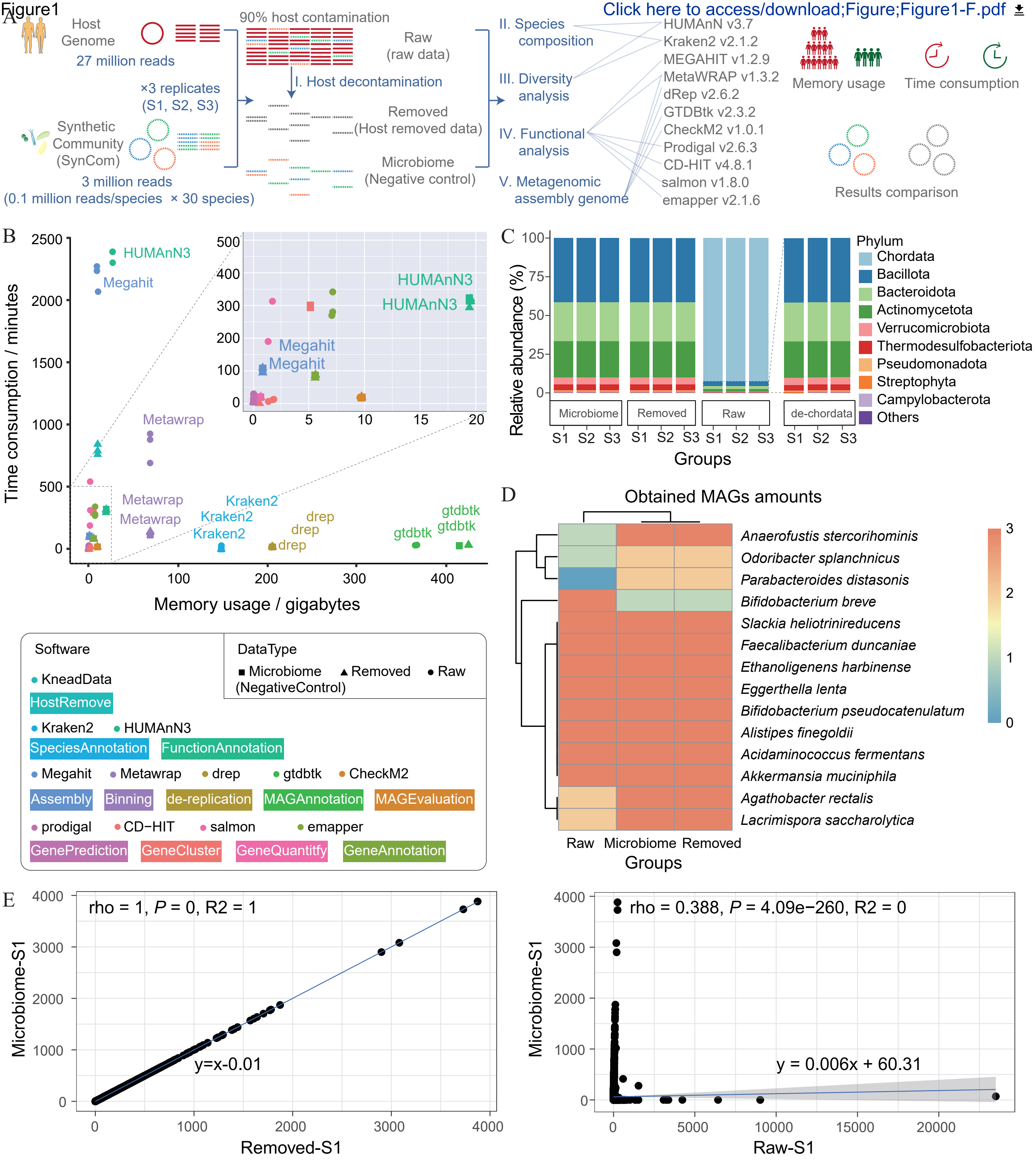

Click here to access/download;Figure;Figure2-F.pdf

Figure2

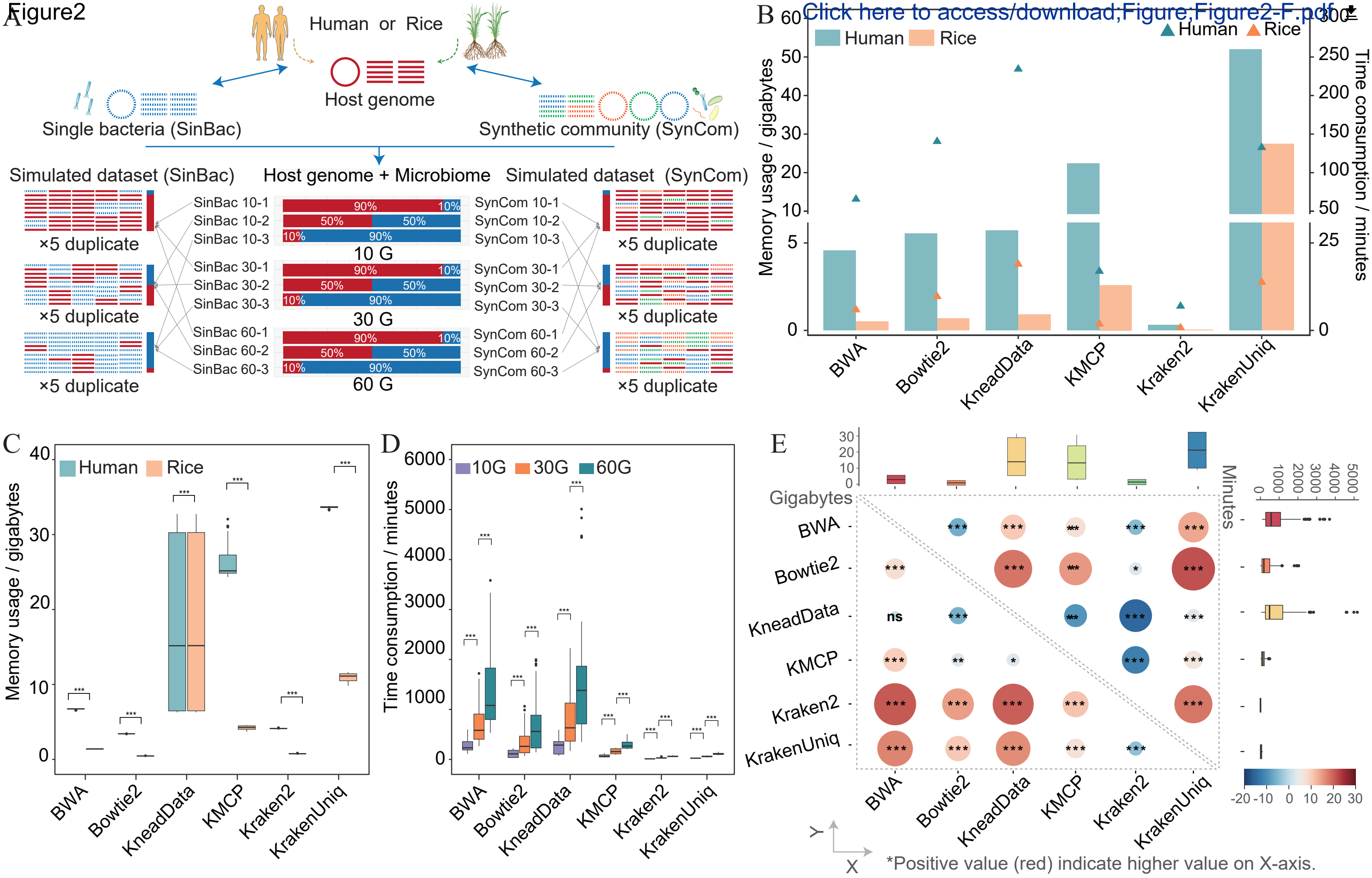

Figure3

Click here to access/download;Figure;Figure3.pdf

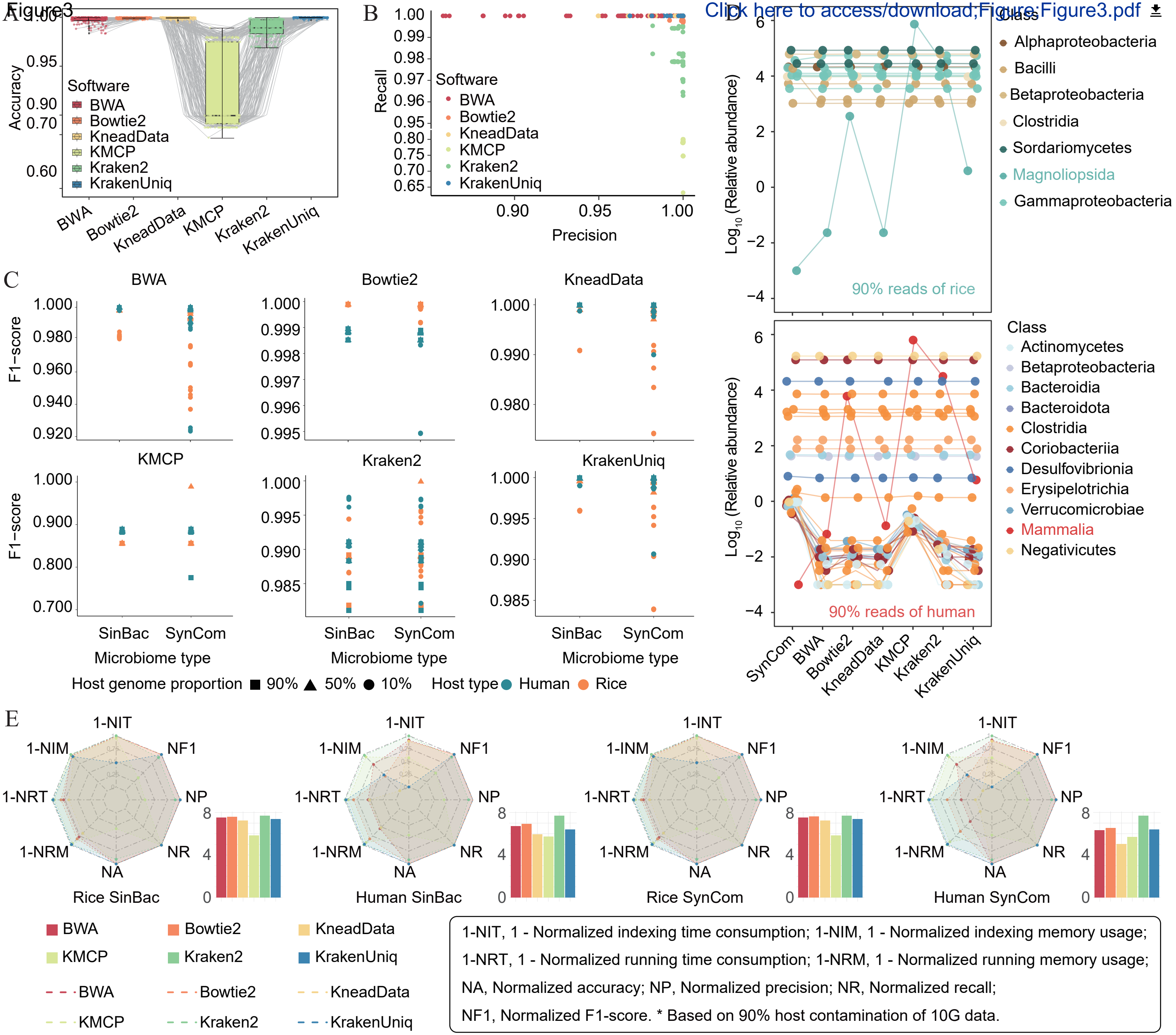

Figure4

Click here to access/download;Figure;Figure4.pdf

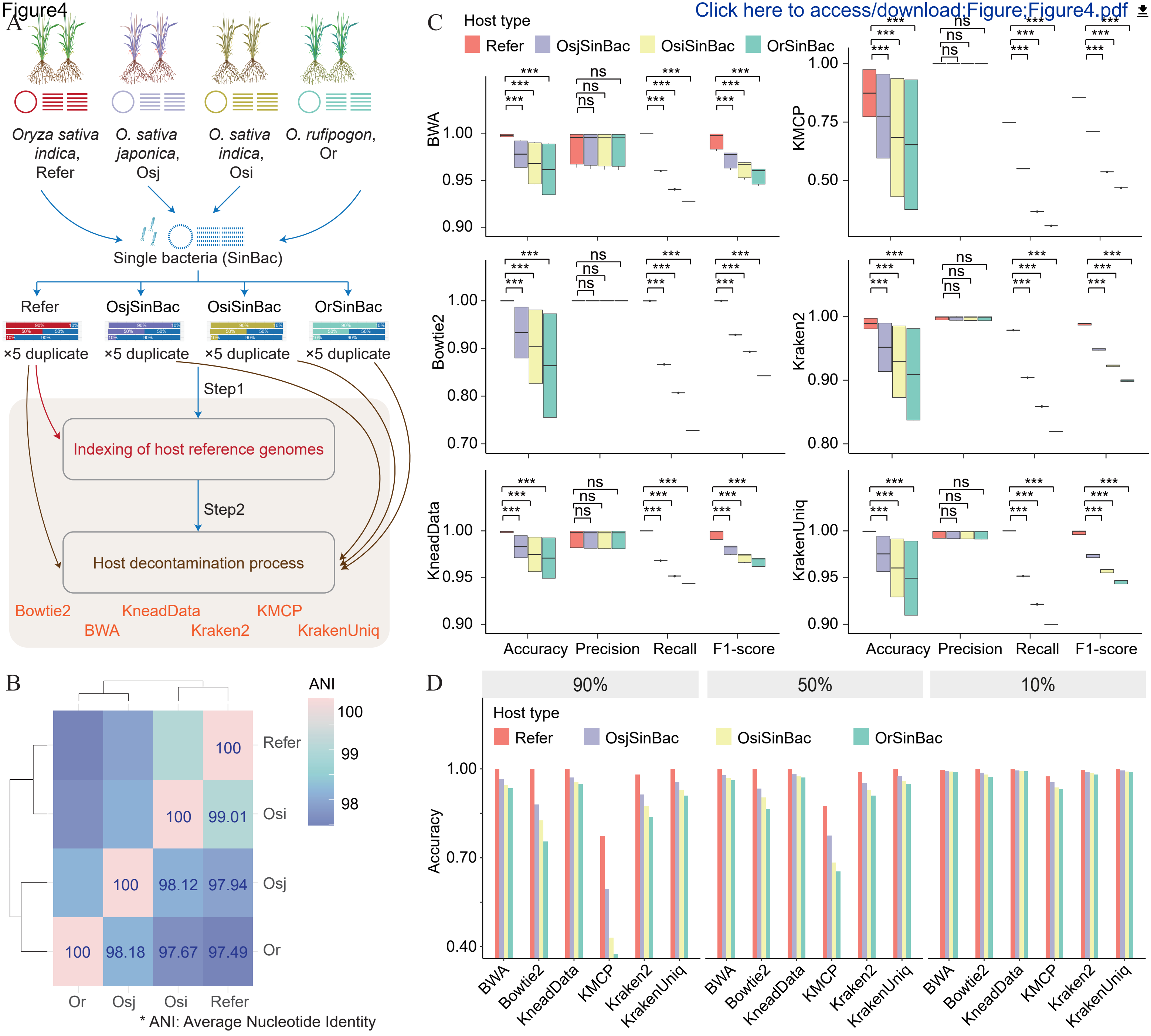

Supplementary tables

Click here to access/download
Supplementary Material
SupplymentaryTables.xlsx

FigureS1

Click here to access/download
Supplementary Material
FigureS1.pdf

FigureS3

Click here to access/download
Supplementary Material
FigureS3.pdf

FigureS4

Click here to access/download
Supplementary Material
FigureS4.pdf

FigureS5

Click here to access/download

Supplementary Material

FigureS5.pdf

FigureS6

Click here to access/download
Supplementary Material
FigureS6.pdf

FigureS2

Click here to access/download
Supplementary Material
FigureS2-F.pdf

GA

Click here to access/download;Graphical Abstract;GA.pdf

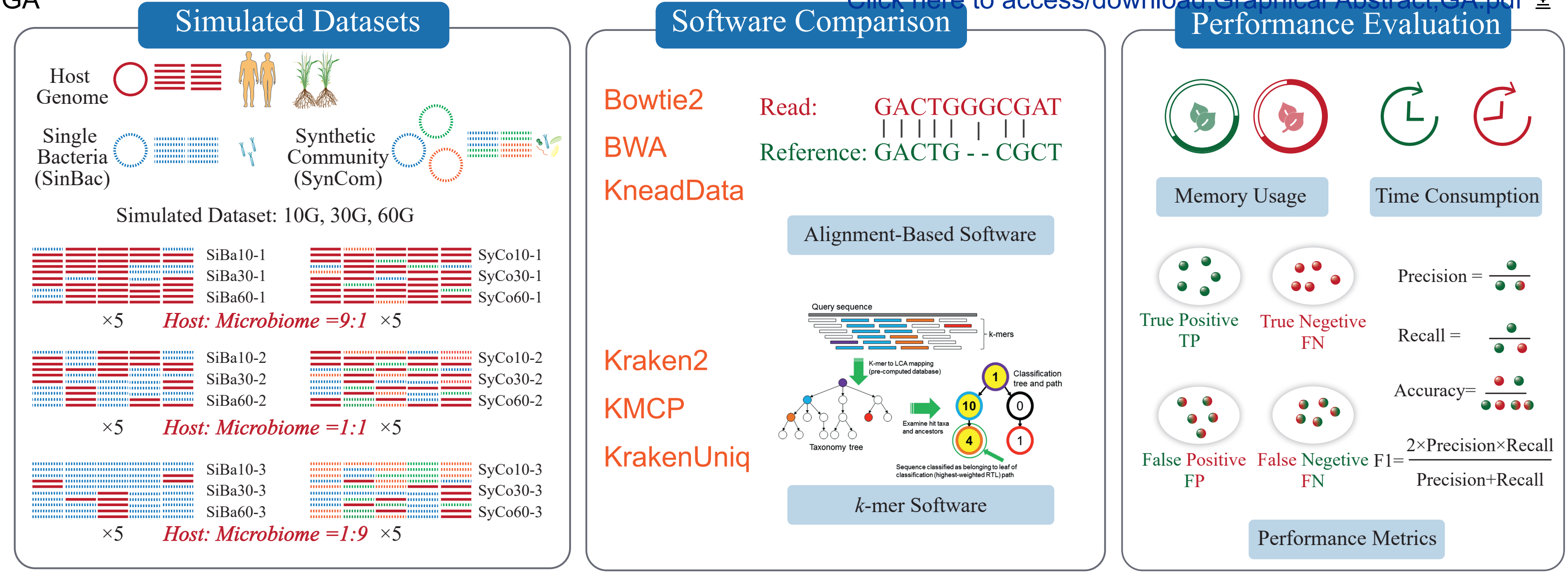

Supplement: giaf004_GIGA-D-24-00318_Revision_1 [file giaf004_giga-d-24-00318_revision_1.pdf]
