# Supplementary material for: Benchmarking short-read metagenomics tools for removing host contamination
Source: Gigascience. 2025 Feb 27;14:giaf004. doi: 10.1093/gigascience/giaf004 (PMC11878760; doi:10.1093/gigascience/giaf004)
Supplement: giaf004_Supplemental_Files [file FigureS5.pdf › FigureS5.pdf]

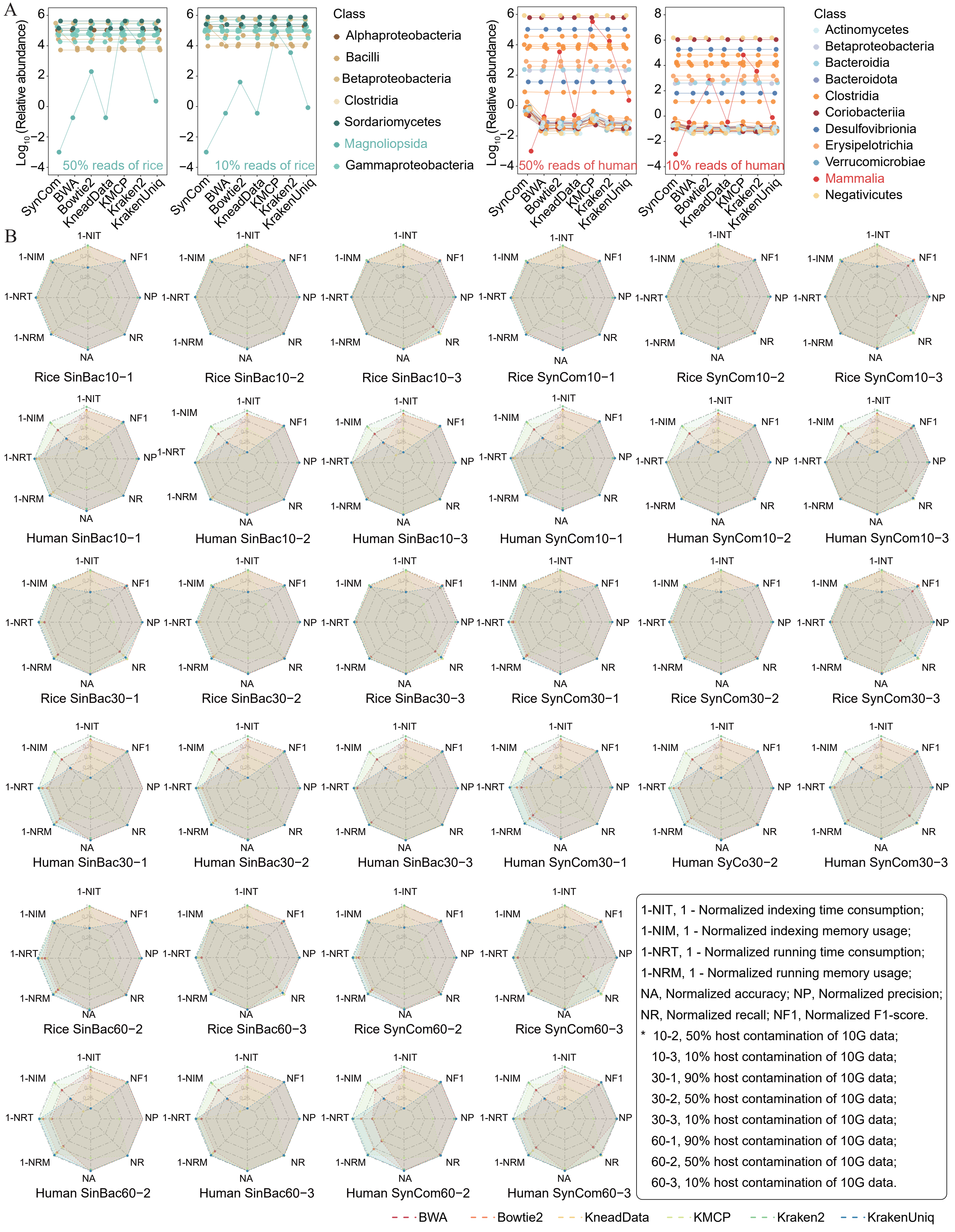

A
Log10(Relative abundance)
50% reads of rice
10% reads of rice
SynCom
BWA
Bowtie2
KneadData
KMCP
Kraken2
KrakenUniq
Class
Alphaproteobacteria
Bacilli
Betaproteobacteria
Clostridia
Sordariomycetes
Magnoliopsida
Gammaproteobacteria
50% reads of human
10% reads of human
Class
Actinomycetes
Betaproteobacteria
Bacteroidia
Bacteroidota
Clostridia
Coriobacteriia
Desulfovibrionia
Erysipelotrichia
Verrucomicrobiae
Mammalia
Negativicutes
B
Rice SinBac10−1
Rice SinBac10−2
Rice SinBac10−3
Rice SynCom10−1
Rice SynCom10−2
Rice SynCom10−3
Human SinBac10−1
Human SinBac10−2
Human SinBac10−3
Human SynCom10−1
Human SynCom10−2
Human SynCom10−3
Rice SinBac30−1
Rice SinBac30−2
Rice SinBac30−3
Rice SynCom30−1
Rice SynCom30−2
Rice SynCom30−3
Human SinBac30−1
Human SinBac30−2
Human SinBac30−3
Human SynCom30−1
Human SyCo30−2
Human SynCom30−3
Rice SinBac60−2
Rice SinBac60−3
Rice SynCom60−2
Rice SynCom60−3
Human SinBac60−2
Human SinBac60−3
Human SynCom60−2
Human SynCom60−3
1-NIT, 1 - Normalized indexing time consumption;
1-NIM, 1 - Normalized indexing memory usage;
1-NRT, 1 - Normalized running time consumption;
1-NRM, 1 - Normalized running memory usage;
NA, Normalized accuracy; NP, Normalized precision;
NR, Normalized recall; NF1, Normalized F1-score.
* 10-2, 50% host contamination of 10G data;
10-3, 10% host contamination of 10G data;
30-1, 90% host contamination of 10G data;
30-2, 50% host contamination of 10G data;
30-3, 10% host contamination of 10G data;
60-1, 90% host contamination of 10G data;
60-2, 50% host contamination of 10G data;
60-3, 10% host contamination of 10G data.
BWA
Bowtie2
KneadData
KMCP
Kraken2
KrakenUniq
