# Supplementary material for: Benchmarking short-read metagenomics tools for removing host contamination
Source: Gigascience. 2025 Feb 27;14:giaf004. doi: 10.1093/gigascience/giaf004 (PMC11878760; doi:10.1093/gigascience/giaf004)
Supplement: giaf004_Response_to_Reviewer_Comments_Original_Submission [file giaf004_response_to_reviewer_comments_original_submission.pdf]

Author's Response To Reviewer Comments

Dear Editor,

We appreciate the thorough review and constructive feedback provided by you and the reviewers. The suggestions have been instrumental in enhancing our manuscript. Here is a summary of the major suggestions and our responses:
1) Introduction & Methods:
We have expanded the introduction to include a brief description of the parameters analyzed and the rationale for selecting human and rice as hosts. Additionally, we have clarified the details regarding the synthetic community design and the simulations (Lines 300–317).
2) Discussion of Limitations:
We have added a section discussing the limitations of our analysis, especially the challenges of applying these tools to real-world data. We have also outlined future directions, including plans to incorporate real data simulations as suggested by the reviewer (Lines 414–422).
3) Terminology Standardization:
We have ensured consistent use of key terms such as Raw, Removed, Microbiome, and Host Removal across the entire manuscript. These changes aim to enhance clarity and avoid any potential confusion for the readers.
4) Research Title:
We have changed our title as ‘Benchmarking short-read metagenomics tools for removing host contamination’.
5) Figures Issues:
We thoroughly reviewed all figures and addressed issues such as adding simulation line functions and correcting typographical errors.
All the detailed answer are attached below, with our responses in blue. We believe these revisions address the reviewers’ concerns and significantly improve our manuscript. Your feedback has been invaluable, and we are committed to further refining our work.

Sincerely,

Yong-Xin Liu
Agricultural Genomics Institute at Shenzhen, Chinese Academy of Agricultural Sciences
liuyongxin@caas.cn
Google Scholar: https://scholar.google.com/citations?user=NoxNy_IAAAAJ

Authors response to Reviewers
Reviewer reports:
Reviewer #1: The authors present the importance of host decontamination for accurate metagenomic analyses and benchmarked six bioinformatics tools for the removal of host reads. The analyses presented is comprehensive and statistically rigorous. This would be a useful resource for other researchers in the field of metagenomics to guide selection of appropriate tools for host read removal. Nevertheless, the introduction of the paper is missing some foundational information that makes it difficult to follow. This includes a brief introduction of the parameters that are being analyzed and the strategy for designing the synthetic communities as well as the motivation of specifically looking at human and rice as host in this analysis. The methods are lacking clarity into how the simulations were carried out and the type of sequencing technology that is being simulated. The paper also lacks discussion on limitations of the analyses, its broader implication in real data and elaboration on the biological relevance of the analysis. How would real samples be reflected/represented by the different percent of host contaminated reads as presented in this analysis. ie is 90% typical when studying skin surfaces? Given artifacts derived from sequencing, the impact of this work can be benefited by including an analysis of real-world data - ie known species spike-ins into known host background that is subsequently sequenced and compared to expected abundances.
Response: Thank you very much for your detailed and thoughtful feedback. We greatly appreciate your positive comments regarding the comprehensiveness and statistical rigor of our analysis. We have carefully

considered your suggestions and have made several revisions to enhance the clarity and depth of the manuscript.
1) For the missing foundational information, we have expanded them to include more essential background information. Specifically:
λ A brief description of the parameters used for synthetic community design has been added in the 'Methods' to improve clarity on the approach used. We also provide more details on how the simulations were conducted, including descriptions of the SynCom and SinBac that were simulated, to clarify this aspect of the methods (Line 309-317).
λ We have explained the rationale behind selecting human and rice as hosts in our analysis (Line 300-301). Human samples are emphasized due to their critical importance in medical research, especially in the context of the human microbiome. Rice, on the other hand, serves as a key model organism in agricultural research. Additionally, both species have well-annotated and complete genomes, which meet our computational needs.
2) For the discussion of limitations and broader implication, we have added the discussion the limitations of our analysis, including the potential challenges when applying these tools to 'real-world data' (Line 414-422). We have also elaborated on the broader implications of our findings for metagenomic research. For example, in microbiome studies, contamination levels can vary significantly, and high contamination percentages are not uncommon. We greatly appreciate your suggestion regarding the inclusion of real sequencing data performance. In future work, we plan to conduct real data simulations and incorporate new algorithms to develop host removal tools suited for various scenarios. Thank you again for your valuable suggestions.

Specific comments:
- Line 58 - Consider adding that sequencing resources could also be wasted in the process of requiring deeper sequencing to capture non-host reads.
Response: Thanks for your advice, here we have addressed this by emphasizing the waste of sequencing resources caused by sequencing unwanted host DNA reads and the additional need for deeper sequencing to accurately capture non-host reads.

- Line 85 - What does "genomes from 30 microbiota: refer to? 30 microbiome samples? Or 30 microbial species?
- What is the rationale for selection of 30 genomes/species? Was this based on a specific environment? Were the abundance of each species equal?
Response: Thank you for your valuable feedback. The phrase "genomes from 30 microbiota" refers to the genomes of 30 microbial species, as detailed in Table S1-2 (Microbiome Composition). We selected these species based on the reference "Cheng AG, Ho P-Y, Aranda-Díaz A, et al. Design, construction, and in vivo augmentation of a complex gut microbiome. Cell 2022; 19:3617-36", where we randomly chose 30 common human-associated microbial species for simulation. Each species was represented by 0.1 million reads, resulting in a total of 3 million reads for the dataset, as described in the Methods section under "Simulated dataset description for the downstream analysis".
To avoid any confusion for the readers, we have revised the wording in the relevant sections. Specifically, in the Results section, we changed "incorporating genomes from 30 microbiota and Homo sapiens (GRCh38)" to "incorporating microbial reads from 30 species, each represented at equal abundance, along with human reads from Homo sapiens (GRCh38)". Additionally, in the Methods section, we provided the reference for the microbial community used in our simulations.

- Line 90 - what part of the metagenomic analysis? Does this refer to the classification task?
- More details can be provided here regarding the types of analyses that are being done downstream to evaluate efficacy of host decontamination, i.e. MAG assembly, functional annotation etc.
Response: Thank you for your insightful comments. We have revised the description of the metagenomic analysis to provide more details about the specific types of analyses performed to evaluate the efficacy of host decontamination, as well as clarifying what part of the analysis we are referring to.

- Line 102 - The different use of lower and uppercase to refer to the samples are confusing. Consistent use of the uppercase version "R"aw, "R"removed, "M"icrobiome would help with clarity.
Response: Thank you for your suggestion. We have standardized the terminology by consistently using uppercase for all sample references: 'Raw', 'Removed', and 'Microbiome'.

- Line 102 - More context can be given here as to why the relative abundance is "altered". It is obvious that the presence of host reads would skew the relative abundances. Would it be more informative to present the absolute counts?
Response: Thank you for your insightful suggestion. In Figure 1C, we observed that the Raw data impacted the relative abundance due to the presence of host reads. However, after applying Kraken2's decontamination (removing Chordata), the relative abundance results aligned with those annotated for the Microbiome and Removed datasets. This is consistent with the rationale presented in our introduction, where we discussed the use of Kraken2 for host decontamination.
However, due to limitations in the reference database, the annotation results may not fully match our simulated data. Although we used the Kraken2 PlusPFP database—the most comprehensive available at 150 GB (as mentioned in the Methods section under 'Metagenomic Analysis')—we opted not to directly compare the classification data with true data to avoid potential misinterpretation by readers. Instead, we addressed this limitation in the Discussion section.
Regarding your suggestion to present absolute abundances, we found that in the Raw data, the majority of reads were classified as Chordata, but after decontamination, the remaining taxa annotations were consistent with those in the Microbiome and Removed datasets (the Table 1 was attached below). After discussion among all co-authors, we decided to retain the current presentation of relative abundance results.
Table 1. Absolute counts of the top 10 phyla.

Phylum	Microbiome data			Raw data			Remove data		
	S1	S2	S3	S1	S2	S3	S1	S2	S3
Bacillota	897000	896623	896190	897001	896623	896190	897000	896623	896190
Bacteroidota	547070	547173	547005	547070	547173	547006	547070	547173	547005
Actinomycetota	502823	502948	502800	502823	502948	502800	502823	502948	502800
Verrucomicrobiota	100065	100099	100105	100065	100099	100105	100065	100099	100105
Thermodesulfobacteriota	80667	80471	80450	80667	80471	80450	80667	80471	80450
Pseudomonadota	16747	16761	16742	16854	16761	16777	16747	16761	16742
Streptophyta	14428	14170	14492	14456	14250	14586	14428	14170	14492
Campylobacterota	1642	1642	1662	1642	1642	1662	1642	1642	1662
Chordata	1343	1303	1316	27001112	27001115	27000944	2828	2855	2879
Mycoplasmatota	736	775	765	736	775	765	736	775	765
Others	3337	3288	3246	3339	3291	3248	3337	3288	3246

- Line 104 - What does "released taxa" refer to? It seems that filtering out reads that align with Chordata achieved similar results as the Microbiome-only sample. If that is the case, what would the benefit of host decontamination be here?
Response: Thank you for your question. By "released taxa", we were referring to the microbial taxa that became visible after removing the Chordata (human) reads from the Raw data. This allowed us to better focus on the microbial community, resulting in similar outcomes to those observed in the Microbiome-only sample. To avoid any confusion, we have replaced "released taxa" with "remaining taxa" and added a reference to Figure 1C, de-chordata group.
Regarding the benefit of host decontamination, in the Raw data, Kraken2's species annotation included a large amount of host information (Chordata), which affected the accuracy of microbial community profiling. In contrast, the Remove data, where host reads were filtered out using KneadData, showed a microbial community composition more similar to the Microbiome data. Although Kraken2 can be used both for species annotation and host removal, this highlights the importance of using dedicated host decontamination tools like KneadData to improve species annotation accuracy. This is also why we further evaluate different tools in the next section of the manuscript.
Additionally, due to limitations in the database of Kraken2, the annotated results differed from the actual simulated data, an issue we address in the Discussion section. We hope that by sharing our findings, we can contribute to the ongoing refinement of metagenomic analysis tools.

- Line 105 - What about the Shannon index results?
Response: Thank you for your question, and we are so sorry that we have included the Shannon index in Figure S2A, while not addressing the Shannon index results in our initial description. Our analysis showed a significant difference in the Shannon index across the Microbiome, Removed, and Raw data. The values for

the Microbiome data were closely grouped (3.3397; 3.3390, 3.3402) and were higher than those for the Removed data (3.3425; 3.3419, 2.2431). In contrast, the Raw data had notably lower values (0.4549; 0.4549, 0.4565). This indicates that the removal of host reads improves the diversity estimates of the microbial community compared to the Raw data. After discussion, we decided to focus solely on the Richness index in our presentation, as it provides a clearer representation of microbial diversity and avoids potential confusion.

- Line 108 - It seems to be unusual that the first axis of the PCoA explains 100% of variation. Is this expected?
Response: Thank you for your insightful question. We acknowledge that it may seem unusual for the first axis of the Principal Coordinates Analysis (PCoA) to explain 100% of the variation. This result typically indicates that the data has low dimensionality, which can occur when the microbial community composition is dominated by a few taxa or when there is minimal variation among the samples.
In our case, this observation suggests that the removal of host reads led to a clearer distinction between the sample groups, resulting in a single axis capturing all the variability. While this is not the most common scenario in more complex datasets, it highlights the significant impact of host decontamination on the observed microbial community structure. We appreciate your attention to this detail and will clarify this point in the manuscript to provide better context for our findings.

- Line 115 - There seems to be no further discussion about the recovery of B. breve from only the raw samples. Any hypotheses of why this could have been the case?
Response: Thank you for your question. To assess the recovery of B. breve, we compared the MAGs obtained from our analysis with the simulated Bifidobacterium breve data (GCF_001025175.1). The ANI similarity between the MAGs and the reference genome ranged from 99.8% to 99.9%. In our manuscript, we used MetaWRAP refinement to improve the quality of our MAGs binning, applying the parameters -c 50 -x 10, meaning we retained only bins with completeness over 50% and contamination below 10% (Table 2, MAGs in blue).
However, when we kept all binning data and adjusted the MetaWRAP refinement parameters to -c 0 -x 100, we indeed recovered additional low-quality MAGs (Table 2, MAGs in green). With this adjustment, B. breve was enriched in a greater number of Microbiome and Remove data, displaying a completeness of 85.42% to 85.89% and contamination of 12.91% to 14.46%. Additionally, we examined all enriched data and found no clear patterns, except that host removal generally improves the recovery of high- and medium-quality MAGs, though some specific groups may still be enriched in raw data.
To provide readers with more information, we have added our MAG filtering parameters to the Materials and Methods section and included this discussion in the discussion section.
Table2. Reconstruetd MAGs of B. breve.
Samples fastANI completeness contamination GC N50 size MAG quality
Microbiome1 99.45 85.42 13.6 0.587 15788 1898963 Low
Microbiome2 99.8024 94.81 8.156 0.587 18589 2083715 Medium
Microbiome3 99.6876 85.89 12.91 0.588 18766 1967196 Low
Remove1 99.4102 85.42 14.46 0.587 15444 1973302 Low
Remove2 99.8168 94.81 8.156 0.587 18589 2083715 Medium
Remove3 99.6882 85.89 12.91 0.588 18766 1965172 Low
Raw1 99.8909 83.18 1.109 0.588 16512 1695982 Medium
Raw2 99.8911 93.28 5.66 0.589 19278 2050916 Medium
Raw3 99.843 90.34 2.707 0.589 19070 1910714 High
* fastANI represents the comparison between reconstructed MAGs of different samples and reference genome (Bifidobacterium breve fasta data (GCF_001025175.1)).
High MAGs, completeness ≥ 90% and Contamination ≤ 5%; Medium MAGs, completeness ≥ 50% and Contamination < 10%; Low MAGs, completeness < 50% or Contamination > 10%

- Line 118 - In what aspect does “higher similarity” refer to here? What are the numbers in the x and y axis of Fig. 1E?
Response: Thank you for your insightful question. The term ‘higher similarity’ refers to the Spearman correlation between the GO terms in different datasets. Specifically, we compared the GO term annotations between Microbiome data, Raw data, and Removed data. In Figure 1E, the x-axis represents the GO term counts in the Microbiome data, while the y-axis represents the GO term counts in the Raw and Removed

data, respectively. To make this clearer, we have now added linear regression lines to the plot for better visualization of the relationship. And we have replaced the phrase 'higher similarity' with 'stronger correlation' in our manuscript to avoid any potential confusion.

- Line 124 - how many species are included in the SynCom and were the communities designed to reflect either a human-associated microbiome and a rice microbiome? This is later mentioned in the methods but stating in here could help with interpretability. Furthermore, what is the rationale for simulating equal abundances of each species?
Response: We apologize for not providing sufficient details regarding the SynCom. In our study, the SynCom included 14 species for the rice microbiome and 35 species for the human-associated microbiome. The simulated data were based on core microbiota reported in the literature (see Table S2-1). For further clarification, we have included a detailed description in the Methods section and noted in Line 124 that readers can find additional information there.
Regarding the rationale for simulating equal abundances of each species, we initially employed the differential mode from the CAMISIM software, which captures a greater degree of randomness and reflects natural samples more closely. However, in the first part of our results, we chose to use equal abundances to emphasize the impact of host decontamination on the recoverable microbial MAGs. In other parts of the manuscript, we utilized a differential approach that better represents natural sample dynamics.

- Line 130 - The description of the abbreviations are somewhat confusing. Is SinBac 60-3 supposed to be SynBac 60-3 here? In Fig. 2A the bar graphs in the center showing the proportions of host vs bacteria reads does not seem to be consistent with the vertical bar graphs in the same plot.
Response: Thank you so much for pointing out this issue. Here, it should be 'SynCom 60-3' instead of 'SinBac 60-3'. We have reviewed the entire manuscript for consistency and made the necessary corrections. Additionally, we have updated Figure 2A to ensure that the proportions of host versus bacterial reads in the bar graphs are consistent with the vertical bar graphs in the same plot.

- Line 182 - What were some of the false positives/ false negatives? Were these specific species that are consistently misclassified? What taxonomic levels were the analysis caried out at? Do these metrics differ at different taxonomic levels?
Response: Thank you for your question. In our study, we evaluated the effectiveness of host read removal using various software tools. True positives refer to host reads that were correctly identified and removed as host genome sequences. False positives represent microbiota reads that were mistakenly classified as host reads and thus removed, while false negatives refer to host reads that were not identified and remained in the dataset.
Our analysis was conducted at the read level, as each simulated metagenomic read was labeled with its specific origin. Therefore, we did not focus on identifying consistently misclassified species at different taxonomic levels, since the classification was based on individual reads rather than specific taxa. However, the potential for misclassification of certain reads is an area of interest, and in future work, we plan to conduct a more detailed analysis of these misclassified reads to better understand patterns in distinguishing between host and microbial sequences more accurately.

- Lines 195-198 - If KrakenUniq also displays low Mammalia values, shouldn't it be considered as being able to remove more host contamination reads rather than less?
Response: Thank you for pointing this out. We appreciate your careful observation. What we intended to express here is that, among the three tools, BWA and KneadData demonstrate better host read removal performance compared to KrakenUniq. We have revised the original text to clarify this point.

- Line 212 - How does different versions of references genomes impact host removal? (ie GRCh38, telomere-to-telomere gapless sequence of the human genome). Can a similar evaluation be carried out to assess the impacts of these versions?
Response: Thanks so much for your suggestion, we acknowledge that different versions of reference genomes may have varying impacts on host removal efficiency. In our future work, we plan to evaluate these differences.

- Line 231 - Does "the absence of a host reference genome" mean absence of a host reference genome that is closely aligned with the host whereby samples/sequences were derived from? If yes, this might

have to be reworded for clarity.
- It is not always possible to obtain host genomes that are specific to a given sample type - are there recommendations for what can be done to select a suitable host genome?
Response: We appreciate your suggestion. We have revised our wording to clarify that ‘the absence of a host reference genome’ now reads as ‘the absence of a closely aligned and accurate reference genome’. Additionally, we have thoroughly reviewed and updated the relevant descriptions throughout the manuscript. Regarding the selection of a suitable host genome, our experience suggests that using a pangenome approach during database construction can be effective. We plan to validate this approach in our upcoming research. Additionally, we are exploring the integration of deep learning algorithms to address the challenges posed by the absence of precise reference genomes.

- Line 245 - How were the simulations carried out in CAMISIM? What were the simulation parameters?
Response: Thank you for your question. We have expanded the Methods section to provide a clearer understanding of how the simulations were conducted in CAMISIM. This includes a detailed description of the simulation parameters used, ensuring readers can better grasp how we obtained the simulated data.

- Lines 299-304 - These text appears to be repeated from the results section above (line 122-127).
Response: Thank you for pointing this out. Our initial intention was to maintain consistency to avoid confusion for readers. We have now streamlined the description in lines 122-127 to allow readers to focus more on the results section.

Please correct all spelling mistakes in the text, figures and figure legends.
For example:
- Figure 1A 0.1million reads/species, Diversity analysis, Memory usage
- Line 367 - 27 million "reads"
Line 369 - "equal"
Response: Thank you so much for your thorough review. We have carefully checked and revised our entire manuscript.

Reviewer #2: Overall Impressions
The authors present a nice comparison of different methods for removing host data from metagenomic sequencing data. As this focuses entirely on short-read metagenomic data, I suggest altering the title to communicate this.
Response: Thank you for your suggestion. We have changed our title as ‘Benchmarking short-read metagenomics tools for removing host contamination’.

Major Issues
Can the authors comment on whether a two stage-approach would be beneficial for data from hosts with a good-quality reference genome (eg. human or mouse). I have noticed in my own data that, after decontamination by Bowtie2 alignment against the human reference genome, some reads are still classified by Kraken2 as Homo sapiens - which I then remove. Would implementing this as standard be beneficial or offer only a marginal increase in data quality in return for the additional processing time and resources.
The authors highlight that Kraken2 seems to be suitable for identifying which reads are from Homo sapiens (L104, Fig 1C) - they could use this info to add another decontamination method to the evaluation (Kraken2 classification of Raw data followed by extracting non-chordata reads using the extract_kraken_reads.py script in KrakenTools).
Response: Thank you for your insightful question. In metagenomic studies, we often work with data from numerous samples, while accurate reference genomes for each host (human) are hardly available. Thus, we typically construct a reference index based on a publicly available host genome and perform host removal analyses using tools like Bowtie2 or KneadData. And it is also common for Kraken2 to annotate additional host reads. Based on our team’s experience, we also directly remove these extra host reads use the extract_kraken_reads.py script.
The issue of residual host contamination due to the lack of an accurate reference genome is evident in our data. For example, when using Bowtie2 with a precise host reference genome, we achieve data accuracies ranging from 0.9973 to 0.9999 (Figure 3A). In contrast, when a precise reference genome is absent, the

accuracy of our data drops to a range of 0.7552 to 0.9999 (Figure 4C).
Regarding the potential benefits of a two-step approach for host contamination removal, we strongly support this method. Host contamination can significantly affect the accuracy of downstream functional annotations (Figure 1E). In our original manuscript, we discussed various host removal strategies that balance speed and accuracy, and we developed a workflow called HostPurge (https://github.com/HaoLuo-leo/HostPurge). In our future work, we will further optimize the HostPurge workflow to provide a precise and efficient solution for host contamination removal.

Why was the number of reads chosen for the microbial species included? The authors focus on the reconstruction of MAGs and note that some species were not recovered in some groups - it is likely that increasing the number of reads overall, without adjusting the % of reads from microbiome and host, will impact this.
Response: Thank you for your question. In our study, we aimed to assess the impact of host reads on the generation of Metagenome-Assembled Genomes (MAGs) from microbial communities, particularly in samples with high host proportions (such as 90%). Typically, metagenomic datasets are around 10 Gb in size. If we were to increase the number of reads from the microbial species, the overall sequencing coverage would also need to be increased, which would significantly raise the costs associated with sequencing and computational analysis.
Our focus was to investigate the effects of high host read proportions on the recoverability of MAGs under standard sequencing conditions. In our simulations, the 30 million reads generated approximately 8 Gb of data, which aligns with the typical sequencing output in routine studies. This design allows us to explore the dynamics of host contamination in a realistic context while keeping the analysis feasible.

Were the MAGs that were not recovered from species with larger genomes? The same number of reads from a larger genome will likely return a smaller MAGs due to a lower breath and depth of coverage. Also, can the authors state why MAGs were reconstructed in the first place? Was it for data quality purposes?
Response: Thank you for your question. The quality of the data is indeed crucial for recovering high-quality MAGs, which serve as a foundation for further investigation into the functionality, metabolism, and evolution of specific species or strains. Reconstructing MAGs is a vital step in metagenomic data analysis, as it enables functional validation of microbial species or strains identified in the sample.
In our study, each microbial species was represented by 0.1 million reads, with genome sizes ranging from approximately 2 to 6 Mb (Table S1-2, S2-1). This resulted in a coverage depth of 5~15× for each genome. To determine whether insufficient coverage might explain the failure to reconstruct certain MAGs, we checked the genomes of reconstructed and unreconstructed MAGs. Here two reconstructed MAGs did not match our reference data, but we did not observe a strong correlation between genome size and the ability to recover MAGs (Table 1).
In this work, we used MetaWRAP refinement to improve the binning of our MAGs with parameters -c 50 -x 10, meaning we retained only those bins with completeness greater than 50% and contamination less than 10%. However, when we retained all binning data and adjusted the MetaWRAP refinement parameters to -c 0 -x 100, we indeed recovered additional low-quality MAGs, while we still did not see the strong correlation
We hypothesize that, in addition to genome size, factors such as the heterogeneity of the simulated data and the presence of specific bacterial genes might influence both the quantity or quality of the MAGs that can be recovered.
Table 1. The genome size of simulated microbiota.
Species Genome_Size Reconstructed MAGs this work Adjusted parameter (-c 0 -x 100)
Veillonella_dispar 2.1Mb No No
Absiella_dolichum 2.2Mb No No
Ruminococcus_bromii 2.2Mb No No
Acidaminococcus_fermentans 2.3Mb Yes Yes
Anaerofustis_stercorihominis 2.3Mb Yes Yes
Collinsella_aerofaciens 2.3Mb No Yes
Bifidobacterium_breve 2.3Mb Yes Yes
Bifidobacterium_pseudocatenulatum 2.3Mb Yes Yes
Holdemanella_biformis 2.5Mb No Yes
Burkholderiales_bacterium 2.6Mb No No
Akkermansia_muciniphila 2.7 Mb Yes Yes

Desulfovibrio_piger 2.9Mb No No
Adlercreutzia_equolifaciens 2.9Mb No No
Ethanoligenens_harbinense 3.0Mb Yes Yes
Slackia_heliotrinireducens 3.1Mb Yes Yes
Clostridiales_bacterium 3.2Mb No No
Anaerobutyricum_hallii 3.3Mb No No
Eubacterium_rectale 3.4 Mb No No
Clostridium_methylpentosum 3.5Mb No No
Eggerthella_lenta 3.6Mb Yes Yes
Holdemania_filiformis 3.6Mb No No
Alistipes_finegoldii 3.7Mb Yes Yes
Clostridium_hylemonae 3.8Mb No No
Roseburia_inulinivorans 4.2Mb No No
Bacteroides_coprocola 4.3Mb No No
Odoribacter_splanchnicus 4.4Mb Yes Yes
Lacrimispora_saccharolytica 4.6Mb Yes Yes
Parabacteroides_distasonis 4.7 Mb Yes Yes
Phocaeicola_vulgatus 5.2 Mb No No
Bacteroides_xylanisolvens 6.0Mb No No

Kneaddata uses Bowtie2 to do the alignments, what is the difference between the Bwotie2 approach presented here and the alignment step performed by Bowtie2 in Kneaddata?
Response: Thank you for your question. In our software evaluation, we used the default or recommended parameters for each tool. While both Bowtie2 and KneadData use the Bowtie2 alignment algorithm, there are differences in how they handle host contamination removal.
For example, KneadData is an integrated pipeline that automates various preprocessing steps for metagenomic data, including quality control (such as trimming low-quality reads) and decontamination (aligning reads to a host genome). In our evaluation, we used the --bypass-trim and --bypass-trf options to skip these preprocessing steps, but our results showed that the outcomes of KneadData and Bowtie2 were not completely identical. Upon further investigation, we found that the default parameters differ between the two: Bowtie2 uses the --sensitive-local setting [-D 15 -R 2 -N 0 -L 22 -i S,1,1.15], while KneadData applies the --very-sensitive-local setting [-D 20 -R 3 -N 0 -L 20 -i S,1,0.5]. This could be one of the reasons for the observed differences. Therefore, we still presented their differences in our work to highlight their variation.
As we have already discussed the parameter settings for different software in the background section, we do not delve into a detailed comparison of parameters in the main body of the manuscript.

Minor Issues
Throughout: the authors switch between the terms "host-depleted" and "Remove" to refer to the data which has been decontaminated. I suggest sticking to one, the former is more descriptive and tends to work better in context, but it is up to the authors.
Response: Thank you for your suggestion. Apart from the section where we compare Raw data, Remove data, and Microbiome data, we have standardized the term 'host-removal' throughout the rest of the manuscript to ensure consistency and improve readability for the readers.

L49: I realise this is not the aim of the manuscript, but could the authors briefly mention the different methods that exist for microbial DNA enrichment (or host DNA depletion) before library preparation and sequencing.
Response: We appreciate your suggestion. A postdoc of our team, who specializes in this area, has written a review on microbial DNA enrichment and host DNA depletion methods during experiments. This review is currently being prepared for submission and we hope it will provide valuable insights to the field on this topic.

L51-52: I agree that this is a concern. Can the authors provide a reference to any manuscripts which have evaluated this? If they exist. It would be important context for the reader and might also address my previous point.
Response: Thank you for your insightful question. As mentioned earlier, the review will also address this

concern, summarizing the different methods and their evaluations, which can provide important context for the reader.

L63: can the authors hypothesise about a particular reason for the increase began in 2015 - is this due to a paper or commentary highlighting the importance? Or is this an artefact of the increased quantity of microbiome papers published over this period?
Response: Thank you for your question. It's hard to pinpoint a specific reason for the increase beginning in 2015. Our survey indicates that the number of research articles employing metagenomic analysis (excluding amplicon studies) gradually increased from 2012 to 2014. However, we cannot definitively say whether this rise correlates with specific events, such as the release of Kraken in 2014.
Moreover, in our opinion, it's possible that authors may have employed these steps but chose not to include details in their descriptions. While we apologize for not being able to provide more specific information, it is clear that an increasing number of articles are beginning to recognize the importance of detailing host removal procedures.

L130: I think the description is incorrect here. The even split would be SinBac 60-2, while the 90% host and 10% community would be SynCom 60-1?
Response: Thank you so much for pointing out this issue. Here, it should be 'SynCom 60-3' instead of 'SinBac 60-3'. We have reviewed the entire manuscript for consistency and made the necessary corrections.

L182- L188 - can the authors please comment on a) whether there were specific microbial species, genes, or sets thereof which were commonly erroneously identified as host, and b) were these erroneously unmapped reads subsequently assigned taxonomy by Kraken2. These are important considerations as they may impact the downstream microbiome profile.
Response: Thank you very much for your insightful question. Indeed, we encountered instances in our study where some reads were detected by Kraken2 after being filtered for host contamination using Bowtie2 or KneadData. We believe this discrepancy arises from the differences between alignment-based algorithms and k-mer algorithms, as well as our inability to provide precise host reference genomes.
As for whether specific microbial species, genes, or sets of reads were commonly misidentified as host, we do not have definitive conclusions at this time. However, identifying these specific data types will be a key focus of our future work. We appreciate your valuable suggestions.

Discussion: generally, journals prefer that you do not refer to Figures and Tables in the discussion section. They should already be introduced in the results and methods section. Check that this is allowed by Gigascience.
Response: Thank you for your advice. We checked the formatting guidelines of GigaScience, and while we did not find specific requirements regarding the referencing of figures and tables in the discussion section, we all agree that your suggestion is reasonable. Therefore, we have removed the relevant references from the discussion. Thank you for your helpful feedback.

L369: "equal"
Response: We appreciate your feedback and have made the necessary changes.

L387-388: This could be tested by including reference genomes from other genera in the same family, if available.
Response: Thank you very much for your suggestion. We plan to implement this work in the future development of the HostPurge pipeline https://github.com/HaoLuo-leo/HostPurge. In our upcoming research, we will investigate whether our software performs better with reference genomes from the same genera or family, or even in cases where no reference genomes are available.
