# Supplementary material for: Benchmarking short-read metagenomics tools for removing host contamination
Source: Gigascience. 2025 Feb 27;14:giaf004. doi: 10.1093/gigascience/giaf004 (PMC11878760; doi:10.1093/gigascience/giaf004)
Supplement: giaf004_Response_to_Reviewer_Comments_Revision_1 [file giaf004_response_to_reviewer_comments_revision_1.pdf]

Author's Response To Reviewer Comments

Dear Hans Zauner,
Thank you for your email and for providing the reviewers' feedback on our manuscript, "Benchmarking short-read metagenomics tools for removing host contamination" (GIGA-D-24-00318R1)". We appreciate the time and effort both you and the reviewers have invested in assessing our work.
We are grateful for the constructive comments, which we believe have helped us improve the manuscript further. Below, we provide a point-by-point response to the reviewers' suggestions and address the additional editorial issues raised in your letter.
Editorial Issues
• Adding RRIDs for software tools:
We have now included the RRID identifiers for all software tools mentioned in the Methods section where available. For instance, Megahit 1.0 is now cited as "Megahit 1.0 (RRID:SCR_018551)" (line 286). However, for some software, such as CAMISIM, KneadData, Kraken2, HUMAnN3, MetaWRAP, dRep, GTDBtk, CheckM2, emapper, KMCP, and KrakenUniq, we were unable to locate RRIDs despite thorough searches in the RRID database. If there are alternative databases or resources where these RRIDs might be found, we would be happy to conduct further searches or update the manuscript accordingly.
• In-text URLs/DOIs moved to bibliography:
We have moved all URLs to the bibliography and cited them in the text by number.
• Abstract restructuring:
The abstract has been restructured into 'Background - Results – Conclusions' format as requested.
• ORCID IDs:
ORCID for all authors have been added.
• Software availability section:
Thank you for the reminder. We have now added the requested software section in the specified format as instructed.
• Conformance to journal style:
We have carefully reviewed the manuscript to ensure it adheres to the journal's style guidelines.
Response to Reviewer #1
Thank you for your language suggestions. We have made the recommended changes and thoroughly reviewed the manuscript.
• Adding discussion of the Low-quality B. breve MAGs observation
Response: We agree with your suggestion. A discussion regarding the detection of low-quality B. breve MAGs in Microbiome and Raw samples has been added to the Discussion section in Line 285-292.
Response to Reviewer #2
• Regarding the parameters of Bowtie 2:
While this comment was not included in the initial review, we agree that it raises an interesting point. We have added a brief discussion of this in the relevant section of the manuscript (see lines 304-314). We hope this addition adequately addresses the reviewer's concern.

Thank you once again for your guidance and support throughout the review process. We look forward to your further feedback and the opportunity to publish our work in GigaScience.

Best regards,
Yong-Xin Liu
On behalf of all co-authors
