# Supplementary material for: Benchmarking short-read metagenomics tools for removing host contamination
Source: Gigascience. 2025 Feb 27;14:giaf004. doi: 10.1093/gigascience/giaf004 (PMC11878760; doi:10.1093/gigascience/giaf004)
Supplement: giaf004_Reviewer_1_Report_Original_Submission — Car Reen Kok -- 9/19/2024 [file giaf004_reviewer_1_report_original_submission.pdf]

Reviewer Report

Title: Benchmarking short-read metagenomics tools for removing host contamination

Version: Original Submission **Date:** 9/19/2024

Reviewer name: Car Reen Kok

Reviewer Comments to Author:

The authors present the importance of host decontamination for accurate metagenomic analyses and benchmarked six bioinformatics tools for the removal of host reads. The analyses presented is comprehensive and statistically rigorous. This would be a useful resource for other researchers in the field of metagenomics to guide selection of appropriate tools for host read removal. Nevertheless, the introduction of the paper is missing some foundational information that makes it difficult to follow. This includes a brief introduction of the parameters that are being analyzed and the strategy for designing the synthetic communities as well as the motivation of specifically looking at human and rice as host in this analysis. The methods are lacking clarity into how the simulations were carried out and the type of sequencing technology that is being simulated. The paper also lacks discussion on limitations of the analyses, its broader implication in real data and elaboration on the biological relevance of the analysis. How would real samples be reflected/represented by the different percent of host contaminated reads as presented in this analysis. ie is 90% typical when studying skin surfaces? Given artifacts derived from sequencing, the impact of this work can be benefited by including an analysis of real-world data - ie known species spike-ins into known host background that is subsequently sequenced and compared to expected abundances.
Specific comments:
- Line 58 - Consider adding that sequencing resources could also be wasted in the process of requiring deeper sequencing to capture non-host reads.
- Line 85 - What does "genomes from 30 microbiota: refer to? 30 microbiome samples? Or 30 microbial species?
- What is the rationale for selection of 30 genomes/species? Was this based on a specific environment? Were the abundance of each species equal?
- Line 90 - what part of the metagenomic analysis? Does this refer to the classification task?
- More details can be provided here regarding the types of analyses that are being done downstream to evaluate efficacy of host decontamination,
ie MAG assembly, functional annotation etc.
- Line 102 - The different use of lower and uppercase to refer to the samples are confusing. Consistent use of the uppercase version "R"aw, "R"removed, "M"icrobiome would help with clarity.
- Line 102 - More context can be given here as to why the relative abundance is "altered". It is obvious that the presence of host reads would skew the relative abundances. Would it be more informative to present the absolute counts?
- Line 104 - What does "released taxa" refer to? It seems that filtering out reads that align with Chordata achieved similar results as the Microbiome-only sample. If that is the case, what would the benefit of

host decontamination be here?
- Line 105 - What about the Shannon index results?
- Line 108 - It seems to be unusual that the first axis of the PCoA explains 100% of variation. Is this expected?
- Line 115 - There seems to be no further discussion about the recovery of B. breve from only the raw samples. Any hypotheses of why this could have been the case?
- Line 118 - In what aspect does "higher similarity" refer to here? What are the numbers in the x and y axis of Fig. 1E?
- Line 124 - how many species are included in the SynCom and were the communities designed to reflect either a human-associated microbiome and a rice microbiome? This is later mentioned in the methods but stating in here could help with interpretability. Furthermore, what is the rationale for simulating equal abundances of each species?
- Line 130 - The description of the abbreviations are somewhat confusing. Is SinBac 60-3 supposed to be SynBac 60-3 here? In Fig. 2A the bar graphs in the center showing the proportions of host vs bacteria reads does not seem to be consistent with the vertical bar graphs in the same plot.
- Line 182 - What were some of the false positives/ false negatives? Were these specific species that are consistently misclassified? What taxonomic levels were the analysis caried out at? Do these metrics differ at different taxonomic levels?
- Lines 195-198 - If KrakenUniq also displays low Mammalia values, shouldn't it be considered as being able to remove more host contamination reads rather than less?
- Line 212 - How does different versions of references genomes impact host removal? (ie GRCh38, telomere-to-telomere gapless sequence of the human genome). Can a similar evaluation be carried out to assess the impacts of these versions?
- Line 231 - Does "the absence of a host reference genome" mean absence of a host reference genome that is closely aligned with the host whereby samples/sequences were derived from? If yes, this might have to be reworded for clarity.
- It is not always possible to obtain host genomes that are specific to a given sample type - are there recommendations for what can be done to select a suitable host genome?
- Line 245 - How were the simulations carried out in CAMISIM? What were the simulation parameters?
- Lines 299-304 - These text appears to be repeated from the results section above (line 122-127).

Please correct all spelling mistakes in the text, figures and figure legends.
For example:
- Figure 1A) 0.1million reads/species, Diversity analysis, Memory usage
- Line 367 - 27 million "reads"

Line 369 - "equal"

Methods

Are the methods appropriate to the aims of the study, are they well described, and are necessary controls included? Choose an item.

Conclusions

Are the conclusions adequately supported by the data shown? Choose an item.

Reporting Standards

Does the manuscript adhere to the journal's guidelines on minimum standards of reporting? Choose an item.

Choose an item.

Statistics

Are you able to assess all statistics in the manuscript, including the appropriateness of statistical tests used? Choose an item.

Quality of Written English

Please indicate the quality of language in the manuscript: Choose an item.

Declaration of Competing Interests

Please complete a declaration of competing interests, considering the following questions:

- Have you in the past five years received reimbursements, fees, funding, or salary from an organisation that may in any way gain or lose financially from the publication of this manuscript, either now or in the future?
- Do you hold any stocks or shares in an organisation that may in any way gain or lose financially from the publication of this manuscript, either now or in the future?
- Do you hold or are you currently applying for any patents relating to the content of the manuscript?
- Have you received reimbursements, fees, funding, or salary from an organization that holds or has applied for patents relating to the content of the manuscript?
- Do you have any other financial competing interests?
- Do you have any non-financial competing interests in relation to this paper?

If you can answer no to all of the above, write 'I declare that I have no competing interests' below. If your reply is yes to any, please give details below.

I declare that I have no competing interests

I agree to the open peer review policy of the journal. I understand that my name will be included on my report to the authors and, if the manuscript is accepted for publication, my named report including any attachments I upload will be posted on the website along with the authors' responses. I agree for my report to be made available under an Open Access Creative Commons CC-BY license (http://creativecommons.org/licenses/by/4.0/). I understand that any comments which I do not wish to

be included in my named report can be included as confidential comments to the editors, which will not be published.

Choose an item.

To further support our reviewers, we have joined with Publons, where you can gain additional credit to further highlight your hard work (see: https://publons.com/journal/530/gigascience). On publication of this paper, your review will be automatically added to Publons, you can then choose whether or not to claim your Publons credit. I understand this statement.

Yes Choose an item.
