# Supplementary material for: Benchmarking short-read metagenomics tools for removing host contamination
Source: Gigascience. 2025 Feb 27;14:giaf004. doi: 10.1093/gigascience/giaf004 (PMC11878760; doi:10.1093/gigascience/giaf004)
Supplement: giaf004_Reviewer_2_Report_Original_Submission — Calum Walsh -- 9/25/2024 [file giaf004_reviewer_2_report_original_submission.pdf]

Reviewer Report

Title: Benchmarking short-read metagenomics tools for removing host contamination

Version: Original Submission **Date:** 9/25/2024

Reviewer name: Calum Walsh

Reviewer Comments to Author:

Overall Impressions
The authors present a nice comparison of different methods for removing host data from metagenomic sequencing data. As this focuses entirely on short-read metagenomic data, I suggest altering the title to communicate this.
Major Issues
Can the authors comment on whether a two stage-approach would be beneficial for data from hosts with a good-quality reference genome (eg. human or mouse). I have noticed in my own data that, after decontamination by Bowtie2 alignment against the human reference genome, some reads are still classified by Kraken2 as Homo sapiens - which I then remove. Would implementing this as standard be beneficial or offer only a marginal increase in data quality in return for the additional processing time and resources. The authors highlight that Kraken2 seems to be suitable for identifying which reads are from Homo sapiens (L104, Fig 1C) - they could use this info to add another decontamination method to the evaluation (Kraken2 classification of Raw data followed by extracting non-chordata reads using the extract_kraken_reads.py script in KrakenTools).
Why was the number of reads chosen for the microbial species included? The authors focus on the reconstruction of MAGs and note that some species were not recovered in some groups - it is likely that increasing the number of reads overall, without adjusting the % of reads from microbiome and host, will impact this.
Were the MAGs that were not recovered from species with larger genomes? The same number of reads from a larger genome will likely return a smaller MAGs due to a lower breath and depth of coverage. Also, can the authors state why MAGs were reconstructed in the first place? Was it for data quality purposes?
Kneaddata uses Bowtie2 to do the alignments, what is the difference between the Bwotie2 approach presented here and the alignment step performed by Bowtie2 in Kneaddata?
Minor Issues
Throughout: the authors switch between the terms "host-depleted" and "Remove" to refer to the data which has been decontaminated. I suggest sticking to one, the former is more descriptive and tends to work better in context, but it is up to the authors.
L49: I realise this is not the aim of the manuscript, but could the authors briefly mention the different methods that exist for microbial DNA enrichment (or host DNA depletion) before library preparation and sequencing.
L51-52: I agree that this is a concern. Can the authors provide a reference to any manuscripts which have evaluated this? If they exist. It would be important context for the reader and might also address

my previous point.
L63: can the authors hypothesise about a particular reason for the increase began in 2015 - is this due to a paper or commentary highlighting the importance? Or is this an artefact of the increased quantity of microbiome papers published over this period?
L130: I think the description is incorrect here. The even split would be SinBac 60-2, while the 90% host and 10% community would be SynCom 60-1?
L182- L188 - can the authors please comment on a) whether there were specific microbial species, genes, or sets thereof which were commonly erroneously identified as host, and b) were these erroneously unmapped reads subsequently assigned taxonomy by Kraken2. These are important considerations as they may impact the downstream microbiome profile.
Discussion: generally, journals prefer that you do not refer to Figures and Tables in the discussion section. They should already be introduced in the results and methods section. Check that this is allowed by Gigascience.
L369: "equal"
L387-388: This could be tested by including reference genomes from other genera in the same family, if available.

Methods

Are the methods appropriate to the aims of the study, are they well described, and are necessary controls included? Choose an item.

Conclusions

Are the conclusions adequately supported by the data shown? Choose an item.

Reporting Standards

Does the manuscript adhere to the journal's guidelines on minimum standards of reporting? Choose an item.

Choose an item.

Statistics

Are you able to assess all statistics in the manuscript, including the appropriateness of statistical tests used? Choose an item.

Quality of Written English

Please indicate the quality of language in the manuscript: Choose an item.

Declaration of Competing Interests

Please complete a declaration of competing interests, considering the following questions:

- Have you in the past five years received reimbursements, fees, funding, or salary from an organisation that may in any way gain or lose financially from the publication of this manuscript, either now or in the future?
- Do you hold any stocks or shares in an organisation that may in any way gain or lose financially from the publication of this manuscript, either now or in the future?
- Do you hold or are you currently applying for any patents relating to the content of the manuscript?
- Have you received reimbursements, fees, funding, or salary from an organization that holds or has applied for patents relating to the content of the manuscript?
- Do you have any other financial competing interests?
- Do you have any non-financial competing interests in relation to this paper?

If you can answer no to all of the above, write 'I declare that I have no competing interests' below. If your reply is yes to any, please give details below.

I declare that I have no competing interests

I agree to the open peer review policy of the journal. I understand that my name will be included on my report to the authors and, if the manuscript is accepted for publication, my named report including any attachments I upload will be posted on the website along with the authors' responses. I agree for my report to be made available under an Open Access Creative Commons CC-BY license (http://creativecommons.org/licenses/by/4.0/). I understand that any comments which I do not wish to be included in my named report can be included as confidential comments to the editors, which will not be published.

Choose an item.

To further support our reviewers, we have joined with Publons, where you can gain additional credit to further highlight your hard work (see: https://publons.com/journal/530/gigascience). On publication of this paper, your review will be automatically added to Publons, you can then choose whether or not to claim your Publons credit. I understand this statement.

Yes Choose an item.
